# Supplementary material for: Metal Chelation Therapy and Parkinson’s Disease: A Critical Review on the Thermodynamics of Complex Formation between Relevant Metal Ions and Promising or Established Drugs
Source: Biomolecules. 2019 Jul 9;9(7):269. doi: 10.3390/biom9070269 (PMC6681387; doi:10.3390/biom9070269)
Supplement: Supplementary file 1 [file biomolecules-09-00269-s001.pdf]

Supporting information

Metal Chelation Therapy and Parkinson's Disease: A Critical Review on the Thermodynamics of Complex Formation Between Relevant Metal Ions and Promising or Established Drugs

Marianna Tosato and Valerio Di Marco*

Department of Chemical Sciences, via Marzolo 1, 35131 Padova, Italy. email: valerio.dimarco@unipd.it

Method and database used for the bibliografic search.

The used database was Web of Science (http://apps.webofknowledge.com/UA_GeneralSearch_input.do?product=UA&SID=D6Y2B9Fsbyg9IOPYezY&search_mode=GeneralSearch).

The year span was from 2014 until April 2019

The following subsequent bibliographic searches were performed (* = wildcard character):

1) anti *and* parkinson *and* drug*

2) parkinson *and* neuroprotective *and* agent*

3) parkinson *and* therapeutic *and* agent*

Spot cross-comparisons were performed with bibliographic results obtained in the databases Scopus (https://www.scopus.com/search/form.uri?display=basic) and Pubmed (https://www.ncbi.nlm.nih.gov/pubmed/)

TABLE S1

Compounds used, tested or proposed for the therapy against Parkinson’s disease, as obtained from a Literature survey in the year range 2014–2019 (April). For each substance, name(s), structure and latest reference(s) are given (if a reference is a review, it is explicitly indicated). Compounds highlighted in grey do not have metal chelation properties. Compounds highlighted in blue likely have metal chelation properties, but to the best of our knowledge their metal speciation has neither been studied nor can be estimated due to complicated and/or peptidic structure (in these cases structures were often omitted). These highlighted substances have not been further considered in the present review.

7DH	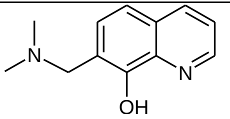	Iron Chelators and Antioxidants Regenerate Neuritic Tree and Nigrostriatal Fibers of MPP plus /MPTP-Lesioned Dopaminergic Neurons, Aguirre, P; Mena, NP; Carrasco, CM; Munoz, Y; Perez-Henriquez, P; Morales, RA; Cassels, BK; Mendez-Galvez, C; Garcia-Beltran, O; Gonzalez-Billault, C et al, PLOS ONE, 10, 12, Article Number: e0144848, DOI: 10.1371/journal.pone.0144848, DEC 14 2015
7MH	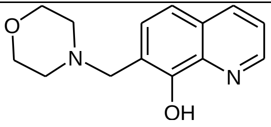	Iron Chelators and Antioxidants Regenerate Neuritic Tree and Nigrostriatal Fibers of MPP plus /MPTP-Lesioned Dopaminergic Neurons, Aguirre, P; Mena, NP; Carrasco, CM; Munoz, Y; Perez-Henriquez, P; Morales, RA; Cassels, BK; Mendez-Galvez, C; Garcia-Beltran, O; Gonzalez-Billault, C et al, PLOS ONE, 10, 12, Article Number: e0144848, DOI: 10.1371/journal.pone.0144848, DEC 14 2015
8A	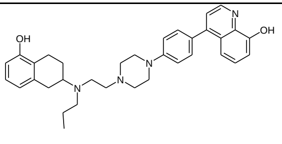 8A	A review on iron chelators as potential therapeutic agents for the treatment of Alzheimer's and Parkinson's diseases. Singh, YP; Pandey, A; Vishwakarma, S; Modi, G, Molecular diversity, DOI:10.1007/s11030-018-9878-4, 2018-Oct-06. **Document type: review**
8B	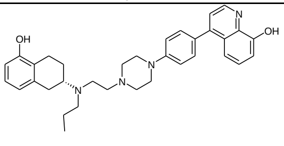 8B	A review on iron chelators as potential therapeutic agents for the treatment of Alzheimer's and Parkinson's diseases. Singh, YP; Pandey, A; Vishwakarma, S; Modi, G, Molecular diversity, DOI:10.1007/s11030-018-9878-4, 2018-Oct-06. **Document type: review**
8C	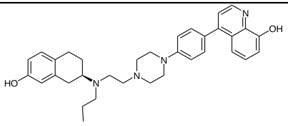 8C	A review on iron chelators as potential therapeutic agents for the treatment of Alzheimer's and Parkinson's diseases. Singh, YP; Pandey, A; Vishwakarma, S; Modi, G, Molecular diversity, DOI:10.1007/s11030-018-9878-4, 2018-Oct-06. **Document type: review**
8E	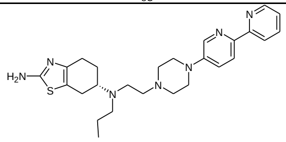 8E	A review on iron chelators as potential therapeutic agents for the treatment of Alzheimer's and Parkinson's diseases. Singh, YP; Pandey, A; Vishwakarma, S; Modi, G, Molecular diversity, DOI:10.1007/s11030-018-9878-4, 2018-Oct-06. **Document type: review**
8F	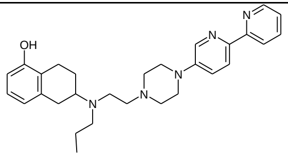 8F	A review on iron chelators as potential therapeutic agents for the treatment of Alzheimer's and Parkinson's diseases. Singh, YP; Pandey, A; Vishwakarma, S; Modi, G, Molecular diversity, DOI:10.1007/s11030-018-9878-4, 2018-Oct-06. **Document type: review**
AADC		Advances in non-dopaminergic treatments for Parkinson's disease. Stayte, S; Vissel, B ; FRONTIERS IN NEUROSCIENCE, 8, Article Number: 113, DOI: 10.3389/fnins.2014.00113, MAY 22 2014
Ac-[Phe(pI)(6), Nle(17)]PACAP(1-27)		Characterizations of a synthetic pituitary adenylate cyclase-activating polypeptide analog displaying potent neuroprotective activity and reduced in vivo cardiovascular side effects in a Parkinson's disease model, Lamine, A; Letourneau, M; Doan, ND; Maucotel, J; Couvineau, A; Vaudry, H; Chatenet, D; Vaudry, D; Fournier, A, NEUROPHARMACOLOGY, 108, 440-450, DOI: 10.1016/j.neuropharm.2015.05.014, SEP 2016
1-Acetyl-19,21-epoxy-15,16-dimethoxy aspidospermidine-17-ol	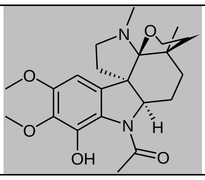	Identification of an aspidospermine derivative from borage extract as an anti-amyloid compound: A possible link between protein aggregation and antimalarial drugs, Kalhor, HR; Ashrafian, H, PHYTOCHEMISTRY, 140, 134-140, DOI: 10.1016/j.phytochem.2017.05.001, AUG 2017

Acetylcorynoline	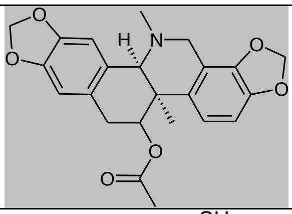	Acetylcorynoline attenuates dopaminergic neuron degeneration and alpha-synuclein aggregation in animal models of Parkinson's disease, Fu, RH; Wang, YC; Chen, CS; Tsai, RT; Liu, SP; Chang, WL; Lin, HL; Lu, CH; Wei, JR; Wang, ZW, et al, NEUROPHARMACOLOGY, 82, 108-120, DOI: 10.1016/j.neuropharm.2013.08.007, JUL 2014
N-acetylcysteine	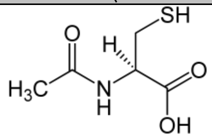	**1)** N-acetylcysteine protects against motor, optomotor and morphological deficits induced by 6- in zebrafish larvae, Benvenutti, R; Marcon, M; Reis, CG; Nery, LR; Miguel, C; Herrmann, AP; Vianna, MRM; Piato, A, PEERJ, 6, Article Number: e4957, DOI: 10.7717/peerj.4957, JUN 1 2018, **2)** 17 beta-Estradiol/N-Acetylcysteine Interaction Enhances the Neuroprotective Effect on Dopaminergic Neurons in the Weaver Model of Dopamine Deficiency, Botsakis, K; Theodoritsi, S; Grintzalis, K; Angelatou, F; Antonopoulos, I; Georgiou, CD; Margarity, M; Matsokis, NA; Panagopoulos, NT, NEUROSCIENCE, 320, 221-229, DOI: 10.1016/j.neuroscience.2016.01.068, APR 21 2016, **3) et cetera**
2-Acetyl-7-hydroxy-6-methoxy-1-methyl-1,2,3,4,-tetrahydroisoquinoline	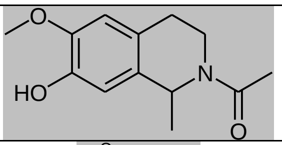	2-Acetyl-7-hydroxy-6-methoxy-1-methyl-1,2,3,4,-tetrahydroisoquinoline exhibits anti-inflammatory properties and protects the nigral dopaminergic neurons, Son, HJ; Han, SH; Lee, JA Lee, CS; Seo, JW; Chi, DY; Hwang, O, EUROPEAN JOURNAL OF PHARMACOLOGY, 771, 152-161, DOI: 10.1016/j.ejphar.2015.12.009, JAN 15 2016
Acetyl-L-carnitine	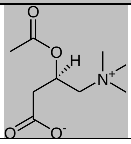	Redox-based therapeutics in neurodegenerative disease. McBean, GJ; Lopez, MG; Wallner, FK. BRITISH JOURNAL OF PHARMACOLOGY, 174, 12, 1750-1770, DOI: 10.1111/bph.13551, JUN 2017
Achyranthes bidentata polypeptides		**1)** Achyranthes bidentata polypeptide protects dopaminergic neurons from apoptosis induced by rotenone and 6-hydroxydopamine, Peng, S; Xu, L; Ma, JY; Gu, XS; Sun, C, NEURAL REGENERATION RESEARCH, 13, 11, 1981-1987, DOI: 10.4103/1673-5374.239446, NOV 2018, **2)** Achyranthes bidentata polypeptide protects dopaminergic neurons from apoptosis in Parkinson's disease models both in vitro and in vivo, Peng, S; Wang, CP; Ma, JY; Jiang, KT; Jiang, YH; Gu, XS; Sun, C; BRITISH JOURNAL OF PHARMACOLOGY, 175, 4, 631-643, DOI: 10.1111/bph.14110, FEB 2018
ACPT-I	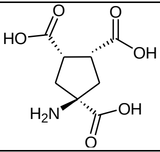	Neuroprotective effects of metabotropic glutamate receptor group II and III activators against MPP-induced cell death in human neuroblastoma SH-SY5Y cells: The impact of cell differentiation state, Jantas, D; Greda, A; Golda, S; Korostynski, M; Grygier, B; Roman, A; Pilc, A; Lason, W; NEUROPHARMACOLOGY, 83, 36-53, DOI: 10.1016/j.neuropharm.2014.03.019, AUG 2014
Acutilonine F	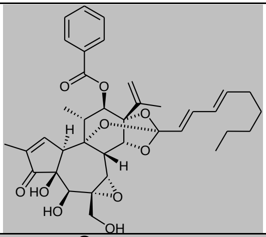	**1)** Daphnane and Phorbol Diterpenes, Anti-neuroinflammatory Compounds with Nurr1 Activation from the Roots and Stems of Daphne genkwa, Han, BS; Van Minh, N; Choi, HY; Byun, JS; Kim, WG, BIOLOGICAL & PHARMACEUTICAL BULLETIN,40, 12, 2205-2212, DEC 2017, **2)** Diterpenes: Advances in Neurobiological Drug Research, Islam, MT; da Silva, CB; de Alencar, MVOB (Oliveira Barros de Alencar, Marcus Vinicius); Paz, MFCJ; Almeida, FRD; Melo-Cavalcante, AAD (de Carvalho Melo-Cavalcante, Ana Amelia), PHYTOTHERAPY RESEARCH,30, 6, 915-928, DOI: 10.1002/ptr.5609, JUN 2016, **Document Type: Review**
N-Acyl Derivatives of 4-Phenoxyaniline	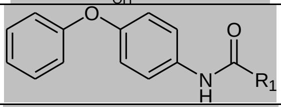	N-Acyl Derivatives of 4-Phenoxyaniline as Neuroprotective Agents, Barho, MT; Oppermann, S; Schrader, FC; Degenhardt, I; Elsasser, K; Wegscheid-Gerlach, C; Culmsee, C; Schlitzer, M; CHEMMEDCHEM, 9, 10, 2260-2273, DOI: 10.1002/cmdc.201402195, OCT 2014
N-Adamantyl-4-methylthiazol-2-amine	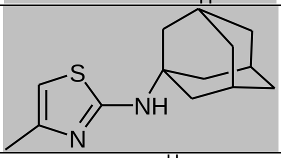	Anti-inflammatory mechanisms of N-adamantyl-4-methylthiazol-2-amine in lipopolysaccharide-stimulated BV-2 microglial cells, Kim, EA; Han, AR; Choi, J; Ahn, JY; Choi, SY; Cho, SW; INTERNATIONAL IMMUNOPHARMACOLOGY, 22, 1, 73-83, DOI: 10.1016/j.intimp.2014.06.022, SEP 2014
ADX88178	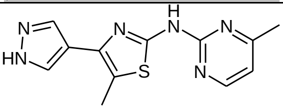	The Metabotropic Glutamate Receptor 4 Positive Allosteric Modulator ADX88178 Inhibits Inflammatory Responses in Primary Microglia, Ponnazhagan, R; Harms, AS; Thome, AD; Jurkuvenaite, A; Gogliotti, R; Niswender, CM; Conn, PJ; Standaert, DG; JOURNAL OF NEUROIMMUNE PHARMACOLOGY, 11, 2, 231-237, DOI: 10.1007/s11481-016-9655-z, JUN 2016
Affitope (PD01A)		**1)** Therapeutic strategies for Parkinson disease: beyond dopaminergic drugs.Charvin, D; Medori, R; Hauser, RA; Rascol, O. NATURE REVIEWS DRUG DISCOVERY, 17, 11, 804-822, DOI: 10.1038/nrd.2018.136, NOV 2018, **2)** Disease-Modifying Drugs in Parkinson's Disease, Park, A; Stacy, M; DRUGS, 75, 18, 2065-2071, DOI: 10.1007/s40265-015-0497-4, DEC 2015, **3)** Advances in Drug Development for Parkinson's Disease: Present Status. Reddy, DH; Misra, S; Medhi, B. PHARMACOLOGY, 93, 5-6, 260-271, DOI: 10.1159/000362419, 2014
Agmatine	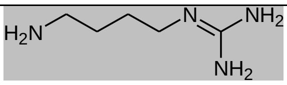	**1)** Neuroprotective effect of agmatine against oxidative stress and neuroinflammation in rotenone model of Parkinson's disease, El-Sayed, EK; Ahmed, AAE; El Morsy, EM; Nofal, S; HUMAN & EXPERIMENTAL TOXICOLOGY, 38, 2, 173-184, DOI: 10.1177/0960327118788139, FEB 2019, **2)**

		Neuroprotective Role of Agmatine in Neurological Diseases, Xu, WL; Gao, LS; Li, T; Shao, AW; Zhang, JM; CURRENT NEUROPHARMACOLOGY, 16, 9, 1296-1305, DOI: 10.2174/1570159X15666170808120633, 2018, **3) et cetera**
AK-918/42829299	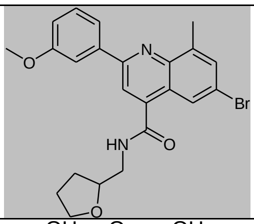	Virtual Screening against Phosphoglycerate Kinase 1 in Quest of Novel Apoptosis Inhibitors, Xia, J; Feng, B; Shao, QH; Yuan, YH; Wang, XS; Chen, NH; Wu, S; MOLECULES, 22, 6, Article Number: 1029, DOI: 10.3390/molecules22061029, JUN 2017
Alaternin	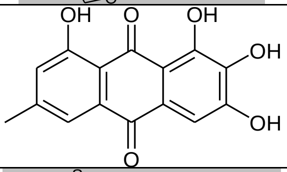	Recent advances in discovery and development of natural products as source for anti-Parkinson's disease lead compounds, Zhang, HJ; Bai, L; He, J; Zhong, L; Duan, XM; Ouyang, L; Zhu, YX; Wang, T; Shi, JY; Zhang, YW, EUROPEAN JOURNAL OF MEDICINAL CHEMISTRY,141, 257-272, DOI: 10.1016/j.ejmech.2017.09.068, DEC 1 2017, **Document Type:Review**
Albendazole	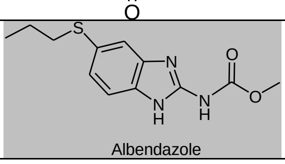 Albendazole	Antihelminthic Benzimidazoles Are Novel HIF Activators That Prevent Oxidative Neuronal Death via Binding to Tubulin, Aleyasin, H; Karuppagounder, SS; Kumar, A; Sleiman, S; Basso, M; Ma, T; Siddiq, A; Chinta, SJ; Brochier, C; Langley, B, et al, ANTIOXIDANTS & REDOX SIGNALING, 22, 2, 121-134, DOI: 10.1089/ars.2013.5595, JAN 10 2015
Albiglutide		Parkinson's Disease, Diabetes and Cognitive Impairment, Ashraghi, MR; Pagano, G; Polychronis, S; Niccolini, F; Politis, M, RECENT PATENTS ON ENDOCRINE METABOLIC & IMMUNE DRUG DISCOVERY,10, 1, 11-21, DOI: 10.2174/1872214810999160628105549, 2016, **Document Type: Review**
Alogliptin	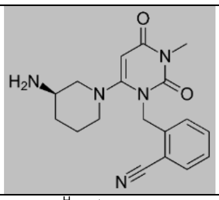	Parkinson's Disease, Diabetes and Cognitive Impairment, Ashraghi, MR; Pagano, G; Polychronis, S; Niccolini, F; Politis, M, RECENT PATENTS ON ENDOCRINE METABOLIC & IMMUNE DRUG DISCOVERY,10, 1, 11-21, DOI: 10.2174/1872214810999160628105549, 2016, **Document Type: Review**
Alvespimycin	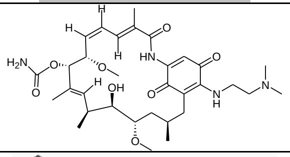	Discovery of the neuroprotective effects of alvespimycin by computational prioritization of potential anti-parkinson agents, Gao, L; Zhao, G; Fang, JS; Yuan, TY; Liu, AL; Du, GH; FEBS JOURNAL, 281, 4, 1110-1122, DOI: 10.1111/febs.12672, FEB 2014
AM-251	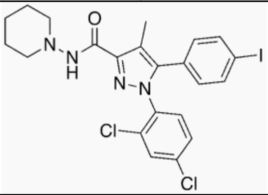	**1)** The role of cannabinoids and leptin in neurological diseases, Agar, E; ACTA NEUROLOGICA SCANDINAVICA, 132, 6, 371-380, DOI: 10.1111/ane.12411, DEC 2015, **Document Type:Review, 2)** Promising cannabinoid-based therapies for Parkinson's disease: motor symptoms to neuroprotection, More, SV; Choi, DK; MOLECULAR NEURODEGENERATION, 10, Article Number: 17, DOI: 10.1186/s13024-015-0012-0, APR 8 2015, **Document Type:Review, 3) et cetera**
Amantadine	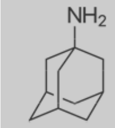	**1)** Current approaches to the treatment of Parkinson's Disease, Ellis, JM; Fell, MJ, BIOORGANIC & MEDICINAL CHEMISTRY LETTERS, 27, 18, 4247-4255, DOI: 10.1016/j.bmcl.2017.07.075, Published: SEP 15 2017**, 2)** Amantadine and the Risk of Dyskinesia in Patients with Early Parkinson's Disease: An Open-Label, Pragmatic Trial, Kim, A; Kim, YE; Yun, JY; Kim, HJ; Yang, HJ; Lee, WW; Shin, CW; Park, H; Jung, YJ; Kim, A et al, JOURNAL OF MOVEMENT DISORDERS, 11, 2, 65-71, DOI: 10.14802/jmd.18005, MAY 2018, **3)** Prevalence and Correlates of Anti-Parkinson Drug Use in a Nationally Representative Sample, Dahodwala, N; Willis, AW; Li, PX; Doshi, JA; MOVEMENT DISORDERS CLINICAL PRACTICE, 4, 3, 335-341, DOI: 10.1002/mdc3.12422, MAY-JUN 2017, **4) et cetera**
Ambroxol	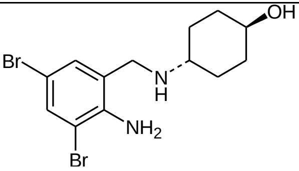	**1)** Ambroxol modulates 6-Hydroxydopamine-induced temporal reduction in Glucocerebrosidase enzymatic activity and Parkinson's disease symptoms, Mishra, A; Chandravanshi, LP; Trigun, SK; Krishnamurthy, S; BIOCHEMICAL PHARMACOLOGY, 155, 479-493, DOI: 10.1016/j.bcp.2018.07.028, SEP 2018, **2)** Combined beta-glucosylceramide and ambroxol hydrochloride in patients with Gaucher related Parkinson disease: From clinical observations to drug development, Ishay, Y; Zimran, A; Szer, J; Dinur, T; Ilana, Y; Arkadir, D; BLOOD CELLS MOLECULES AND DISEASES, 68, 117-120 Special : SI, DOI: 10.1016/j.bcmd.2016.10.028, FEB 2018, **3)** Current approaches to the treatment of Parkinson's Disease, Ellis, JM; Fell, MJ, BIOORGANIC & MEDICINAL CHEMISTRY LETTERS, 27, 18, 4247-4255, DOI: 10.1016/j.bmcl.2017.07.075, Published: SEP 15 2017
3-(7-Amino-5-(cyclohexylamino)-[1,2,4]triazolo[1,5-a][1,3,5]triazin-2-yl)-2-cyanoacrylamide	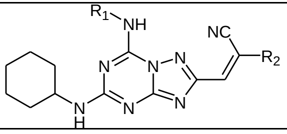	A Triazolotriazine-Based Dual GSK-3 beta/CK-1 delta Ligand as a Potential Neuroprotective Agent Presenting Two Different Mechanisms of Enzymatic Inhibition, Redenti, S; Marcovich, I; De Vita, T; Perez, C; De Zorzi, R; Demitri, N; Perez, DI; Bottegoni, G; Bisignano, P; Bissaro, M, et al, CHEMMEDCHEM, 14, 3, 310-314, DOI: 10.1002/cmdc.201800778, FEB 5 2019

(6aR)-11-Amino-N-propyl-noraporphine	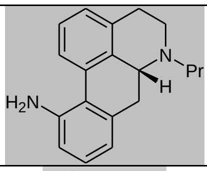	-11-Amino-N-propyl-noraporphine, a new dopamine D-2 and serotonin 5-HT1A dual agonist, elicits potent antiparkinsonian action and attenuates levodopa-induced dyskinesia in a 6-OHDA-lesioned rat model of Parkinson's disease, Zhao, R; Lu, WJ; Fang, X; Guo, L; Yang, Z; Ye, N; Zhao, JH; Liu, ZL; Jia, J; Zheng, LT et al, PHARMACOLOGY BIOCHEMISTRY AND BEHAVIOR, 124, 204-210, DOI: 10.1016/j.pbb.2014.06.011, SEP 2014
Aminoindan	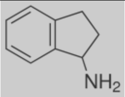	BDNF levels are increased by aminoindan and rasagiline in a double lesion model of Parkinson's disease, Ledreux, A; Boger, HA; Hinson, VK; Cantwell, K; Granholm, AC; BRAIN RESEARCH, 1631, 34-45, DOI: 10.1016/j.brainres.2015.11.028, JAN 15 2016
5-Aminoisoquinolin-1-one	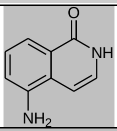	5-Aminoisoquinolin-1-one; a Water-Soluble Inhibitor of the PolyPolymerases; Threadgill, MD; CURRENT MEDICINAL CHEMISTRY, 22, 33, 3807-3829, DOI: 10.2174/0929867322666151002110602, 2015
2-Amino-8-[2-(4-morpholinyl)ethoxy]-4-phenyl-5H-indeno[1,2-d]pyrimidin-5-one	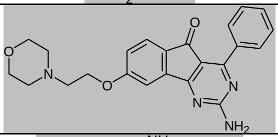	JNJ-40255293, a Novel Adenosine A/A Antagonist with Efficacy in Preclinical Models of Parkinson's Disease, Atack, JR; Shook, BC; Rassnick, S; Jackson, PF; Rhodes, K; Drinkenburg, WH; Ahnaou, A; te Riele, P; Langlois, X; Hrupka, B et al, ACS CHEMICAL NEUROSCIENCE, 5, 10, 1005-1019, DOI: 10.1021/cn5001606, OCT 2014
3-(2-Amino-6-phenylpyrimidin-4-yl) phenyl morpholine-4-carboxylate	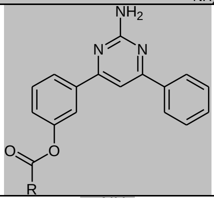	Carbamate substituted 2-amino-4,6-diphenylpyrimidines as adenosine receptor antagonists, Robinson, SJ; Petzer, JP; Rousseau, AL; Terre'Blanche, G; Petzer, A; Lourens, ACU; BIOORGANIC & MEDICINAL CHEMISTRY LETTERS, 26, 3, 734-738, DOI: 10.1016/j.bmcl.2016.01.004, FEB 1 2016
4-Aminopyridine/ Dalfampridine	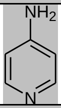	Advances in Drug Development for Parkinson's Disease: Present Status. Reddy, DH; Misra, S; Medhi, B. PHARMACOLOGY, 93, 5-6, 260-271, DOI: 10.1159/000362419, 2014, **Document Type:Review**
4-Aminopyridine derivatives	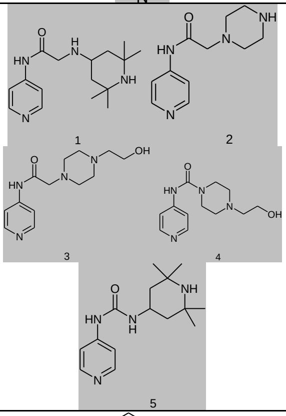	**1)** Design, synthesis, immunocytochemistry evaluation, and molecular docking investigation of several 4-aminopyridine derivatives as potential neuroprotective agents for treating Parkinson's disease, Li, SL; Wei, DY; Mao, Z; Chen, LG; Yan, XL; Li, Y; Dong, SJ; Wang, DH; BIOORGANIC CHEMISTRY, 73, 63-75, DOI: 10.1016/j.bioorg.2017.05.010, AUG 2017, **2)** Blockade of fast A-type and TEA-sensitive potassium channels provide an antiparkinsonian effect in a 6-OHDA animal model, Haghdoost-Yazdi, H; Piri, H; Najafipour, R; Faraji, A; Fraidouni, N; Dargahi, T; Heidari, MA; NEUROSCIENCES, 22, 1, 44-50, DOI: 10.17712/nsj.2017.1.20160266, JAN 2017
Aminothiazoles derivatives as SUMOylation activators	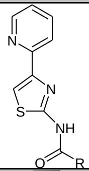	Small molecule SUMOylation activators are novel neuroprotective agents, Krajnak, K; Dahl, R, BIOORGANIC & MEDICINAL CHEMISTRY LETTERS,28, 3, 405-409, DOI: 10.1016/j.bmcl.2017.12.028, FEB 1 2018
Amitriptyline	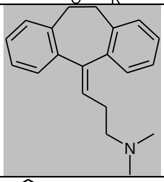	Imipramine and Amitriptyline Ameliorate the Rotenone Model of Parkinson's Disease in Rats, Kandil, EA; Abdelkader, NF; El-Sayeh, BM; Saleh, S; NEUROSCIENCE, 332, 26-37, DOI: 10.1016/j.neuroscience.2016.06.040, SEP 22 2016
AMN082	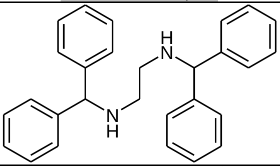	Neuroprotective effects of metabotropic glutamate receptor group II and III activators against MPP-induced cell death in human neuroblastoma SH-SY5Y cells: The impact of cell differentiation state, Jantas, D; Greda, A; Golda, S; Korostynski, M; Grygier, B; Roman, A; Pilc, A; Lason, W; NEUROPHARMACOLOGY, 83, 36-53, DOI: 10.1016/j.neuropharm.2014.03.019, AUG 2014
Amodiaquine	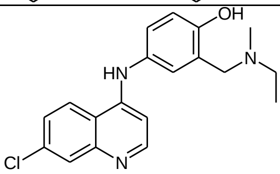	**1)** Novel anti-adipogenic activity of anti-malarial amodiaquine through suppression of PPAR gamma activity, Kim, TH; Kim, HK; Hwang, ES; ARCHIVES OF PHARMACAL RESEARCH, 40, 11, 1336-1343, DOI: 10.1007/s12272-017-0965-3, NOV 2017, **2)** New phenylaniline derivatives as modulators of amyloid protein precursor metabolism, Gay, M; Carato, P; Coevoet, M; Renault, N; Larchanche, PE; Barczyk, A; Yous, S; Buee, L; Sergeant, N; Melnyk, P; BIOORGANIC & MEDICINAL CHEMISTRY, 26, 8, 2151-2164, DOI: 10.1016/j.bmc.2018.03.016, MAY 1 2018

AN-465/41520984	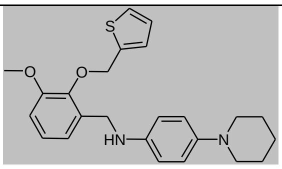	Virtual Screening against Phosphoglycerate Kinase 1 in Quest of Novel Apoptosis Inhibitors, Xia, J; Feng, B; Shao, QH; Yuan, YH; Wang, XS; Chen, NH; Wu, S; MOLECULES, 22, 6, Article Number: 1029, DOI: 10.3390/molecules22061029, JUN 2017
Andrographolide	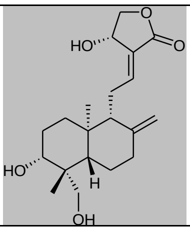	Andrographolide, a diterpene lactone from Andrographis paniculata and its therapeutic promises in cancer, Islam, MT; Ali, ES; Uddin, SJ; Islam, MA; Shaw, S; Khan, IN; Saravi, SSS; Ahmad, S; Rehman, S; Gupta, VK, et al, CANCER LETTERS, 420, 129-145, DOI: 10.1016/j.canlet.2018.01.074, 2018, **Document Type:Review**
Angiotensin(1-7)		Neuroprotective effect of angiotensin- against rotenone-induced oxidative damage in CATH.a neurons, Zhou, Y; Li, M; Zhu, DL; Jiang, T; Gao, Q; Tang, XH; Zhang, SG; Lu, J; Zhang, YD; TOXICOLOGY IN VITRO, 50, 373-382, DOI: 10.1016/j.tiv.2018.04.005, AUG 2018
ANLE 138b	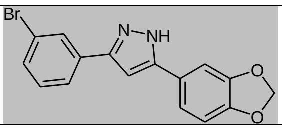	Current and experimental treatments of Parkinson disease: A guide for neuroscientists. Oertel, W; Schulz, JB. JOURNAL OF NEUROCHEMISTRY, 139, 325-337, Supplement: 1, DOI: 10.1111/jnc.13750, OCT 2016, **Document Type: Review**
Annexin A1		Overexpression of Annexin A1 Suppresses Pro-Inflammatory Factors in PC12 Cells Induced by 1-Methyl-4-Phenylpyridinium, Kiani-Esfahani, A; Sheykhshabani, SK; Peymani, M; Hashemi, MS; Ghaedi, K; Nasr-Esfahani, MH; CELL JOURNAL, 18, 2, SUM 2016
Antagonist of the A(2A) adenosine receptor - derivative 49	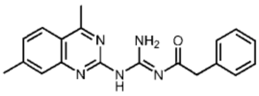	Discovery of Novel and Selective Adenosine A Receptor Antagonists for Treating Parkinson's Disease through Comparative Structure-Based Virtual Screening, Tian, S; Wang, X; Li, LL; Zhang, XH; Li, YY; Zhu, F; Hou, TJ; Zhen, XC; JOURNAL OF CHEMICAL INFORMATION AND MODELING, 57, 6, 1474-1487, DOI: 10.1021/acs.jcim.7b00188, JUN 2017
Antagonist of the A(2A) adenosine receptor - other derivatives	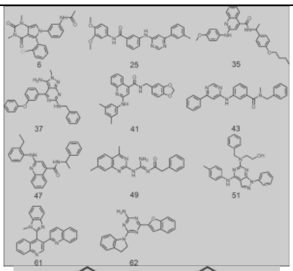 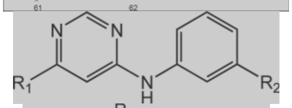 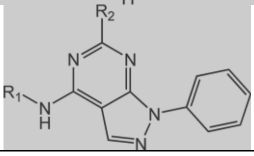	Discovery of Novel and Selective Adenosine A Receptor Antagonists for Treating Parkinson's Disease through Comparative Structure-Based Virtual Screening, Tian, S; Wang, X; Li, LL; Zhang, XH; Li, YY; Zhu, F; Hou, TJ; Zhen, XC; JOURNAL OF CHEMICAL INFORMATION AND MODELING, 57, 6, 1474-1487, DOI: 10.1021/acs.jcim.7b00188, JUN 2017
APAU	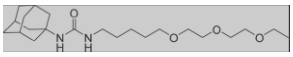	Soluble epoxide hydrolase inhibitor, APAU, protects dopaminergic neurons against rotenone induced neurotoxicity: Implications for Parkinson's disease, Lakkappa, N; Krishnamurthy, PT; Pandareesh, MD; Hammock, BD; Hwang, SH; NEUROTOXICOLOGY, 70, 135-145, DOI: 10.1016/j.neuro.2018.11.010, JAN 2019
Apigenin	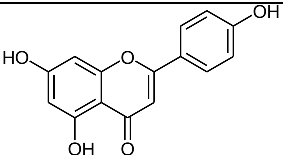	**1)** Flavonoids and its Neuroprotective Effects on Brain Ischemia and Neurodegenerative Diseases, Putteeraj, M; Lim, WL; Teoh, SL; Yahaya, MF, CURRENT DRUG TARGETS,19, 14, 1710-1720, DOI: 10.2174/1389450119666180326125252, 2018, **Document Type:Review**, **2)** Apigenin as neuroprotective agent: Of mice and men, Nabavi, SF; Khan, H; D'onofrio, G; Samec, D; Shirooie, S; Dehpour, AR; Arguelles, S; Habtemariam, S; Sobarzo-Sanchez, E, PHARMACOLOGICAL RESEARCH, 128, 359-365, DOI: 10.1016/j.phrs.2017.10.008, FEB 2018, **Document Type:Review, 3)** Protective role of apigenin on rotenone induced rat model of Parkinson's disease: Suppression of neuroinflammation and oxidative stress mediated apoptosis, Anusha, C; Sumathi, T; Joseph, LD; CHEMICO-BIOLOGICAL INTERACTIONS, 269, 67-79, DOI: 10.1016/j.cbi.2017.03.016, MAY 1 2017, **4)** Health functionality of apigenin: A review, Ali, F; Rahul; Naz, F; Jyoti, S; Siddique, YH; INTERNATIONAL JOURNAL OF FOOD PROPERTIES, 20, 6, 1197-1238, DOI: 10.1080/10942912.2016.1207188, 2017, **Document Type:Review, 5) et cetera**
Aplindore (SLS 006)	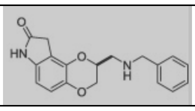	Current approaches to the treatment of Parkinson's Disease, Ellis, JM; Fell, MJ, BIOORGANIC & MEDICINAL CHEMISTRY LETTERS, 27, 18, 4247-4255, DOI: 10.1016/j.bmcl.2017.07.075, Published: SEP 15 2017

Apocynin	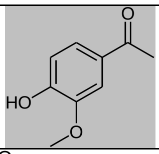	Redox-based therapeutics in neurodegenerative disease. McBean, GJ; Lopez, MG; Wallner, FK. BRITISH JOURNAL OF PHARMACOLOGY, 174, 12, 1750-1770, DOI: 10.1111/bph.13551, JUN 2017, **Document Type:Review**
Apomorphine	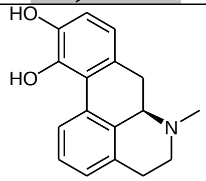	**1)** Intranasal administration of sodium dimethyldithiocarbamate induces motor deficits and dopaminergic dysfunction in mice, Mack, JM; Moura, TM; Lanznaster, D; Bobinski, F; Massari, CM; Sampaio, TB; Schmitz, AE; Souza, LF; Walz, R; Tasca, CI et al, NEUROTOXICOLOGY, 66, 107-120, DOI: 10.1016/j.neuro.2018.03.011, MAY 2018, **2)** Duration of drug action of dopamine D2 agonists in mice with 6-hydroxydopamine-induced lesions, Tsuchioka, A; Oana, F; Suzuki, T; Yamauchi, Y; Ijiro, T; Kaidoh, K; Hiratochi, M; NEUROREPORT, 26, 18, 1126-1132, DOI: 10.1097/WNR.0000000000000484, DEC 16 2015, **3) et cetera**
Arabinogalactan protein (AGP)		A generic class of amyloid fibril inhibitors, Ow, SY; Bekard, I; Blencowe, A; Qiao, GG; Dunstan, DE; JOURNAL OF MATERIALS CHEMISTRY B, 3, 7, 1350-1359, DOI: 10.1039/c4tb01762e, 2015
L-Arginine	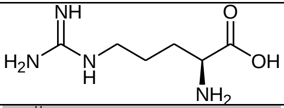	Effects of L-arginine pre-treatment in 1-methyl-4-phenyl-1,2,3,6-tetrahydropyridine-induced Parkinson's diseases in Balb/c mice, Hami, J; Hosseini, M; Shahi, S; Lotfi, N; Talebi, A; Afshar, M; IRANIAN JOURNAL OF NEUROLOGY, 14, 4, 195-203, FAL 2015
Aripiprazole	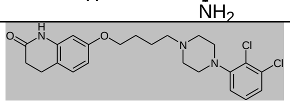	Dual Ligands Targeting Dopamine D-2 and Serotonin 5-HT1A Receptors as New Antipsychotical or Anti-Parkinsonian Agents, Ye, N; Song, ZL; Zhang, A; CURRENT MEDICINAL CHEMISTRY, 21, 4, 437-457, FEB 2014, **Document Type:Review**
Aromadendrin	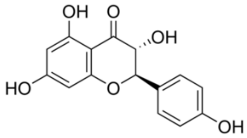	Flavonoids and its Neuroprotective Effects on Brain Ischemia and Neurodegenerative Diseases, Putteeraj, M; Lim, WL; Teoh, SL; Yahaya, MF, CURRENT DRUG TARGETS,19, 14, 1710-1720, DOI: 10.2174/1389450119666180326125252, 2018, **Document Type:Review**
Artesunate	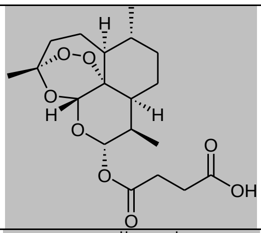	Neuroprotective Effect of Artesunate in Experimental Model of Traumatic Brain Injury, Gugliandolo, E; D'Amico, R; Cordaro, M; Fusco, R; Siracusa, R; Crupi, R; Impellizzeri, D; Cuzzocrea, S; Di Paola, R; FRONTIERS IN NEUROLOGY, 9, Article Number: 590, DOI: 10.3389/fneur.2018.00590, JUL 31 2018
Arylalkenylpropargylamines	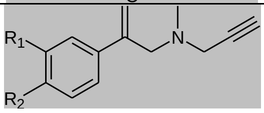	Novel Arylalkenylpropargylamines as Neuroprotective, Potent, and Selective Monoamine Oxidase B Inhibitors for the Treatment of Parkinson's Disease, Huleatt, PB; Khoo, ML; Chua, YY; Tan, TW; Liew, RS; Balogh, B; Deme, R; Goloncser, F; Magyar, K; Sheela, DP, et al, JOURNAL OF MEDICINAL CHEMISTRY, 58, 3, 1400-1419, DOI: 10.1021/jm501722s, FEB 12 2015
3-Arylcoumarin derivatives	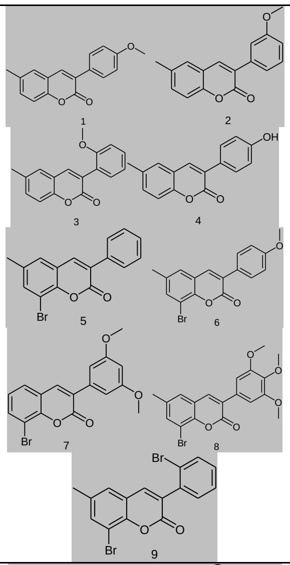	Quantum chemical analysis of potential anti-Parkinson agents, Razzaghi-Asla, N; Shahabipour, S; Ebadi, A; Bagheri, A; JOURNAL OF CHEMICAL SCIENCES, 127, 7, 1211-1220, DOI: 10.1007/s12039-015-0889-8, JUL 2015
16-Arylidene steroidal derivatives	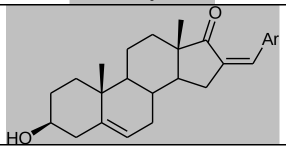	Investigations on 16-Arylideno Steroids as a New Class of Neuroprotective Agents for the Treatment of Alzheimer's and Parkinson's Diseases, Singh, R; Bansal, R; ACS CHEMICAL NEUROSCIENCE, 8, 1, 186-200, DOI:10.1021/acschemneuro.6b00313, JAN 2017

	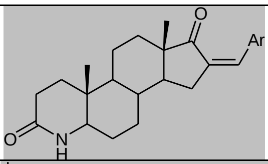	
α- and β-Asarone	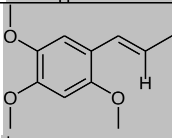 α-asarone 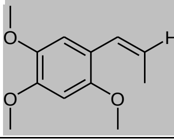 β-asarone	**1)** Pharmacology and toxicology of alpha- and beta-Asarone: A review of preclinical evidence, Chellian, R; Pandy, V; Mohamed, Z; PHYTOMEDICINE, 32, 41-58, DOI: 10.1016/j.phymed.2017.04.003, AUG 15 2017, **Document Type:Review, 2)** Neuroprotective Effects of beta-Asarone Against 6-Hydroxy Dopamine-Induced Parkinsonism via JNK/Bcl-2/Beclin-1 Pathway, Zhang, S; Gui, XH; Huang, LP; Deng, MZ; Fang, RM; Ke, XH; He, YP; Li, L; Fang, YQ; MOLECULAR NEUROBIOLOGY, 53, 1, 83-94, DOI: 10.1007/s12035-014-8950-z, JAN 2016, **3) et cetera**
Ascorbic acid	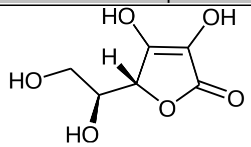	**1)** Liquid levodopa-carbidopa in advanced Parkinson's disease with motor complications, Yang, HJ; Ehm, G; Kim, YE; Yun, JY; Lee, WW; Kim, A; Kim, HJ; Jeon, B; JOURNAL OF THE NEUROLOGICAL SCIENCES, 377, 6-11, DOI: 10.1016/j.jns.2017.03.039, JUN 15 2017, **2)** Quantifying L-ascorbic acid-driven inhibitory effect on amyloid fibrillation, Lee, W; Kim, I; Lee, SW; Lee, H; Lee, G; Kim, S; Lee, SW; Yoon, DS; MACROMOLECULAR RESEARCH, 24, 10, 868-873, DOI: 10.1007/s13233-016-4126-1, OCT 2016
ASI-1	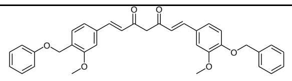	Development of Multifunctional Molecules as Potential Therapeutic Candidates for Alzheimer's Disease, Parkinson's Disease, and Amyotrophic Lateral Sclerosis in the Last Decade. Savelieff, MG; Nam, G; Kang, J; Lee, HJ; Lee, M; Lim, MH. CHEMICAL REVIEWS, 119, 2, 1221-1322, DOI: 10.1021/acs.chemrev.8b00138, JAN 23 2019, **Document Type:Review**
ASI-5	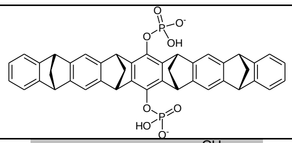	Development of Multifunctional Molecules as Potential Therapeutic Candidates for Alzheimer's Disease, Parkinson's Disease, and Amyotrophic Lateral Sclerosis in the Last Decade. Savelieff, MG; Nam, G; Kang, J; Lee, HJ; Lee, M; Lim, MH. CHEMICAL REVIEWS, 119, 2, 1221-1322, DOI: 10.1021/acs.chemrev.8b00138, JAN 23 2019, **Document Type:Review**
Asiatic acid	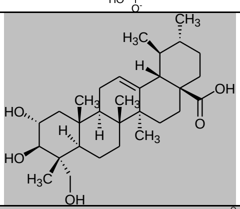	Neuroprotective effect of asiatic acid on rotenone-induced mitochondrial dysfunction and oxidative stress-mediated apoptosis in differentiated SH-SYS5Y cells, Nataraj, J; Manivasagam, T; Thenmozhi, AJ; Essa, MM; NUTRITIONAL NEUROSCIENCE, 20, 6, 351-359, DOI: 10.1080/1028415X.2015.1135559, 2017
Astaxanthin	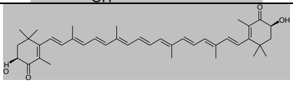	**1)** Astaxanthin attenuates neurotoxicity in a mouse model of Parkinson's disease, Grimmig, B; Daly, L; Hudson, C; Nash, KR; Bickford, PC; FUNCTIONAL FOODS IN HEALTH AND DISEASE, 7, 8, 562-576, DOI: 10.31989/ffhd.v7i8.352, AUG 2017, **2)** Neuroprotective Properties of the Marine Carotenoid Astaxanthin and Omega-3 Fatty Acids, and Perspectives for the Natural Combination of Both in Krill Oil, Barros, MP; Poppe, SC; Bondan, EF; NUTRIENTS, 6, 3, 1293-1317, DOI: 10.3390/nu6031293, MAR 2014, **Document Type:Review, 3)** Immunomodulators as Therapeutic Agents in Mitigating the Progression of Parkinson's Disease, Grimmig, B; Morganti, J; Nash, K; Bickford, PC; BRAIN SCIENCES, 6, 4, Article Number: 41, DOI: 10.3390/brainsci6040041, DEC 2016, **Document Type:Review**
Astilbin	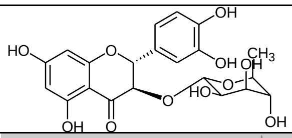	Neuroprotective effects of Astilbin on MPTP-induced Parkinson's disease mice: Glial reaction, alpha-synuclein expression and oxidative stress, Zhu, YL; Sun, MF; Jia, XB; Cheng, K; Xu, YD; Zhou, ZL; Zhang, PH; Qiao, CM; Cui, C; Chen, X et al, INTERNATIONAL IMMUNOPHARMACOLOGY, 66, 19-27, DOI: 10.1016/j.intimp.2018.11.004, JAN 2019
Astragaloside IV	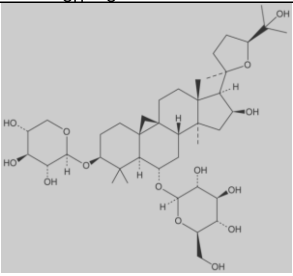	**1)** Neuroprotective effects of astragaloside IV on Parkinson disease models of mice and primary astrocytes, Xia, L; Guo, DX; Chen, B; EXPERIMENTAL AND THERAPEUTIC MEDICINE, 14, 6, 5569-5575, DOI: 10.3892/etm.2017.5238, DEC 2017, **2)** Astragaloside IV rescues MPP+-induced mitochondrial dysfunction through upregulation of methionine sulfoxide reductase A, Liu, Y; Chong, L; Li, XQ; Tang, P; Liu, P; Hou, C; Zhang, X; Li, R; EXPERIMENTAL AND THERAPEUTIC MEDICINE, 14, 3, 2650-2656 Part: B, DOI: 10.3892/etm.2017.4834, SEP 2017, **3)** Recent advances in discovery and development of natural products as source for anti-Parkinson's disease lead compounds, Zhang, HJ; Bai, L; He, J; Zhong, L; Duan, XM; Ouyang, L; Zhu, YX; Wang, T; Shi, JY; Zhang, YW, EUROPEAN JOURNAL OF MEDICINAL CHEMISTRY,141, 257-272, DOI: 10.1016/j.ejmech.2017.09.068, DEC 1 2017, **Document Type:Review**
AT- 051/43421517	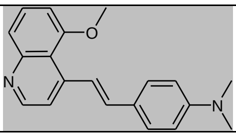	Virtual Screening against Phosphoglycerate Kinase 1 in Quest of Novel Apoptosis Inhibitors, Xia, J; Feng, B; Shao, QH; Yuan, YH; Wang, XS; Chen, NH; Wu, S; MOLECULES, 22, 6, Article Number: 1029, DOI: 10.3390/molecules22061029, JUN 2017

Atomoxetine	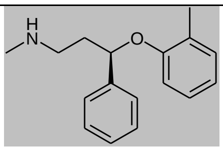	Treatment with the noradrenaline re-uptake inhibitor atomoxetine alone and in combination with the alpha 2-adrenoceptor antagonist idazoxan attenuates loss of dopamine and associated motor deficits in the LPS inflammatory rat model of Parkinson's disease, Yssel, JD; O'Neill, E; Nolan, YM; Connor, TJ; Harkin, A; BRAIN BEHAVIOR AND IMMUNITY, 69, 456-469, DOI: 10.1016/j.bbi.2018.01.004, MAR 2018
Atorvastatin	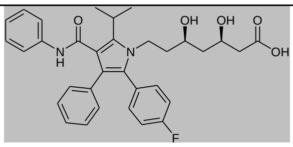	**1)** Atorvastatin Prevents Early Oxidative Events and Modulates Inflammatory Mediators in the Striatum Following Intranasal 1-Methyl-4-phenyl-1,2,3,6-tetrahydropyridine Administration in Rats, Marques, NF; Castro, AA; Mancini, G; Rocha, FL; Santos, ARS; Prediger, RD; De Bem, AF; Tasca, CI; NEUROTOXICITY RESEARCH, 33, 3, 549-559, DOI: 10.1007/s12640-017-9840-8, APR 2018, **2)** The beneficial effects of HMG-CoA reductase inhibitors in the processes of neurodegeneration, Saravi, SSS; Saravi, SSS; Arefidoust, A; Dehpour, AR, METABOLIC BRAIN DISEASE,32, 4, 949-965, DOI: 10.1007/s11011-017-0021-5, AUG 2017, **Document Type:Review**, **3)** Neuroprotective mechanisms of statins in neurodegenerative diseases, Yan, JQ; Sun, JC; Li, XM; Zhai, MM; Jia, XF, INTERNATIONAL JOURNAL OF CLINICAL AND EXPERIMENTAL MEDICINE,9, 6, 9799-9805, 2016, **Document Type:Review**
Atractylenolide-I	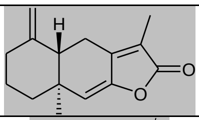	Neuroprotective Role of Atractylenolide-I in an In Vitro and In Vivo Model of Parkinson's Disease, More, S; Choi, DK; NUTRIENTS, 9, 5, Article Number: 451, DOI: 10.3390/nu9050451, MAY 2017
Atropine	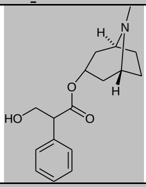	Cholinergic manipulation of motor disability and L-DOPA-induced dyskinesia in 1-methyl-4-phenyl-1,2,3,6-tetrahydropyridine -treated common marmosets, Jackson, MJ; Swart, T; Pearce, RKB; Jenner, P; JOURNAL OF NEURAL TRANSMISSION, 121, 2, 163-169, DOI: 10.1007/s00702-013-1082-1, FEB 2014
Auranofin	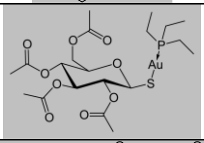	Gold drug auranofin could reduce neuroinflammation by inhibiting microglia cytotoxic secretions and primed respiratory burst, Madeira, JM; Bajwa, E; Stuart, MJ; Hashioka, S; Klegeris, A; JOURNAL OF NEUROIMMUNOLOGY, 276, 1-2, 71-79, DOI: 10.1016/j.jneuroim.2014.08.615, NOV 15 2014
Auraptene derivatives	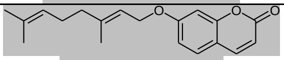 AUR (Auraptene) 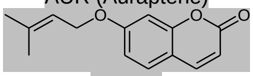 7-IP (7-isopentenyloxycoumarin) 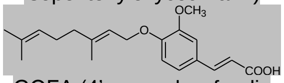 GOFA (4’-geranyloxyferulic acid)	Auraptene and Other Prenyloxyphenylpropanoids Suppress Microglial Activation and Dopaminergic Neuronal Cell Death in a Lipopolysaccharide-Induced Model of Parkinson's Disease, Okuyama, S; Semba, T; Toyoda, N; Epifano, F; Genovese, S; Fiorito, S; Taddeo, VA; Sawamoto, A; Nakajima, M; Furukawa, Y, INTERNATIONAL JOURNAL OF MOLECULAR SCIENCES,17, 10, Article Number: 1716, DOI: 10.3390/ijms17101716, OCT 2016
AVN-101	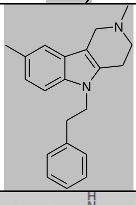	AVN-101: A Multi-Target Drug Candidate for the Treatment of CNS Disorders, Ivachtchenko, AV; Lavrovsky, Y; Okun, I; JOURNAL OF ALZHEIMERS DISEASE, 53, 2, 583-620, DOI: 10.3233/JAD-151146, 2016
AZ12216052 (2-[(4-bromophenyl)methylsulfanyl]-~{N}-(4-butan-2-ylphenyl)acetamide)	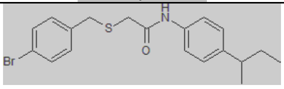	Neuroprotective effects of metabotropic glutamate receptor group II and III activators against MPP-induced cell death in human neuroblastoma SH-SY5Y cells: The impact of cell differentiation state, Jantas, D; Greda, A; Golda, S; Korostynski, M; Grygier, B; Roman, A; Pilc, A; Lason, W; NEUROPHARMACOLOGY, 83, 36-53, DOI: 10.1016/j.neuropharm.2014.03.019, AUG 2014
AZD3241 (Verdiperstat)	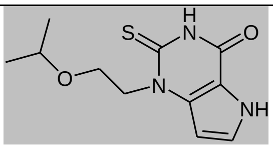	**1)** Current approaches to the treatment of Parkinson's Disease, Ellis, JM; Fell, MJ, BIOORGANIC & MEDICINAL CHEMISTRY LETTERS, 27, 18, 4247-4255, DOI: 10.1016/j.bmcl.2017.07.075, Published: SEP 15 2017**, 2)** The ongoing pursuit of neuroprotective therapies in Parkinson disease, Athauda, D; Foltynie, T; NATURE REVIEWS NEUROLOGY, 11, 1, 25-40, DOI: 10.1038/nrneurol.2014.226, JAN 2015, **Document Type:Review**
AZD5904	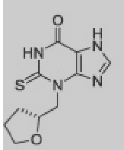	**1)** Current approaches to the treatment of Parkinson's Disease, Ellis, JM; Fell, MJ, BIOORGANIC & MEDICINAL CHEMISTRY LETTERS, 27, 18, 4247-4255, DOI: 10.1016/j.bmcl.2017.07.075, Published: SEP 15 2017**, 2)** The ongoing pursuit of neuroprotective therapies in Parkinson disease, Athauda, D; Foltynie, T; NATURE REVIEWS NEUROLOGY, 11, 1, 25-40, DOI: 10.1038/nrneurol.2014.226, JAN 2015, **Document Type:Review**

Azilsartan	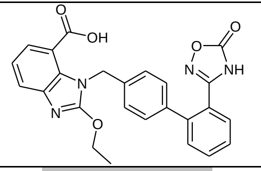	Azilsartan ameliorates apoptosis of dopaminergic neurons and rescues characteristic parkinsonian behaviors in a rat model of Parkinson's disease, Gao, Q; Ou, Z; Jiang, T; Tian, YY; Zhou, JS; Wu, L; Shi, JQ; Zhang, YD; ONCOTARGET, 8, 15, 24099-24109, DOI: 10.18632/oncotarget.15732, APR 11 2017
B12 (4-methyl-7-hydroxy-8-(1-hydroxyethyl) coumarin)	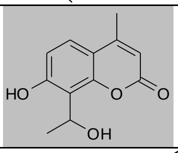	Quantitative proteomics study of the neuroprotective effects of B12 on hydrogen peroxide-induced apoptosis in SH-SY5Y cells, Zhong, LJ; Zhou, JT; Chen, X; Lou, YX; Liu, D; Zou, XJ; Yang, B; Yin, YX; Pan, Y; SCIENTIFIC REPORTS, 6, Article Number: 22635, DOI: 10.1038/srep22635, MAR 8 2016
Baicalein	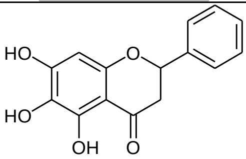	**1)** Formulation and anti-neurotoxic activity of baicalein-incorporating neutral nanoliposome, Aliakbari, F; Shabani, AA; Bardania, H; Mohammad-Beigi, H; Marvian, AT; Esmatabad, FD; Vafaei, AA; Shojaosadati, SA; Saboury, AA; Christiansen, G, et al, COLLOIDS AND SURFACES B-BIOINTERFACES, 161, 578-587, DOI: 10.1016/j.colsurfb.2017.11.023, JAN 1 2018, **2)** Baicalein as a potent neuroprotective agent: A review, Sowndhararajan, K; Deepa, P; Kim, M; Park, SJ; Kim, S; BIOMEDICINE & PHARMACOTHERAPY, 95, 1021-1032, DOI: 10.1016/j.biopha.2017.08.135, NOV 2017, **Document Type:Review, 3)** Pluronic P85/F68 Micelles of Baicalein Could Interfere with Mitochondria to Overcome MRP2-Mediated Efflux and Offer Improved Anti-Parkinsonian Activity, Chen, TK; Li, Y; Li, CW; Yi, X; Wang, RB; Lee, SMY; Zheng, Y; MOLECULAR PHARMACEUTICS, 14, 10, 3331-3342, DOI: 10.1021/acs.molpharmaceut.7b00374, OCT 2017, **4) et cetera**
Bavachin	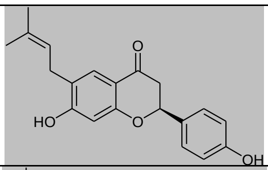	Evaluation of the Inhibitory Effects of Bavachinin and Bavachin on Human Monoamine Oxidases A and B, Zarmouh, NO; Mazzio, EA; Elshami, FM; Messeha, SS; Eyunni, SVK; Soliman, KFA; EVIDENCE-BASED COMPLEMENTARY AND ALTERNATIVE MEDICINE, Article Number: 852194, DOI: 10.1155/2015/852194, 2015
Bavachinin	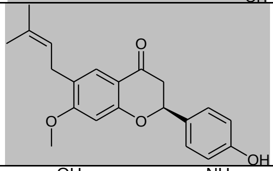	Evaluation of the Inhibitory Effects of Bavachinin and Bavachin on Human Monoamine Oxidases A and B, Zarmouh, NO; Mazzio, EA; Elshami, FM; Messeha, SS; Eyunni, SVK; Soliman, KFA; EVIDENCE-BASED COMPLEMENTARY AND ALTERNATIVE MEDICINE, Article Number: 852194, DOI: 10.1155/2015/852194, 2015
Benserazide	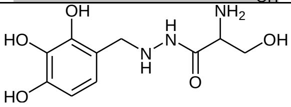	**1)** Molecular basis of the inhibition and disaggregation of thermally-induced amyloid fibrils of human serum albumin by an anti-Parkinson's drug, benserazide hydrochloride, Chandel, TI; Masroor, A; Siddiqi, MK; Siddique, IA; Jahan, I; Ali, M; Nayeem, SM; Uversky, VN; Khan, RH; JOURNAL OF MOLECULAR LIQUIDS, 278, 553-567, DOI: 10.1016/j.molliq.2018.12.127, MAR 15 2019, **2)** A multiparametric analysis of the synergistic impact of anti-Parkinson's drugs on the fibrillation of human serum albumin, Chandel, TI; Zaidi, N; Zaman, M; Jahan, I; Masroor, A; Siddique, IA; Nayeem, SM; Ali, M; Uversky, VN; Khan, RH; BIOCHIMICA ET BIOPHYSICA ACTA-PROTEINS AND PROTEOMICS, 1867, 3, 275-285, DOI: 10.1016/j.bbapap.2018.10.003, MAR 2019
7H-Benzo, perimidin-7-one derivatives (R_6 = H or OCH_3)	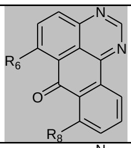	A Medical Approach to the Monoamine Oxidase Inhibition by Using 7H-benzo, perimidin-7-one Derivatives, Bacho, M; Coelho-Cerqueira, E; Follmer, C; Nabavi, SM; Rastrelli, L; Uriarte, E; Sobarzo-Sanchez, E; CURRENT TOPICS IN MEDICINAL CHEMISTRY, 17, 4, 489-497, DOI: 10.2174/1568026616666160824120929, 2017, **Document Type:Review**
7H-Benzo, perimidin-7-one derivatives (R_6 = OH)	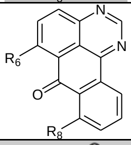	A Medical Approach to the Monoamine Oxidase Inhibition by Using 7H-benzo, perimidin-7-one Derivatives, Bacho, M; Coelho-Cerqueira, E; Follmer, C; Nabavi, SM; Rastrelli, L; Uriarte, E; Sobarzo-Sanchez, E; CURRENT TOPICS IN MEDICINAL CHEMISTRY, 17, 4, 489-497, DOI: 10.2174/1568026616666160824120929, 2017, **Document Type:Review**
Benzopyran derivatives	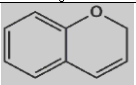	Oxidative Stress and Neurodegenerative Diseases: Looking for a Therapeutic Solution Inspired on Benzopyran Chemistry, Gaspar, A; Milhazes, N; Santana, L; Uriarte, E; Borges, F; Matos, MJ; CURRENT TOPICS IN MEDICINAL CHEMISTRY, 15, 5, 432-445, DOI: 10.2174/1568026614666141229124141, 2015, **Document Type:Review**
4H-1-Benzopyran-4-one	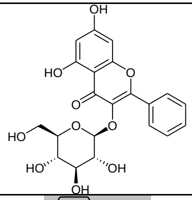	Structural Exploration of Synthetic Chromones as Selective MAO-B Inhibitors: A Mini Review, Mathew, B; Mathew, GE; Petzer, JP; Petzer, A; COMBINATORIAL CHEMISTRY & HIGH THROUGHPUT SCREENING, 20, 6, 522-532, DOI: 10.2174/1386207320666170227155517, 2017, **Document Type:Review**
Benzothiazole Derivatives as SUMOylation activators	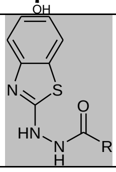	Small molecule SUMOylation activators are novel neuroprotective agents, Krajnak, K; Dahl, R, BIOORGANIC & MEDICINAL CHEMISTRY LETTERS,28, 3, 405-409, DOI: 10.1016/j.bmcl.2017.12.028, FEB 1 2018

2H-1,3-Benzoxathiol-2-one derivatives	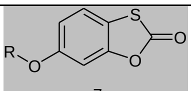 7	Inhibition of monoamine oxidase by benzoxathiolone analogues, Mostert, S; Petzer, A; Petzer, JP; BIOORGANIC & MEDICINAL CHEMISTRY LETTERS, 26, 4, 1200-1204, DOI: 10.1016/j.bmcl.2016.01.034, FEB 15 2016
Benzatropine	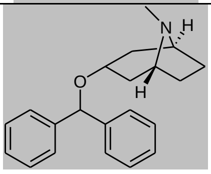	**1)** Prevalence and Correlates of Anti-Parkinson Drug Use in a Nationally Representative Sample, Dahodwala, N; Willis, AW; Li, PX; Doshi, JA; MOVEMENT DISORDERS CLINICAL PRACTICE, 4, 3, 335-341, DOI: 10.1002/mdc3.12422, MAY-JUN 2017, **2)** Current approaches to the treatment of Parkinson's Disease, Ellis, JM; Fell, MJ, BIOORGANIC & MEDICINAL CHEMISTRY LETTERS, 27, 18, 4247-4255, DOI: 10.1016/j.bmcl.2017.07.075, Published: SEP 15 2017, **3)** Advances in non-dopaminergic treatments for Parkinson's disease. Stayte, S; Vissel, B; FRONTIERS IN NEUROSCIENCE, 8, Article Number: 113, DOI: 10.3389/fnins.2014.00113, MAY 22 2014, **Document Type: Review**
5-Benzyloxyisatin derivatives	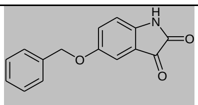 8	Inhibition of monoamine oxidase by benzoxathiolone analogues, Mostert, S; Petzer, A; Petzer, JP; BIOORGANIC & MEDICINAL CHEMISTRY LETTERS, 26, 4, 1200-1204, DOI: 10.1016/j.bmcl.2016.01.034, FEB 15 2016
5-Benzyloxyphthalimide derivatives	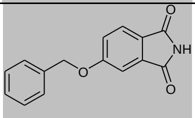 9	Inhibition of monoamine oxidase by benzoxathiolone analogues, Mostert, S; Petzer, A; Petzer, JP; BIOORGANIC & MEDICINAL CHEMISTRY LETTERS, 26, 4, 1200-1204, DOI: 10.1016/j.bmcl.2016.01.034, FEB 15 2016
8-Benzyl-tetrahydropyrazino, purinedione derivatives (various R)	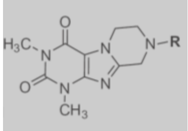	8-Substituted 1,3-dimethyltetrahydropyrazino, purinediones: Water-soluble adenosine receptor antagonists and monoamine oxidase B inhibitors, Brunschweiger, A; Koch, P; Schlenk, M; Rafehi, M; Radjainia, H; Kuppers, P; Hinz, S; Pineda, F; Wiese, M; Hockemeyer, J et al, BIOORGANIC & MEDICINAL CHEMISTRY, 24, 21, 5462-5480, DOI: 10.1016/j.bmc.2016.09.003, NOV 1 2016
8-Benzyl-tetrahydropyrazino, purinedione derivatives (derivative n.57)	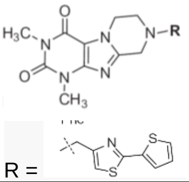	8-Substituted 1,3-dimethyltetrahydropyrazino, purinediones: Water-soluble adenosine receptor antagonists and monoamine oxidase B inhibitors, Brunschweiger, A; Koch, P; Schlenk, M; Rafehi, M; Radjainia, H; Kuppers, P; Hinz, S; Pineda, F; Wiese, M; Hockemeyer, J et al, BIOORGANIC & MEDICINAL CHEMISTRY, 24, 21, 5462-5480, DOI: 10.1016/j.bmc.2016.09.003, NOV 1 2016
3-Benzyloxy-beta-nitrostyrene derivatives	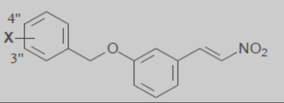	Benzyloxynitrostyrene analogues - A novel class of selective and highly potent inhibitors of monoamine oxidase B, Van der Walt, MM; Terre'Blanche, G; Petzer, JP; Petzer, A; EUROPEAN JOURNAL OF MEDICINAL CHEMISTRY, 125, 1193-1199, DOI: 10.1016/j.ejmech.2016.11.016, JAN 5 2017
4-(Benzyloxy) phenyl derivatives	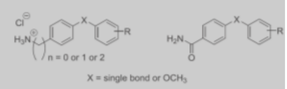	Synthesis and evaluation of biaryl derivatives for structural characterization of selective monoamine oxidase B inhibitors toward Parkinson's disease therapy, Yeon, SK; Choi, JW; Park, JH; Lee, YR; Kim, HJ; Shin, SJ; Jang, BK; Kim, S; Bahn, YS; Han, G et al, BIOORGANIC & MEDICINAL CHEMISTRY, 26, 1, 232-244, DOI: 10.1016/j.bmc.2017.11.036, JAN 1 2018
Berberine	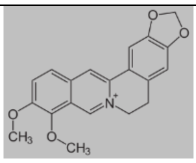	**1)** Combating neurodegenerative diseases with the plant alkaloid berberine: molecular mechanisms and therapeutic potential., Fan, Dahua; Liu, Liping; Wu, Zhengzhi; Cao, Meiqun, Current neuropharmacology, DOI:10.2174/1570159X16666180419141613, 2018-Apr-19; **2)** Preliminary studies of berberine and its semi-synthetic derivatives as a promising class of multi-target anti-parkinson agents, Ribaudo, G; Zanforlin, E; Canton, M; Bova, S; Zagotto, G; NATURAL PRODUCT RESEARCH, 32, 12, 1395-1401, DOI: 10.1080/14786419.2017.1350669, 2018, **3)** Berberine and neurodegeneration: A review of literature, Ahmed, T; Gilani, AUH; Abdollahi, M; Daglia, M; Nabavi, SF; Nabavi, SM; PHARMACOLOGICAL REPORTS, 67, 5, 970-979, DOI: 10.1016/j.pharep.2015.03.002, 2015, **Document Type:Review**
Betulin	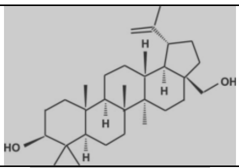	Neuroprotective Effects of Betulin in Pharmacological and Transgenic Caenorhabditis elegans Models of Parkinson's Disease, Tsai, CW; Tsai, RT; Liu, SP; Chen, CS; Tsai, MC; Chien, SH; Hung, HS; Lin, SZ; Shyu, WC; Fu, RH; CELL TRANSPLANTATION, 26, 12, 1903-1918, DOI: 10.1177/0963689717738785, DEC 2017
Bicyclogermacrene	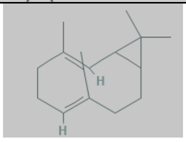	Eplingiella fruticosa leaf essential oil complexed with beta-cyclodextrin produces a superior neuroprotective and behavioral profile in a mice model of Parkinson's disease, Beserra, JIA; de Macedo, AM; Leao, AHFF; Bispo, JMM; Santos, JR; de Oliveira-Melo, AJ; Menezes, PD; Duarte, MC; Araujo, AAD; Silva, RH et al, FOOD AND CHEMICAL TOXICOLOGY, 124, 17-29, DOI: 10.1016/j.fct.2018.11.056, FEB 2019
BIIB054		Therapeutic strategies for Parkinson disease: beyond dopaminergic drugs.Charvin, D; Medori, R; Hauser, RA; Rascol, O. NATURE REVIEWS DRUG DISCOVERY, 17, 11, 804-822, DOI: 10.1038/nrd.2018.136, NOV 2018
Bikaverin	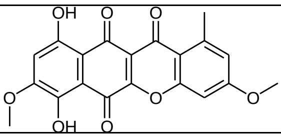	Neuroprotective Effects of Bikaverin on H2O2-Induced Oxidative Stress Mediated Neuronal Damage in SH-SY5Y Cell Line, Nirmaladevi, D; Venkataramana, M; Chandranayaka, S; Ramesha, A; Jameel, NM; Srinivas, C; CELLULAR AND MOLECULAR NEUROBIOLOGY, 34, 7, 973-985, DOI: 10.1007/s10571-014-0073-6, OCT 2014

BIOD303	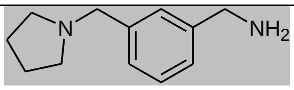	Development of Multifunctional Molecules as Potential Therapeutic Candidates for Alzheimer's Disease, Parkinson's Disease, and Amyotrophic Lateral Sclerosis in the Last Decade. Savelieff, MG; Nam, G; Kang, J; Lee, HJ; Lee, M; Lim, MH. CHEMICAL REVIEWS, 119, 2, 1221-1322, DOI: 10.1021/acs.chemrev.8b00138, JAN 23 2019, **Document Type: Review**
Biperiden	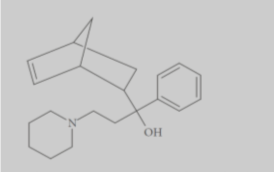	Anti-Parkinson Drug Biperiden Inhibits Enzyme Acetylcholinesterase, Kostelnik, A; Cegan, A; Pohanka, M; BIOMED RESEARCH INTERNATIONAL, Article Number: 2532764, DOI: 10.1155/2017/2532764, 2017
Biphenyl-4-yl derivatives	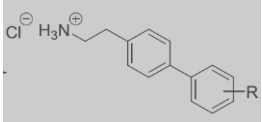	Synthesis and evaluation of biaryl derivatives for structural characterization of selective monoamine oxidase B inhibitors toward Parkinson's disease therapy, Yeon, SK; Choi, JW; Park, JH; Lee, YR; Kim, HJ; Shin, SJ; Jang, BK; Kim, S; Bahn, YS; Han, G et al, BIOORGANIC & MEDICINAL CHEMISTRY, 26, 1, 232-244, DOI: 10.1016/j.bmc.2017.11.036, JAN 1 2018
(-)-N6-(2-(4-(Biphenyl-4-yl)piperazin-1-yl)-ethyl)-N6-propyl-4,5,6,7-tetrahydrobenzo, thiazole-2,6-diamine derivatives	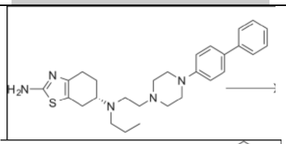 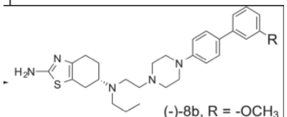 (-)-8b, R = $-OCH_3$ (-)-9b, R = -OH	Structural Modifications of Neuroprotective Anti-Parkinsonian -N6--N6-propyl-4,5,6,7-tetrahydrobenzo, thiazole-2,6-diamine : An Effort toward the Improvement of in Vivo Efficacy of the Parent Molecule, Modi, G; Antonio, T; Reith, M; Dutta, A; JOURNAL OF MEDICINAL CHEMISTRY, 57, 4, 1557-1572, DOI: 10.1021/jm401883v, FEB 27 2014
2.2'-Bipyridyl (2,2'-bipyridine)	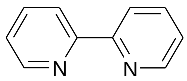	Iron Chelators and Antioxidants Regenerate Neuritic Tree and Nigrostriatal Fibers of MPP plus /MPTP-Lesioned Dopaminergic Neurons, Aguirre, P; Mena, NP; Carrasco, CM; Munoz, Y; Perez-Henriquez, P; Morales, RA; Cassels, BK; Mendez-Galvez, C; Garcia-Beltran, O; Gonzalez-Billault, C et al, PLOS ONE,10, 12, Article Number: e0144848, DOI: 10.1371/journal.pone.0144848, DEC 14 2015
Boswellic acids	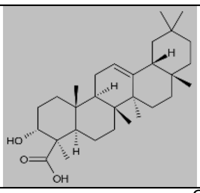	Anti-inflammatory and neuroprotective activity of boswellic acids in rotenone parkinsonian rats, Ameen, AM; Elkazaz, AY; Mohammad, HMF; Barakat, BM; CANADIAN JOURNAL OF PHYSIOLOGY AND PHARMACOLOGY, 95, 7, 819-829, DOI: 10.1139/cjpp-2016-0158, JUL 2017
4-((5-Bromo-3-chloro-2-hydroxybenzyl) amino)-2-hydroxybenzoic acid (LX007, ZL006)	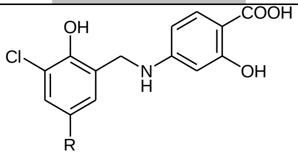	**1)** The Anti-inflammatory Effects of 4--2-hydroxybenzoic Acid in Lipopolysaccharide-Activated Primary Microglial Cells, Cao, X; Jin, YXZ; Zhang, H; Yu, LJ; Bao, XY; Li, F; Xu, Y; INFLAMMATION, 41, 2, 530-540, DOI: 10.1007/s10753-017-0709-z, MAR 2018, **2)** Small-molecule inhibitors at the PSD-95/nNOS interface attenuate MPP+-induced neuronal injury through Sirt3 mediated inhibition of mitochondrial dysfunction, Hu, W; Guan, LS; Dang, XB; Ren, PY; Zhang, YL, NEUROCHEMISTRY INTERNATIONAL,79, 57-64, DOI: 10.1016/j.neuint.2014.10.005, DEC 2014
Bromocriptine	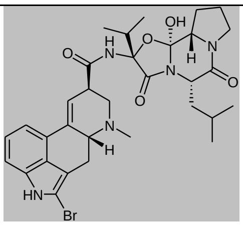	**1)** Novel Trajectories of Bromocriptine Antidiabetic Action: Leptin-IL-6/JAK2/p-STAT3/SOCS3, p-IR/p-AKT/GLUT4, PPAR-gamma/Adiponectin, Nrf2/PARP-1, and GLP-1, Reda, E; Hassaneen, S; El-Abhar, HS; FRONTIERS IN PHARMACOLOGY, 9, Article Number: 771, DOI: 10.3389/fphar.2018.00771, JUL 18 2018, **2)** Pharmacological characterization of nicotine-induced tremor: Responses to anti-tremor and anti-epileptic agents, Kunisawa, N; Shimizu, S; Kato, M; Iha, HA; Iwai, C; Hashimura, M; Ogawa, M; Kawaji, S; Kawakita, K; Abe, K et al, JOURNAL OF PHARMACOLOGICAL SCIENCES, 137, 2, 162-169, DOI: 10.1016/j.jphs.2018.05.007, JUN 2018, **3) et cetera**
Buspirone	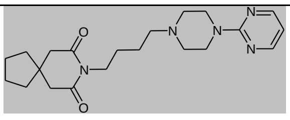	**1)** Effect of Buspirone, Fluoxetine and 8-OH-DPAT on Striatal Expression of Bax, Caspase-3 and Bcl-2 Proteins in 6-Hydroxydopamine-Induced Hemi-Parkinsonian Rats, Sharifi, H; Nayebi, AM; Farajnia, S; Haddadi, R; ADVANCED PHARMACEUTICAL BULLETIN, 5, 4, 491-495, NOV 2015, **2)** Pharmacological targeting of dopamine D3 receptors: Possible clinical applications of selective drugs, Pich, EM; Collo, G; EUROPEAN NEUROPSYCHOPHARMACOLOGY, 25, 9, 1437-1447 Special : SI, DOI: 10.1016/j.euroneuro.2015.07.012, SEP 2015
dl-3-n-Butylphthalide	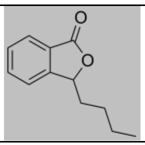	DL-3-n-butylphthalide reduces microglial activation in lipopolysaccharide-induced Parkinson's disease model mice, Chen, YH; Jiang, MJ; Li, L; Ye, M; Yu, ML; Zhang, LN; Ge, BB; Xu, WF; Wei, DX; MOLECULAR MEDICINE REPORTS, 17, 3, 3884-3890, DOI: 10.3892/mmr.2017.8332, MAR 2018

n-Butylidenephthalide	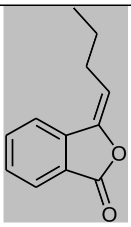	**1)** Adipose-derived Stem Cells Stimulated with n-Butylidenephthalide Exhibit Therapeutic Effects in a Mouse Model of Parkinson's Disease, Chi, K; Fu, RH; Huang, YC; Chen, SY; Hsu, CJ; Lin, SZ; Tu, CT; Chang, LH; Wu, PA; Liu, SP; CELL TRANSPLANTATION, 27, 3, 456-470, DOI: 10.1177/0963689718757408, MAR 2018**, 2)** Protective effects of DL-3-n-butylphthalide in the lipopolysaccharide-induced mouse model of Parkinson's disease, Jiang, MJ; Chen, YH; Li, L; Xu, L; Liu, H; Qu, XL; Xu, JJ; Ge, BB; Qu, HD; MOLECULAR MEDICINE REPORTS, 16, 5, 6184-6189, DOI: 10.3892/mmr.2017.7352, NOV 2017, **3)** n-Butylidenephthalide Protects against Dopaminergic Neuron Degeneration and alpha-Synuclein Accumulation in Caenorhabditis elegans Models of Parkinson's Disease, Fu, RH; Harn, HJ; Liu, SP; Chen, CS; Chang, WL; Chen, YM; Huang, JE; Li, RJ; Tsai, SY; Hung, HS et al, PLOS ONE, 9, 1, Article Number: e85305, DOI: 10.1371/journal.pone.0085305, JAN 8 2014
C-3 (α carboxyfullerene)	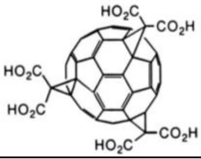	Carboxyfullerene Neuroprotection Postinjury in Parkinsonian Nonhuman Primates, Dugan, LL; Tian, LL; Quick, KL; Hardt, JI; Karimi, M; Brown, C; Loftin, S; Flores, H; Moerlein, SM; Polich, J et al, ANNALS OF NEUROLOGY, 76, 3, 393-402, DOI: 10.1002/ana.24220, SEP 2014
C-8-6-I	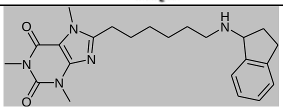	Novel Dimer Compounds That Bind alpha-Synuclein Can Rescue Cell Growth in a Yeast Model Overexpressing alpha-Synuclein. A Possible Prevention Strategy for Parkinson's Disease, Kakish, J; Allen, KJH; Harkness, TA; Krol, ES; Lee, JS; ACS CHEMICAL NEUROSCIENCE, 7, 12, 1671-1680, DOI: 10.1021/acschemneuro.6b00209, DEC 2016
C-8-6-N	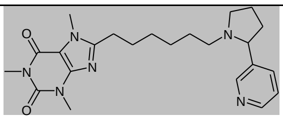	Novel Dimer Compounds That Bind alpha-Synuclein Can Rescue Cell Growth in a Yeast Model Overexpressing alpha-Synuclein. A Possible Prevention Strategy for Parkinson's Disease, Kakish, J; Allen, KJH; Harkness, TA; Krol, ES; Lee, JS; ACS CHEMICAL NEUROSCIENCE, 7, 12, 1671-1680, DOI: 10.1021/acschemneuro.6b00209, DEC 2016
C Domain of Tetanus Toxin		Effectiveness of Fragment C Domain of Tetanus Toxin and Pramipexole in an Animal Model of Parkinson's Disease, Patricio, F; Parra, I; Martinez, I; Perez-Severiano, F; Montes, S; Aguilera, J; Limon, ID; Tizabi, Y; Mendieta, L; NEUROTOXICITY RESEARCH, 35, 3, 699-710, DOI: 10.1007/s12640-018-9990-3, APR 2019
Cabergoline	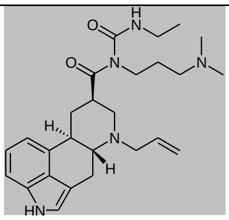	**1)** Prevalence and Correlates of Anti-Parkinson Drug Use in a Nationally Representative Sample, Dahodwala, N; Willis, AW; Li, PX; Doshi, JA, MOVEMENT DISORDERS CLINICAL PRACTICE,4, 3, 335-341, DOI: 10.1002/mdc3.12422, MAY-JUN 2017 **2)** Advances in non-dopaminergic treatments for Parkinson's disease. Stayte, S; Vissel, B; FRONTIERS IN NEUROSCIENCE, 8, Article Number: 113, DOI: 10.3389/fnins.2014.00113, MAY 22 2014, **Document Type: Review, 3)** Duration of drug action of dopamine D2 agonists in mice with 6-hydroxydopamine-induced lesions, Tsuchioka, A; Oana, F; Suzuki, T; Yamauchi, Y; Ijiro, T; Kaidoh, K; Hiratochi, M; NEUROREPORT, 26, 18, 1126-1132, DOI: 10.1097/WNR.0000000000000484, DEC 16 2015
Caffeic acid amide analogues	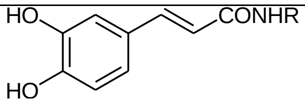	**1)** N-Propargyl Caffeamide Ameliorates Dopaminergic Neuronal Loss and Motor Dysfunctions in MPTP Mouse Model of Parkinson's Disease and in MPP+-Induced Neurons via Promoting the Conversion of proNGF to NGF, Luo, D; Zhao, J; Cheng, YY; Lee, SMY; Rong, JH, MOLECULAR NEUROBIOLOGY,55, 3, 2258-2267, DOI: 10.1007/s12035-017-0486-6, MAR 2018**, 2)** Derivatives of caffeic acid, a natural antioxidant, as the basis for the discovery of novel nonpeptidic neurotrophic agents, Moosavi, F; Hosseini, R; Rajaian, H; Silva, T; Silva, DME; Saso, L; Edraki, N; Miri, R; Borges, F; Firuzi, O; BIOORGANIC & MEDICINAL CHEMISTRY, 25, 12, 3233-+, DOI: 10.1016/j.bmc.2017.04.026, JUN 15 2017, **3)** Neuroprotection comparison of chlorogenic acid and its metabolites against mechanistically distinct cell death-inducing agents in cultured cerebellar granule neurons, Taram, F; Winter, AN; Linseman, DA; BRAIN RESEARCH, 1648, 69-80 Part: A, DOI: 10.1016/j.brainres.2016.07.028, OCT 1 2016**, 4)** Anti-fibrillation potency of caffeic acid against an antidepressant induced fibrillogenesis of human alpha-synuclein: Implications for Parkinson's disease, Fazili, NA; Naeem, A; BIOCHIMIE, 108, 178-185, DOI: 10.1016/j.biochi.2014.11.011, JAN 2015, **5) et cetera**
Caffeine	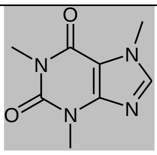	**1)** Adenosine role in brain functions: Pathophysiological influence on Parkinson's disease and other brain disorders, Soliman, AM; Fathalla, AM; Moustafa, AA; PHARMACOLOGICAL REPORTS, 70, 4, 661-667, DOI: 10.1016/j.pharep.2018.02.003, AUG 2018, **Document Type:Review, 2)** Caffeine; the Forgotten Potential for Parkinson's Disease, Negida, A; Elfil, M; Attia, A; Farahat, E; Gabr, M; Essam, A; Attia, D; Ahmed, H; CNS & NEUROLOGICAL DISORDERS-DRUG TARGETS, 16, 6, 652-657, DOI: 10.2174/1871527315666161107091149, 2017, **Document Type:Review, 3)** Caffeine as a Lead Compound for the Design of Therapeutic Agents for the Treatment of Parkinson's Disease, Petzer, JP; Petzer, A; CURRENT MEDICINAL CHEMISTRY, 22, 8, 975-988, DOI: 10.2174/0929867322666141215160015, 2015
Caffeine - Docosahexaenoic acid dimer	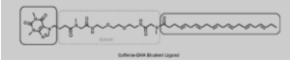	Synthesis and Characterization of a New Bivalent Ligand Combining Caffeine and Docosahexaenoic Acid, Fernandez-Duenas, V; Azuaje, J; Morato, X; Cordobilla, B; Domingo, JC; Sotelo, E; Ciruela, F; MOLECULES, 22, 3, Article Number: 366, DOI: 10.3390/molecules22030366, MAR 2017

Capreomycin	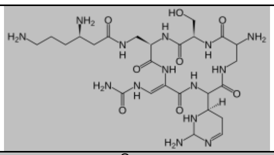	Capreomycin inhibits the initiation of amyloid fibrillation and suppresses amyloid induced cell toxicity, Siddiqi, MK; Alam, P; Chaturvedi, SK; Khan, MV; Nusrat, S; Malik, S; Khan, RH; BIOCHIMICA ET BIOPHYSICA ACTA-PROTEINS AND PROTEOMICS, 1866, 4, 549-557, DOI: 10.1016/j.bbapap.2018.02.005, APR 2018
Capsaicin	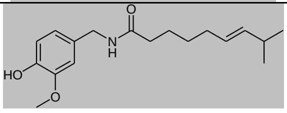	Capsaicin prevents degeneration of dopamine neurons by inhibiting glial activation and oxidative stress in the MPTP model of Parkinson's disease, Chung, YC; Baek, JY; Kim, SR; Ko, HW; Bok, E; Shin, WH; Won, SY; Jin, BK; EXPERIMENTAL AND MOLECULAR MEDICINE, 49, Article Number: e298, DOI: 10.1038/emm.2016.159, MAR 2017
Carbazole-derived compounds	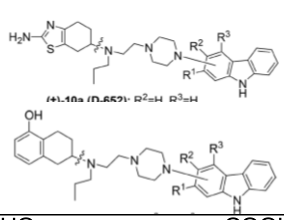	Design, Synthesis, and Pharmacological Characterization of Carbazole Based Dopamine Agonists as Potential Symptomatic and Neuroprotective Therapeutic Agents for Parkinson's Disease, Elmabruk, A; Das, B; Yedlapudi, D; Xu, LP; Antonio, T; Reith, MEA; Dutta, AK; ACS CHEMICAL NEUROSCIENCE, 10, 1, 396-411, DOI: 10.1021/acschemneuro.8b00291, JAN 2019
Carbidopa	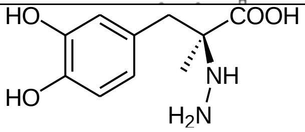	**1)** Interference of carbidopa and other catechols with reactions catalyzed by peroxidases, Gasowska-Bajger, B; Nishigaya, Y; Hirsz-Wiktorzak, K; Rybczynska, A; Yamazaki, T; Wojtasek, H; BIOCHIMICA ET BIOPHYSICA ACTA-GENERAL SUBJECTS, 1862, 7, 1626-1634, DOI: 10.1016/j.bbagen.2018.04.007, JUL 2018, **2)** Liquid levodopa-carbidopa in advanced Parkinson's disease with motor complications, Yang, HJ; Ehm, G; Kim, YE; Yun, JY; Lee, WW; Kim, A; Kim, HJ; Jeon, B; JOURNAL OF THE NEUROLOGICAL SCIENCES, 377, 6-11, DOI: 10.1016/j.jns.2017.03.039, JUN 15 2017
Cariprazine	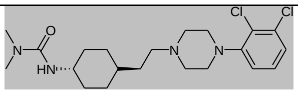	Dual Ligands Targeting Dopamine D-2 and Serotonin 5-HT1A Receptors as New Antipsychotical or Anti-Parkinsonian Agents, Ye, N; Song, ZL; Zhang, A; CURRENT MEDICINAL CHEMISTRY, 21, 4, 437-457, FEB 2014, **Document Type:Review**
Carnosic acid	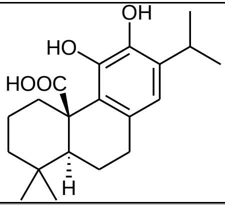	**1)** Neuroprotection Comparison of Rosmarinic Acid and Carnosic Acid in Primary Cultures of Cerebellar Granule Neurons, Taram, F; Ignowski, E; Duval, N; Linseman, DA; MOLECULES, 23, 11, Article Number: 2956, DOI: 10.3390/molecules23112956, NOV 2018, **2)** Protective effect of carnosic acid against paraquat-induced redox impairment and mitochondrial dysfunction in SH-SY5Y cells: Role for PI3K/Akt/Nrf2 pathway, de Oliveira, MR; Ferreira, GC; Schuck, PF; TOXICOLOGY IN VITRO, 32, 41-54, DOI: 10.1016/j.tiv.2015.12.005, APR 2016
Carnosine	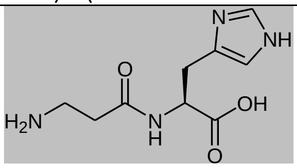	**1)** Neuroprotective effect of the carnosine - alpha-lipoic acid nanomicellar complex in a model of early-stage Parkinson's disease, Kulikova, OI; Berezhnoy, DS; Stvolinsky, SL; Lopachev, AV; Orlova, VS; Fedorova, TN; REGULATORY TOXICOLOGY AND PHARMACOLOGY, 95, 254-259, DOI: 10.1016/j.yrtph.2018.03.025, JUN 2018, **2)** Neuroprotective effect of carnosine against salsolinol-induced Parkinson's disease, Zhao, J; Shi, L; Zhang, LR; EXPERIMENTAL AND THERAPEUTIC MEDICINE, 14, 1, 664-670 Part: B, DOI: 10.3892/etm.2017.4571, JUL 2017
β-Caryophyllene	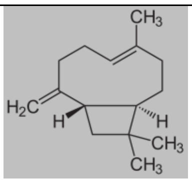	**1)** Eplingiella fruticosa leaf essential oil complexed with beta-cyclodextrin produces a superior neuroprotective and behavioral profile in a mice model of Parkinson's disease, Beserra, JIA; de Macedo, AM; Leao, AHFF; Bispo, JMM; Santos, JR; de Oliveira-Melo, AJ; Menezes, PD; Duarte, MC; Araujo, AAD; Silva, RH et al, FOOD AND CHEMICAL TOXICOLOGY, 124, 17-29, DOI: 10.1016/j.fct.2018.11.056, FEB 2019, **2)** beta-Caryophyllene ameliorates MPP plus induced cytotoxicity, Wang, GF; Ma, WB; Du, JW; BIOMEDICINE & PHARMACOTHERAPY, 103, 1086-1091, DOI: 10.1016/j.biopha.2018.03.168, JUL 2018, **3)** Neuroprotective Effects of beta-Caryophyllene against Dopaminergic Neuron Injury in a Murine Model of Parkinson's Disease Induced by MPTP, Viveros-Paredes, JM; Gonzalez-Castaneda, RE; Gertsch, J; Chaparro-Huerta, V; Lopez-Roa, RI; Vazquez-Valls, E; Beas-Zarate, C; Camins-Espuny, A; Flores-Soto, ME; PHARMACEUTICALS, 10, 3, Article Number: UNSP 60, DOI: 10.3390/ph10030060, SEP 2017
Carvacrol	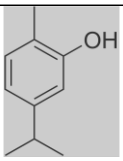	**1)** Carvacrol prevents impairments in motor and neurochemical parameters in a model of progressive parkinsonism induced by reserpine, Lins, LCRF; Souza, MF; Bispo, JMM; Gois, AM; Melo, TCS; Andrade, RAS; Quintans, LJ; Ribeiro, AM; Silva, RH; Santos, JR et al, BRAIN RESEARCH BULLETIN, 139, 9-15, DOI: 10.1016/j.brainresbull.2018.01.017, MAY 2018, **2)** Application of Monoterpenoids and their Derivatives for Treatment of Neurodegenerative Disorders, Volcho, KP; Laev, SS; Ashraf, GM; Aliev, G; Salakhutdinov, NF, CURRENT MEDICINAL CHEMISTRY,25, 39, 5327-5346, DOI: 10.2174/0929867324666170112101837, 2018, **Document Type:Review**
Catalpol	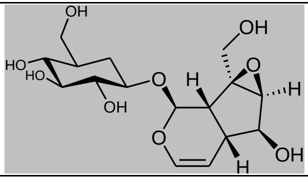	**1)** Application of Monoterpenoids and their Derivatives for Treatment of Neurodegenerative Disorders, Volcho, KP; Laev, SS; Ashraf, GM; Aliev, G; Salakhutdinov, NF, CURRENT MEDICINAL CHEMISTRY,25, 39, 5327-5346, DOI: 10.2174/0929867324666170112101837, 2018, **Document Type:Review**, **2)** Recent advances in discovery and development of natural products as source for anti-Parkinson's disease lead compounds, Zhang, HJ; Bai, L; He, J; Zhong, L; Duan, XM; Ouyang, L; Zhu, YX; Wang, T; Shi, JY; Zhang, YW, EUROPEAN

		JOURNAL OF MEDICINAL CHEMISTRY,141, 257-272, DOI: 10.1016/j.ejmech.2017.09.068, DEC 1 2017, **Document Type:Review3)** Catalpol: A Potential Therapeutic for Neurodegenerative Diseases, Jiang, B; Shen, RF; Bi, J; Tian, XS; Hinchliffe, T; Xia, Y; CURRENT MEDICINAL CHEMISTRY, 22, 10, 1278-1291, DOI: 10.2174/0929867322666150114151720, 2015
Catechin	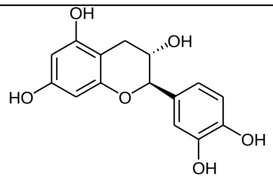	**1)** Iron Chelating Strategies in Systemic Metal Overload, Neurodegeneration and Cancer. Gumienna-Kontecka, E; Pyrkosz-Bulska, M; Szebesczyk, A; Ostrowska, M. CURRENT MEDICINAL CHEMISTRY, 21, 33, 3741-3767, DOI: 10.2174/0929867321666140706143402, 2014. **Document Type:Review**, **2)** Flavonoids and its Neuroprotective Effects on Brain Ischemia and Neurodegenerative Diseases, Putteeraj, M; Lim, WL; Teoh, SL; Yahaya, MF, CURRENT DRUG TARGETS,19, 14, 1710-1720, DOI: 10.2174/1389450119666180326125252, 2018, **Document Type:Review, 3) et cetera**
CBG cannabinoid	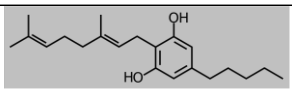	**1)** The role of cannabinoids and leptin in neurological diseases, Agar, E; ACTA NEUROLOGICA SCANDINAVICA, 132, 6, 371-380, DOI: 10.1111/ane.12411, DEC 2015, **Document Type:Review, 2)** Promising cannabinoid-based therapies for Parkinson's disease: motor symptoms to neuroprotection, More, SV; Choi, DK; MOLECULAR NEURODEGENERATION, 10, Article Number: 17, DOI: 10.1186/s13024-015-0012-0, APR 8 2015, **Document Type:Review, 3) et cetera**
Ceftriaxone	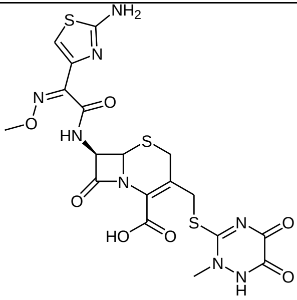	**1)** Repurposing of the beta-Lactam Antibiotic, Ceftriaxone for Neurological Disorders: A Review, Yimer, EM; Hishe, HZ; Tuem, KB; FRONTIERS IN NEUROSCIENCE, 13, Article Number: 236, DOI: 10.3389/fnins.2019.00236, MAR 26 2019, **Document Type:Review**, **2)** Development of Multifunctional Molecules as Potential Therapeutic Candidates for Alzheimer's Disease, Parkinson's Disease, and Amyotrophic Lateral Sclerosis in the Last Decade. Savelieff, MG; Nam, G; Kang, J; Lee, HJ; Lee, M; Lim, MH. CHEMICAL REVIEWS, 119, 2, 1221-1322, DOI: 10.1021/acs.chemrev.8b00138, JAN 23 2019, **Document Type:Review**, **3)** Novel tactics for neuroprotection in Parkinson's disease: Role of antibiotics, polyphenols and neuropeptides, Reglodi, D; Renaud, J; Tamas, A; Tizabi, Y; Socias, SB; Del-Bel, E; Raisman-Vozari, R; PROGRESS IN NEUROBIOLOGY, 155, 120-148 Special : SI, DOI: 10.1016/j.pneurobio.2015.10.004, AUG 2017, **Document Type:Review**, **4)** Ceftriaxone Blocks the Polymerization of alpha-Synuclein and Exerts Neuroprotective Effects in Vitro, Ruzza, P; Siligardi, G; Hussain, R; Marchiani, A; Islami, M; Bubacco, L; Delogu, G; Fabbri, D; Dettori, MA; Sechi, M et al, ACS CHEMICAL NEUROSCIENCE, 5, 1, 30-38, DOI: 10.1021/cn400149k, JAN 2014
Celastrol	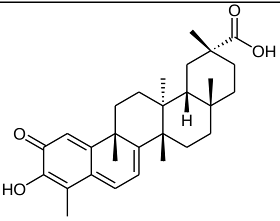	**1)** Celastrol and Its Role in Controlling Chronic Diseases, Venkatesha, SH; Moudgil, KD; ANTI-INFLAMMATORY NUTRACEUTICALS AND CHRONIC DISEASES, Edited Gupta, SC; Prasad, S; Aggarwal, BB, Book Series: Advances in Experimental Medicine and Biology, 928, 267-289, DOI: 10.1007/978-3-319-41334-1_12, 2016**, 2)** Celastrol from 'Thunder God Vine' Protects SH-SY5Y Cells Through the Preservation of Mitochondrial Function and Inhibition of p38 MAPK in a Rotenone Model of Parkinson's Disease, Choi, BS; Kim, H; Lee, HJ; Sapkota, K; Park, SE; Kim, S; Kim, SJ; NEUROCHEMICAL RESEARCH, 39, 1, 84-96, DOI: 10.1007/s11064-013-1193-y, JAN 2014
Celecoxib	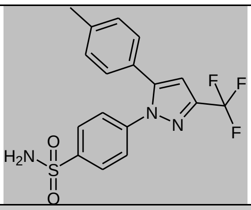	**1)** Celecoxib, indomethacin and ibuprofen prevent 6-hydroxydopamine-induced PC12 cell death through the inhibition of NF kappa B and SAPK/JNK pathways, Ramazani, E; Tayarani-Najaran, Z; Fereidoni, M; IRANIAN JOURNAL OF BASIC MEDICAL SCIENCES, 22, 5, 477-484, 2019, **2)** NSAIDs ameliorate cognitive and motor impairment in a model of parkinsonism induced by chlorpromazine, Naeem, S; Ikram, R; Khan, SS; Rao, SS; PAKISTAN JOURNAL OF PHARMACEUTICAL SCIENCES, 30, 3, 801-808, MAY 2017
Cembranoids	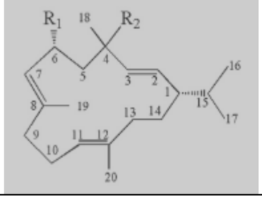	Chemical structures, biosynthesis, bioactivities, biocatalysis and semisynthesis of tobacco cembranoids: An overview, Yan, N; Du, YM; Liu, XM; Zhang, HB; Liu, YH; Zhang, P; Gong, DP; Zhang, ZF; INDUSTRIAL CROPS AND PRODUCTS, 83, 66-80, DOI: 10.1016/j.indcrop.2015.12.031, MAY 2016
CEP-1347	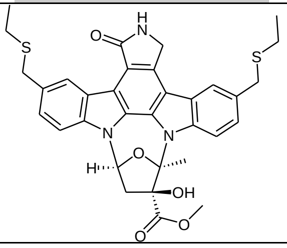	**1)** Marine-derived protein kinase inhibitors for neuroinflammatory diseases, Ning, C; Wang, HMD; Gao, R; Chang, YC; Hu, FQ; Meng, XJ; Huang, SY, BIOMEDICAL ENGINEERING ONLINE,17, Article Number: 46, DOI: 10.1186/s12938-018-0477-5, APR 24 2018, **Document Type:Review**, **2)** Repositioning CEP-1347, a chemical agent originally developed for the treatment of Parkinson's disease, as an anti-cancer stem cell drug, Okada, M; Takeda, H; Sakaki, H; Kuramoto, K; Suzuki, S; Sanomachi, T; Togashi, K; Seino, S; Kitanaka, C; ONCOTARGET, 8, 55, 94872-94882, DOI: 10.18632/oncotarget.22033, NOV 7 2017

Celocoxib	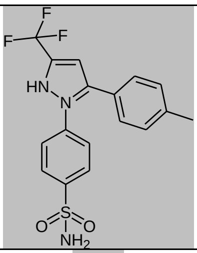	Advances in non-dopaminergic treatments for Parkinson's disease. Stayte, S; Vissel, B; FRONTIERS IN NEUROSCIENCE, 8, Article Number: 113, DOI: 10.3389/fnins.2014.00113, MAY 22 2014, **Document Type: Review**
Cerium oxide nanoparticles	Ce_2O	The possible role of cerium oxide nanoparticles in prevention of neurobehavioral and neurochemical changes in 6-hydroxydopamine-induced parkinsonian disease, Hegazy, MA; Maklad, HM; Abd Elmonsif, DA; Elnozhy, FY; Alqubiea, MA; Alenezi, FA; Al Abbas, OM; Al Abbas, MM; ALEXANDRIA JOURNAL OF MEDICINE, 53, 4, 351-360, DOI: 10.1016/j.ajme.2016.12.006, DEC 2017
Chebulagic acid	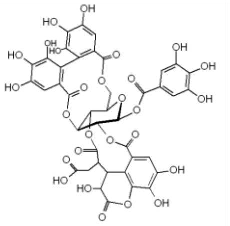	Neuroprotective Effect of Chebulagic Acid via Autophagy Induction in SH-SY5Y Cells, Kim, HJ; Kim, J; Kang, KS; Lee, KT; Yang, HO; BIOMOLECULES & THERAPEUTICS, 22, 4, 275-281, DOI: 10.4062/biomolther.2014.068, JUL 31 2014
Chlorogenic acid	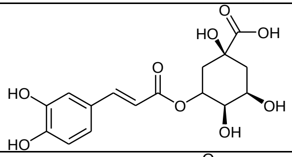	Acanthopanax senticosus Protects Structure and Function of Mesencephalic Mitochondria in A Mouse Model of Parkinson's Disease, Liu, SM; Li, XZ; Zhang, SN; Yang, ZM; Wang, KX; Lu, F; Wang, CZ; Yuan, CS; CHINESE JOURNAL OF INTEGRATIVE MEDICINE, 24, 11, 835-843, DOI: 10.1007/s11655-018-2935-5, NOV 2018
3'-O-(3-Chloropivaloyl) quercetin	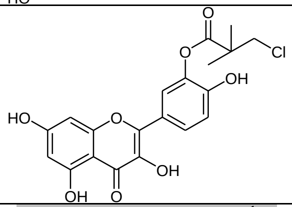	Modulation of BV-2 microglia functions by novel quercetin pivaloyl ester, Mrvova, N; Skandik, M; Kuniakova, M; Rackova, L; NEUROCHEMISTRY INTERNATIONAL, 90, 246-254, DOI: 10.1016/j.neuint.2015.09.005, NOV 2015
Chloroquine	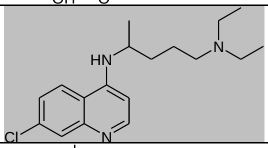	New phenylaniline derivatives as modulators of amyloid protein precursor metabolism, Gay, M; Carato, P; Coevoet, M; Renault, N; Larchanche, PE; Barczyk, A; Yous, S; Buee, L; Sergeant, N; Melnyk, P; BIOORGANIC & MEDICINAL CHEMISTRY, 26, 8, 2151-2164, DOI: 10.1016/j.bmc.2018.03.016, MAY 1 2018
Chlorpromazine	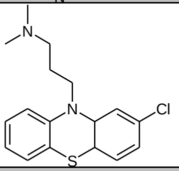	Redox-based therapeutics in neurodegenerative disease. McBean, GJ; Lopez, MG; Wallner, FK. BRITISH JOURNAL OF PHARMACOLOGY, 174, 12, 1750-1770, DOI: 10.1111/bph.13551, JUN 2017, **Document Type: Review**
Chondroitin sulfate	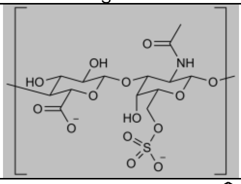	Neuroprotective effect of chondroitin sulfate on SH-SY5Y cells overexpressing wild-type or A53T mutant alpha-synuclein, Ju, CX; Gao, JJ; Hou, L; Wang, L; Zhang, F; Sun, FS; Zhang, TT; Xu, PP; Shi, ZY; Hu, F et al, MOLECULAR MEDICINE REPORTS, 16, 6, 8721-8728, DOI: 10.3892/mmr.2017.7725, DEC 2017
Chrysin	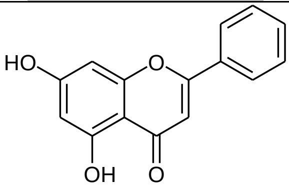	**1)** Flavonoids and its Neuroprotective Effects on Brain Ischemia and Neurodegenerative Diseases, Putteeraj, M; Lim, WL; Teoh, SL; Yahaya, MF, CURRENT DRUG TARGETS,19, 14, 1710-1720, DOI: 10.2174/1389450119666180326125252, 2018, **Document Type:Review**, **2)** Connection between Systemic Inflammation and Neuroinflammation Underlies Neuroprotective Mechanism of Several Phytochemicals in Neurodegenerative Diseases, Wang, JT; Song, YT; Chen, Z; Leng, SAX, OXIDATIVE MEDICINE AND CELLULAR LONGEVITY, Article Number: 1972714, DOI: 10.1155/2018/1972714, 2018, **Document Type: Review, 3)** Examining the neuroprotective effects of protocatechuic acid and chrysin on in vitro and in vivo models of Parkinson disease, Zhang, ZJ; Li, GH; Szeto, SSW; Chong, CM; Quan, Q; Huang, C; Cui, W; Guo, BJ; Wang, YQ; Han, YF et al, FREE RADICAL BIOLOGY AND MEDICINE, 84, 331-343, DOI: 10.1016/j.freeradbiomed.2015.02.030, JUL 2015
Cilostazol	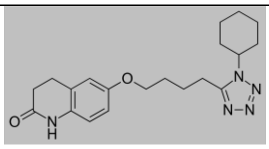	Cilostazol Mediated Nurr1 and Autophagy Enhancement: Neuroprotective Activity in Rat Rotenone PD Model, Hedya, SA; Safar, MM; Bahgat, AK; MOLECULAR NEUROBIOLOGY, 55, 9, 7579-7587, DOI: 10.1007/s12035-018-0923-1, SEP 2018

1,8-Cineole	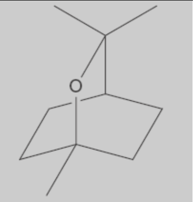	**1)** Eplingiella fruticosa leaf essential oil complexed with beta-cyclodextrin produces a superior neuroprotective and behavioral profile in a mice model of Parkinson's disease, Beserra, JIA; de Macedo, AM; Leao, AHFF; Bispo, JMM; Santos, JR; de Oliveira-Melo, AJ; Menezes, PD; Duarte, MC; Araujo, AAD; Silva, RH et al, FOOD AND CHEMICAL TOXICOLOGY, 124, 17-29, DOI: 10.1016/j.fct.2018.11.056, FEB 2019, **2)** In vitro neuroprotective potential of the monoterpenes alpha-pinene and 1,8-cineole against H2O2-induced oxidative stress in PC12 cells, Porres-Martinez, M; Gonzalez-Burgos, E; Carretero, ME; Gomez-Serranillos, MP; ZEITSCHRIFT FUR NATURFORSCHUNG SECTION C-A JOURNAL OF BIOSCIENCES, 71, 7-8, 191-199, DOI: 10.1515/znc-2014-4135, JUL 2016
trans-Cinnamaldehyde	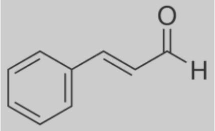	trans-Cinnamaldehyde Inhibits Microglial Activation and Improves Neuronal Survival against Neuroinflammation in BV2 Microglial Cells with Lipopolysaccharide Stimulation, Fu, Y; Yang, P; Zhao, Y; Zhang, LQ; Zhang, ZG; Dong, XW; Wu, ZP; Xu, Y; Chen, YJ; EVIDENCE-BASED COMPLEMENTARY AND ALTERNATIVE MEDICINE, Article Number: 4730878, DOI: 10.1155/2017/4730878, 2017
Citalopram	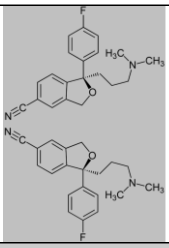	Side effect profile of 5-HT treatments for Parkinson's disease and L-DOPA-induced dyskinesia in rats, Lindenbach, D; Palumbo, N; Ostock, CY; Vilceus, N; Conti, MM; Bishop, C; BRITISH JOURNAL OF PHARMACOLOGY, 172, 1, 119-130, DOI: 10.1111/bph.12894, JAN 2015
Citronellal	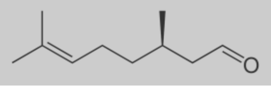	Citrus hystrix-derived 3,7-dimethyloct-6-enal and 3,7-dimethyloct-6-enyl acetate ameliorate acetylcholine deficits, Sammi, SR; Mishra, DP; Trivedi, S; Smita, SS; Nagar, A; Tandon, S; Pandey, R; RSC ADVANCES, 6, 73, 68870-68884, DOI: 10.1039/c6ra12522k, 2016
CJH1 (CLR4001)		Advances in Drug Development for Parkinson's Disease: Present Status. Reddy, DH; Misra, S; Medhi, B. PHARMACOLOGY, 93, 5-6, 260-271, DOI: 10.1159/000362419, 2014
Clioquinol	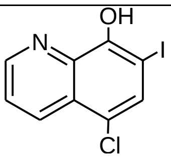	**1)** Electrochemical Detection of Interaction Between alpha-Synuclein and Clioquinol, Cheng, XR; Kerman, K; ELECTROANALYSIS, 27, 6, 1436-1442, DOI: 10.1002/elan.201500044, JUN 2015, **2)** A review on iron chelators as potential therapeutic agents for the treatment of Alzheimer's and Parkinson's diseases. Singh, YP; Pandey, A; Vishwakarma, S; Modi, G, Molecular diversity, DOI:10.1007/s11030-018-9878-4, 2018-Oct-06. **Document type: review, 3)** Genetic or pharmacological iron chelation prevents mptp-induced neurotoxicity in vivo: A novel therapy for parkinson's disease. Kaur, D., Yantiri, F., Rajagopalan, S., Kumar, J., Mo, J.Q., Boonplueang, R., Viswanath, V., Jacobs, R., Yang, L., Beal, M.F., DiMonte, D., Volitaskis, I., Ellerby, L., Cherny, R.A., Bush, A.I., Andersen, J.K. Neuron, 37, 899-909, 2003, **4)** Clioquinol rescues parkinsonism and dementia phenotypes of the tau knockout mouse. Lei, P., Ayton, S., Appukuttan, A.T., Volitakis, I., Adlard, P.A., Finkelstein, D.I., Bush, A.I. Neurobiology of disease, 81, 168-175, 2015
Clioquinol-selegiline hybrid	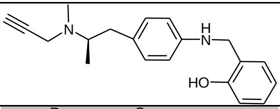	New Perspectives in Iron Chelation Therapy for the Treatment of Neurodegenerative Diseases. Nunez, MT; Chana-Cuevas, P. PHARMACEUTICALS, 11, 4, Article Number: UNSP 109, DOI: 10.3390/ph11040109, DEC 2018, **Document Type: Review**
Clovamide analogues (R_1 or R_2 = H or OCH_3, and R_3 or R_4 = H or OCH_3)	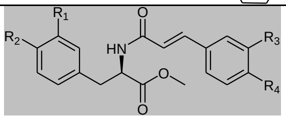	Synthesis and biological evaluation of clovamide analogues with catechol functionality as potent Parkinson's disease agents in vitro and in vivo, Feng, JH; Hu, XL; Lv, XY; Wang, BL; Lin, J; Zhang, XQ; Ye, WC; Xiong, F; Wang, H; BIOORGANIC & MEDICINAL CHEMISTRY LETTERS, 29, 2, 302-312, DOI: 10.1016/j.bmcl.2018.11.030, JAN 15 2019
Clovamide analogues (R_1 and R_2 = OH, and/or R_3 and R_4 = OH)	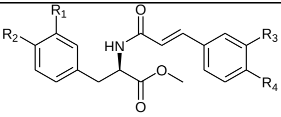	Synthesis and biological evaluation of clovamide analogues with catechol functionality as potent Parkinson's disease agents in vitro and in vivo, Feng, JH; Hu, XL; Lv, XY; Wang, BL; Lin, J; Zhang, XQ; Ye, WC; Xiong, F; Wang, H; BIOORGANIC & MEDICINAL CHEMISTRY LETTERS, 29, 2, 302-312, DOI: 10.1016/j.bmcl.2018.11.030, JAN 15 2019
Clozapine	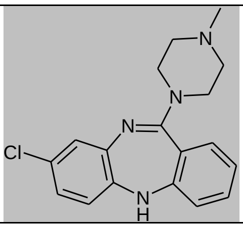	**1)** Clozapine metabolites protect dopaminergic neurons through inhibition of microglial NADPH oxidase, Jiang, LL; Wu, XF; Wang, S; Chen, SH; Zhou, H; Wilson, B; Jin, CY; Lu, RB; Xie, KQ; Wang, QS et al, JOURNAL OF NEUROINFLAMMATION, 13, Article Number: 110, DOI: 10.1186/s12974-016-0573-z, MAY 16 2016, **2)** Clozapine in Parkinsonian Rest Tremor: A Review of Outcomes, Adverse Reactions, and Possible Mechanisms of Action, Yaw, TK; Fox, SH; Lang, AE; MOVEMENT DISORDERS CLINICAL PRACTICE, 3, 2, 116-124, DOI: 10.1002/mdc3.12266, MAR-APR 2016, **Document Type:Review**

Clozapine N-oxide	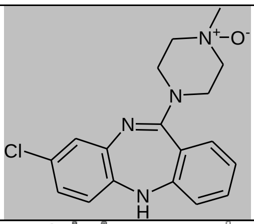	**1)** Clozapine metabolites protect dopaminergic neurons through inhibition of microglial NADPH oxidase, Jiang, LL; Wu, XF; Wang, S; Chen, SH; Zhou, H; Wilson, B; Jin, CY; Lu, RB; Xie, KQ; Wang, QS et al, JOURNAL OF NEUROINFLAMMATION, 13, Article Number: 110, DOI: 10.1186/s12974-016-0573-z, MAY 16 2016, **2)** Clozapine in Parkinsonian Rest Tremor: A Review of Outcomes, Adverse Reactions, and Possible Mechanisms of Action, Yaw, TK; Fox, SH; Lang, AE; MOVEMENT DISORDERS CLINICAL PRACTICE, 3, 2, 116-124, DOI: 10.1002/mdc3.12266, MAR-APR 2016, **Document Type:Review**
CLR01	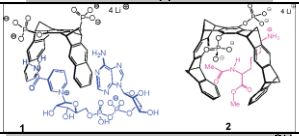	A Molecular Tweezer Ameliorates Motor Deficits in Mice Overexpressing alpha-Synuclein, Richter, F; Subramaniam, SR; Magen, I; Lee, P; Hayes, J; Attar, A; Zhu, CN; Franich, NR; Bove, N; De la Rosa, K et al, NEUROTHERAPEUTICS, 14, 4, 1107-1119, DOI: 10.1007/s13311-017-0544-9, OCT 2017
Co-101244	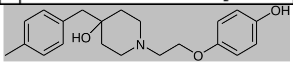	Unprecedented Therapeutic Potential with a Combination of A/NR2B Receptor Antagonists as Observed in the 6-OHDA Lesioned Rat Model of Parkinson's Disease, Michel, A; Downey, P; Nicolas, JM; Scheller, D; PLOS ONE, 9, 12, Article Number: e114086, DOI: 10.1371/journal.pone.0114086, DEC 16 2014
"Compound 1"	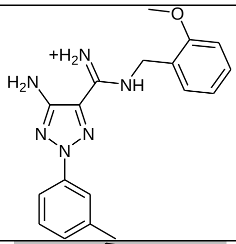	Crystal structure of the adenosine A receptor bound to an antagonist reveals a potential allosteric pocket, Sun, BF; Bachhawat, P; Chu, MLH; Wood, M; Ceska, T; Sands, ZA; Mercier, J; Lebon, F; Kobilka, TS; Kobilka, BK; PROCEEDINGS OF THE NATIONAL ACADEMY OF SCIENCES OF THE UNITED STATES OF AMERICA, 114, 8, 2066-2071, DOI: 10.1073/pnas.1621423114, FEB 21 2017
"Compound 1"	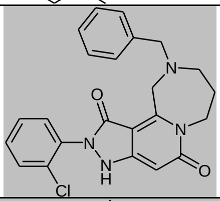	Redox-based therapeutics in neurodegenerative disease. McBean, GJ; Lopez, MG; Wallner, FK. BRITISH JOURNAL OF PHARMACOLOGY, 174, 12, 1750-1770, DOI: 10.1111/bph.13551, JUN 2017, **Document Type: Review**
"Compound 2"	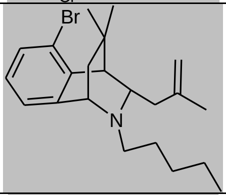	Redox-based therapeutics in neurodegenerative disease. McBean, GJ; Lopez, MG; Wallner, FK. BRITISH JOURNAL OF PHARMACOLOGY, 174, 12, 1750-1770, DOI: 10.1111/bph.13551, JUN 2017, **Document Type: Review**
"Compound 3"	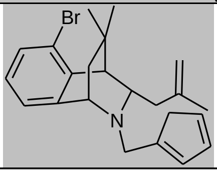	Redox-based therapeutics in neurodegenerative disease. McBean, GJ; Lopez, MG; Wallner, FK. BRITISH JOURNAL OF PHARMACOLOGY, 174, 12, 1750-1770, DOI: 10.1111/bph.13551, JUN 2017, **Document Type: Review**
"Compound 3f"	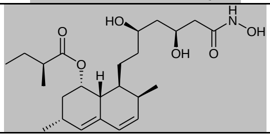	Neurocytoprotective Effects of Aliphatic Hydroxamates from Lovastatin, a Secondary Metabolite from Monascus-Fermented Red Mold Rice, in 6-Hydroxydopamine -Treated Nerve Growth Factor -Differentiated PC12 Cells, Lin, CM; Lin, YT; Lin, RD; Huang, WJ; Lee, MH; ACS CHEMICAL NEUROSCIENCE, 6, 5, 716-724, DOI: 10.1021/cn500275k, MAY 2015
"Compound (-) -8a"	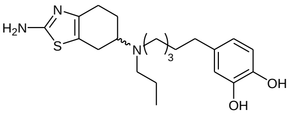	Novel multifunctional dopamine D-2/D-3 receptors agonists with potential neuroprotection and anti-alpha synuclein protein aggregation properties, Luo, D; Sharma, H; Yedlapudi, D; Antonio, T; Reith, MEA; Dutta, AK, BIOORGANIC & MEDICINAL CHEMISTRY,24, 21, 5088-5102, DOI: 10.1016/j.bmc.2016.08.021, NOV 1 2016
"Compound 8"	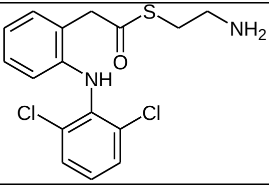	Design, synthesis and evaluation of a series of non-steroidal anti-inflammatory drug conjugates as novel neuroinflammatory inhibitors, Xu, ZX; Wu, J; Zheng, JY; Ma, HK; Zhang, HJ; Zhen, XC; Zheng, LT; Zhang, XH, INTERNATIONAL IMMUNOPHARMACOLOGY,25, 2, 528-537, DOI: 10.1016/j.intimp.2015.02.033, APR 2015
"Compound 21", derivative of 3-methyl-1-(2,4,6-trihydroxyphenyl) butan-1-one	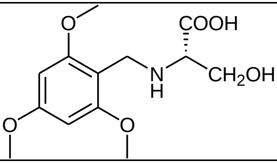	A Novel Parkinson's Disease Drug Candidate with Potent Anti-neuroinflammatory Effects through the Src Signaling Pathway, Wang, YD; Bao, XQ; Xu, S; Yu, WW; Cao, SN; Hu, JP; Li, Y; Wang, XL; Zhang, D; Yu, SS, JOURNAL OF MEDICINAL CHEMISTRY,59, 19, 9062-9079, DOI: 10.1021/acs.jmedchem.6b00976, OCT 13 2016
"Compound (-) – 21 a", derivative of N-6-(2-(4-(1H-indol-5-yl)piperazin-1-yl)ethyl)-N-6-propyl-4,5,6,7-tetrahydrobenzo[d]thiazole-		Development of a Highly Potent D-2/D-3 Agonist and a Partial Agonist from Structure-Activity Relationship Study of N-6-(2-ethyl)-N-6-propyl-4,5,6,7-tetrahydrobenzo,thiazole-2,6-diamine Analogues: Implication in the Treatment of Parkinson's Disease, Das, B; Vedachalam, S; Luo, D; Antonio, T; Reith, MEA; Dutta, AK, JOURNAL OF MEDICINAL CHEMISTRY,58, 23, 9179-9195, DOI:

2,6-diamine	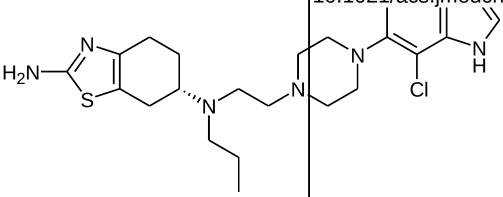	10.1021/acs.jmedchem.5b01031, DEC 10 2015
3-Coumaranone derivatives	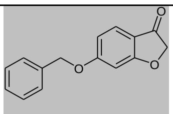 10	Inhibition of monoamine oxidase by benzoxathiolone analogues, Mostert, S; Petzer, A; Petzer, JP; BIOORGANIC & MEDICINAL CHEMISTRY LETTERS, 26, 4, 1200-1204, DOI: 10.1016/j.bmcl.2016.01.034, FEB 15 2016
Coumarin-tris hybrid CT51	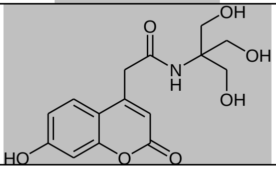	New Perspectives in Iron Chelation Therapy for the Treatment of Neurodegenerative Diseases. Nunez, MT; Chana-Cuevas, P. PHARMACEUTICALS, 11, 4, Article Number: UNSP 109, DOI: 10.3390/ph11040109, DEC 2018, **Document Type: Review**
CPP		Advances in non-dopaminergic treatments for Parkinson's disease. Stayte, S; Vissel, B ; FRONTIERS IN NEUROSCIENCE, 8, Article Number: 113, DOI: 10.3389/fnins.2014.00113, MAY 22 2014
Creatine	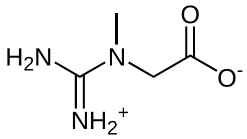	**1)** Meta-Analysis of Creatine for Neuroprotection Against Parkinson's Disease, Attia, A; Ahmed, H; Gadelkarim, M; Morsi, M; Awad, K; Elnenny, M; Ghanem, E; El-Jaafary, S; Negida, A, CNS & NEUROLOGICAL DISORDERS-DRUG TARGETS,16, 2, 169-175, DOI: 10.2174/1871527315666161104161855, 2017, **Document Type: Review**, **2)** Creatine Revealed Anticonvulsant Properties on Chemically and Electrically Induced Seizures in Mice, Shafaroodi, H; Shahbek, F; Faizi, M; Ebrahimi, F; Moezi, L, IRANIAN JOURNAL OF PHARMACEUTICAL RESEARCH,15, 4, 843-850, FAL 2016
CSC	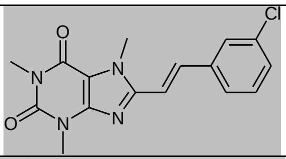	Xanthine derivatives as agents affecting non-dopaminergic neuroprotection in Parkinson`s disease., Kasabova-Angelova, A; Tzankova, D; Mitkov, J; Georgieva, M; Tzankova, V; Zlatkov, A; Kondeva-Burdina, M, Current medicinal chemistry, DOI:10.2174/0929867325666180821153316, 2018-Aug-21
Cu-II (atsm), a copper bis(thiosemicarbazonato) complex	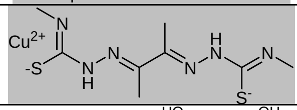	Gene dysregulation is restored in the Parkinson's disease MPTP neurotoxic mice model upon treatment of the therapeutic drug Cu-II, Cheng, L; Quek, CYJ; Hung, LW; Sharples, RA; Sherratt, NA; Barnham, KJ; Hill, AF, SCIENTIFIC REPORTS,6, Article Number: 22398, DOI: 10.1038/srep22398, MAR 1 2016
Cudraflavone B	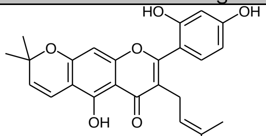	Cudarflavone B Provides Neuroprotection against Glutamate-Induced Mouse Hippocampal HT22 Cell Damage through the Nrf2 and PI3K/Akt Signaling Pathways, Lee, DS; Ko, W; Kim, DC; Kim, YC; Jeong, GS, MOLECULES,19, 8, 10818-10831, DOI: 10.3390/molecules190810818, AUG 2014
10-O-trans-p-Cumaroylcatalpol	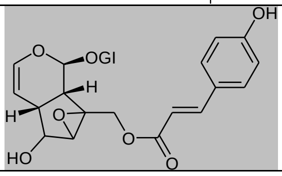	Recent advances in discovery and development of natural products as source for anti-Parkinson's disease lead compounds, Zhang, HJ; Bai, L; He, J; Zhong, L; Duan, XM; Ouyang, L; Zhu, YX; Wang, T; Shi, JY; Zhang, YW, EUROPEAN JOURNAL OF MEDICINAL CHEMISTRY,141, 257-272, DOI: 10.1016/j.ejmech.2017.09.068, DEC 1 2017, **Document Type:Review**
Curcumin	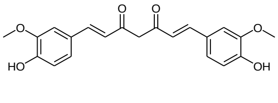	**1)** The effect of curcumin against 6-hydroxydopamine induced cell death and Akt/GSK disruption in human neuroblastoma cells, Moosavi, M; Farrokhi, MR; Tafreshi, N, PHYSIOLOGY AND PHARMACOLOGY,22, 3, 163-171, SEP 2018, **2)** Use of Curcumin, a Natural Polyphenol for Targeting Molecular Pathways in Treating Age-Related Neurodegenerative Diseases, Maiti, P; Dunbar, GL, INTERNATIONAL JOURNAL OF MOLECULAR SCIENCES,19, 6, Article Number: 1637, DOI: 10.3390/ijms19061637, JUN 2018, **Document Type:Review**, **3)** Curcumin affords neuroprotection and inhibits alpha-synuclein aggregation in lipopolysaccharide-induced Parkinson's disease model, Sharma, N; Nehru, B, INFLAMMOPHARMACOLOGY,26, 2, 349-360, DOI: 10.1007/s10787-017-0402-8, APR 2018, **4)** A review on iron chelators as potential therapeutic agents for the treatment of Alzheimer's and Parkinson's diseases. Singh, YP; Pandey, A; Vishwakarma, S; Modi, G, Molecular diversity, DOI:10.1007/s11030-018-9878-4, 2018-Oct-06. **Document type: review**, **5)** Recent advances in discovery and development of natural products as source for anti-Parkinson's disease lead compounds, Zhang, HJ; Bai, L; He, J; Zhong, L; Duan, XM; Ouyang, L; Zhu, YX; Wang, T; Shi, JY; Zhang, YW, EUROPEAN JOURNAL OF MEDICINAL CHEMISTRY,141, 257-272, DOI: 10.1016/j.ejmech.2017.09.068, DEC 1 2017, **Document Type:Review, 6) et cetera**
Curcumin pyrazole derivatives	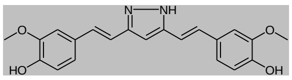	Curcumin Pyrazole and its derivative (N- Curcumin inhibit aggregation, disrupt fibrils and modulate toxicity of Wild type and Mutant alpha-Synuclein, Ahsan, N; Mishra, S; Jain, MK; Surolia, A; Gupta, S, SCIENTIFIC REPORTS,5, Article Number: 9862, DOI: 10.1038/srep09862, MAY 18 2015

	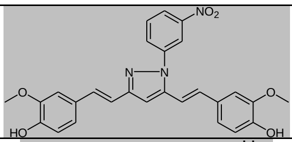	
CP-94253	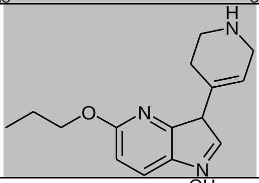	Advances in non-dopaminergic treatments for Parkinson's disease. Stayte, S; Vissel, B; FRONTIERS IN NEUROSCIENCE, 8, Article Number: 113, DOI: 10.3389/fnins.2014.00113, MAY 22 2014, **Document Type: Review**
Cyanidin	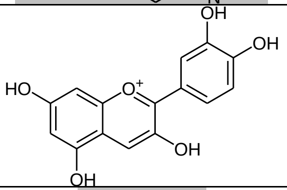	**1)** Anthocyanins Improve Hippocampus-Dependent Memory Function and Prevent Neurodegeneration via JNK/Akt/GSK3 Signaling in LPS-Treated Adult Mice, Khan, MS; Ali, T; Kim, MW; Jo, MH; Chung, JI; Kim, MO; MOLECULAR NEUROBIOLOGY, 56, 1, 671-687, DOI: 10.1007/s12035-018-1101-1, JAN 2019, **2)** Cyanidin Protects SH-SY5Y Human Neuroblastoma Cells from 1-Methyl-4-Phenylpyridinium-Induced Neurotoxicity, Chen, J; Sun, J; Jiang, JL; Zhou, J, PHARMACOLOGY,102, 3-4, 126-132, DOI: 10.1159/000489853, 2018
2-Cyclopropylimino-3-methyl-1,3-thiazoline	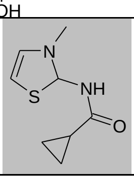	2-Cyclopropylimino-3-Methyl-1,3-Thiazoline Hydrochloride Inhibits Microglial Activation by Suppression of Nuclear Factor-Kappa B and Mitogen-Activated Protein Kinase Signaling, Kim, EA; Choi, J; Han, AR; Cho, CH; Choi, SY; Ahn, JY; Cho, SW, JOURNAL OF NEUROIMMUNE PHARMACOLOGY,9, 4, 461-467, DOI: 10.1007/s11481-014-9542-4, SEP 2014
D-Cycloserine	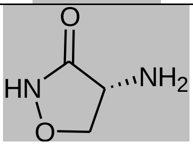	**1)** Novel tactics for neuroprotection in Parkinson's disease: Role of antibiotics, polyphenols and neuropeptides, Reglodi, D; Renaud, J; Tamas, A; Tizabi, Y; Socias, SB; Del-Bel, E; Raisman-Vozari, R; PROGRESS IN NEUROBIOLOGY, 155, 120-148 Special : SI, DOI: 10.1016/j.pneurobio.2015.10.004, AUG 2017, **Document Type:Review 2)** Development of Multifunctional Molecules as Potential Therapeutic Candidates for Alzheimer's Disease, Parkinson's Disease, and Amyotrophic Lateral Sclerosis in the Last Decade. Savelieff, MG; Nam, G; Kang, J; Lee, HJ; Lee, M; Lim, MH. CHEMICAL REVIEWS, 119, 2, 1221-1322, DOI: 10.1021/acs.chemrev.8b00138, JAN 23 2019, **Document Type:Review**
Cyclotides		Inhibition of Human Prolyl Oligopeptidase Activity by the Cyclotide Psysol 2 Isolated from Psychotria solitudinum, Hellinger, R; Koehbach, J; Puigpinos, A; Clark, RJ; Tarrago, T; Giralt, E; Gruber, CW, JOURNAL OF NATURAL PRODUCTS,78, 5, 1073-1082, DOI: 10.1021/np501061t, MAY 2015
Cytochrome P450 2D6		Cytochrome P450 2D6 and Parkinson's Disease: Polymorphism, Metabolic Role, Risk and Protection, Rasheed, MSU; Mishra, AK; Singh, MP, NEUROCHEMICAL RESEARCH,42, 12, 3353-3361, DOI: 10.1007/s11064-017-2384-8, DEC 2017, **Document Type: Review**
D123	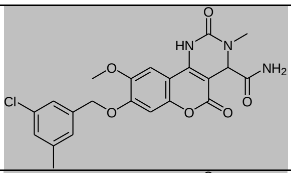	Understanding the Molecular Determinant of Reversible Human Monoamine Oxidase B Inhibitors Containing 2H-Chromen-2-One Core: Structure-Based and Ligand-Based Derived Three-Dimensional Quantitative Structure Activity Relationships Predictive Models, Mladenovic, M; Patsilinakos, A; Pirolli, A; Sabatino, M; Ragno, R, JOURNAL OF CHEMICAL INFORMATION AND MODELING,57, 4, 787-814, DOI: 10.1021/acs.jcim.6b00608, APR 2017
D124	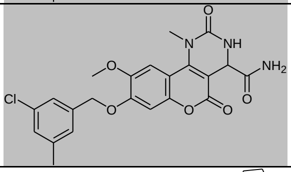	Understanding the Molecular Determinant of Reversible Human Monoamine Oxidase B Inhibitors Containing 2H-Chromen-2-One Core: Structure-Based and Ligand-Based Derived Three-Dimensional Quantitative Structure Activity Relationships Predictive Models, Mladenovic, M; Patsilinakos, A; Pirolli, A; Sabatino, M; Ragno, R, JOURNAL OF CHEMICAL INFORMATION AND MODELING,57, 4, 787-814, DOI: 10.1021/acs.jcim.6b00608, APR 2017
D-512	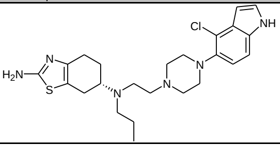	D-512, a novel dopamine D-2/3 receptor agonist, demonstrates greater anti-Parkinsonian efficacy than ropinirole in Parkinsonian rats, Lindenbach, D; Das, B; Conti, MM; Meadows, SM; Dutta, AK; Bishop, C, BRITISH JOURNAL OF PHARMACOLOGY,174, 18, 3058-3071, DOI: 10.1111/bph.13937, SEP 2017
D-607 (bipyridyl-D2R/D3R agonist hybrid)	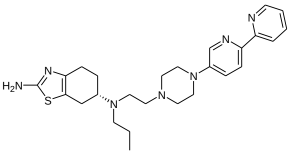	**1)** Development of Multifunctional Molecules as Potential Therapeutic Candidates for Alzheimer's Disease, Parkinson's Disease, and Amyotrophic Lateral Sclerosis in the Last Decade. Savelieff, MG; Nam, G; Kang, J; Lee, HJ; Lee, M; Lim, MH. CHEMICAL REVIEWS, 119, 2, 1221-1322, DOI: 10.1021/acs.chemrev.8b00138, JAN 23 2019, **Document Type: Review 2)** A novel iron preferring dopamine agonist chelator D-607 significantly suppresses alpha-syn- and MPTP-induced toxicities in vivo, Das, B; Rajagopalan, S; Joshi, GS; Xu, LP; Luo, D; Andersen, JK; Todi, SV; Dutta, AK, NEUROPHARMACOLOGY,123, 88-99, DOI: 10.1016/j.neuropharm.2017.05.019, SEP 1 2017, **3)** A Novel Iron Preferring Dopamine Agonist Chelator as Potential Symptomatic and Neuroprotective Therapeutic Agent for Parkinson's Disease, Das, B; Kandegedara, A; Xu, LP; Antonio, T; Stemmler, T; Reith, MEA; Dutta, AK, ACS CHEMICAL NEUROSCIENCE,8, 4, 723-730, DOI: 10.1021/acschemneuro.6b00356, APR 2017

DA-2 (8D)	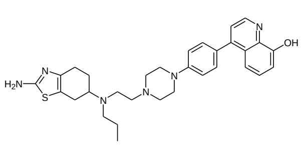	**1)** Development of Multifunctional Molecules as Potential Therapeutic Candidates for Alzheimer's Disease, Parkinson's Disease, and Amyotrophic Lateral Sclerosis in the Last Decade. Savelieff, MG; Nam, G; Kang, J; Lee, HJ; Lee, M; Lim, MH. CHEMICAL REVIEWS, 119, 2, 1221-1322, DOI: 10.1021/acs.chemrev.8b00138, JAN 23 2019, **Document Type: Review, 2)** A review on iron chelators as potential therapeutic agents for the treatment of Alzheimer's and Parkinson's diseases. Singh, YP; Pandey, A; Vishwakarma, S; Modi, G, Molecular diversity, DOI:10.1007/s11030-018-9878-4, 2018-Oct-06. **Document type: review**
DA-3	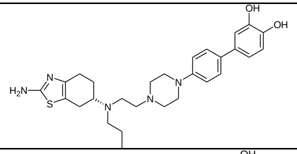	Development of Multifunctional Molecules as Potential Therapeutic Candidates for Alzheimer's Disease, Parkinson's Disease, and Amyotrophic Lateral Sclerosis in the Last Decade. Savelieff, MG; Nam, G; Kang, J; Lee, HJ; Lee, M; Lim, MH. CHEMICAL REVIEWS, 119, 2, 1221-1322, DOI: 10.1021/acs.chemrev.8b00138, JAN 23 2019, **Document Type: Review**
DA-4	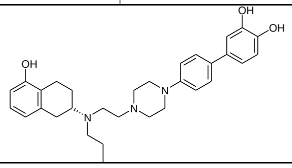	Development of Multifunctional Molecules as Potential Therapeutic Candidates for Alzheimer's Disease, Parkinson's Disease, and Amyotrophic Lateral Sclerosis in the Last Decade. Savelieff, MG; Nam, G; Kang, J; Lee, HJ; Lee, M; Lim, MH. CHEMICAL REVIEWS, 119, 2, 1221-1322, DOI: 10.1021/acs.chemrev.8b00138, JAN 23 2019, **Document Type: Review**
DA-JC1		A novel dual GLP-1 and GIP receptor agonist is neuroprotective in the MPTP mouse model of Parkinson's disease by increasing expression of BNDF, Ji, CH; Xue, GF; Cao, LJ; Feng, P; Li, DF; Li, L; Li, GL; Holscher, C, BRAIN RESEARCH,1634, 1-11, DOI: 10.1016/j.brainres.2015.09.035, MAR 1 2016
Dabigatran etexilate	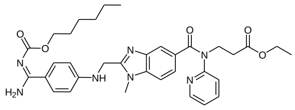	Modulatory Role of Nurr1 Activation and Thrombin Inhibition in the Neuroprotective Effects of Dabigatran Etexilate in Rotenone-Induced Parkinson's Disease in Rats, Kandil, EA; Sayed, RH; Ahmed, LA; Abd El Fattah, MA; El-Sayeh, BM, MOLECULAR NEUROBIOLOGY,55, 5, 4078-4089, DOI: 10.1007/s12035-017-0636-x, MAY 2018
Dabrafenib	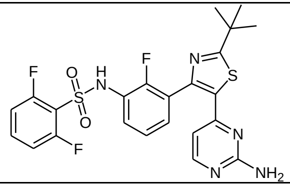	In silico drug screening by using genome-wide association study data repurposed dabrafenib, an anti-melanoma drug, for Parkinson's disease, Uenaka, T; Satake, W; Cha, PC; Hayakawa, H; Baba, K; Jiang, SY; Kobayashi, K; Kanagawa, M; Okada, Y; Mochizuki, H et al, HUMAN MOLECULAR GENETICS,27, 22, 3974-3985, DOI: 10.1093/hmg/ddy279, NOV 2018
Daidzein	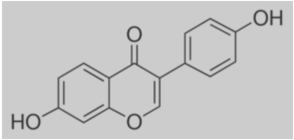	**1)** Flavonoids and its Neuroprotective Effects on Brain Ischemia and Neurodegenerative Diseases, Putteeraj, M; Lim, WL; Teoh, SL; Yahaya, MF, CURRENT DRUG TARGETS,19, 14, 1710-1720, DOI: 10.2174/1389450119666180326125252, 2018, **Document Type:Review, 2)** Recent advances in discovery and development of natural products as source for anti-Parkinson's disease lead compounds, Zhang, HJ; Bai, L; He, J; Zhong, L; Duan, XM; Ouyang, L; Zhu, YX; Wang, T; Shi, JY; Zhang, YW, EUROPEAN JOURNAL OF MEDICINAL CHEMISTRY,141, 257-272, DOI: 10.1016/j.ejmech.2017.09.068, DEC 1 2017, **Document Type:Review**
Dapsone (diaminodiphenyl sulfone)	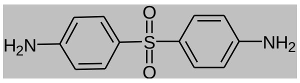	**1)** Repurposing of Copper -chelating Drugs for the Treatment of Neurodegenerative Diseases. Lanza, V; Milardi, D; Di Natale, G; Pappalardo, G. CURRENT MEDICINAL CHEMISTRY, 25, 4, 525-539, DOI: 10.2174/0929867324666170518094404, 2018, **Document Type: Review**, **2)** Diaminodiphenyl sulfone-induced parkin ameliorates age-dependent dopaminergic neuronal loss, Lee, YI; Kang, H; Ha, YW; Chang, KY; Cho, SC; Song, SO; Kim, H; Jo, A; Khang, R; Choi, JY et al, NEUROBIOLOGY OF AGING,41, 1-10, DOI: 10.1016/j.neurobiolaging.2015.11.008, MAY 2016
(S)-3,4-DCPG	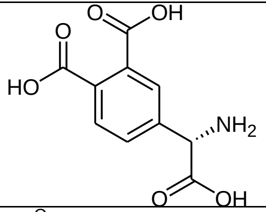	Neuroprotective effects of metabotropic glutamate receptor group II and III activators against MPP-induced cell death in human neuroblastoma SH-SY5Y cells: The impact of cell differentiation state, Jantas, D; Greda, A; Golda, S; Korostynski, M; Grygier, B; Roman, A; Pilc, A; Lason, W, NEUROPHARMACOLOGY,83, 36-53, DOI: 10.1016/j.neuropharm.2014.03.019, AUG 2014
Deferasirox	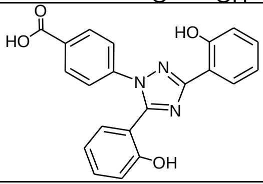	Iron Chelating Strategies in Systemic Metal Overload, Neurodegeneration and Cancer. Gumienna-Kontecka, E; Pyrkosz-Bulska, M; Szebesczyk, A; Ostrowska, M. CURRENT MEDICINAL CHEMISTRY, 21, 33, 3741-3767, DOI: 10.2174/0929867321666140706143402, 2014. **Document Type: Review**
Deferricoprogen	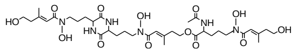	Neuroprotective effects of dimerumic acid and deferricoprogen from Monascus purpureus NTU 568-fermented rice against 6-hydroxydopamine-induced oxidative stress and apoptosis in differentiated pheochromocytoma PC-12 cells, Tseng, WT; Hsu, YW; Pan, TM, PHARMACEUTICAL BIOLOGY,54, 8, 1434-1444, DOI: 10.3109/13880209.2015.1104698, AUG 2016

Delphinidin	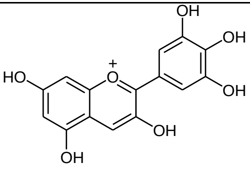 II	**1)** Anthocyanins Improve Hippocampus-Dependent Memory Function and Prevent Neurodegeneration via JNK/Akt/GSK3 Signaling in LPS-Treated Adult Mice, Khan, MS; Ali, T; Kim, MW; Jo, MH; Chung, JI; Kim, MO; MOLECULAR NEUROBIOLOGY, 56, 1, 671-687, DOI: 10.1007/s12035-018-1101-1, JAN 2019, **2)** Natural polyphenols effects on protein aggregates in Alzheimer's and Parkinson's prion-like diseases, Freyssin, A; Page, G; Fauconneau, B; Bilan, AR, NEURAL REGENERATION RESEARCH,13, 6, 955-961, DOI: 10.4103/1673-5374.233432, JUN 2018, **Document Type:Review**, **3)** Polyphenols in Parkinson's Disease: A Systematic Review of In Vivo Studies, Kujawska, M; Jodynis-Liebert, J, NUTRIENTS,10, 5, Article Number: 642, DOI: 10.3390/nu10050642, MAY 2018, **Document Type:Review**, **4)** Novel tactics for neuroprotection in Parkinson's disease: Role of antibiotics, polyphenols and neuropeptides, Reglodi, D; Renaud, J; Tamas, A; Tizabi, Y; Socias, SB; Del-Bel, E; Raisman-Vozari, R, PROGRESS IN NEUROBIOLOGY,155, 120-148 Special : SI, DOI: 10.1016/j.pneurobio.2015.10.004, AUG 2017, **Document Type:Review**, **5) et cetera**
Demethoxycurcumin	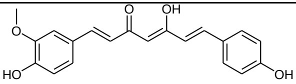	Neuroprotective effect of Demethoxycurcumin, a natural derivative of Curcumin on rotenone induced neurotoxicity in SH-SY 5Y Neuroblastoma cells, Ramkumar, M; Rajasankar, S; Gobi, VV; Dhanalakshmi, C; Manivasagam, T; Thenmozhi, AJ; Essa, MM; Kalandar, A; Chidambaram, R, BMC COMPLEMENTARY AND ALTERNATIVE MEDICINE,17, Article Number: 217, DOI: 10.1186/s12906-017-1720-5, APR 18 2017
Dendropanax morbifera active compound	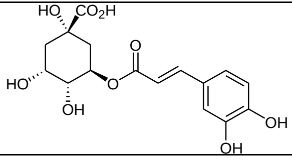	Aqueous Extract of Dendropanax morbiferus Leaves Effectively Alleviated Neuroinflammation and Behavioral Impediments in MPTP-Induced Parkinson's Mouse Model, Park, SY; Karthivashan, G; Ko, HM; Cho, DY; Kim, J; Cho, DJ; Ganesan, P; Su-Kim, I; Choi, DK, OXIDATIVE MEDICINE AND CELLULAR LONGEVITY, Article Number: 3175214, DOI: 10.1155/2018/3175214, 2018
Desferoxamine (desferrioxamine, DFO)	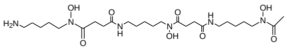	Iron Chelators and Antioxidants Regenerate Neuritic Tree and Nigrostriatal Fibers of MPP plus /MPTP-Lesioned Dopaminergic Neurons, Aguirre, P; Mena, NP; Carrasco, CM; Munoz, Y; Perez-Henriquez, P; Morales, RA; Cassels, BK; Mendez-Galvez, C; Garcia-Beltran, O; Gonzalez-Billault, C et al, PLOS ONE,10, 12, Article Number: e0144848, DOI: 10.1371/journal.pone.0144848, DEC 14 2015
N-Desmethylclozapine	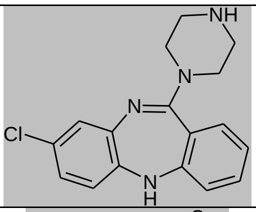	**1)** Clozapine metabolites protect dopaminergic neurons through inhibition of microglial NADPH oxidase, Jiang, LL; Wu, XF; Wang, S; Chen, SH; Zhou, H; Wilson, B; Jin, CY; Lu, RB; Xie, KQ; Wang, QS et al, JOURNAL OF NEUROINFLAMMATION, 13, Article Number: 110, DOI: 10.1186/s12974-016-0573-z, MAY 16 2016, **2)** Clozapine in Parkinsonian Rest Tremor: A Review of Outcomes, Adverse Reactions, and Possible Mechanisms of Action, Yaw, TK; Fox, SH; Lang, AE; MOVEMENT DISORDERS CLINICAL PRACTICE, 3, 2, 116-124, DOI: 10.1002/mdc3.12266, MAR-APR 2016, **Document Type:Review**
Dextromethorphan	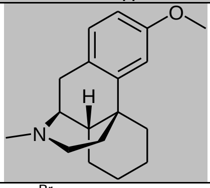	Advances in non-dopaminergic treatments for Parkinson's disease. Stayte, S; Vissel, B; FRONTIERS IN NEUROSCIENCE, 8, Article Number: 113, DOI: 10.3389/fnins.2014.00113, MAY 22 2014, **Document Type: Review**
(S)-N-(3-(3,6-Dibromo-9H-carbazol-9-yl)-2-fluoropropyl)-6-methoxypyridin-2-amine	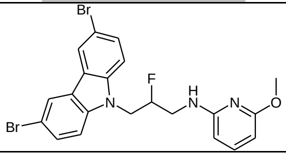	Discovery of a Neuroprotective Chemical, -N-(3--2-fluoropropyl)-6-methoxypyridin-2-amine, with Improved Druglike Properties, Naidoo, J; De Jesus-Cortes, H; Huntington, P; Estill, S; Morlock, LK; Starwalt, R; Mangano, TJ; Williams, NS; Pieper, AA; Ready, JM, JOURNAL OF MEDICINAL CHEMISTRY,57, 9, 3746-3754, DOI: 10.1021/jm401919s, MAY 8 2014
4,5-O-Dicaffeoyl-1-O-(malic acid methyl ester)-quinic acid derivatives (R_1, R_2, R_3, R_4, and R_5 = H, 5-hydroxypentanoyl, 4-methylester-maloyl, or 4-methylestersuccinyl)	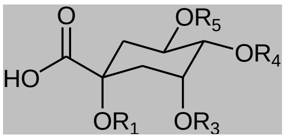	Caffeoylquinic Acid Derivatives Protect SH-SY5Y Neuroblastoma Cells from Hydrogen Peroxide-Induced Injury Through Modulating Oxidative Status, Jiang, XW; Bai, JP; Zhang, Q; Hu, XL; Tian, X; Zhu, J; Liu, J; Meng, WH; Zhao, QC, CELLULAR AND MOLECULAR NEUROBIOLOGY,37, 3, 499-509, DOI: 10.1007/s10571-016-0387-7, APR 2017
4,5-O-Dicaffeoyl-1-O-(malic acid methyl ester)-quinic acid derivatives (R_1, R_2, R_3, R_4, or R_5 = caffeoyl)	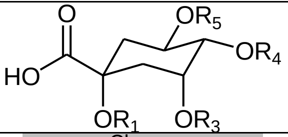	Caffeoylquinic Acid Derivatives Protect SH-SY5Y Neuroblastoma Cells from Hydrogen Peroxide-Induced Injury Through Modulating Oxidative Status, Jiang, XW; Bai, JP; Zhang, Q; Hu, XL; Tian, X; Zhu, J; Liu, J; Meng, WH; Zhao, QC, CELLULAR AND MOLECULAR NEUROBIOLOGY,37, 3, 499-509, DOI: 10.1007/s10571-016-0387-7, APR 2017
Diclofenac	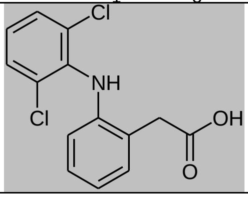	Inhibition of striatal cholinergic interneuron activity by the Kv7 opener retigabine and the nonsteroidal anti-inflammatory drug diclofenac, Paz, RM; Tubert, C; Stahl, A; Diaz, AL; Etchenique, R; Murer, MG; Rela, L, NEUROPHARMACOLOGY,137, 309-321, DOI: 10.1016/j.neuropharm.2018.05.010, JUL 15 2018

Dieckol	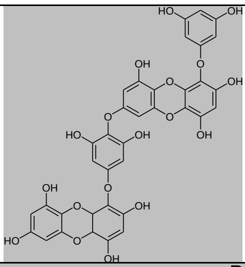	Dieckol, an edible seaweed polyphenol, retards rotenone-induced neurotoxicity and alpha-synuclein aggregation in human dopaminergic neuronal cells, Cha, SH; Heo, SJ; Jeon, YJ; Park, SM, RSC ADVANCES,6, 111, 110040-110046, DOI: 10.1039/c6ra21697h, 2016
1-[(2,3-Dihydro-1-benzofuran-2-yl) methyl] piperazine derivatives	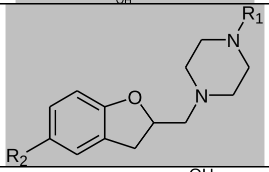	Pharmacological Characterization of 5-Substituted 1-piperazines: Novel Antagonists for the Histamine H-3 and H-4 Receptors with Anti-inflammatory Potential, Correa, MF; Barbosa, AJR; Teixeira, LB; Duarte, DA; Simoes, SC; Parreiras-e-Silva, LT; Balbino, AM; Landgraf, RG; Bouvier, M; Costa-Neto, CM et al, FRONTIERS IN PHARMACOLOGY,8, Article Number: 825, DOI: 10.3389/fphar.2017.00825, NOV 14 2017
Dihydromyricetin	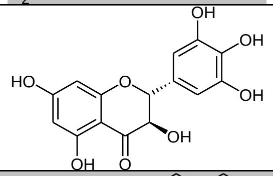	Dihydromyricetin protects neurons in an MPTP-induced model of Parkinson's disease by suppressing glycogen synthase kinase-3 beta activity, Ren, ZX; Zhao, YF; Cao, T; Zhen, XC, ACTA PHARMACOLOGICA SINICA,37, 10, 1315-1324, DOI: 10.1038/aps.2016.42, OCT 2016
3,4-Dihydro-2(1H)-quinolinone derivatives	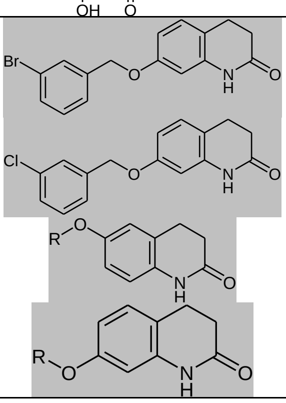	C6- and C7-Substituted 3,4-dihydro-2-quinolinones as Inhibitors of Monoamine Oxidase, Meiring, L; Petzer, JP; Petzer, A, DRUG RESEARCH,67, 3, 170-178, DOI: 10.1055/s-0042-120116, MAR 2017
5-(3,4-Dihydroxybenzylidene) -2,2-dimethyl-1,3-dioxane-4,6-dione	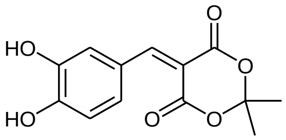	KM-34, a Novel Antioxidant Compound, Protects against 6-Hydroxydopamine-Induced Mitochondrial Damage and Neurotoxicity., Fonseca-Fonseca, Luis Arturo; Nunez-Figueredo, Yanier; Sanchez, Jeney Ramirez; Guerra, Maylin Wong; Ochoa-Rodriguez, Estael; Verdecia-Reyes, Yamila; Hernadez, Rene Delgado; Menezes-Filho, Noelio J; Costa, Teresa Cristina Silva; de Santana, Wagno Alcantara et al, Neurotoxicity research, DOI: 10.1007/s12640-017-9851-5, 2018-Jan-02
7,8-Dihydroxycoumarin derivative DHC12	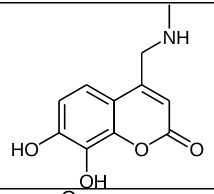	New Perspectives in Iron Chelation Therapy for the Treatment of Neurodegenerative Diseases. Nunez, MT; Chana-Cuevas, P. PHARMACEUTICALS, 11, 4, Article Number: UNSP 109, DOI: 10.3390/ph11040109, DEC 2018, **Document Type: Review**
3',4'-Dihydroxyflavone	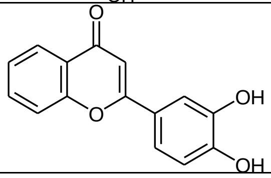	Synthetic 3 ',4 '-Dihydroxyflavone Exerts Anti-Neuroinflammatory Effects in BV2 Microglia and a Mouse Model, Kim, N; Yoo, HS; Ju, YJ; Oh, MS; Lee, KT; Inn, KS; Kim, NJ; Lee, JK, BIOMOLECULES & THERAPEUTICS,26, 2, 210-217, DOI: 10.4062/biomolther.2018.008, MAR 2018
7,8-Dihydroxyflavone	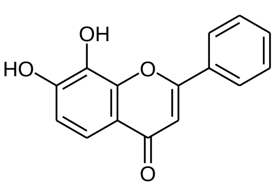	**1)** 7,8-Dihydroxyflavone Protects Nigrostriatal Dopaminergic Neurons from Rotenone-Induced Neurotoxicity in Rodents, Nie, SK; Ma, K; Sun, MK; Lee, M; Tan, Y; Chen, GQ; Zhang, ZT; Zhang, ZH; Cao, XB, PARKINSONS DISEASE, Article Number: 9193534, DOI: 10.1155/2019/9193534, 2019, **2)** Neuroprotective Effects of 7, 8-dihydroxyflavone on Midbrain Dopaminergic Neurons in MPP+-treated Monkeys, He, JJ; Xiang, Z; Zhu, XQ; Ai, ZY; Shen, JS; Huang, TZ; Liu, LG; Ji, WZ; Li, TQ, SCIENTIFIC REPORTS,6, Article Number: 34339, DOI: 10.1038/srep34339, OCT 12 2016
5,7-Dihydroxy-4'-methoxyflavone	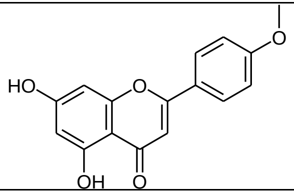	Acacetin inhibits neuronal cell death induced by 6-hydroxydopamine in cellular Parkinson's disease model, Kim, SM; Park, YJ; Shin, MS; Kim, HR; Kim, MJ; Lee, SH; Yun, SP; Kwon, SH, BIOORGANIC & MEDICINAL CHEMISTRY LETTERS,27, 23, 5207-5212, DOI: 10.1016/j.bmcl.2017.10.048, DEC 1 2017

(E)-3,4-Dihydroxystyryl aralkyl sulfones	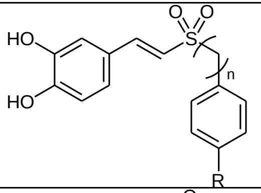	Neuroprotective effects of -3,4-diacetoxystyryl sulfone and sulfoxide derivatives in vitro models of Parkinson's disease, Ning, XL; Yuan, MM; Guo, Y; Tian, C; Wang, XW; Zhang, ZL; Liu, JY, JOURNAL OF ENZYME INHIBITION AND MEDICINAL CHEMISTRY,31, 3, 464-469, DOI: 10.3109/14756366.2015.1037750, MAY 3 2016
(E)-3,4-Dihydroxystyryl aralkyl sulfoxides	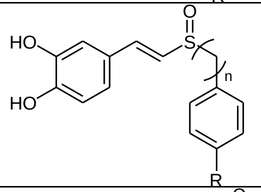	Neuroprotective effects of -3,4-diacetoxystyryl sulfone and sulfoxide derivatives in vitro models of Parkinson's disease, Ning, XL; Yuan, MM; Guo, Y; Tian, C; Wang, XW; Zhang, ZL; Liu, JY, JOURNAL OF ENZYME INHIBITION AND MEDICINAL CHEMISTRY,31, 3, 464-469, DOI: 10.3109/14756366.2015.1037750, MAY 3 2016
5,3'-dihydroxy-3,7,4'-trimethoxyflavone	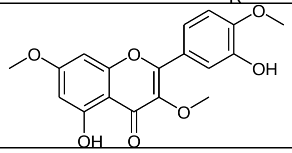	Involvement of heme oxygenase-1 induction in the cytoprotective and neuroinflammatory activities of Siegesbeckia Pubescens isolated from 5,3'-dihydroxy-3,7,4'-trimethoxyflavone in HT22 cells and BV2 cells, Lee, DS; Lee, M; Sung, SH; Jeong, GS, INTERNATIONAL IMMUNOPHARMACOLOGY,40, 65-72, DOI: 10.1016/j.intimp.2016.08.030, NOV 2016
3,3'-Diindolylmethane	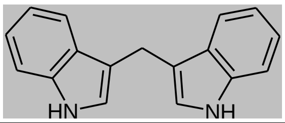	Selective Aryl Hydrocarbon Receptor Modulator 3,3'-Diindolylmethane Impairs AhR and ARNT Signaling and Protects Mouse Neuronal Cells Against Hypoxia, Rzemieniec, J; Litwa, E; Wnuk, A; Lason, W; Krzeptowski, W; Kajta, M, MOLECULAR NEUROBIOLOGY,53, 8, 5591-5606, DOI: 10.1007/s12035-015-9471-0, OCT 2016
Dimerumic acid	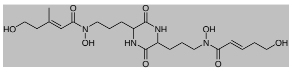	Neuroprotective effects of dimerumic acid and deferricoprogen from Monascus purpureus NTU 568-fermented rice against 6-hydroxydopamine-induced oxidative stress and apoptosis in differentiated pheochromocytoma PC-12 cells, Tseng, WT; Hsu, YW; Pan, TM, PHARMACEUTICAL BIOLOGY,54, 8, 1434-1444, DOI: 10.3109/13880209.2015.1104698, AUG 2016
2-[[(1,1-Dimethylethyl) oxidoimino]-methyl]-3,5,6-trimethylpyrazine	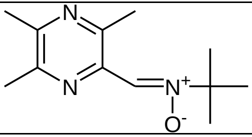	Tetramethylpyrazine Nitrone Improves Neurobehavioral Functions and Confers Neuroprotection on Rats with Traumatic Brain Injury, Zhang, GX; Zhang, F; Zhang, T; Gu, JB; Li, CM; Sun, YW; Yu, P; Zhang, ZJ; Wang, YQ, NEUROCHEMICAL RESEARCH,41, 11, 2948-2957, DOI: 10.1007/s11064-016-2013-y, NOV 2016
Dipentylammonium	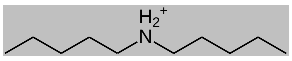	Dipentylammonium Binds to the Sigma-1 Receptor and Protects Against Glutamate Toxicity, Attenuates Dopamine Toxicity and Potentiates Neurite Outgrowth in Various Cultured Cell Lines, Brimson, JM; Safrany, ST; Qassam, H; Tencomnao, T, NEUROTOXICITY RESEARCH,34, 2, 263-272, DOI: 10.1007/s12640-018-9883-5, AUG 2018
Dipraglurant	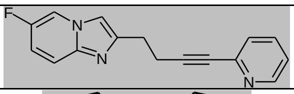	Advances in Drug Development for Parkinson's Disease: Present Status. Reddy, DH; Misra, S; Medhi, B. PHARMACOLOGY, 93, 5-6, 260-271, DOI: 10.1159/000362419, 2014, **Document Type: Review**
Diphenyleneiodonium	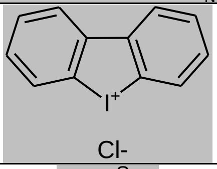	Subpicomolar Diphenyleneiodonium Inhibits Microglial NADPH Oxidase with High Specificity and Shows Great Potential as a Therapeutic Agent for Neurodegenerative Diseases, Wang, QS; Chu, CH; Oyarzabal, E; Jiang, LL; Chen, SH; Wilson, B; Qian, L; Hong, JS, GLIA,62, 12, 2034-2043, DOI: 10.1002/glia.22724, DEC 2014
Dithiolethiones	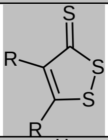	Further structure-activity relationships study of substituted dithiolethiones as glutathione-inducing neuroprotective agents, Brown, DA; Betharia, S; Yen, JH; Kuo, PC; Mistry, H, CHEMISTRY CENTRAL JOURNAL,10, Article Number: 64, DOI: 10.1186/s13065-016-0210-z, OCT 19 2016
Dizocilpine	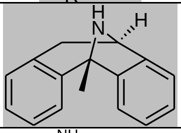	Advances in non-dopaminergic treatments for Parkinson's disease. Stayte, S; Vissel, B; FRONTIERS IN NEUROSCIENCE, 8, Article Number: 113, DOI: 10.3389/fnins.2014.00113, MAY 22 2014, **Document Type: Review**
DKP	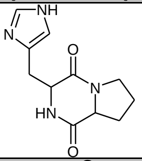	Repurposing of Copper -chelating Drugs for the Treatment of Neurodegenerative Diseases. Lanza, V; Milardi, D; Di Natale, G; Pappalardo, G. CURRENT MEDICINAL CHEMISTRY, 25, 4, 525-539, DOI: 10.2174/0929867324666170518094404, 2018, **Document type: review**
DMPX	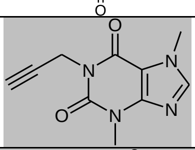	Repurposing of Copper -chelating Drugs for the Treatment of Neurodegenerative Diseases. Lanza, V; Milardi, D; Di Natale, G; Pappalardo, G. CURRENT MEDICINAL CHEMISTRY, 25, 4, 525-539, DOI: 10.2174/0929867324666170518094404, 2018, **Document type: review**
Domperidone	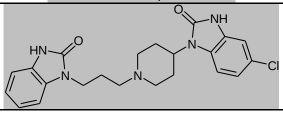	Evaluation and comparison of different brands of domperidone tablets available in Karachi, Pakistan, Khan, MQ; Razvi, N; Anjum, F; Ghazal, L; Siddiqui, SA; Shoaib, MH, PAKISTAN JOURNAL OF PHARMACEUTICAL SCIENCES,27, 4, 935-938, JUL 2014

Donepezil	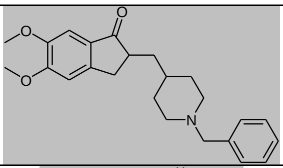	Current and experimental treatments of Parkinson disease: A guide for neuroscientists. Oertel, W; Schulz, JB. JOURNAL OF NEUROCHEMISTRY, 139, 325-337, Supplement: 1, DOI: 10.1111/jnc.13750, OCT 2016, **Document Type: Review**
Docosahexaenoic acid	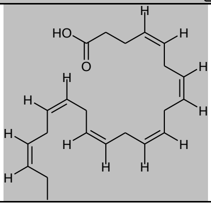	Advances in Drug Development for Parkinson's Disease: Present Status. Reddy, DH; Misra, S; Medhi, B. PHARMACOLOGY, 93, 5-6, 260-271, DOI: 10.1159/000362419, 2014, **Document Type: Review**
L-DOPA (levodopa, CVT-301)	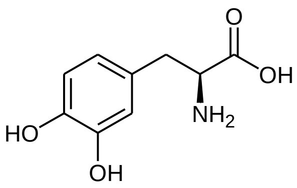	**1)** Intranasal administration of sodium dimethyldithiocarbamate induces motor deficits and dopaminergic dysfunction in mice, Mack, JM; Moura, TM; Lanznaster, D; Bobinski, F; Massari, CM; Sampaio, TB; Schmitz, AE; Souza, LF; Walz, R; Tasca, CI et al, NEUROTOXICOLOGY,66, 107-120, DOI: 10.1016/j.neuro.2018.03.011, MAY 2018, **2)** Pharmacological characterization of nicotine-induced tremor: Responses to anti-tremor and anti-epileptic agents, Kunisawa, N; Shimizu, S; Kato, M; Iha, HA; Iwai, C; Hashimura, M; Ogawa, M; Kawaji, S; Kawakita, K; Abe, K et al, JOURNAL OF PHARMACOLOGICAL SCIENCES,137, 2, 162-169, DOI: 10.1016/j.jphs.2018.05.007, JUN 2018, **3)** Liquid levodopa-carbidopa in advanced Parkinson's disease with motor complications, Yang, HJ; Ehm, G; Kim, YE; Yun, JY; Lee, WW; Kim, A; Kim, HJ; Jeon, B, JOURNAL OF THE NEUROLOGICAL SCIENCES,377, 6-11, DOI: 10.1016/j.jns.2017.03.039, JUN 15 2017 **4) et cetera**
DOPA-derived peptido-mimetics (deprotected)	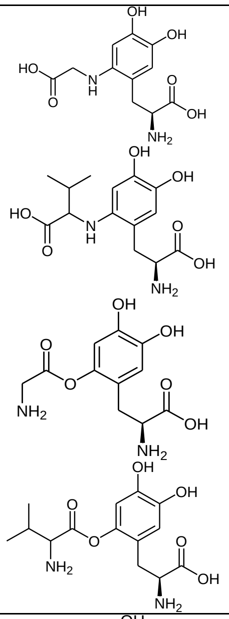	Tyrosinase mediated oxidative functionalization in the synthesis of DOPA-derived peptidomimetics with anti-Parkinson activity, Bizzarri, BM; Martini, A; Serafini, F; Aversa, D; Piccinino, D; Botta, L; Berretta, N; Guatteo, E; Saladino, R, RSC ADVANCES,7, 33, 20502-20509, DOI: 10.1039/c7ra03326e, 2017
DOPA-derived peptido-mimetics (protected)	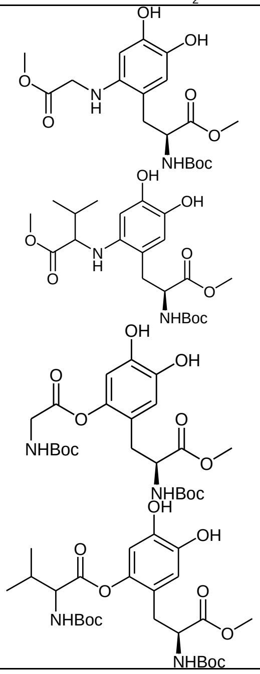	Tyrosinase mediated oxidative functionalization in the synthesis of DOPA-derived peptidomimetics with anti-Parkinson activity, Bizzarri, BM; Martini, A; Serafini, F; Aversa, D; Piccinino, D; Botta, L; Berretta, N; Guatteo, E; Saladino, R, RSC ADVANCES,7, 33, 20502-20509, DOI: 10.1039/c7ra03326e, 2017

L-DOPA deuterated (D3-L-DOPA)		Deuterium-substituted L-DOPA displays increased behavioral potency and dopamine output in an animal model of Parkinson's disease: comparison with the effects produced by L-DOPA and an MAO-B inhibitor, Malmlof, T; Feltmann, K; Konradsson-Geuken, A; Schneider, F; Alken, RG; Svensson, TH; Schilstrom, B, JOURNAL OF NEURAL TRANSMISSION,122, 2, 259-272, DOI: 10.1007/s00702-014-1247-6, FEB 2015
Doxycycline	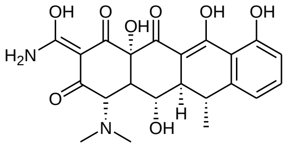	**1)** Repurposing doxycycline for synucleinopathies: remodelling of alpha-synuclein oligomers towards non-oxic parallel beta-sheet structured species, Gonzalez-Lizarraga, F; Socias, SB; Avila, CL; Torres-Bugeau, CM; Barbosa, LRS; Binolfi, A; Sepulveda-Diaz, JE; Del-Bel, E; Fernandez, CO; Papy-Garcia, D et al, SCIENTIFIC REPORTS,7, Article Number: 41755, DOI: 10.1038/srep41755, FEB 3 2017, **2)** Doxycycline Suppresses Microglial Activation by Inhibiting the p38 MAPK and NF-kB Signaling Pathways, Santa-Cecilia, FV; Socias, B; Ouidja, MO; Sepulveda-Diaz, JE; Acuna, L; Silva, RL; Michel, PP; Del-Bel, E; Cunha, TM; Raisman-Vozari, R, NEUROTOXICITY RESEARCH,29, 4, 447-459, DOI: 10.1007/s12640-015-9592-2, MAY 2016
5-OH-DPAT	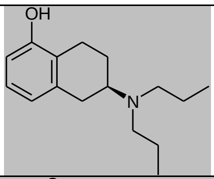	Development of Multifunctional Molecules as Potential Therapeutic Candidates for Alzheimer's Disease, Parkinson's Disease, and Amyotrophic Lateral Sclerosis in the Last Decade. Savelieff, MG; Nam, G; Kang, J; Lee, HJ; Lee, M; Lim, MH. CHEMICAL REVIEWS, 119, 2, 1221-1322, DOI: 10.1021/acs.chemrev.8b00138, JAN 23 2019, **Document Type: Review**
DPMTX	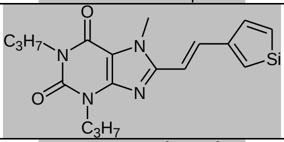	Xanthine derivatives as agents affecting non-dopaminergic neuroprotection in Parkinson`s disease., Kasabova-Angelova, A; Tzankova, D; Mitkov, J; Georgieva, M; Tzankova, V; Zlatkov, A; Kondeva-Burdina, M, Current medicinal chemistry, DOI:10.2174/0929867325666180821153316, 2018-Aug-21
Duloxetine	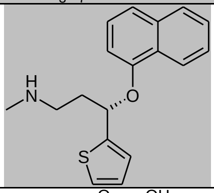	Current and experimental treatments of Parkinson disease: A guide for neuroscientists. Oertel, W; Schulz, JB. JOURNAL OF NEUROCHEMISTRY, 139, 325-337, Supplement: 1, DOI: 10.1111/jnc.13750, OCT 2016, **Document Type: Review**
Droxidopa	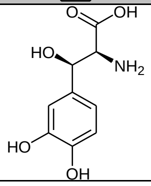	**1)** Current approaches to the treatment of Parkinson's Disease, Ellis, JM; Fell, MJ, BIOORGANIC & MEDICINAL CHEMISTRY LETTERS, 27, 18, 4247-4255, DOI: 10.1016/j.bmcl.2017.07.075, Published: SEP 15 2017**, 2)** Current and experimental treatments of Parkinson disease: A guide for neuroscientists. Oertel, W; Schulz, JB. JOURNAL OF NEUROCHEMISTRY, 139, 325-337, Supplement: 1, DOI: 10.1111/jnc.13750, OCT 2016, **Document Type: Review**
Dulaglutide		Parkinson's Disease, Diabetes and Cognitive Impairment, Ashraghi, MR; Pagano, G; Polychronis, S; Niccolini, F; Politis, M, RECENT PATENTS ON ENDOCRINE METABOLIC & IMMUNE DRUG DISCOVERY,10, 1, 11-21, DOI: 10.2174/1872214810999160628105549, 2016, **Document Type: Review**
β-Ecdysterone (β-Ecd)	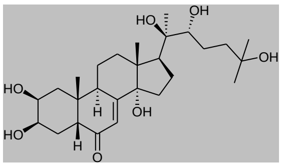	**1)** Synergistic protective effect of paeoniflorin and beta-ecdysterone against rotenone-induced neurotoxicity in PC12 cells, Liu, H; Yu, CL; Xu, TJ; Zhang, XJ; Dong, MX; APOPTOSIS, 21, 12, 1354-1365, DOI: 10.1007/s10495-016-1293-7, DEC 2016, **2)** beta-Ecdysterone Protects SH-SY5Y Cells Against 6-Hydroxydopamine-Induced Apoptosis via Mitochondria-Dependent Mechanism: Involvement of p38-p53 Signaling Pathway, Pan, Z; Niu, YC; Liang, YN; Zhang, XJ; Dong, MX; NEUROTOXICITY RESEARCH, 30, 3, 453-466, DOI: 10.1007/s12640-016-9631-7, OCT 2016
Echinacoside	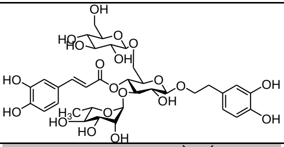	Echinacoside protects against MPTP/MPP+-induced neurotoxicity via regulating autophagy pathway mediated by Sirt1, Chen, C; Xia, BM; Tang, LL; Wu, W; Tang, JJ; Liang, Y; Yang, H; Zhang, ZN; Lu, Y; Chen, G et al, METABOLIC BRAIN DISEASE,34, 1, 203-212, DOI: 10.1007/s11011-018-0330-3, FEB 2019
Echinomycin	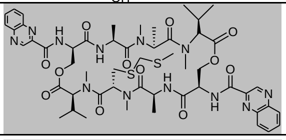	miRNA profile of neuroprotection mechanism of echinomycin in Parkinson's disease, Kwon, D; Liew, H, MOLECULAR & CELLULAR TOXICOLOGY,13, 2, 229-238, DOI: 10.1007/s13273-017-0025-6, JUN 2017
Eicosanoyl-5-hydroxytryptamide	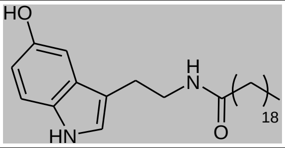	Eicosanoyl-5-hydroxytryptamide prevents Alzheimer's disease-related cognitive and electrophysiological impairments in mice exposed to elevated concentrations of oligomeric beta-amyloid, Asam, K; Staniszewski, A; Zhang, H; Melideo, SL; Mazzeo, A; Voronkov, M; Huber, KL; Perez, E; Stock, M; Stock, JB et al, PLOS ONE,12, 12, Article Number: e0189413, DOI: 10.1371/journal.pone.0189413, DEC 18 2017
Eleutherosides B, B1, E	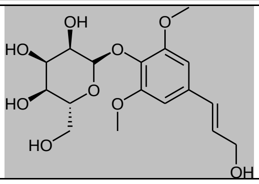	**1)** Acanthopanax senticosus Protects Structure and Function of Mesencephalic Mitochondria in A Mouse Model of Parkinson's Disease, Liu, SM; Li, XZ; Zhang, SN; Yang, ZM; Wang, KX; Lu, F; Wang, CZ; Yuan, CS; CHINESE JOURNAL OF INTEGRATIVE MEDICINE, 24, 11, 835-843, DOI: 10.1007/s11655-018-2935-5, NOV 2018, **2)** Recent advances in discovery and development of natural products as source for anti-Parkinson's disease lead compounds, Zhang, HJ; Bai, L; He, J; Zhong, L; Duan, XM; Ouyang, L; Zhu, YX; Wang, T; Shi, JY;

	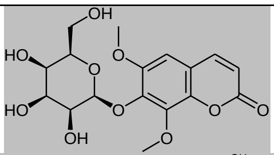 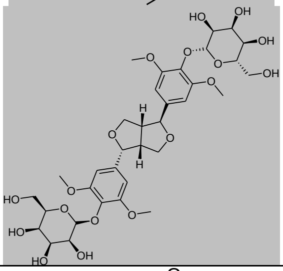	Zhang, YW, EUROPEAN JOURNAL OF MEDICINAL CHEMISTRY,141, 257-272, DOI: 10.1016/j.ejmech.2017.09.068, DEC 1 2017, **Document Type:Review**
Ellagic acid	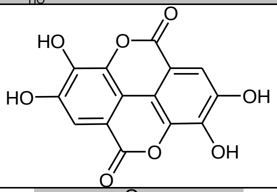	Molecular recognition between potential natural inhibitors of the Keap1-Nrf2 complex, Bello, M; Morales-Gonzalez, JA, INTERNATIONAL JOURNAL OF BIOLOGICAL MACROMOLECULES,105, 981-992 Part: 1, DOI: 10.1016/j.ijbiomac.2017.07.117, DEC 2017
ELN484228	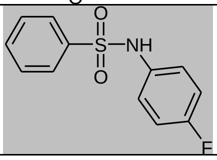	Development of Multifunctional Molecules as Potential Therapeutic Candidates for Alzheimer's Disease, Parkinson's Disease, and Amyotrophic Lateral Sclerosis in the Last Decade. Savelieff, MG; Nam, G; Kang, J; Lee, HJ; Lee, M; Lim, MH. CHEMICAL REVIEWS, 119, 2, 1221-1322, DOI: 10.1021/acs.chemrev.8b00138, JAN 23 2019, **Document Type: Review**
Eltoprazine	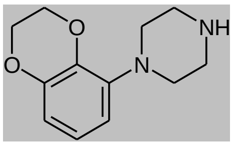	**1)** Current approaches to the treatment of Parkinson's Disease, Ellis, JM; Fell, MJ, BIOORGANIC & MEDICINAL CHEMISTRY LETTERS, 27, 18, 4247-4255, DOI: 10.1016/j.bmcl.2017.07.075, Published: SEP 15 2017, **2)** A preclinical study on the combined effects of repeated eltoprazine and preladenant treatment for alleviating L-DOPA-induced dyskinesia in Parkinson's disease, Ko, WKD; Li, Q; Cheng, LY; Morelli, M; Carta, M; Bezard, E, EUROPEAN JOURNAL OF PHARMACOLOGY,813, 10-16, DOI: 10.1016/j.ejphar.2017.07.030, OCT 15 2017
Entacapone (Comtan, ASI-6)	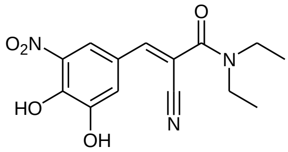	**1)** Development of Multifunctional Molecules as Potential Therapeutic Candidates for Alzheimer's Disease, Parkinson's Disease, and Amyotrophic Lateral Sclerosis in the Last Decade. Savelieff, MG; Nam, G; Kang, J; Lee, HJ; Lee, M; Lim, MH. CHEMICAL REVIEWS, 119, 2, 1221-1322, DOI: 10.1021/acs.chemrev.8b00138, JAN 23 2019, **Document Type:Review**, **2)** GLYCINE AND GOLD NANOPARTICLES FOR THE ELECTROCHEMICAL DETERMINATION OF AN ANTI-PARKINSON'S DRUG IN A TERTIARY MIXTURE, Azab, SM; Fekry, AM; Mohamed, MA, INTERNATIONAL JOURNAL OF PHARMACEUTICAL SCIENCES AND RESEARCH,8, 11, 4839-4847, DOI: 10.13040/IJPSR.0975-8232.8.4839-47, NOV 2017, **3)** Potential of microemulsified entacapone drug delivery systems in the management of acute Parkinson's disease, Vadlamudi, HC; Yalavarthi, PR; Venkata, BRM; Thanniru, J; Vandana, KR; Sundaresan, CR, JOURNAL OF ACUTE DISEASE,5, 4, 315-325, DOI: 10.1016/j.joad.2016.05.004, JUL 1 2016
Enzastaurin	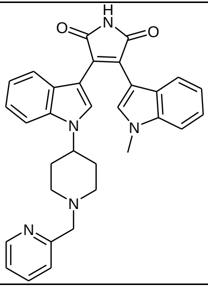	Marine-derived protein kinase inhibitors for neuroinflammatory diseases, Ning, C; Wang, HMD; Gao, R; Chang, YC; Hu, FQ; Meng, XJ; Huang, SY, BIOMEDICAL ENGINEERING ONLINE,17, Article Number: 46, DOI: 10.1186/s12938-018-0477-5, APR 24 2018, **Document Type:Review**
Epicatechin	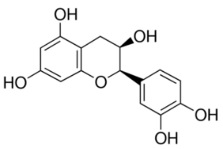	**1)** Flavonoids and its Neuroprotective Effects on Brain Ischemia and Neurodegenerative Diseases, Putteeraj, M; Lim, WL; Teoh, SL; Yahaya, MF, CURRENT DRUG TARGETS,19, 14, 1710-1720, DOI: 10.2174/1389450119666180326125252, 2018, **Document Type:Review, 2)** Natural polyphenols effects on protein aggregates in Alzheimer's and Parkinson's prion-like diseases, Freyssin, A; Page, G; Fauconneau, B; Bilan, AR, NEURAL REGENERATION RESEARCH,13, 6, 955-961, DOI: 10.4103/1673-5374.233432, JUN 2018, **Document Type:Review**, **3)** Polyphenols in Parkinson's Disease: A Systematic Review of In Vivo Studies, Kujawska, M; Jodynis-Liebert, J, NUTRIENTS,10, 5, Article Number: 642, DOI: 10.3390/nu10050642, MAY 2018, **Document Type:Review**, **4)** Novel tactics for neuroprotection in Parkinson's disease: Role of antibiotics, polyphenols and neuropeptides, Reglodi, D; Renaud, J; Tamas, A; Tizabi, Y; Socias, SB; Del-Bel, E; Raisman-Vozari, R, PROGRESS IN NEUROBIOLOGY,155, 120-148 Special : SI, DOI: 10.1016/j.pneurobio.2015.10.004, AUG 2017, **Document Type:Review**, **5) et cetera**

Epigallocatechin-3-gallate	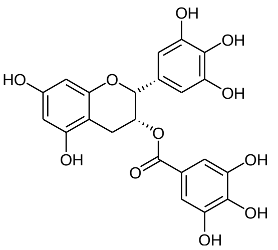	**1)** Effects and mechanism of epigallocatechin-3-gallate on apoptosis and mTOR/AKT/GSK-3 beta pathway in substantia nigra neurons in Parkinson rats, Zhou, W; Chen, L; Hu, XQ; Cao, SS; Yang, JX, NEUROREPORT,30, 2, 60-65, DOI: 10.1097/WNR.0000000000001149, JAN 16 2019, **2)** Recent advances in discovery and development of natural products as source for anti-Parkinson's disease lead compounds, Zhang, HJ; Bai, L; He, J; Zhong, L; Duan, XM; Ouyang, L; Zhu, YX; Wang, T; Shi, JY; Zhang, YW, EUROPEAN JOURNAL OF MEDICINAL CHEMISTRY,141, 257-272, DOI: 10.1016/j.ejmech.2017.09.068, DEC 1 2017, **Document Type:Review, 3)** Molecular interaction studies of green tea catechins as multitarget drug candidates for the treatment of Parkinson's disease: computational and structural insights, Azam, F; Mohamed, N; Alhussen, F, NETWORK-COMPUTATION IN NEURAL SYSTEMS,26, 3-4, 97-115, DOI: 10.3109/0954898X.2016.1146416, OCT 2 2015, **4)** Epigallocatechin-3-Gallate, a Promising Molecule for Parkinson's Disease?, Renaud, J; Nabavi, SF; Daglia, M; Nabavi, SM; Martinoli, MG, REJUVENATION RESEARCH,18, 3, 257-269, DOI: 10.1089/rej.2014.1639, JUN 1 2015, **Document Type:Review**, **5) et cetera**
Epothilone B	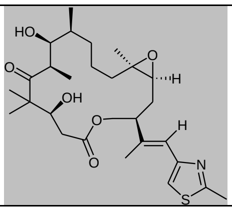	Epothilone B Benefits Nigral Dopaminergic Neurons by Attenuating Microglia Activation in the 6-Hydroxydopamine Lesion Mouse Model of Parkinson's Disease, Yu, ZY; Yang, L; Yang, Y; Chen, SY; Sun, DY; Xu, HW; Fan, XT, FRONTIERS IN CELLULAR NEUROSCIENCE,12, Article Number: 324, DOI: 10.3389/fncel.2018.00324, SEP 28 2018
Erucin	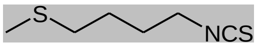	Comparison of Adaptive Neuroprotective Mechanisms of Sulforaphane and its Interconversion Product Erucin in in Vitro and in Vivo Models of Parkinson's Disease, Morroni, F; Sita, G; Djemil, A; D'Amico, M; Pruccoli, L; Cantelli-Forti, G; Hrelia, P; Tarozzi, A, JOURNAL OF AGRICULTURAL AND FOOD CHEMISTRY,66, 4, 856-865, DOI: 10.1021/acs.jafc.7b04641, JAN 31 2018
Erythropoietin		**1)** The ongoing pursuit of neuroprotective therapies in Parkinson disease, Athauda, D; Foltynie, T, NATURE REVIEWS NEUROLOGY,11, 1, 25-40, DOI: 10.1038/nrneurol.2014.226, JAN 2015, **Document Type:Review**, **2)** Evidence that erythropoietin modulates neuroinflammation through differential action on neurons, astrocytes, and microglia, Bond, WS; Rex, TS, FRONTIERS IN IMMUNOLOGY,5, Article Number: 523, DOI: 10.3389/fimmu.2014.00523, OCT 22 2014, **Document Type:Review**
Escitalopram	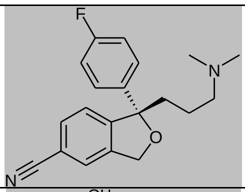	PET imaging of dopamine transporters with FE-PE2I: Effects of anti-Parkinsonian drugs, Bang, JI; Jung, IS; Song, YS; Park, HS; Moon, BS; Lee, BC; Kim, SE, NUCLEAR MEDICINE AND BIOLOGY,43, 2, 158-164, DOI: 10.1016/j.nucmedbio.2015.11.002, FEB 2016
Esculin	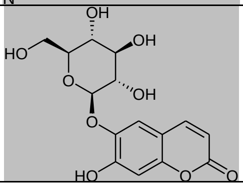	Recent advances in discovery and development of natural products as source for anti-Parkinson's disease lead compounds, Zhang, HJ; Bai, L; He, J; Zhong, L; Duan, XM; Ouyang, L; Zhu, YX; Wang, T; Shi, JY; Zhang, YW, EUROPEAN JOURNAL OF MEDICINAL CHEMISTRY,141, 257-272, DOI: 10.1016/j.ejmech.2017.09.068, DEC 1 2017, **Document Type:Review**
Estradiol	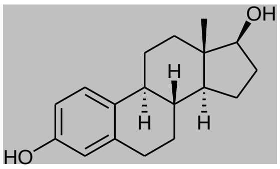	**1)** Mechanistic evaluation of neuroprotective effect of estradiol on rotenone and 6-OHDA induced Parkinson's disease, Shen, DF; Tian, XY; Zhang, BB; Song, RR, PHARMACOLOGICAL REPORTS,69, 6, 1178-1185, DOI: 10.1016/j.pharep.2017.06.008, DEC 2017, **2)** 17 beta-estradiol/n-acetylcysteine interaction enhances the neuroprotective effect on dopaminergic neurons in the weaver model of dopamine deficiency, Botsakis, K; Theodoritsi, S; Grintzalis, K; Angelatou, F; Antonopoulos, I; Georgiou, CD; Margarity, M; Matsokis, NA; Panagopoulos, NT, NEUROSCIENCE,320, 221-229, DOI: 10.1016/j.neuroscience.2016.01.068, APR 21 2016
7 β-(3-Ethylcis-crotonoyloxy)-1 alpha-(2-methylbutyryloxy)-3,14-dehydro-Z-notonipetranone	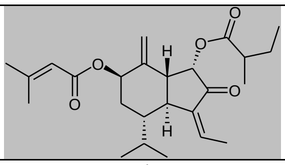	Neuroprotection against 6-OHDA toxicity in PC12 cells and mice through the Nrf2 pathway by a sesquiterpenoid from Tussilago farfara, Lee, J; Song, K; Huh, E; Oh, MS; Kim, YS, REDOX BIOLOGY,18, 6-15, DOI: 10.1016/.redox.2018.05.015, SEP 2018
Etidronate (HEDPA)	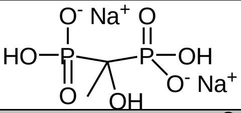	Neuroprotective Effects of Etidronate and 2,3,3-Trisphosphonate Against Glutamate-Induced Toxicity in PC12 Cells, Li, W; Cheong, YK; Wang, H; Ren, GG; Yang, Z, NEUROCHEMICAL RESEARCH,41, 4, 844-854, DOI: 10.1007/s11064-015-1761-4, APR 2016
Exemestane	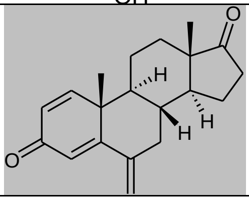	Potential repositioning of exemestane as a neuroprotective agent for Parkinson's disease, Son, HJ; Han, SH; Lee, JA; Shin, EJ; Hwang, O, FREE RADICAL RESEARCH,51, 6, 633-645, DOI: 10.1080/10715762.2017.1353688, 2017
Exenatide (Exendin-4)		**1)** Mitral cells and the glucagon-like peptide 1 receptor: The sweet smell of

		success?, Bagnoli, E; FitzGerald, U, EUROPEAN JOURNAL OF NEUROSCIENCE,49, 4, 422-439 Special : SI, DOI: 10.1111/ejn.14115, FEB 2019, **2)** Alpha-Synuclein Glycation and the Action of Anti-Diabetic Agents in Parkinson's Disease, Konig, A; Vicente Miranda, H; Outeiro, TF, JOURNAL OF PARKINSONS DISEASE, 8, 1, 33-43, DOI: 10.3233/JPD-171285, 2018, **Document Type:Review, 3)** Neuroprotective Effects of Exenatide in a Rotenone-Induced Rat Model of Parkinson's Disease, Aksoy, D; Solmaz, V; Cavusoglu, T; Meral, A; Ates, U; Erbas, O, AMERICAN JOURNAL OF THE MEDICAL SCIENCES,354, 3, 319-324, SEP 2017, **4)** Neuroprotective exendin-4 enhances hypothermia therapy in a model of hypoxic-ischaemic encephalopathy, Rocha-Ferreira, E; Poupon, L; Zelco, A; Leverin, AL; Nair, S; Jonsdotter, A; Carlsson, Y; Thornton, C; Hagberg, H; Rahim, AA, BRAIN,141, 2925-2942 Part: 10, DOI: 10.1093/brain/awy220, OCT 2018, **5)** Exendin-4 Treatment Improves LPS-Induced Depressive-Like Behavior Without Affecting ProInflammatory Cytokines, Ventorp, F; Bay-Richter, C; Nagendra, AS; Janelidze, S; Matsson, VS; Lipton, J; Nordstrom, U; Westrin, A; Brundin, P; Brundin, L, JOURNAL OF PARKINSONS DISEASE,7, 2, 263-273, DOI: 10.3233/JPD-171068, 2017, **6)** The ongoing pursuit of neuroprotective therapies in Parkinson disease, Athauda, D; Foltynie, T, NATURE REVIEWS NEUROLOGY,11, 1, 25-40, DOI: 10.1038/nrneurol.2014.226, JAN 2015, **Document Type:Review, 7)** Disease-Modifying Drugs in Parkinson's Disease, Park, A; Stacy, M, DRUGS,75, 18, 2065-2071, DOI: 10.1007/s40265-015-0497-4, DEC 2015, **Document Type:Review, 8) et cetera**
Exifone	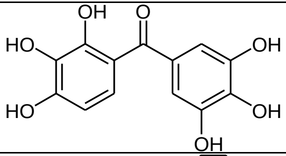	Development of Multifunctional Molecules as Potential Therapeutic Candidates for Alzheimer's Disease, Parkinson's Disease, and Amyotrophic Lateral Sclerosis in the Last Decade. Savelieff, MG; Nam, G; Kang, J; Lee, HJ; Lee, M; Lim, MH. CHEMICAL REVIEWS, 119, 2, 1221-1322, DOI: 10.1021/acs.chemrev.8b00138, JAN 23 2019, **Document Type: Review**
F13714	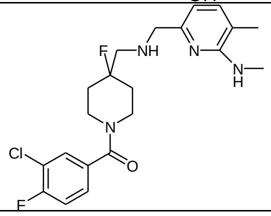	Effects of the Serotonin 5-HT1A Receptor Biased Agonists, F13714 and F15599, on Striatal Neurotransmitter Levels Following l-DOPA Administration in Hemi-Parkinsonian Rats, Newman-Tancredi, A; Varney, MA; McCreary, AC, NEUROCHEMICAL RESEARCH,43, 5, 1035-1046, DOI: 10.1007/s11064-018-2514-y, MAY 2018
F15599	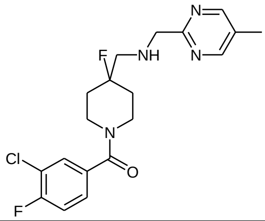	**1)** Effects of the Serotonin 5-HT1A Receptor Biased Agonists, F13714 and F15599, on Striatal Neurotransmitter Levels Following l-DOPA Administration in Hemi-Parkinsonian Rats, Newman-Tancredi, A; Varney, MA; McCreary, AC, NEUROCHEMICAL RESEARCH,43, 5, 1035-1046, DOI: 10.1007/s11064-018-2514-y, MAY 2018, **2)** The highly-selective 5-HT1A agonist F15599 reduces L-DOPA-induced dyskinesia without compromising anti-parkinsonian benefits in the MPTP-lesioned macaque, Huot, P; Johnston, TH; Fox, SH; Newman-Tancredi, A; Brotchie, JM, NEUROPHARMACOLOGY,97, 306-311, DOI: 10.1016/j.neuropharm.2015.05.033, OCT 2015
Farrerol	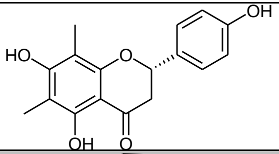	Farrerol attenuates MPP+ -induced inflammatory response by TLR4 signaling in a microglia cell line, Cui, Bei; Guo, Xiaqing; You, Yanhui; Fu, Rongli, Phytotherapy research: PTR, DOI:10.1002/ptr.6307, 2019-Feb-08
Fascaplysin	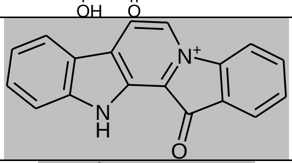	Marine-derived protein kinase inhibitors for neuroinflammatory diseases, Ning, C; Wang, HMD; Gao, R; Chang, YC; Hu, FQ; Meng, XJ; Huang, SY, BIOMEDICAL ENGINEERING ONLINE,17, Article Number: 46, DOI: 10.1186/s12938-018-0477-5, APR 24 2018, **Document Type:Review**
Fasudil	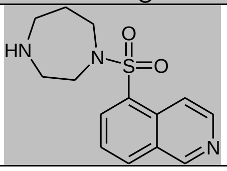	Fasudil attenuates aggregation of alpha-synuclein in models of Parkinson's disease, Tatenhorst, L; Eckermann, K; Dambeck, V; Fonseca-Ornelas, L; Walle, H; da Fonseca, TL; Koch, JC; Becker, S; Toenges, L; Bahr, M et al, ACTA NEUROPATHOLOGICA COMMUNICATIONS,4, Article Number: UNSP 39, DOI: 10.1186/s40478-016-0310-y, APR 22 2016
Fatty acids	R-COOH	**1),** Connection between Systemic Inflammation and Neuroinflammation Underlies Neuroprotective Mechanism of Several Phytochemicals in Neurodegenerative Diseases, Wang, JT; Song, YT; Chen, Z; Leng, SAX, OXIDATIVE MEDICINE AND CELLULAR LONGEVITY, Article Number: 1972714, DOI: 10.1155/2018/1972714, 2018, **Document Type: Review, 2)** Targeting mTORs by omega-3 fatty acids: A possible novel therapeutic strategy for neurodegeneration?, Shirooie, S; Nabavi, SF; Dehpour, AR; Belwal, T; Habtemariam, S; Arguelles, S; Sureda, A; Daglia, M; Tomczyk, M; Sobarzo-Sanchez, E et al, PHARMACOLOGICAL RESEARCH,135, 37-48, DOI: 10.1016/j.phrs.2018.07.004, SEP 2018, **Document Type:Review, 3)** Bioassay-guided Isolation of Neuroprotective Fatty Acids from Nigella sativa against 1-methyl-4-phenylpyridinium-induced Neurotoxicity, Hosseinzadeh, L; Monaghash, H; Ahmadi, F; Ghiasvand, N; Shokoohinia, Y, PHARMACOGNOSY MAGAZINE,13, 52, 627-633, DOI: 10.4103/pm.pm_470_16, OCT-DEC 2017, **4)**

		Fatty acids and their therapeutic potential in neurological disorders, Lei, E; Vacy, K; Boon, WC, NEUROCHEMISTRY INTERNATIONAL,95, 75-84, DOI: 10.1016/j.neuint.2016.02.014, MAY 2016, **Document Type: Review**
Fenbendazole	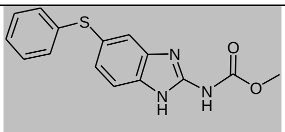 Fenbendazole	Antihelminthic Benzimidazoles Are Novel HIF Activators That Prevent Oxidative Neuronal Death via Binding to Tubulin, Aleyasin, H; Karuppagounder, SS; Kumar, A; Sleiman, S; Basso, M; Ma, T; Siddiq, A; Chinta, SJ; Brochier, C; Langley, B et al, ANTIOXIDANTS & REDOX SIGNALING, 22, 2, 121-134, DOI: 10.1089/ars.2013.5595, JAN 10 2015
Fenofibrate	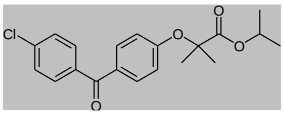	PPAR-alpha. agonist fenofibrate protects against the damaging effects of MPTP in a rat model of Parkinson's disease, Barbiero, JK; Santiago, R; Tonin, FS; Boschen, S; da Silva, LM; Werner, MFD; da Cunha, C; Lima, MMS; Vital, MABF, PROGRESS IN NEURO-PSYCHOPHARMACOLOGY & BIOLOGICAL PSYCHIATRY,53, 35-44, DOI: 10.1016/j.pnpbp.2014.02.009, AUG 4 2014
Ferulic acid	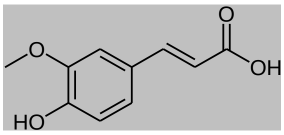	**1)** Ferulic acid and its water-soluble derivatives inhibit nitric oxide production and inducible nitric oxide synthase expression in rat primary astrocytes, Kikugawa, M; Ida, T; Ihara, H; Sakamoto, T, BIOSCIENCE BIOTECHNOLOGY AND BIOCHEMISTRY,81, 8, 1607-1611, DOI: 10.1080/09168451.2017.1336925, 2017, **2)** Protective effect of ferulic acid against 2,2 '-azobis dihydrochloride-induced oxidative stress in PC12 cells, Shen, Y; Zhang, H; Wang, L; Qian, H; Qi, Y; Miao, X; Cheng, L; Qi, X, CELLULAR AND MOLECULAR BIOLOGY,62, 1, 109-116, 2016, **3) et cetera**
FGF8b		Expression of functional recombinant human fibroblast growth factor 8b and its protective effects on MPP+-lesioned PC12 cells, Chen, NZ; Ma, JS; Zhao, Y; Wu, MY; Yang, HH; Gong, WY; Chao, J; Li, XK, APPLIED MICROBIOLOGY AND BIOTECHNOLOGY,100, 2, 625-635, DOI: 10.1007/s00253-015-7004-4, JAN 2016
Fipamezole	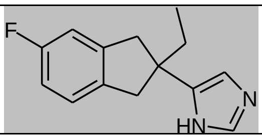	Advances in Drug Development for Parkinson's Disease: Present Status. Reddy, DH; Misra, S; Medhi, B. PHARMACOLOGY, 93, 5-6, 260-271, DOI: 10.1159/000362419, 2014, **Document Type: Review**
Fisetin (3,3',4',7-Tetrahydroxyflavone)	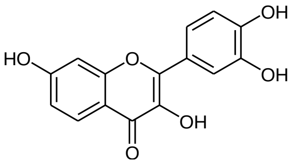	**1)** Protective Effects of Fisetin Against 6-OHDA-Induced Apoptosis by Activation of PI3K-Akt Signaling in Human Neuroblastoma SH-SY5Y Cells, Watanabe, R; Kurose, T; Morishige, Y; Fujimori, K, NEUROCHEMICAL RESEARCH,43, 2, 488-499, DOI: 10.1007/s11064-017-2445-z, FEB 2018, **2)** Neuroprotective Effects of Fisetin in Alzheimer's and Parkinson's Diseases: From Chemistry to Medicine, Nabavi, SF; Braidy, N; Habtemariam, S; Sureda, A; Manayi, A; Nabavi, SM, CURRENT TOPICS IN MEDICINAL CHEMISTRY,16, 17, 1910-1915, DOI: 10.2174/1568026616666160204121725, 2016, **Document Type: Review**, **3)** Dietary flavonoid fisetin regulates aluminium chloride-induced neuronal apoptosis in cortex and hippocampus of mice brain, Prakash, D; Sudhandiran, G, JOURNAL OF NUTRITIONAL BIOCHEMISTRY,26, 12, 1527-1539, DOI: 10.1016/j.jnutbio.2015.07.017, DEC 2015
Fluoxetine	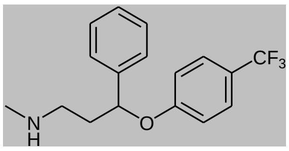	**1)** Fluoxetine-mediated inhibition of endoplasmic reticulum stress is Involved in the neuroprotective effects of Parkinson's disease, Peng, T; Liu, XY; Wang, JT; Liu, Y; Fu, ZQ; Ma, XR; Li, JM; Sun, GF; Ji, YF; Lu, JJ et al, AGING-US,10, 12, 4188-4196, DOI: 10.18632/aging.101716, DEC 2018, **2)** Effect of Buspirone, Fluoxetine and 8-OH-DPAT on Striatal Expression of Bax, Caspase-3 and Bcl-2 Proteins in 6-Hydroxydopamine-Induced Hemi-Parkinsonian Rats, Sharifi, H; Nayebi, AM; Farajnia, S; Haddadi, R, ADVANCED PHARMACEUTICAL BULLETIN,5, 4, 491-495, NOV 2015
Fluvastatin	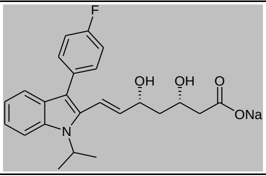	**1)** The beneficial effects of HMG-CoA reductase inhibitors in the processes of neurodegeneration, Saravi, SSS; Saravi, SSS; Arefidoust, A; Dehpour, AR, METABOLIC BRAIN DISEASE,32, 4, 949-965, DOI: 10.1007/s11011-017-0021-5, AUG 2017, **Document Type:Review**, **2)** Neuroprotective mechanisms of statins in neurodegenerative diseases, Yan, JQ; Sun, JC; Li, XM; Zhai, MM; Jia, XF, INTERNATIONAL JOURNAL OF CLINICAL AND EXPERIMENTAL MEDICINE,9, 6, 9799-9805, 2016, **Document Type:Review**
FLZ = N-[2-(4-hydroxy-phenyl)-ethyl]-2-(2,5-dimethoxy-phenyl)-3-(3-methoxy-4-hydroxy-phenyl)-acrylamide	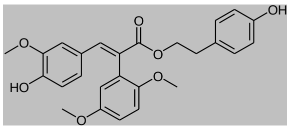	**1)** Simultaneous determination of FLZ and its metabolite in human plasma and urine by UHPLC-MS/MS: Application to a pharmacokinetic study, Cui, C; Chen, R; Jiang, J; Liu, R; Wang, W; Zhao, Q; Hu, P, JOURNAL OF PHARMACEUTICAL AND BIOMEDICAL ANALYSIS,164, 32-40, DOI: 10.1016/j.jpba.2018.10.018, FEB 5 2019, **2)** P-Glycoprotein Mediated Efflux Limits the Transport of the Novel Anti-Parkinson's Disease Candidate Drug FLZ across the Physiological and PD Pathological In Vitro BBB Models, Liu, Q; Hou, JF; Chen, XG; Liu, GT; Zhang, D; Sun, H; Zhang, JL, PLOS ONE,9, 7, Article Number: e102442, DOI: 10.1371/journal.pone.0102442, JUL 18 2014, **3)** et cetera
FNO75	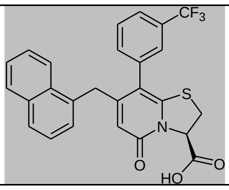	Development of Multifunctional Molecules as Potential Therapeutic Candidates for Alzheimer's Disease, Parkinson's Disease, and Amyotrophic Lateral Sclerosis in the Last Decade. Savelieff, MG; Nam, G; Kang, J; Lee, HJ; Lee, M; Lim, MH. CHEMICAL REVIEWS, 119, 2, 1221-1322, DOI: 10.1021/acs.chemrev.8b00138, JAN 23 2019, **Document Type: Review**

Foliglurax	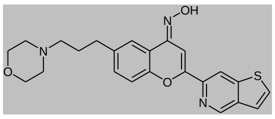	Therapeutic strategies for Parkinson disease: beyond dopaminergic drugs. Charvin, D; Medori, R; Hauser, RA; Rascol, O. NATURE REVIEWS DRUG DISCOVERY, 17, 11, 804-822, DOI: 10.1038/nrd.2018.136, NOV 2018, **Document Type: Review**
Formononetin	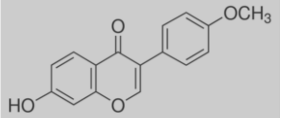	Flavonoids and its Neuroprotective Effects on Brain Ischemia and Neurodegenerative Diseases, Putteeraj, M; Lim, WL; Teoh, SL; Yahaya, MF, CURRENT DRUG TARGETS,19, 14, 1710-1720, DOI: 10.2174/1389450119666180326125252, 2018, **Document Type:Review**
Fractalkine		Immunomodulators as Therapeutic Agents in Mitigating the Progression of Parkinson's Disease, Grimmig, B; Morganti, J; Nash, K; Bickford, PC, BRAIN SCIENCES,6, 4, Article Number: 41, DOI: 10.3390/brainsci6040041, DEC 2016, **Document Type:Review**
Fraxetin	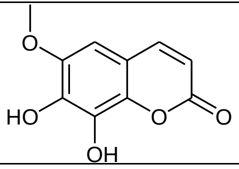	Recent advances in discovery and development of natural products as source for anti-Parkinson's disease lead compounds, Zhang, HJ; Bai, L; He, J; Zhong, L; Duan, XM; Ouyang, L; Zhu, YX; Wang, T; Shi, JY; Zhang, YW, EUROPEAN JOURNAL OF MEDICINAL CHEMISTRY,141, 257-272, DOI: 10.1016/j.ejmech.2017.09.068, DEC 1 2017, **Document Type:Review**
FTY720 derivatives	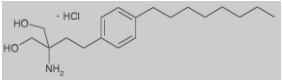	**1)** Up-regulation of protective neuronal MicroRNAs by FTY720 and novel FTY720-derivatives, Vargas-Medrano, J; Yang, B; Garza, NT; Segura-Ulate, I; Perez, RG, NEUROSCIENCE LETTERS,690, 178-180, DOI: 10.1016/j.neulet.2018.10.040, JAN 18 2019, **2)** FTY720-derivatives do not induce FTY720-like lymphopenia, Segura-Ulate, I; Belcher, TK; Vidal-Martinez, G; Vargas-Medrano, J; Perez, RG, JOURNAL OF PHARMACOLOGICAL SCIENCES,133, 3, 187-189, DOI: 10.1016/j.jphs.2017.02.006, MAR 2017, **3)** Preclinical Metabolism, Pharmacokineticsand In Vivo Analysis of New Blood-Brain-Barrier Penetrant Fingolimod Analogues: FTY720-C2 and FTY720-Mitoxy, Enoru, JO; Yang, B; Krishnamachari, S; Villanueva, E; DeMaio, W; Watanyar, A; Chinnasamy, R; Arterburn, JB; Perez, RG, PLOS ONE,11, 9, Article Number: e0162162, DOI: 10.1371/journal.pone.0162162, SEP 9 2016
GAD		Advances in non-dopaminergic treatments for Parkinson's disease. Stayte, S; Vissel, B ; FRONTIERS IN NEUROSCIENCE, 8, Article Number: 113, DOI: 10.3389/fnins.2014.00113, MAY 22 2014
Galangin	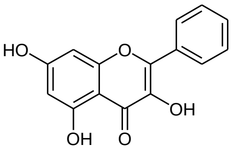	**1)** Anti-neuroinflammatory effects of galangin in LPS-stimulated BV-2 microglia through regulation of IL-1 production and the NF-B signaling pathways, Kim, ME; Park, PR; Na, JY; Jung, I; Cho, JH; Lee, JS, MOLECULAR AND CELLULAR BIOCHEMISTRY,451, 1-2, 145-153, DOI: 10.1007/s11010-018-3401-1, JAN 2019, **2)** Flavonoids and its Neuroprotective Effects on Brain Ischemia and Neurodegenerative Diseases, Putteeraj, M; Lim, WL; Teoh, SL; Yahaya, MF, CURRENT DRUG TARGETS,19, 14, 1710-1720, DOI: 10.2174/1389450119666180326125252, 2018, **Document Type:Review**
Gallic acid and derivatives	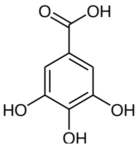	**1)** Gallic Acid Protects 6-OHDA Induced Neurotoxicity by Attenuating Oxidative Stress in Human Dopaminergic Cell Line, Chandrasekhar, Y; Kumar, GP; Ramya, EM; Anilakumar, KR, NEUROCHEMICAL RESEARCH,43, 6, 1150-1160, DOI: 10.1007/s11064-018-2530-y, JUN 2018, **2)** Molecular recognition between potential natural inhibitors of the Keap1-Nrf2 complex, Bello, M; Morales-Gonzalez, JA, INTERNATIONAL JOURNAL OF BIOLOGICAL MACROMOLECULES,105, 981-992 Part: 1, DOI: 10.1016/j.ijbiomac.2017.07.117, DEC 2017, **3)** Structure activity relationship of phenolic acid inhibitors of alpha-synuclein fibril formation and toxicity, Ardah, MT; Paleologou, KE; Lv, GH; Khair, SBA; Kazim, AS; Minhas, ST; Al-Tel, TH; Al-Hayani, AA; Haque, ME; Eliezer, D et al, FRONTIERS IN AGING NEUROSCIENCE,6, Article Number: 197, DOI: 10.3389/fnagi.2014.00197, AUG 5 2014. **4) et cetera**
Gallocatechin	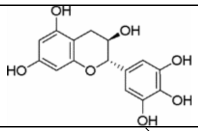	Flavonoids and its Neuroprotective Effects on Brain Ischemia and Neurodegenerative Diseases, Putteeraj, M; Lim, WL; Teoh, SL; Yahaya, MF, CURRENT DRUG TARGETS,19, 14, 1710-1720, DOI: 10.2174/1389450119666180326125252, 2018, **Document Type:Review**
Garcinol	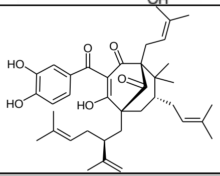	Garcinol prevents hyperhomocysteinemia and enhances bioavailability of L-DOPA by inhibiting catechol-O-methyltransferase: an in-silico approach, Mazumder, MK; Bhattacharjee, N; Borah, A, MEDICINAL CHEMISTRY RESEARCH,25, 1, 116-122, DOI: 10.1007/s00044-015-1472-z, JAN 2016
Gastrodin	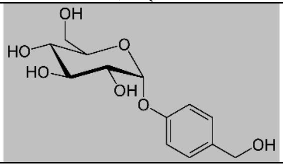	Gastrodin and Isorhynchophylline Synergistically Inhibit MPP+-Induced Oxidative Stress in SH-SY5Y Cells by Targeting ERK1/2 and GSK-3 beta Pathways: Involvement of Nrf2 Nuclear Translocation, Li, Q; Niu, CG; Zhang, XJ; Dong, MX, ACS CHEMICAL NEUROSCIENCE,9, 3, 482-493, DOI: 10.1021/acschemneuro.7b00247, MAR 2018

Genipin	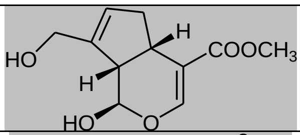	Application of Monoterpenoids and their Derivatives for Treatment of Neurodegenerative Disorders, Volcho, KP; Laev, SS; Ashraf, GM; Aliev, G; Salakhutdinov, NF, CURRENT MEDICINAL CHEMISTRY,25, 39, 5327-5346, DOI: 10.2174/0929867324666170112101837, 2018, **Document Type:Review**
Geniposide	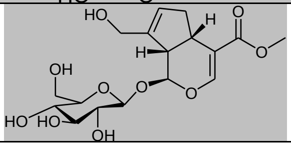	Application of Monoterpenoids and their Derivatives for Treatment of Neurodegenerative Disorders, Volcho, KP; Laev, SS; Ashraf, GM; Aliev, G; Salakhutdinov, NF, CURRENT MEDICINAL CHEMISTRY,25, 39, 5327-5346, DOI: 10.2174/0929867324666170112101837, 2018, **Document Type:Review**
Genistein	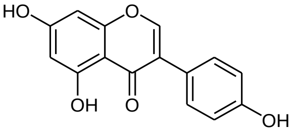	**1)** Neuroprotective effects of genistein on SH-SY5Y cells overexpressing A53T mutant alpha-synuclein, Wu, HC; Hu, QL; Zhang, SJ; Wang, YM; Jin, ZK; Lv, LF; Zhang, S; Liu, ZL; Wu, HL; Cheng, OM, NEURAL REGENERATION RESEARCH,13, 8, 1375-1383, DOI: 10.4103/1673-5374.235250, AUG 2018, **2)** Flavonoids and its Neuroprotective Effects on Brain Ischemia and Neurodegenerative Diseases, Putteeraj, M; Lim, WL; Teoh, SL; Yahaya, MF, CURRENT DRUG TARGETS,19, 14, 1710-1720, DOI: 10.2174/1389450119666180326125252, 2018, **Document Type:Review**, **3)** Recent advances in discovery and development of natural products as source for anti-Parkinson's disease lead compounds, Zhang, HJ; Bai, L; He, J; Zhong, L; Duan, XM; Ouyang, L; Zhu, YX; Wang, T; Shi, JY; Zhang, YW, EUROPEAN JOURNAL OF MEDICINAL CHEMISTRY,141, 257-272, DOI: 10.1016/j.ejmech.2017.09.068, DEC 1 2017, **Document Type:Review, 4)** Evaluation of the Isoflavone Genistein as Reversible Human Monoamine Oxidase-A and -B Inhibitor, Zarmouh, NO; Messeha, SS; Elshami, F; Soliman, KFA, EVIDENCE-BASED COMPLEMENTARY AND ALTERNATIVE MEDICINE, Article Number: 1423052, DOI: 10.1155/2016/1423052, 2016
Geranium aceto nitrile (Acetonyl geranine)	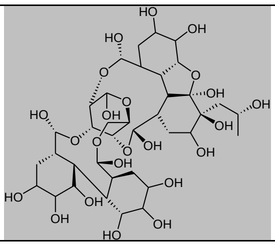	Molecular recognition between potential natural inhibitors of the Keap1-Nrf2 complex, Bello, M; Morales-Gonzalez, JA, INTERNATIONAL JOURNAL OF BIOLOGICAL MACROMOLECULES,105, 981-992 Part: 1, DOI: 10.1016/j.ijbiomac.2017.07.117, DEC 2017
Geranylgeranylacetone	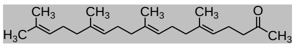	Pretreatment of glial cell-derived neurotrophic factor and geranylgeranylacetone ameliorates brain injury in Parkinson's disease by its anti-apoptotic and anti-oxidative property, Yue, PJ; Gao, L; Wang, XJ; Ding, XB; Teng, JF, JOURNAL OF CELLULAR BIOCHEMISTRY,119, 7, 5491-5502, DOI: 10.1002/jcb.26712, JUL 2018
GIF-0726-r	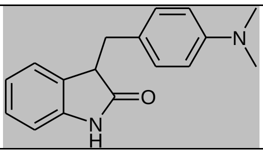	Novel oxindole derivatives prevent oxidative stress-induced cell death in mouse hippocampal HT22 cells, Hirata, Y; Yamada, C; Ito, Y; Yamamoto, S; Nagase, H; Oh-hashi, K; Kiuchi, K; Suzuki, H; Sawada, M; Furuta, K, NEUROPHARMACOLOGY,135, 242-252, DOI: 10.1016/j.neuropharm.2018.03.015, JUN 2018
Ginsenoside Rb1	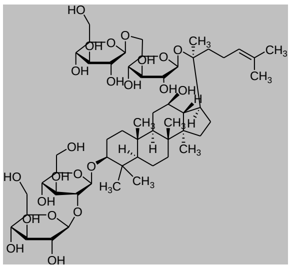	**1)** Ginsenoside Rb1 confers neuroprotection via promotion of glutamate transporters in a mouse model of Parkinson's disease, Zhang, YL; Liu, Y; Kang, XP; Dou, CY; Zhuo, RG; Huang, SQ; Peng, L; Wen, L, NEUROPHARMACOLOGY,131, 223-237, DOI: 10.1016/j.neuropharm.2017.12.012, MAR 15 2018, **2)** Ginsenosides: A Potential Neuroprotective Agent, Zheng, MM; Xin, YZ; Li, YJ; Xu, FX; Xi, XZ; Guo, H; Cui, XW; Cao, H; Zhang, X; Han, CC, BIOMED RESEARCH INTERNATIONAL, Article Number: 8174345, DOI: 10.1155/2018/8174345, 2018, **Document Type:Review**, **3)** Ginsenoside Rb1 inhibits fibrillation and toxicity of alpha-synuclein and disaggregates preformed fibrils, Ardah, MT; Paleologou, KE; Lv, GH; Menon, SA; Abul Khair, SB; Lu, JH; Safieh-Garabedian, B; Al-Hayani, AA; Eliezer, D; Li, M et al, NEUROBIOLOGY OF DISEASE,74, 89-101, DOI: 10.1016/j.nbd.2014.11.007, FEB 2015, **3) et cetera**
Ginsenoside Rd, Re, Rg1	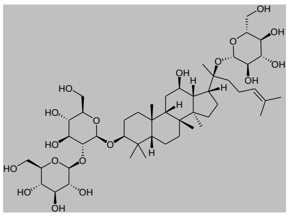	**1)** Connection between Systemic Inflammation and Neuroinflammation Underlies Neuroprotective Mechanism of Several Phytochemicals in Neurodegenerative Diseases, Wang, JT; Song, YT; Chen, Z; Leng, SAX, OXIDATIVE MEDICINE AND CELLULAR LONGEVITY, Article Number: 1972714, DOI: 10.1155/2018/1972714, 2018, **Document Type: Review, 2)** Recent advances in discovery and development of natural products as source for anti-Parkinson's disease lead compounds, Zhang, HJ; Bai, L; He, J; Zhong, L; Duan, XM; Ouyang, L; Zhu, YX; Wang, T; Shi, JY; Zhang, YW, EUROPEAN JOURNAL OF MEDICINAL CHEMISTRY,141, 257-272, DOI: 10.1016/j.ejmech.2017.09.068, DEC 1 2017, **Document Type:Review**

	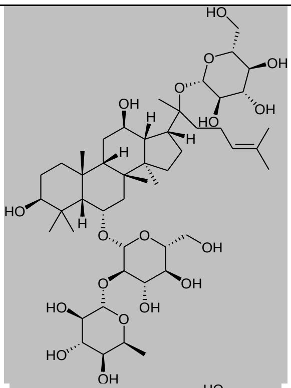<br>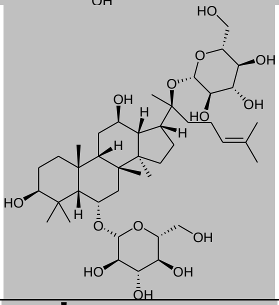	
GinkgolideB	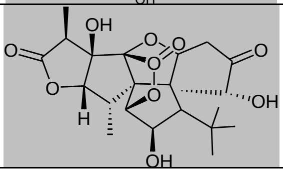	Recent advances in discovery and development of natural products as source for anti-Parkinson's disease lead compounds, Zhang, HJ; Bai, L; He, J; Zhong, L; Duan, XM; Ouyang, L; Zhu, YX; Wang, T; Shi, JY; Zhang, YW, EUROPEAN JOURNAL OF MEDICINAL CHEMISTRY,141, 257-272, DOI: 10.1016/j.ejmech.2017.09.068, DEC 1 2017, **Document Type:Review**
Gintonin		**1)** Gintonin Mitigates MPTP-Induced Loss of Nigrostriatal Dopaminergic Neurons and Accumulation of -Synuclein via the Nrf2/HO-1 Pathway, Jo, MG; Ikram, M; Jo, MH; Yoo, L; Chung, KC; Nah, SY; Hwang, H; Rhim, H; Kim, MO, MOLECULAR NEUROBIOLOGY,56, 1, 39-55, DOI: 10.1007/s12035-018-1020-1, JAN 2019, **2)** Multi-Target Protective Effects of Gintonin in 1-Methyl-4-phenyl-1,2,3,6-tetrahydropyridine-Mediated Model of Parkinson's Disease via Lysophosphatidic Acid Receptors, Choi, JH; Jang, M; Oh, S; Nah, SY; Cho, IH, FRONTIERS IN PHARMACOLOGY,9, Article Number: 515, DOI: 10.3389/fphar.2018.00515, MAY 23 2018
GKT137831	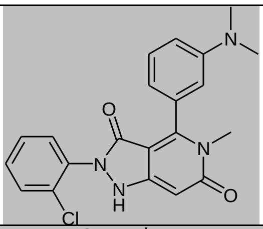	Redox-based therapeutics in neurodegenerative disease. McBean, GJ; Lopez, MG; Wallner, FK. BRITISH JOURNAL OF PHARMACOLOGY, 174, 12, 1750-1770, DOI: 10.1111/bph.13551, JUN 2017, **Document Type: Review**
GKT136901	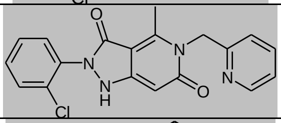	Redox-based therapeutics in neurodegenerative disease. McBean, GJ; Lopez, MG; Wallner, FK. BRITISH JOURNAL OF PHARMACOLOGY, 174, 12, 1750-1770, DOI: 10.1111/bph.13551, JUN 2017, **Document Type: Review**
Glaucocalyxin B	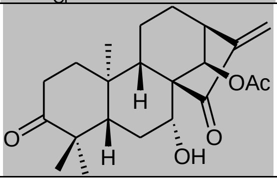	Anti-inflammatory effects of glaucocalyxin B in microglia cells, Gan, P; Zhang, L; Chen, YK; Zhang, Y; Zhang, FL; Zhou, X; Zhang, XH; Gao, B; Zhen, XC; Zhang, J et al, JOURNAL OF PHARMACOLOGICAL SCIENCES,128, 1, 35-46, DOI: 10.1016/j.jphs.2015.04.005, MAY 2015
Glial cell-derived neurotrophic factor		Pretreatment of glial cell-derived neurotrophic factor and geranylgeranylacetone ameliorates brain injury in Parkinson's disease by its anti-apoptotic and anti-oxidative property, Yue, PJ; Gao, L; Wang, XJ; Ding, XB; Teng, JF, JOURNAL OF CELLULAR BIOCHEMISTRY,119, 7, 5491-5502, DOI: 10.1002/jcb.26712, JUL 2018
Glimepiride	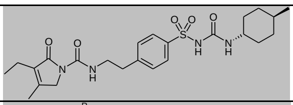	Glimepiride reduces CD14 expression and cytokine secretion from macrophages, Ingham, V; Williams, A; Bate, C, JOURNAL OF NEUROINFLAMMATION,11, Article Number: 115, DOI: 10.1186/1742-2094-11-115, JUN 21 2014
β-Glucosylceramide	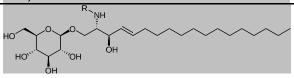	Combined beta-glucosylceramide and ambroxol hydrochloride in patients with Gaucher related Parkinson disease: From clinical observations to drug development, Ishay, Y; Zimran, A; Szer, J; Dinur, T; Ilana, Y; Arkadir, D; BLOOD CELLS MOLECULES AND DISEASES, 68, 117-120 Special : SI, DOI: 10.1016/j.bcmd.2016.10.028, FEB 2018

Glutamine	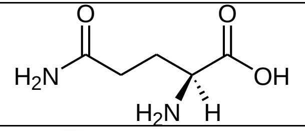	Glutamine promotes Hsp70 and inhibits alpha-Synuclein accumulation in pheochromocytoma PC12 cells, Wang, HY; Tang, CY; Jiang, ZF; Zhou, X; Chen, JH; Na, M; Shen, H; Lin, ZG, EXPERIMENTAL AND THERAPEUTIC MEDICINE,14, 2, 1253-1259, DOI: 10.3892/etm.2017.4580, AUG 2017
Glutathione derivatives	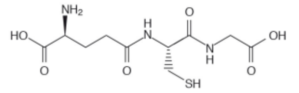	Application of Glutathione as Anti-Oxidative and Anti-Aging Drugs, Homma, T; Fujii, J, CURRENT DRUG METABOLISM,16, 7, 560-571, DOI: 10.2174/1389200216666151015114515, 2015
Glutathione - hydroxyquinoline compound	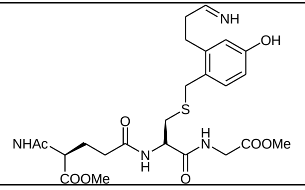	A Glutathione Derivative with Chelating and in vitro Neuroprotective Activities: Synthesis, Physicochemical Properties, and Biological Evaluation, Cacciatore, I; Cornacchia, C; Fornasari, E; Baldassarre, L; Pinnen, F; Sozio, P; Di Stefano, A; Marinelli, L; Dean, A; Fulle, S et al, CHEMMEDCHEM,8, 11, 1818-1829, DOI: 10.1002/cmdc.201300295, NOV 2013
Glutathione - L-DOPA compound	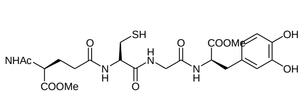	Chelating and antioxidant properties of L-Dopa containing tetrapeptide for the treatment of neurodegenerative diseases, Cacciatore, I; Marinelli, L; Di Stefano, A; Di Marco, V; Orlando, G; Gabriele, M; Gatta, DMP; Ferrone, A; Franceschelli, S; Speranza, L et al, NEUROPEPTIDES,71, 11-20, DOI: 10.1016/j.npep.2018.06.002, OCT 2018
Gly-N-C-DOPA	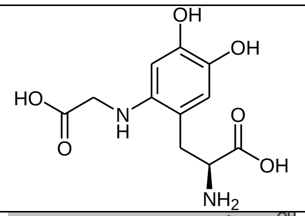	Tyrosinase mediated oxidative functionalization in the synthesis of DOPA-derived peptidomimetics with anti-Parkinson activity, Bizzarri, BM; Martini, A; Serafini, F; Aversa, D; Piccinino, D; Botta, L; Berretta, N; Guatteo, E; Saladino, R, RSC ADVANCES,7, 33, 20502-20509, DOI: 10.1039/c7ra03326e, 2017
Glycitein	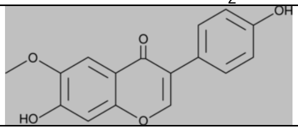	Flavonoids and its Neuroprotective Effects on Brain Ischemia and Neurodegenerative Diseases, Putteeraj, M; Lim, WL; Teoh, SL; Yahaya, MF, CURRENT DRUG TARGETS,19, 14, 1710-1720, DOI: 10.2174/1389450119666180326125252, 2018, **Document Type:Review**
Glycoprotein nonmetastatic melanoma protein B		Glycoprotein NMB: an Emerging Role in Neurodegenerative Disease, Budge, KM; Neal, ML; Richardson, JR; Safadi, FF, MOLECULAR NEUROBIOLOGY,55, 6, 5167-5176, DOI: 10.1007/s12035-017-0707-z, JUN 2018, **Document Type: Review**
Glycyrrhizic acid	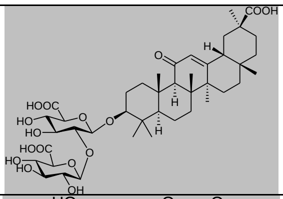	Glycyrrhizic Acid Alleviates 6-Hydroxydopamine and Corticosterone-Induced Neurotoxicity in SH-SY5Y Cells Through Modulating Autophagy, Yang, GY; Li, J; Cai, YL; Yang, ZH; Li, R; Fu, WJ, NEUROCHEMICAL RESEARCH,43, 10, 1914-1926, DOI: 10.1007/s11064-018-2609-5, OCT 2018
Glycyrurol	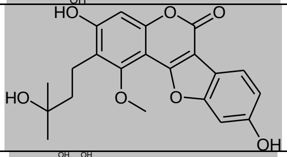	Identification of Licopyranocoumarin and Glycyrurol from Herbal Medicines as Neuroprotective Compounds for Parkinson's Disease, Fujimaki, T; Saiki, S; Tashiro, E; Yamada, D; Kitagawa, M; Hattori, N; Imoto, M, PLOS ONE,9, 6, Article Number: e100395, DOI: 10.1371/journal.pone.0100395, JUN 24 2014
GM1	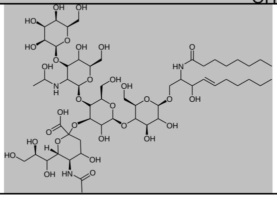	The ongoing pursuit of neuroprotective therapies in Parkinson disease, Athauda, D; Foltynie, T, NATURE REVIEWS NEUROLOGY,11, 1, 25-40, DOI: 10.1038/nrneurol.2014.226, JAN 2015, **Document Type: Review**
GNDF		Advances in non-dopaminergic treatments for Parkinson's disease. Stayte, S; Vissel, B ; FRONTIERS IN NEUROSCIENCE, 8, Article Number: 113, DOI: 10.3389/fnins.2014.00113, MAY 22 2014
GSK2795039	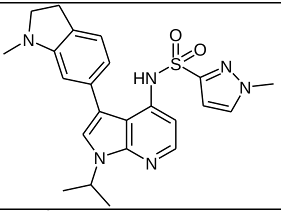	Redox-based therapeutics in neurodegenerative disease. McBean, GJ; Lopez, MG; Wallner, FK. BRITISH JOURNAL OF PHARMACOLOGY, 174, 12, 1750-1770, DOI: 10.1111/bph.13551, JUN 2017, **Document Type: Review**
Guanabenz	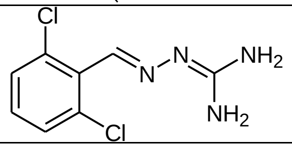	Guanabenz promotes neuronal survival&IT via&IT enhancement of ATF4 and parkin expression in models of Parkinson disease, Sun, XT; Aime, P; Dai, D; Ramalingam, N; Crary, JF; Burke, RE; Greene, LA; Levy, OA, EXPERIMENTAL NEUROLOGY,303, 95-107, DOI: 10.1016/j.expneurol.2018.01.015, MAY 2018

Guanosine	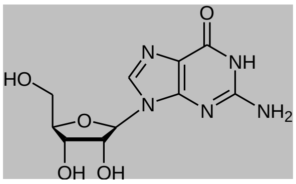	**1)** Guanosine Protects Striatal Slices Against 6-OHDA-Induced Oxidative Damage, Mitochondrial Dysfunction, and ATP Depletion, Marques, NF; Massari, CM; Tasca, CI, NEUROTOXICITY RESEARCH,35, 2, 475-483, DOI: 10.1007/s12640-018-9976-1, FEB 2019, **2)** Antiparkinsonian Efficacy of Guanosine in Rodent Models of Movement Disorder, Massari, CM; Lopez-Cano, M; Nunez, F; Fernandez-Duenas, V; Tasca, CI; Ciruela, F, FRONTIERS IN PHARMACOLOGY,8, Article Number: 700, DOI: 10.3389/fphar.2017.00700, OCT 4 2017, **3) et cetera**
GW8510	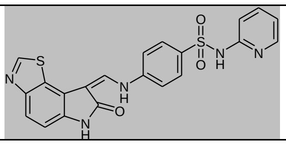	Gene Expression-Based Screen for Parkinson's Disease Identifies GW8510 as a Neuroprotective Agent, Wimalasena, NK; Le, VQ; Wimalasena, K; Schreiber, SL; Karmacharya, R, ACS CHEMICAL NEUROSCIENCE,7, 7, 857-863, DOI: 10.1021/acschemneuro.6b00076, JUL 2016
Gypenosides		Gypenosides ameliorate memory deficits in MPTP-lesioned mouse model of Parkinson's disease treated with L-DOPA, Zhao, TT; Kim, KS; Shin, KS; Park, HJ; Kim, HJ; Lee, KE; Lee, MK, BMC COMPLEMENTARY AND ALTERNATIVE MEDICINE,17, Article Number: 449, DOI: 10.1186/s12906-017-1959-x, SEP 6 2017
GZ667161	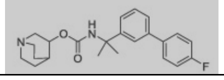	Current approaches to the treatment of Parkinson's Disease, Ellis, JM; Fell, MJ, BIOORGANIC & MEDICINAL CHEMISTRY LETTERS, 27, 18, 4247-4255, DOI: 10.1016/j.bmcl.2017.07.075, Published: SEP 15 2017
Hc-Tetanus Toxin fragment		Peripheral Administration of Tetanus Toxin Hc Fragment Prevents MPP+ Toxicity In Vivo, Moreno-Galarza, N; Mendieta, L; Palafox-Sanchez, V; Herrando-Grabulosa, M; Gil, C; Limon, DI; Aguilera, J, NEUROTOXICITY RESEARCH,34, 1, 47-61, DOI: 10.1007/s12640-017-9853-3, JUL 2018
Hemantane	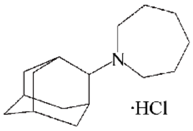	Anti-Inflammatory Activity of Hemantane on Peripheral Inflammation and Lipopolysaccharideinduced Neuro-Inflammation Models, Ivanova, EA; Kapitsa, IG; Nepoklonov, AV; Kokshenev, II; Val'dman, EA; Voronina, TA, PHARMACEUTICAL CHEMISTRY JOURNAL,47, 10, 517-520, DOI: 10.1007/s11094-014-0994-x, JAN 2014
Hesperidin	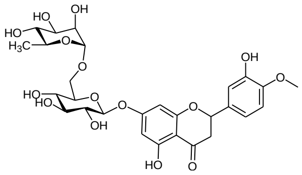	**1)** Hesperidin as a Neuroprotective Agent: A Review of Animal and Clinical Evidence, Hajialyani, M; Farzaei, MH; Echeverria, J; Nabavi, SM; Uriarte, E; Sobarzo-Sanchez, E, MOLECULES,24, 3, Article Number: 648, DOI: 10.3390/molecules24030648, FEB 1 2019, **Document Type: Review, 2)** Flavonoids and its Neuroprotective Effects on Brain Ischemia and Neurodegenerative Diseases, Putteeraj, M; Lim, WL; Teoh, SL; Yahaya, MF, CURRENT DRUG TARGETS,19, 14, 1710-1720, DOI: 10.2174/1389450119666180326125252, 2018, **Document Type:Review**
Hinokitiol	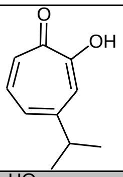	Hinokitiol Offers Neuroprotection Against 6-OHDA-Induced Toxicity in SH-SY5Y Neuroblastoma Cells by Downregulating mRNA Expression of MAO/alpha-Synuclein/LRRK2/PARK7/PINK1/PTEN Genes., Varier, Krishnapriya Madhu; Sumathi, Thangarajan, Neurotoxicity research, DOI:10.1007/s12640-018-9988-x, 2018-Dec-19
Honokiol	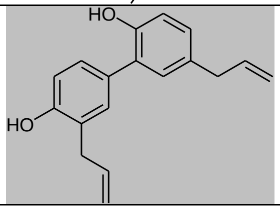	**1)** Protective and therapeutic activity of honokiol in reversing motor deficits and neuronal degeneration in the mouse model of Parkinson's disease, Chen, HH; Chang, PC; Chen, CP; Chan, MH, PHARMACOLOGICAL REPORTS,70, 4, 668-676, DOI: 10.1016/j.pharep.2018.01.003, AUG 2018, **2)** Exploring the Structural Diversity in Inhibitors of alpha-Synuclein Amyloidogenic Folding, Aggregation, and Neurotoxicity, Das, S; Pukala, TL; Smid, SD, FRONTIERS IN CHEMISTRY,6, Article Number: 181, DOI: 10.3389/fchem.2018.00181, MAY 25 2018
8-HQ-MC-5 (VK-28)	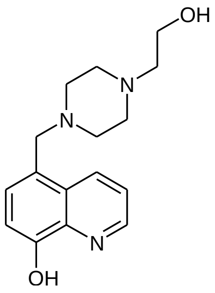	**1)** Development of Multifunctional Molecules as Potential Therapeutic Candidates for Alzheimer's Disease, Parkinson's Disease, and Amyotrophic Lateral Sclerosis in the Last Decade. Savelieff, MG; Nam, G; Kang, J; Lee, HJ; Lee, M; Lim, MH. CHEMICAL REVIEWS, 119, 2, 1221-1322, DOI: 10.1021/acs.chemrev.8b00138, JAN 23 2019, **Document Type: Review, 2)** A review on iron chelators as potential therapeutic agents for the treatment of Alzheimer's and Parkinson's diseases. Singh, YP; Pandey, A; Vishwakarma, S; Modi, G, Molecular diversity, DOI:10.1007/s11030-018-9878-4, 2018-Oct-06,. **Document type: review, 3)** Iron Chelating Strategies in Systemic Metal Overload, Neurodegeneration and Cancer. Gumienna-Kontecka, E; Pyrkosz-Bulska, M; Szebesczyk, A; Ostrowska, M. CURRENT MEDICINAL CHEMISTRY, 21, 33, 3741-3767, DOI: 10.2174/0929867321666140706143402, 2014. **Document Type: Review, 4)** Different 8-hydroxyquinolines protect models of tdp-43 protein, alpha-synuclein, and polyglutamine proteotoxicity through distinct mechanisms. Tardiff, D.F., Tucci, M.L., Caldwell, K.A., Caldwell, G.A., Lindquist, S. J. Biol. Chem. 287, 4107-4120, 2012. **5)** Neuroprotection by a novel brain permeable iron chelator, vk-28, against 6-hydroxydopamine lession in rats. Shachar, D.B., Kahana, N., Kampel, V., Warshawsky, A., Youdim, M.B.H. Neuropharmacology, 46, 254-263, 2004
HSP70		New HSF1 inducer as a therapeutic agent in a rodent model of Parkinson's disease, Ekimova, IV; Plaksina, DV; Pastukhov, YF; Lapshina, KV; Lazarev, VF; Mikhaylova, ER; Polonik, SG; Pani, B; Margulis, BA; Guzhova, IV et al, EXPERIMENTAL NEUROLOGY,306, 199-208, DOI: 10.1016/j.expneurol.2018.04.012, AUG 2018

HU-210	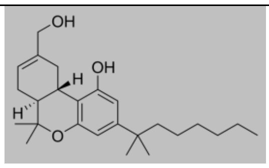	**1)** The role of cannabinoids and leptin in neurological diseases, Agar, E; ACTA NEUROLOGICA SCANDINAVICA, 132, 6, 371-380, DOI: 10.1111/ane.12411, DEC 2015, **Document Type:Review, 2)** Promising cannabinoid-based therapies for Parkinson's disease: motor symptoms to neuroprotection, More, SV; Choi, DK; MOLECULAR NEURODEGENERATION, 10, Article Number: 17, DOI: 10.1186/s13024-015-0012-0, APR 8 2015, **Document Type:Review, 3) et cetera**
[4,4'-(1E, 1'E, 3E, 3'E)-3,3'-(Hydrazine-1,2-diylidene) bis-(prop-1-ene-1-yl-3-ylidene) bis-(2-methoxyphenol)]	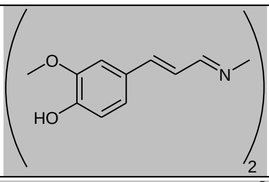	N,N-disubstituted azines attenuate LPS-mediated neuroinflammation in microglia and neuronal apoptosis via inhibiting MAPK signaling pathways, Subedi, L; Kwon, OW; Pak, C; Lee, G; Lee, K; Kim, H; Kim, SY, BMC NEUROSCIENCE,18, Article Number: 82, DOI: 10.1186/s12868-017-0399-3, DEC 28 2017
Cis-N-Hydroxycinnamoyl-7'-methoxyethyltyramine	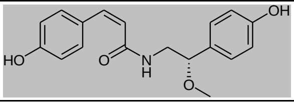	Phenolic amides with anti-Parkinson's disease effects from Nicandra physaloides, Wang, LQ; Wang, Y; Gao, SY; Zhu, LH; Wang, F; Li, H; Chen, LX, JOURNAL OF FUNCTIONAL FOODS,31, 229-236, DOI: 10.1016/j.jff.2017.01.045, APR 2017
(7R,8S)-7-(4-Hydroxy-3,5-dimethoxyphenyl)-8-hydroxy methyl-V-[N-7''-(4''-hydrxyphenyflethyl]carbamoyletheny1-3'-methoxybenzodihydrofuran	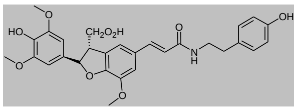	Phenolic amides with anti-Parkinson's disease effects from Nicandra physaloides, Wang, LQ; Wang, Y; Gao, SY; Zhu, LH; Wang, F; Li, H; Chen, LX, JOURNAL OF FUNCTIONAL FOODS,31, 229-236, DOI: 10.1016/j.jff.2017.01.045, APR 2017
4-Hydroxyisophthalic acid	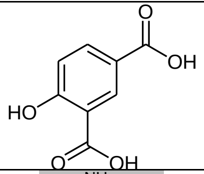	Neuroprotective action of 4-Hydroxyisophthalic acid against paraquat-induced motor impairment involves amelioration of mitochondrial damage and neurodegeneration in Drosophila, Niveditha, S; Shivanandappa, T, NEUROTOXICOLOGY,66, 160-169, DOI: 10.1016/j.neuro.2018.04.006, MAY 2018
3-Hydroxymorphinan	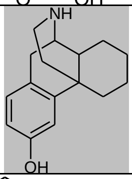	Design, Synthesis, and Biological Evaluation of Phenol Bioisosteric Analogues of 3-Hydroxymorphinan, Li, ZQ; Bao, XQ; Bai, XG; Zhang, GN; Wang, JX; Zhu, M; Wang, Y; Shang, JM; Sheng, CJ; Zhang, D et al, SCIENTIFIC REPORTS,9, Article Number: 2247, DOI: 10.1038/s41598-019-38911-1, FEB 19 2019
1-Hydroxy-2-pyridinone derivatives	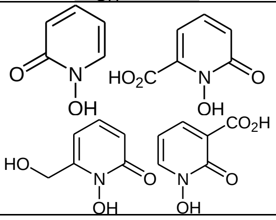	**1)** A review on iron chelators as potential therapeutic agents for the treatment of Alzheimer's and Parkinson's diseases. Singh, YP; Pandey, A; Vishwakarma, S; Modi, G, Molecular diversity, DOI:10.1007/s11030-018-9878-4, 2018-Oct-06 (Epub 2018 Oct 06). **Document type: review, 2)** Protection from neurodegeneration in the 6-hydroxydopamine model of Parkinson's with novel 1-hydroxypyridin-2-one metal chelators, Workman, DG; Tsatsanis, A; Lewis, FW; Boyle, JP; Mousadoust, M; Hettiarachchi, NT; Hunter, M; Peers, CS; Tetard, D; Duce, JA, METALLOMICS,7, 5, 867-876, DOI: 10.1039/c4mt00326h, 2015
3-Hydroxy-4(1H)-pyridinone (deferiprone)	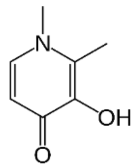	**1)** Protection from neurodegeneration in the 6-hydroxydopamine model of Parkinson's with novel 1-hydroxypyridin-2-one metal chelators, Workman, DG; Tsatsanis, A; Lewis, FW; Boyle, JP; Mousadoust, M; Hettiarachchi, NT; Hunter, M; Peers, CS; Tetard, D; Duce, JA, METALLOMICS,7, 5, 867-876, DOI: 10.1039/c4mt00326h, 2015, **2)** The ongoing pursuit of neuroprotective therapies in Parkinson disease, Athauda, D; Foltynie, T, NATURE REVIEWS NEUROLOGY,11, 1, 25-40, DOI: 10.1038/nrneurol.2014.226, JAN 2015, **Document Type:Review**, **3)** Iron Chelators and Antioxidants Regenerate Neuritic Tree and Nigrostriatal Fibers of MPP plus /MPTP-Lesioned Dopaminergic Neurons, Aguirre, P; Mena, NP; Carrasco, CM; Munoz, Y; Perez-Henriquez, P; Morales, RA; Cassels, BK; Mendez-Galvez, C; Garcia-Beltran, O; Gonzalez-Billault, C et al, PLOS ONE,10, 12, Article Number: e0144848, DOI: 10.1371/journal.pone.0144848, DEC 14 2015
3-Hydroxy-4(1H)-pyridinone derivatives	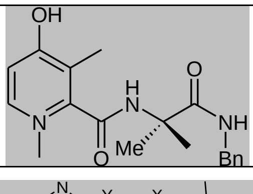	A review on iron chelators as potential therapeutic agents for the treatment of Alzheimer's and Parkinson's diseases. Singh, YP; Pandey, A; Vishwakarma, S; Modi, G, Molecular diversity, DOI:10.1007/s11030-018-9878-4, 2018-Oct-06. **Document type: review**
Hydroxypyridone derivatives (R= Bn)	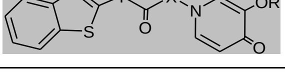	A review on iron chelators as potential therapeutic agents for the treatment of Alzheimer's and Parkinson's diseases. Singh, YP; Pandey, A; Vishwakarma, S; Modi, G, Molecular diversity, DOI:10.1007/s11030-018-9878-4, 2018-Oct-06. **Document type: review**
3-Hydroxy-4(1H)-pyridinone derivatives (R = H)	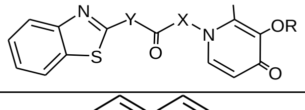	A review on iron chelators as potential therapeutic agents for the treatment of Alzheimer's and Parkinson's diseases. Singh, YP; Pandey, A; Vishwakarma, S; Modi, G, Molecular diversity, DOI:10.1007/s11030-018-9878-4, 2018-Oct-06. **Document type: review**
8-Hydroxyquinoline	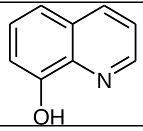	Cyclodextrins 3-Functionalized with 8-Hydroxyquinolines: Copper-Binding Ability and Inhibition of Synuclein Aggregation, Oliveri, V; Sgarlata, C; Vecchio, G, CHEMISTRY-AN ASIAN JOURNAL,11, 17, 2436-2442, DOI: 10.1002/asia.201600824, SEP 6 2016

8-Hydroxyquinoline-2-carboxaldehyde isonicotinoyl hydrazone	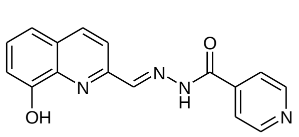	A moderate metal-binding hydrazone meets the criteria for a bioinorganic approach towards Parkinson's disease: Therapeutic potential, blood-brain barrier crossing evaluation and preliminary toxicological studies, Cukierman, DS; Pinheiro, AB; Castineiras, SLP; da Silva, ASP; Miotto, MC; De Falco, A; Ribeiro, TD; Maisonette, S; da Cunha, ALMC; Hauser-Davis, RA et al, JOURNAL OF INORGANIC BIOCHEMISTRY,170, 160-168, DOI: 10.1016/j.jinorgbio.2017.02.020, MAY 2017
Hydroxyquinoline-propargyl hybrid (HLA 20)	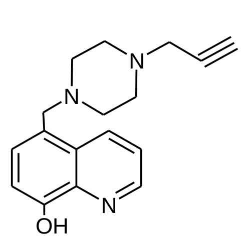	**1)** Development of Multifunctional Molecules as Potential Therapeutic Candidates for Alzheimer's Disease, Parkinson's Disease, and Amyotrophic Lateral Sclerosis in the Last Decade. Savelieff, MG; Nam, G; Kang, J; Lee, HJ; Lee, M; Lim, MH. CHEMICAL REVIEWS, 119, 2, 1221-1322, DOI: 10.1021/acs.chemrev.8b00138, JAN 23 2019**, Document Type: Review**, **2)** A review on iron chelators as potential therapeutic agents for the treatment of Alzheimer's and Parkinson's diseases. Singh, YP; Pandey, A; Vishwakarma, S; Modi, G, Molecular diversity, DOI:10.1007/s11030-018-9878-4, 2018-Oct-06.**Document type: review, 3)** New Perspectives in Iron Chelation Therapy for the Treatment of Neurodegenerative Diseases. Nunez, MT; Chana-Cuevas, P. PHARMACEUTICALS, 11, 4, Article Number: UNSP 109, DOI: 10.3390/ph11040109, DEC 2018, **Document Type: Review**
Hydroxysaffior yellow A	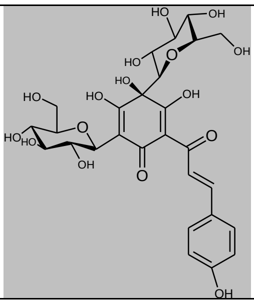	Coadministration of hydroxysafflor yellow A with levodopa attenuates the dyskinesia, Wang, T; Duan, SJ; Wang, SY; Lu, Y; Zhu, Q; Wang, LJ; Han, B, PHYSIOLOGY & BEHAVIOR,147, 193-197, DOI: 10.1016/j.physbeh.2015.04.038, AUG 1 2015
Hydroxytyrosol butyrate	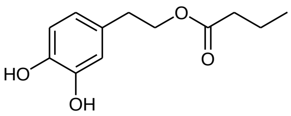	Hydroxytyrosol butyrate inhibits 6-OHDA-induced apoptosis through activation of the Nrf2/HO-1 axis in SH-SY5Y cells, Funakohi-Tago, M; Sakata, T; Fujiwara, S; Sakakura, A; Sugai, T; Tago, K; Tamura, H, EUROPEAN JOURNAL OF PHARMACOLOGY,834, 246-256, DOI: 10.1016/j.ejphar.2018.07.043, SEP 5 2018
Hyperoside	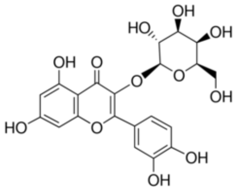	Recent advances in discovery and development of natural products as source for anti-Parkinson's disease lead compounds, Zhang, HJ; Bai, L; He, J; Zhong, L; Duan, XM; Ouyang, L; Zhu, YX; Wang, T; Shi, JY; Zhang, YW, EUROPEAN JOURNAL OF MEDICINAL CHEMISTRY,141, 257-272, DOI: 10.1016/j.ejmech.2017.09.068, DEC 1 2017, **Document Type:Review**
Hypoestoxide	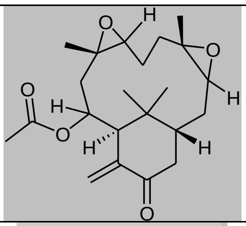	Hypoestoxide reduces neuroinflammation and alpha-synuclein accumulation in a mouse model of Parkinson's disease, Kim, C; Ojo-Amaize, E; Spencer, B; Rockenstein, E; Mante, M; Desplats, P; Wrasidlo, W; Adame, A; Nchekwube, E; Oyemade, O et al, JOURNAL OF NEUROINFLAMMATION,12, Article Number: 236, DOI: 10.1186/s12974-015-0455-9, DEC 18 2015
Ibiglustat	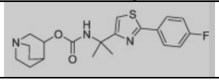	Current approaches to the treatment of Parkinson's Disease, Ellis, JM; Fell, MJ, BIOORGANIC & MEDICINAL CHEMISTRY LETTERS, 27, 18, 4247-4255, DOI: 10.1016/j.bmcl.2017.07.075, Published: SEP 15 2017
Ibuprofen	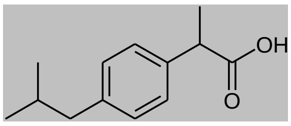	**1)** Stabilizing proteins to prevent conformational changes required for amyloid fibril formation, Siddiqi, MK; Alam, P; Malik, S; Majid, N; Chaturvedi, SK; Rajan, S; Ajmal, MR; Khan, MV; Uversky, VN; Khan, RH, JOURNAL OF CELLULAR BIOCHEMISTRY,120, 2, 2642-2656, DOI: 10.1002/jcb.27576, FEB 2019, **2)** Celecoxib, indomethacin and ibuprofen prevent 6-hydroxydopamine-induced PC12 cell death through the inhibition of NF kappa B and SAPK/JNK pathways, Ramazani, E; Tayarani-Najaran, Z; Fereidoni, M, IRANIAN JOURNAL OF BASIC MEDICAL SCIENCES,22, 5, 477-484, 2019, **3) et cetera**
IC87201	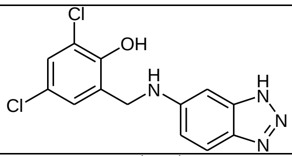	Small-molecule inhibitors at the PSD-95/nNOS interface attenuate MPP+-induced neuronal injury through Sirt3 mediated inhibition of mitochondrial dysfunction, Hu, W; Guan, LS; Dang, XB; Ren, PY; Zhang, YL, NEUROCHEMISTRY INTERNATIONAL,79, 57-64, DOI: 10.1016/j.neuint.2014.10.005, DEC 2014
Icariin	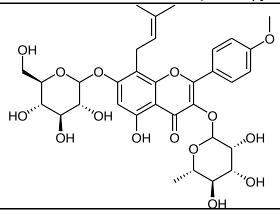	An outline for the pharmacological effect of icariin in the nervous system, Jin, J; Wang, H; Hua, XY; Chen, DJ; Huang, C; Chen, Z, EUROPEAN JOURNAL OF PHARMACOLOGY,842, 20-32, DOI: 10.1016/j.ejphar.2018.10.006, JAN 5 2019, **Document Type: Review**

Icariside II	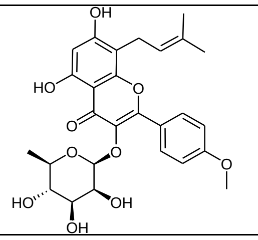	Icariside II attenuates lipopolysaccharide-induced neuroinflammation through inhibiting TLR4/MyD88/NF-kappa B pathway in rats, Zhou, JY; Deng, YY; Li, F; Yin, CX; Shi, JS; Gong, QH, BIOMEDICINE & PHARMACOTHERAPY,111, 315-324, DOI: 10.1016/j.biopha.2018.10.201, MAR 2019
Idazoxan	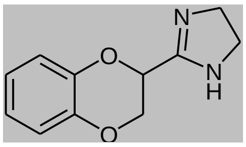	Treatment with the noradrenaline re-uptake inhibitor atomoxetine alone and in combination with the alpha 2-adrenoceptor antagonist idazoxan attenuates loss of dopamine and associated motor deficits in the LPS inflammatory rat model of Parkinson's disease, Yssel, JD; O'Neill, E; Nolan, YM; Connor, TJ; Harkin, A, BRAIN BEHAVIOR AND IMMUNITY,69, 456-469, DOI: 10.1016/j.bbi.2018.01.004, MAR 2018
Ifenprodil	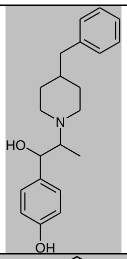	Advances in non-dopaminergic treatments for Parkinson's disease. Stayte, S; Vissel, B; FRONTIERS IN NEUROSCIENCE, 8, Article Number: 113, DOI: 10.3389/fnins.2014.00113, MAY 22 2014, **Document Type: Review**
Imatinib	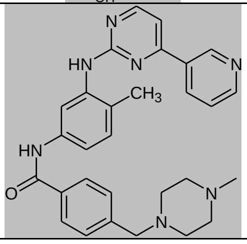	**1)** c-Abl Inhibition Exerts Symptomatic Antiparkinsonian Effects Through a Striatal Postsynaptic Mechanism, Zhou, Y; Yamamura, Y; Ogawa, M; Tsuji, R; Tsuchiya, K; Kasahara, J; Goto, S; FRONTIERS IN PHARMACOLOGY, 9, Article Number: 1311, DOI: 10.3389/fphar.2018.01311, NOV 16 2018, **2)** C-Abl Inhibition; A Novel Therapeutic Target for Parkinson's Disease, Abushouk, AI; Negida, A; Elshenawy, RA; Zein, H; Hammad, AM; Menshawy, A; Mohamed, WMY; CNS & NEUROLOGICAL DISORDERS-DRUG TARGETS, 17, 1, 14-21, DOI: 10.2174/1871527316666170602101538, 2018, **Document Type:Review**
l-(7-Imino-3-propyl-2,3-dihydrothiazolo [4, 5-d]pyrimidin-6(7H)-yl)urea	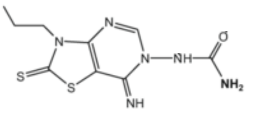	Neuroprotective effect of IDPU (l-(7-imino-3-propyl-2,3-dihydrothiazolo pyrimidin-6-yl)urea) in 6-OHDA induced rodent model of hemiparkinson's disease, Kumari, N; Agrawal, S; Kumari, R; Sharma, D; Luthra, PM, NEUROSCIENCE LETTERS,675, 74-82, DOI: 10.1016/j.neulet.2018.03.040, MAY 14 2018
Imipramine	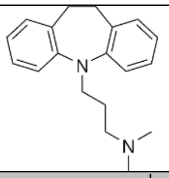	Imipramine and amitriptyline ameliorate the rotenone model of parkinson's disease in rats, Kandil, EA; Abdelkader, NF; El-Sayeh, BM; Saleh, S, NEUROSCIENCE,332, 26-37, DOI: 10.1016/j.neuroscience.2016.06.040, SEP 22 2016
Imperatorin	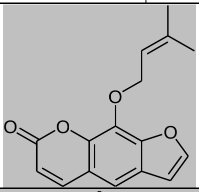	Pharmacokinetics and Brain Distribution of the Active Components of DA-9805, Saikosaponin A, Paeonol and Imperatorin in Rats, Kwon, MH; Jeong, JS; Ryu, J; Cho, YW; Kang, HE, PHARMACEUTICS,10, 3, Article Number: 133, DOI: 10.3390/pharmaceutics10030133, SEP 2018
2,4-bis(Indolin-3-one-2-ylimino)-6-phenyl-1,3,5-triazine metal complexes	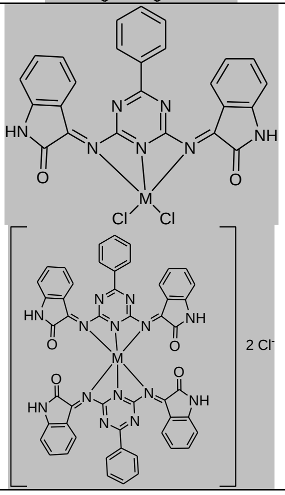	Transition metal complexes of s-triazine derivative: new class of anticonvulsant, anti-inflammatory, and neuroprotective agents, Shanmugakala, R; Tharmaraj, P; Sheela, CD; Chidambaranathan, N, MEDICINAL CHEMISTRY RESEARCH,23, 1, 329-342, DOI: 10.1007/s00044-013-0634-0, JAN 2014

Indoline-like LRRK2 inhibitors	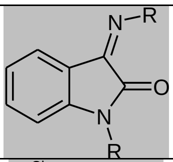	Leucine rich repeat kinase 2 inhibitors based on indolinone scaffold: Potential pro-neurogenic agents, Salado, IG; Zaldivar-Diez, J; Sebastian-Perez, V; Li, LL; Geiger, L; Gonzalez, S; Campillo, NE; Gil, C; Morales, AV; Perez, DI et al, EUROPEAN JOURNAL OF MEDICINAL CHEMISTRY,138, 328-342, DOI: 10.1016/j.ejmech.2017.06.060, SEP 29 2017
Indomethacin	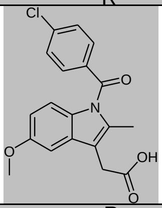	Celecoxib, indomethacin and ibuprofen prevent 6-hydroxydopamine-induced PC12 cell death through the inhibition of NF kappa B and SAPK/JNK pathways, Ramazani, E; Tayarani-Najaran, Z; Fereidoni, M, IRANIAN JOURNAL OF BASIC MEDICAL SCIENCES,22, 5, 477-484, 2019
bis(Indolyl)phenylmethane derivatives	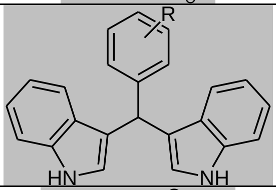	Bisphenylmethane derivatives are effective small molecules for inhibition of amyloid fibril formation by hen lysozyme, Ramshini, H; Mannini, B; Khodayari, K; Ebrahim-Habibi, A; Moghaddasi, AS; Tayebee, R; Chiti, F; EUROPEAN JOURNAL OF MEDICINAL CHEMISTRY, 124, 361-371, DOI: 10.1016/j.ejmech.2016.08.056, NOV 29 2016
Inosine	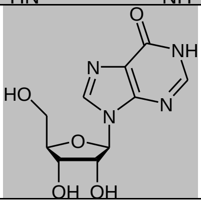	**1)** Disease-Modifying Drugs in Parkinson's Disease, Park, A; Stacy, M, DRUGS,75, 18, 2065-2071, DOI: 10.1007/s40265-015-0497-4, DEC 2015, **Document Type:Review**, **2)** The ongoing pursuit of neuroprotective therapies in Parkinson disease, Athauda, D; Foltynie, T, NATURE REVIEWS NEUROLOGY,11, 1, 25-40, DOI: 10.1038/nrneurol.2014.226, JAN 2015, **Document Type:Review**
Isatin (indol-2,3-dione)	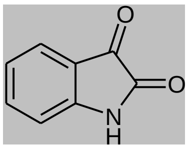	**1)** The effect of deprenyl and isatin administration to mice on the proteomic profile of liver isatin-binding proteins., Buneeva, O A; Kopylov, A T; Zgoda, V G; Medvedev, A E, Biomeditsinskaia khimiia, 64 :4 :354-359, DOI:10.18097/PBMC20186404354, 2018-Aug, **2)** A Recent Perspective on Discovery and Development of Diverse Therapeutic Agents Inspired from Isatin Alkaloids, Rane, RA; Karunanidhi, S; Jain, K; Shaikh, M; Hampannavar, G; Karpoormath, R, CURRENT TOPICS IN MEDICINAL CHEMISTRY,16, 11, 1262-1289, DOI: 10.2174/1568026615666150915112334, 2016, **Document Type: Review**
Isobavachalcone	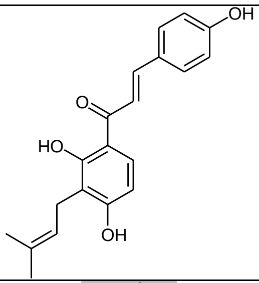	Isobavachalcone Attenuates MPTP-Induced Parkinson's Disease in Mice by Inhibition of Microglial Activation through NF-kappa B Pathway, Jing, HR; Wang, SX; Wang, M; Fu, WL; Zhang, C; Xu, DG, PLOS ONE,12, 1, Article Number: e0169560, DOI: 10.1371/journal.pone.0169560, JAN 6 2017
Isoborneol	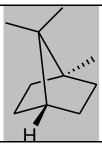	Recent advances in discovery and development of natural products as source for anti-Parkinson's disease lead compounds, Zhang, HJ; Bai, L; He, J; Zhong, L; Duan, XM; Ouyang, L; Zhu, YX; Wang, T; Shi, JY; Zhang, YW, EUROPEAN JOURNAL OF MEDICINAL CHEMISTRY,141, 257-272, DOI: 10.1016/j.ejmech.2017.09.068, DEC 1 2017, **Document Type:Review**
Isochlorogenic acid	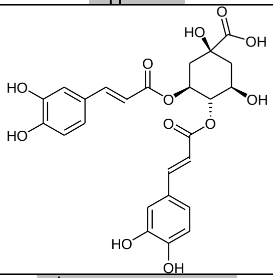	Acanthopanax senticosus Protects Structure and Function of Mesencephalic Mitochondria in A Mouse Model of Parkinson's Disease, Liu, SM; Li, XZ; Zhang, SN; Yang, ZM; Wang, KX; Lu, F; Wang, CZ; Yuan, CS; CHINESE JOURNAL OF INTEGRATIVE MEDICINE, 24, 11, 835-843, DOI: 10.1007/s11655-018-2935-5, NOV 2018
Isofraxidin	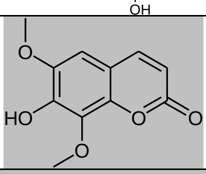	Acanthopanax senticosus Protects Structure and Function of Mesencephalic Mitochondria in A Mouse Model of Parkinson's Disease, Liu, SM; Li, XZ; Zhang, SN; Yang, ZM; Wang, KX; Lu, F; Wang, CZ; Yuan, CS; CHINESE JOURNAL OF INTEGRATIVE MEDICINE, 24, 11, 835-843, DOI: 10.1007/s11655-018-2935-5, NOV 2018
Isoliquiritigenin	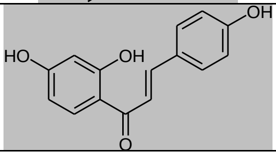	Phytochemical and Pharmacological Role of Liquiritigenin and Isoliquiritigenin From Radix Glycyrrhizae in Human Health and Disease Models, Ramalingam, M; Kim, H; Lee, Y; Lee, YI, FRONTIERS IN AGING NEUROSCIENCE,10, Article Number: 348, DOI: 10.3389/fnagi.2018.00348, NOV 1 2018, **Document Type: Review**

Isoquercetin (Isoquercitrin)	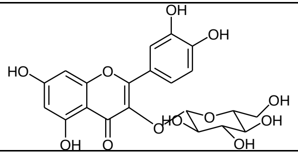	Protective effects of quercetin glycosides, rutin, and isoquercetrin against 6-hydroxydopamine -induced neurotoxicity in rat pheochromocytoma cells, Magalingam, KB; Radhakrishnan, A; Haleagrahara, N, INTERNATIONAL JOURNAL OF IMMUNOPATHOLOGY AND PHARMACOLOGY,29, 1, 30-39, DOI: 10.1177/0394632015613039, MAR 2016
Isorhynchophylline	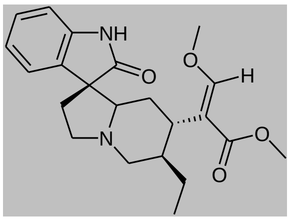	**1)** Gastrodin and Isorhynchophylline Synergistically Inhibit MPP+-Induced Oxidative Stress in SH-SY5Y Cells by Targeting ERK1/2 and GSK-3 beta Pathways: Involvement of Nrf2 Nuclear Translocation, Li, Q; Niu, CG; Zhang, XJ; Dong, MX, ACS CHEMICAL NEUROSCIENCE,9, 3, 482-493, DOI: 10.1021/acschemneuro.7b00247, MAR 2018, **2)** Recent advances in discovery and development of natural products as source for anti-Parkinson's disease lead compounds, Zhang, HJ; Bai, L; He, J; Zhong, L; Duan, XM; Ouyang, L; Zhu, YX; Wang, T; Shi, JY; Zhang, YW, EUROPEAN JOURNAL OF MEDICINAL CHEMISTRY,141, 257-272, DOI: 10.1016/j.ejmech.2017.09.068, DEC 1 2017, **Document Type:Review**
Isothiocyanate sulforaphane	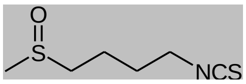	Comparison of Adaptive Neuroprotective Mechanisms of Sulforaphane and its Interconversion Product Erucin in in Vitro and in Vivo Models of Parkinson's Disease, Morroni, F; Sita, G; Djemil, A; D'Amico, M; Pruccoli, L; Cantelli-Forti, G; Hrelia, P; Tarozzi, A, JOURNAL OF AGRICULTURAL AND FOOD CHEMISTRY,66, 4, 856-865, DOI: 10.1021/acs.jafc.7b04641, JAN 31 2018
Isradipine	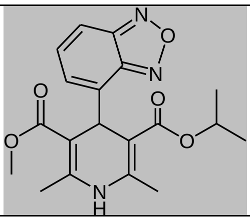	**1)** Disease-Modifying Drugs in Parkinson's Disease, Park, A; Stacy, M, DRUGS,75, 18, 2065-2071, DOI: 10.1007/s40265-015-0497-4, DEC 2015, **Document Type:Review**, **2)** The ongoing pursuit of neuroprotective therapies in Parkinson disease, Athauda, D; Foltynie, T, NATURE REVIEWS NEUROLOGY,11, 1, 25-40, DOI: 10.1038/nrneurol.2014.226, JAN 2015, **Document Type:Review**
Istradefylline	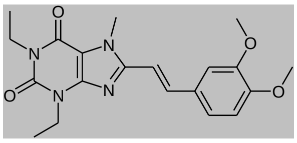	**1)** Istradefylline for the treatment of Parkinson's disease: is it a promising strategy?, Torti, M; Vacca, L; Stocchi, F, EXPERT OPINION ON PHARMACOTHERAPY,19, 16, 1821-1828, DOI: 10.1080/14656566.2018.1524876, NOV 2 2018, **Document Type:Review**, **2)** An Anti-Parkinson's Disease Drug via Targeting Adenosine A Receptor Enhances Amyloid-beta Generation and gamma-Secretase Activity, Lu, J; Cui, J; Li, XH; Wang, X; Zhou, Y; Yang, WJ; Chen, M; Zhao, J; Pei, G, PLOS ONE,11, 11, Article Number: e0166415, DOI: 10.1371/journal.pone.0166415, NOV 11 2016, **3)** The adenosine A receptor antagonist, istradefylline enhances anti-parkinsonian activity induced by combined treatment with low doses of L-DOPA and dopamine agonists in MPTP-treated common marmosets, Uchida, S; Soshiroda, K; Okita, E; Kawai-Uchida, M; Mori, A; Jenner, P; Kanda, T, EUROPEAN JOURNAL OF PHARMACOLOGY,766, 25-30, DOI: 10.1016/j.ejphar.2015.09.028, NOV 5 2015, **4) et cetera**
ITC-57		A novel synthetic isothiocyanate ITC-57 displays antioxidant, anti-inflammatory, and neuroprotective properties in a mouse Parkinson's disease model, Lee, JA; Son, HJ; Kim, JH; Park, KD; Shin, N; Kim, HR; Kim, EM; Kim, DJ; Hwang, O, FREE RADICAL RESEARCH,50, 11, 1188-1199, DOI: 10.1080/10715762.2016.1223293, NOV 2016
JM-20	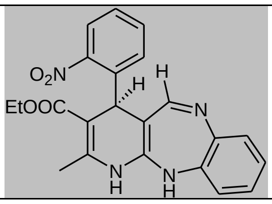	JM-20, a novel hybrid molecule, protects against rotenone-induced neurotoxicity in experimental model of Parkinson's disease, Fonseca-Fonseca, LA; Wong-Guerra, M; Ramirez-Sanchez, J; Montano-Peguero, Y; Yaquis, ASP; Rodriguez, AM; da Silva, VDA; Costa, SL; Pardo-Andreu, GL; Nunez-Figueredo, Y, NEUROSCIENCE LETTERS,690, 29-35, DOI: 10.1016/j.neulet.2018.10.008, JAN 18 2019
JNJ27063699		Anti-Parkinson effects of a selective alpha2C-adrenoceptor antagonist in the MPTP marmoset model, Philippens, IHCHM; Joosen, MJA; Ahnaou, A; Andres, I; Drinkenburg, WHIM, BEHAVIOURAL BRAIN RESEARCH,269, 81-86, DOI: 10.1016/j.bbr.2014.04.028, AUG 1 2014
JWH-015	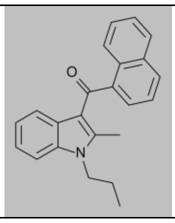	**1)** The role of cannabinoids and leptin in neurological diseases, Agar, E; ACTA NEUROLOGICA SCANDINAVICA, 132, 6, 371-380, DOI: 10.1111/ane.12411, DEC 2015, **Document Type:Review, 2)** Promising cannabinoid-based therapies for Parkinson's disease: motor symptoms to neuroprotection, More, SV; Choi, DK; MOLECULAR NEURODEGENERATION, 10, Article Number: 17, DOI: 10.1186/s13024-015-0012-0, APR 8 2015, **Document Type:Review, 3) et cetera**
Kaempferol	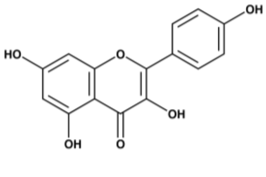	**1)** Flavonoids and its Neuroprotective Effects on Brain Ischemia and Neurodegenerative Diseases, Putteeraj, M; Lim, WL; Teoh, SL; Yahaya, MF, CURRENT DRUG TARGETS,19, 14, 1710-1720, DOI: 10.2174/1389450119666180326125252, 2018, **Document Type:Review, 2)** Natural polyphenols effects on protein aggregates in Alzheimer's and Parkinson's prion-like diseases, Freyssin, A; Page, G; Fauconneau, B; Bilan, AR, NEURAL REGENERATION RESEARCH,13, 6, 955-961, DOI: 10.4103/1673-5374.233432, JUN 2018, **Document Type:Review**, **3)** Polyphenols in Parkinson's Disease: A Systematic Review of In Vivo Studies, Kujawska, M; Jodynis-Liebert, J, NUTRIENTS,10, 5, Article Number: 642, DOI:

		10.3390/nu10050642, MAY 2018, **Document Type:Review**, **4)** Novel tactics for neuroprotection in Parkinson's disease: Role of antibiotics, polyphenols and neuropeptides, Reglodi, D; Renaud, J; Tamas, A; Tizabi, Y; Socias, SB; Del-Bel, E; Raisman-Vozari, R, PROGRESS IN NEUROBIOLOGY,155, 120-148 Special : SI, DOI: 10.1016/j.pneurobio.2015.10.004, AUG 2017, **Document Type:Review**, **5) et cetera**
Kaempferol, 3-O-a-L arabinofuranoside-7-O-a-L-rhamnopyranoside	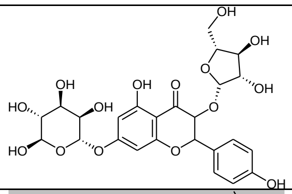	Molecular recognition between potential natural inhibitors of the Keap1-Nrf2 complex, Bello, M; Morales-Gonzalez, JA; INTERNATIONAL JOURNAL OF BIOLOGICAL MACROMOLECULES, 105, 981-992 Part: 1, DOI: 10.1016/j.ijbiomac.2017.07.117, DEC 2017
KF-17837	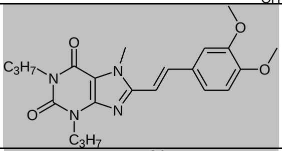	Xanthine derivatives as agents affecting non-dopaminergic neuroprotection in Parkinson`s disease., Kasabova-Angelova, A; Tzankova, D; Mitkov, J; Georgieva, M; Tzankova, V; Zlatkov, A; Kondeva-Burdina, M, Current medicinal chemistry, DOI:10.2174/0929867325666180821153316, 2018-Aug-21
KM-819	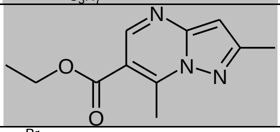	Therapeutic strategies for Parkinson disease: beyond dopaminergic drugs. Charvin, D; Medori, R; Hauser, RA; Rascol, O. NATURE REVIEWS DRUG DISCOVERY, 17, 11, 804-822, DOI: 10.1038/nrd.2018.136, NOV 2018, **Document Type: Review**
KR33493	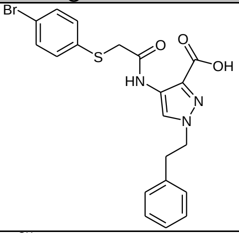	Subacute toxicity evaluation of KR-33493, FAF1 inhibitor for a new anti-parkinson's disease agent, after oral administration in rats and dogs, Jeong, JW; Yu, C; Lee, JH; Moon, KS; Kim, E; Yoo, SE; Koo, TS, REGULATORY TOXICOLOGY AND PHARMACOLOGY,81, 387-396, DOI: 10.1016/j.yrtph.2016.09.022, NOV 2016
Kukoamine	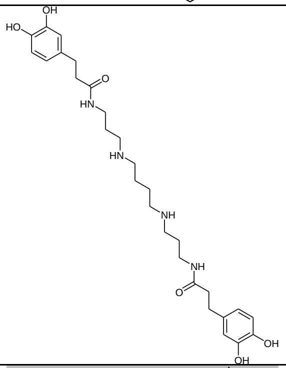	Neuroprotective effects of Kukoamine B against hydrogen peroxide-induced apoptosis and potential mechanisms in SH-SY5Y cells, Hu, XL; Niu, YX; Zhang, Q; Tian, X; Gao, LY; Guo, LP; Meng, WH; Zhao, QC, ENVIRONMENTAL TOXICOLOGY AND PHARMACOLOGY,40, 1, 230-240, DOI: 10.1016/j.etap.2015.06.017, JUL 2015
KW-6002	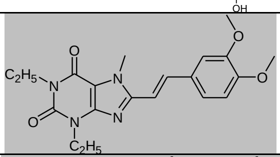	Xanthine derivatives as agents affecting non-dopaminergic neuroprotection in Parkinson`s disease., Kasabova-Angelova, A; Tzankova, D; Mitkov, J; Georgieva, M; Tzankova, V; Zlatkov, A; Kondeva-Burdina, M, Current medicinal chemistry, DOI:10.2174/0929867325666180821153316, 2018-Aug-21
L-701,324	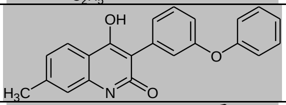	Advances in non-dopaminergic treatments for Parkinson's disease. Stayte, S; Vissel, B; FRONTIERS IN NEUROSCIENCE, 8, Article Number: 113, DOI: 10.3389/fnins.2014.00113, MAY 22 2014, **Document Type: Review**
L-745,870	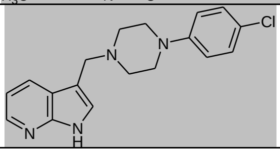	L-745,870 reduces the expression of abnormal involuntary movements in the 6-OHDA-lesioned rat, Huot, P; Johnston, TH; Koprich, JB; Espinosa, MC; Reyes, MG; Fox, SH; Brotchie, JM, BEHAVIOURAL PHARMACOLOGY,26, 1-2, 101-108 Special : SI, DOI: 10.1097/FBP.0000000000000096, FEB 2015
Lactoferrin		Lactoferrin ameliorates dopaminergic neurodegeneration and motor deficits in MPTP-treated mice., Xu, Shuang-Feng; Zhang, Yan-Hui; Wang, Shan; Pang, Zhong-Qiu; Fan, Yong-Gang; Li, Jia-Yi; Wang, Zhan-You; Guo, Chuang, Redox biology, 21 :101090, DOI:10.1016/j.redox.2018.101090, 2019-02
Landipirdine (sYN-120)	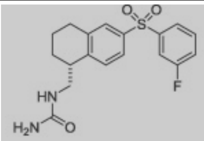	**1)** Therapeutic strategies for Parkinson disease: beyond dopaminergic drugs.Charvin, D; Medori, R; Hauser, RA; Rascol, O. NATURE REVIEWS DRUG DISCOVERY, 17, 11, 804-822, DOI: 10.1038/nrd.2018.136, NOV 2018, **2)** Current approaches to the treatment of Parkinson's Disease, Ellis, JM; Fell, MJ, BIOORGANIC & MEDICINAL CHEMISTRY LETTERS, 27, 18, 4247-4255, DOI: 10.1016/j.bmcl.2017.07.075, Published: SEP 15 2017

Lenalidomide	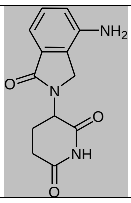	Lenalidomide reduces microglial activation and behavioral deficits in a transgenic model of Parkinson's disease, Valera, E; Mante, M; Anderson, S; Rockenstein, E; Masliah, E, JOURNAL OF NEUROINFLAMMATION,12, Article Number: 93, DOI: 10.1186/s12974-015-0320-x, MAY 14 2015
Leptin		The role of cannabinoids and leptin in neurological diseases, Agar, E, ACTA NEUROLOGICA SCANDINAVICA,132, 6, 371-380, DOI: 10.1111/ane.12411, DEC 2015, **Document Type:Review**
Lestaurtinib	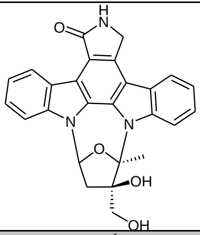	Marine-derived protein kinase inhibitors for neuroinflammatory diseases, Ning, C; Wang, HMD; Gao, R; Chang, YC; Hu, FQ; Meng, XJ; Huang, SY, BIOMEDICAL ENGINEERING ONLINE,17, Article Number: 46, DOI: 10.1186/s12938-018-0477-5, APR 24 2018, **Document Type:Review**
Levetiracetam	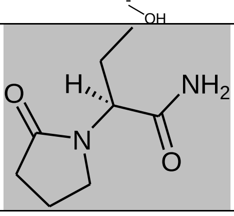	**1)** Levetiracetam attenuates rotenone-induced toxicity: A rat model of Parkinson's disease, Erbas, O; Yilmaz, M; Taskiran, D, ENVIRONMENTAL TOXICOLOGY AND PHARMACOLOGY, 42, 226-230, DOI: 10.1016/j.etap.2016.02.005, MAR 2016, **2)** Neuroprotective effects of levetiracetam target xCT in astrocytes in parkinsonian mice, Miyazaki, I; Murakami, S; Torigoe, N; Kitamura, Y; Asanuma, M, JOURNAL OF NEUROCHEMISTRY,136, 1, 194-204, DOI: 10.1111/jnc.13405, JAN 2016
Liatermin		Advances in Drug Development for Parkinson's Disease: Present Status. Reddy, DH; Misra, S; Medhi, B. PHARMACOLOGY, 93, 5-6, 260-271, DOI: 10.1159/000362419, 2014
Licopyranocoumarin	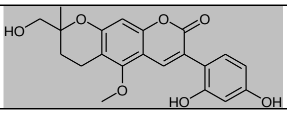	Identification of Licopyranocoumarin and Glycyrurol from Herbal Medicines as Neuroprotective Compounds for Parkinson's Disease, Fujimaki, T; Saiki, S; Tashiro, E; Yamada, D; Kitagawa, M; Hattori, N; Imoto, M, PLOS ONE,9, 6, Article Number: e100395, DOI: 10.1371/journal.pone.0100395, JUN 24 2014
Licochalcone A	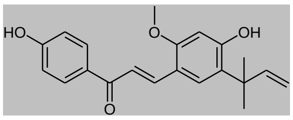	Licochalcone A Prevents the Loss of Dopaminergic Neurons by Inhibiting Microglial Activation in Lipopolysaccharide -Induced Parkinson's Disease Models, Huang, BX; Liu, JX; Ju, C; Yang, DX; Chen, GX; Xu, SY; Zeng, YL; Yan, X; Wang, W; Liu, DF et al, INTERNATIONAL JOURNAL OF MOLECULAR SCIENCES,18, 10, Article Number: 2043, DOI: 10.3390/ijms18102043, OCT 2017
Limonoids	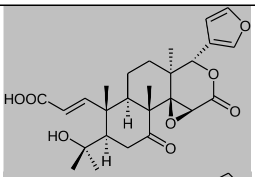 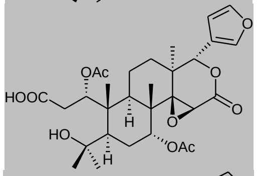 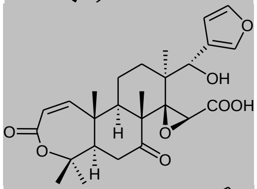 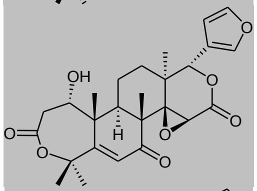 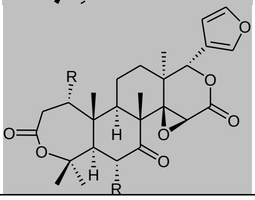	Limonoids from the root bark of Dictamnus angustifolius: potent neuroprotective agents with biometal chelation and halting copper redox cycling properties, Sun, JB; Jiang, N; Lv, MY; Wang, P; Xu, FG; Liang, JY; Qu, W, RSC ADVANCES,5, 31, 24750-24757, DOI: 10.1039/c5ra00278h, 2015

	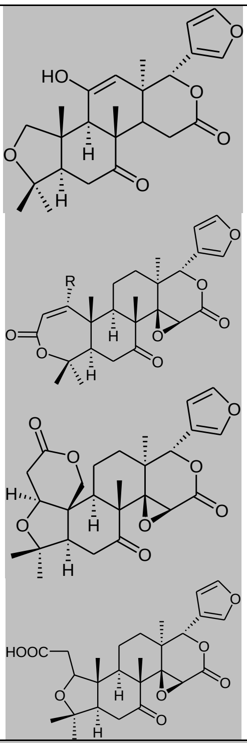	
Linagliptin	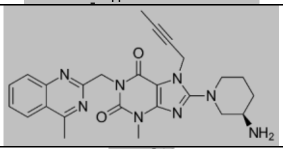	Parkinson's Disease, Diabetes and Cognitive Impairment, Ashraghi, MR; Pagano, G; Polychronis, S; Niccolini, F; Politis, M, RECENT PATENTS ON ENDOCRINE METABOLIC & IMMUNE DRUG DISCOVERY,10, 1, 11-21, DOI: 10.2174/1872214810999160628105549, 2016, **Document Type: Review**
Linalool	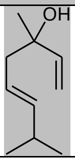	Application of Monoterpenoids and their Derivatives for Treatment of Neurodegenerative Disorders, Volcho, KP; Laev, SS; Ashraf, GM; Aliev, G; Salakhutdinov, NF, CURRENT MEDICINAL CHEMISTRY,25, 39, 5327-5346, DOI: 10.2174/0929867324666170112101837, 2018, **Document Type:Review**
Lipoic acid	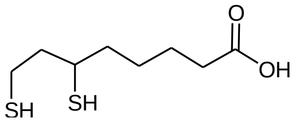	**1)** alpha-Lipoic acid, functional fatty acid, as a novel therapeutic alternative for central nervous system diseases, Seifar, F; Khalili, M; Khaledyan, H; Moghadam, SA; Izadi, A; Azimi, A; Shakouri, SK, NUTRITIONAL NEUROSCIENCE,22, 5, 306-316, DOI: 10.1080/1028415X.2017.1386755, 2019, **Document Type: Review**, **2)** Neuroprotective effect of the carnosine - alpha-lipoic acid nanomicellar complex in a model of early-stage Parkinson's disease, Kulikova, OI; Berezhnoy, DS; Stvolinsky, SL; Lopachev, AV; Orlova, VS; Fedorova, TN, REGULATORY TOXICOLOGY AND PHARMACOLOGY,95, 254-259, DOI: 10.1016/j.yrtph.2018.03.025, JUN 2018, **3)** Lipoic acid alleviates L-DOPA-induced dyskinesia in 6-OHDA parkinsonian rats via anti-oxidative stress, Zhang, SF; Xie, CL; Lin, JY; Wang, MH; Wang, XJ; Liu, ZG, MOLECULAR MEDICINE REPORTS,17, 1, 1118-1124, DOI: 10.3892/mmr.2017.7974, JAN 2018, **4) et cetera**
Liquiritigenin	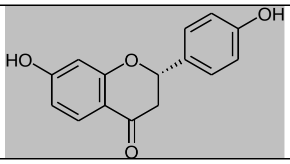	Phytochemical and Pharmacological Role of Liquiritigenin and Isoliquiritigenin From Radix Glycyrrhizae in Human Health and Disease Models, Ramalingam, M; Kim, H; Lee, Y; Lee, YI, FRONTIERS IN AGING NEUROSCIENCE,10, Article Number: 348, DOI: 10.3389/fnagi.2018.00348, NOV 1 2018, **Document Type: Review**
Liraglutide		**1)** Sitagliptin and liraglutide reversed nigrostriatal degeneration of rodent brain in rotenone-induced Parkinson's disease, Badawi, GA; Abd El Fattah, MA; Zaki, HF; El Sayed, MI, INFLAMMOPHARMACOLOGY,25, 3, 369-382, DOI: 10.1007/s10787-017-0331-6, JUN 2017, 2) Parkinson's Disease, Diabetes and Cognitive Impairment, Ashraghi, MR; Pagano, G; Polychronis, S; Niccolini, F; Politis, M, RECENT PATENTS ON ENDOCRINE METABOLIC & IMMUNE DRUG DISCOVERY,10, 1, 11-21, DOI: 10.2174/1872214810999160628105549, 2016, **Document Type: Review, 3)** Neuroprotective Effects of Lixisenatide and Liraglutide in the 1-Methyl-4-Phenyl-1,2,3,6-Tetrahydropyridine Mouse Model of Parkinson's Disease, Liu, W; Jalewa, J; Sharma, M; Li, G; Li, L; Holscher, C, NEUROSCIENCE,303, 42-50, DOI: 10.1016/j.neuroscience.2015.06.054, SEP 10 2015

Lisuride	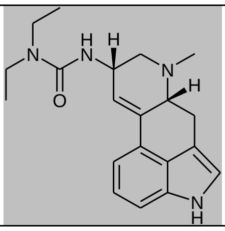	Determination of six anti-Parkinson drugs using cyclodextrin-capillary electrophoresis method: application to pharmaceutical dosage forms, Zeid, AM; Nasr, JJM; Belal, FF; Kitagawa, S; Kaji, N; Baba, Y; Walash, MI, RSC ADVANCES,6, 21, 17519-17530, DOI: 10.1039/c5ra26473a, 2016
Lithium	Li	**1)** Molecular Mechanisms of Lithium Action: Switching the Light on Multiple Targets for Dementia Using Animal Models, Kerr, F; Bjedov, I; Sofola-Adesakin, O, FRONTIERS IN MOLECULAR NEUROSCIENCE,11, Article Number: 297, DOI: 10.3389/fnmol.2018.00297, AUG 28 2018, **Document Type:Review**, **2)** Potential application of lithium in Parkinson's and other neurodegenerative diseases, Lazzara, CA; Kim, YH, FRONTIERS IN NEUROSCIENCE,9, Article Number: 403, DOI: 10.3389/fnins.2015.00403, OCT 27 2015, **Document Type:Review**, **3) et cetera**
Lixisenatide		**1)** Parkinson's Disease, Diabetes and Cognitive Impairment, Ashraghi, MR; Pagano, G; Polychronis, S; Niccolini, F; Politis, M, RECENT PATENTS ON ENDOCRINE METABOLIC & IMMUNE DRUG DISCOVERY,10, 1, 11-21, DOI: 10.2174/1872214810999160628105549, 2016, **Document Type: Review, 2)** Neuroprotective effects of lixisenatide and liraglutide in the 1-methyl-4-phenyl-1,2,3,6-tetrahydropyridine mouse model of parkinson's disease, Liu, W; Jalewa, J; Sharma, M; Li, G; Li, L; Holscher, C, NEUROSCIENCE,303, 42-50, DOI: 10.1016/j.neuroscience.2015.06.054, SEP 10 2015
Lobinaline	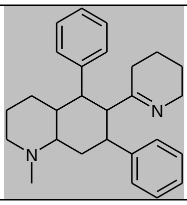	Novel multifunctional pharmacology of lobinaline, the major alkaloid from Lobelia cardinalis, Brown, DP; Rogers, DT; Pomerleau, F; Siripurapu, KB; Kulshrestha, M; Gerhardt, GA; Littleton, JM, FITOTERAPIA,111, 109-123, DOI: 10.1016/j.fitote.2016.04.013, JUN 2016
Loganin	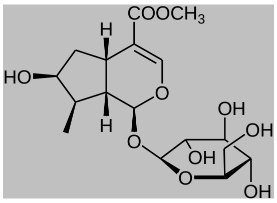	**1)** Application of Monoterpenoids and their Derivatives for Treatment of Neurodegenerative Disorders, Volcho, KP; Laev, SS; Ashraf, GM; Aliev, G; Salakhutdinov, NF, CURRENT MEDICINAL CHEMISTRY,25, 39, 5327-5346, DOI: 10.2174/0929867324666170112101837, 2018, **Document Type:Review, 2)** Unexpected Neuroprotective Effects of Loganin on 1-Methyl-4-Phenyl-1,2,3,6-Tetrahydropyridine-Induced Neurotoxicity and Cell Death in Zebrafish, Yao, L; Peng, SX; Xu, YD; Lin, SL; Li, YH; Liu, CJ; Zhao, HD; Wang, LF; Shen, YQ, JOURNAL OF CELLULAR BIOCHEMISTRY,118, 3, 615-628, DOI: 10.1002/jcb.25749, MAR 2017
Lovastatin	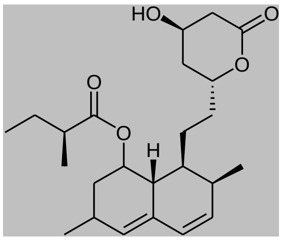	**1)** The beneficial effects of HMG-CoA reductase inhibitors in the processes of neurodegeneration, Saravi, SSS; Saravi, SSS; Arefidoust, A; Dehpour, AR, METABOLIC BRAIN DISEASE,32, 4, 949-965, DOI: 10.1007/s11011-017-0021-5, AUG 2017, **Document Type:Review**, **2)** Neuroprotective mechanisms of statins in neurodegenerative diseases, Yan, JQ; Sun, JC; Li, XM; Zhai, MM; Jia, XF, INTERNATIONAL JOURNAL OF CLINICAL AND EXPERIMENTAL MEDICINE,9, 6, 9799-9805, 2016, **Document Type:Review, 3)** Lovastatin protects neurite degeneration in LRRK2-G2019S parkinsonism through activating the Akt/Nrf pathway and inhibiting GSK3 beta activity, Lin, CH; Lin, HI; Chen, ML; Lai, TT; Cao, LP; Farrer, MJ; Wu, RM; Chien, CT, HUMAN MOLECULAR GENETICS,25, 10, 1965-1978, DOI: 10.1093/hmg/ddw068, MAY 15 2016
LTI-291	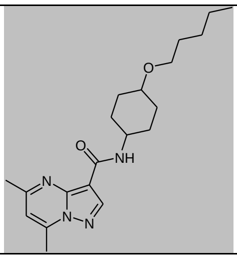	Therapeutic strategies for Parkinson disease: beyond dopaminergic drugs. Charvin, D; Medori, R; Hauser, RA; Rascol, O. NATURE REVIEWS DRUG DISCOVERY, 17, 11, 804-822, DOI: 10.1038/nrd.2018.136, NOV 2018, **Document Type: Review**
Lumbricusin		Neurotropic and neuroprotective activities of the earthworm peptide Lumbricusin, Kim, DH; Lee, IH; Nam, ST; Hong, J; Zhang, P; Hwang, JS; Seok, H; Choi, H; Lee, DG; Kim, JI et al, BIOCHEMICAL AND BIOPHYSICAL RESEARCH COMMUNICATIONS,448, 3, 292-297, DOI: 10.1016/j.bbrc.2014.04.105, JUN 6 2014
Luteolin	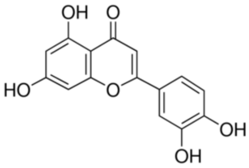	Flavonoids and its Neuroprotective Effects on Brain Ischemia and Neurodegenerative Diseases, Putteeraj, M; Lim, WL; Teoh, SL; Yahaya, MF, CURRENT DRUG TARGETS,19, 14, 1710-1720, DOI: 10.2174/1389450119666180326125252, 2018, **Document Type:Review**

LY354740	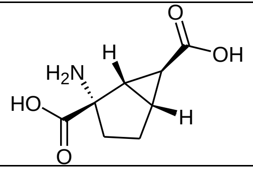	Neuroprotective effects of metabotropic glutamate receptor group II and III activators against MPP-induced cell death in human neuroblastoma SH-SY5Y cells: The impact of cell differentiation state, Jantas, D; Greda, A; Golda, S; Korostynski, M; Grygier, B; Roman, A; Pilc, A; Lason, W, NEUROPHARMACOLOGY,83, 36-53, DOI: 10.1016/j.neuropharm.2014.03.019, AUG 2014
LY367386		Advances in non-dopaminergic treatments for Parkinson's disease. Stayte, S; Vissel, B ; FRONTIERS IN NEUROSCIENCE, 8, Article Number: 113, DOI: 10.3389/fnins.2014.00113, MAY 22 2014
M10	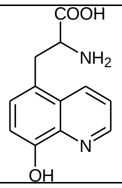	Iron Chelating Strategies in Systemic Metal Overload, Neurodegeneration and Cancer. Gumienna-Kontecka, E; Pyrkosz-Bulska, M; Szebesczyk, A; Ostrowska, M. CURRENT MEDICINAL CHEMISTRY, 21, 33, 3741-3767, DOI: 10.2174/0929867321666140706143402, 2014. **Document Type: Review**
M30 (VAR10303)	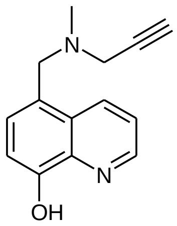	**1)** The novel multitarget iron chelating and propargylamine drug M30 affects APP regulation and processing activities in Alzheimer's disease models, Amit, T; Bar-Am, O; Mechlovich, D; Kupershmidt, L; Youdim, MBH; Weinreb, O, NEUROPHARMACOLOGY,123, 359-367, DOI: 10.1016/j.neuropharm.2017.05.026, SEP 1 2017, **Document Type:Review**, **2)** Iron Chelators and Antioxidants Regenerate Neuritic Tree and Nigrostriatal Fibers of MPP plus /MPTP-Lesioned Dopaminergic Neurons, Aguirre, P; Mena, NP; Carrasco, CM; Munoz, Y; Perez-Henriquez, P; Morales, RA; Cassels, BK; Mendez-Galvez, C; Garcia-Beltran, O; Gonzalez-Billault, C et al, PLOS ONE,10, 12, Article Number: e0144848, DOI: 10.1371/journal.pone.0144848, DEC 14 2015
M30D	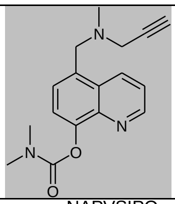	Development of Multifunctional Molecules as Potential Therapeutic Candidates for Alzheimer's Disease, Parkinson's Disease, and Amyotrophic Lateral Sclerosis in the Last Decade. Savelieff, MG; Nam, G; Kang, J; Lee, HJ; Lee, M; Lim, MH. CHEMICAL REVIEWS, 119, 2, 1221-1322, DOI: 10.1021/acs.chemrev.8b00138, JAN 23 2019, **Document Type: Review**
M99	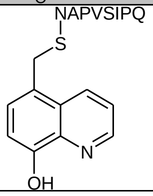	Iron Chelating Strategies in Systemic Metal Overload, Neurodegeneration and Cancer. Gumienna-Kontecka, E; Pyrkosz-Bulska, M; Szebesczyk, A; Ostrowska, M. CURRENT MEDICINAL CHEMISTRY, 21, 33, 3741-3767, DOI: 10.2174/0929867321666140706143402, 2014. **Document Type: Review**
Macranthoin G	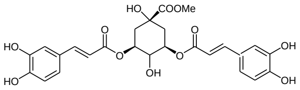	Neuroprotective effects of macranthoin G from Eucommia ulmoides against hydrogen peroxide-induced apoptosis in PC12 cells via inhibiting NF-kappa B activation, Hu, WC; Wang, GC; Li, PX; Wang, YN; Si, CL; He, J; Long, W; Bai, YJ; Feng, ZS; Wang, XF, CHEMICO-BIOLOGICAL INTERACTIONS,224, 108-116, DOI: 10.1016/j.cbi.2014.10.011, DEC 5 2014
Magnesium lithospermate B	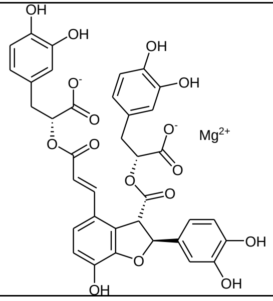	Magnesium Lithospermate B Suppresses Lipopolysaccharide-Induced Neuroinflammation in BV2 Microglial Cells and Attenuates Neurodegeneration in Lipopolysaccharide-Injected Mice, Tai, YJ; Qiu, YJ; Bao, ZC, JOURNAL OF MOLECULAR NEUROSCIENCE,64, 1, 80-92, DOI: 10.1007/s12031-017-1007-9, JAN 2018
Magnolol	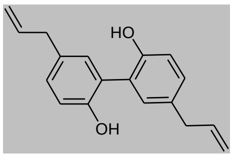	**1)** Quantitative analysis of the therapeutic effect of magnolol on MPTP-induced mouse model of Parkinson's disease using in vivo F-18-9fluoropropyl--dihydrotetrabenazine PET imaging, Weng, CC; Chen, ZA; Chao, KT; Ee, TW; Lin, KJ; Chan, MH; Hsiao, IT; Yen, TC; Kung, MP; Hsu, CH et al, PLOS ONE,12, 3, Article Number: e0173503, DOI: 10.1371/journal.pone.0173503, MAR 3 2017, **2)** Recent advances in discovery and development of natural products as source for anti-Parkinson's disease lead compounds, Zhang, HJ; Bai, L; He, J; Zhong, L; Duan, XM; Ouyang, L; Zhu, YX; Wang, T; Shi, JY; Zhang, YW, EUROPEAN JOURNAL OF MEDICINAL CHEMISTRY,141, 257-272, DOI: 10.1016/j.ejmech.2017.09.068, DEC 1 2017, **Document Type:Review**
Malvidin	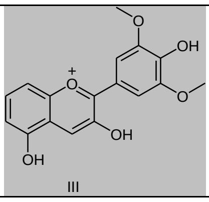 III	Anthocyanins Improve Hippocampus-Dependent Memory Function and Prevent Neurodegeneration via JNK/Akt/GSK3 Signaling in LPS-Treated Adult Mice, Khan, MS; Ali, T; Kim, MW; Jo, MH; Chung, JI; Kim, MO; MOLECULAR NEUROBIOLOGY, 56, 1, 671-687, DOI: 10.1007/s12035-018-1101-1, JAN 2019

α-Mangostin	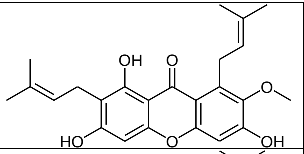	Neuroprotective Effects of Alpha-Mangostin on MPP+-Induced Apoptotic Cell Death in Neuroblastoma SH-SY5Y Cells, Janhom, P; Dharmasaroja, P, JOURNAL OF TOXICOLOGY, Article Number: 919058, DOI: 10.1155/2015/919058, 2015
γ-Mangostin	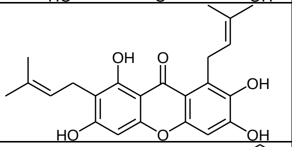	Protective effects of gamma-mangostin on 6-OHDA-induced toxicity in SH-SY5Y cells, Jaisin, Y; Ratanachamnong, P; Kuanpradit, C; Khumpum, W; Suksamrarn, S, NEUROSCIENCE LETTERS,665, 229-235, DOI: 10.1016/j.neulet.2017.11.059, FEB 5 2018
MAOI-1	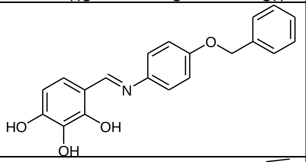	Development of Multifunctional Molecules as Potential Therapeutic Candidates for Alzheimer's Disease, Parkinson's Disease, and Amyotrophic Lateral Sclerosis in the Last Decade. Savelieff, MG; Nam, G; Kang, J; Lee, HJ; Lee, M; Lim, MH. CHEMICAL REVIEWS, 119, 2, 1221-1322, DOI: 10.1021/acs.chemrev.8b00138, JAN 23 2019, **Document Type: Review**
MAOI-2	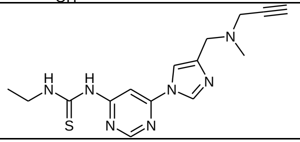	Development of Multifunctional Molecules as Potential Therapeutic Candidates for Alzheimer's Disease, Parkinson's Disease, and Amyotrophic Lateral Sclerosis in the Last Decade. Savelieff, MG; Nam, G; Kang, J; Lee, HJ; Lee, M; Lim, MH. CHEMICAL REVIEWS, 119, 2, 1221-1322, DOI: 10.1021/acs.chemrev.8b00138, JAN 23 2019, **Document Type: Review**
MAOI-3	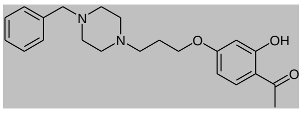	Development of Multifunctional Molecules as Potential Therapeutic Candidates for Alzheimer's Disease, Parkinson's Disease, and Amyotrophic Lateral Sclerosis in the Last Decade. Savelieff, MG; Nam, G; Kang, J; Lee, HJ; Lee, M; Lim, MH. CHEMICAL REVIEWS, 119, 2, 1221-1322, DOI: 10.1021/acs.chemrev.8b00138, JAN 23 2019, **Document Type: Review**
MAOI-4	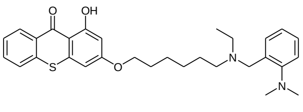	Development of Multifunctional Molecules as Potential Therapeutic Candidates for Alzheimer's Disease, Parkinson's Disease, and Amyotrophic Lateral Sclerosis in the Last Decade. Savelieff, MG; Nam, G; Kang, J; Lee, HJ; Lee, M; Lim, MH. CHEMICAL REVIEWS, 119, 2, 1221-1322, DOI: 10.1021/acs.chemrev.8b00138, JAN 23 2019, **Document Type: Review**
MAOI-5	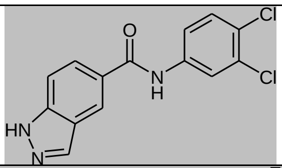	Development of Multifunctional Molecules as Potential Therapeutic Candidates for Alzheimer's Disease, Parkinson's Disease, and Amyotrophic Lateral Sclerosis in the Last Decade. Savelieff, MG; Nam, G; Kang, J; Lee, HJ; Lee, M; Lim, MH. CHEMICAL REVIEWS, 119, 2, 1221-1322, DOI: 10.1021/acs.chemrev.8b00138, JAN 23 2019, **Document Type: Review**
MAOI-6	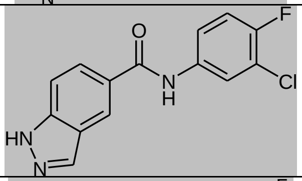	Development of Multifunctional Molecules as Potential Therapeutic Candidates for Alzheimer's Disease, Parkinson's Disease, and Amyotrophic Lateral Sclerosis in the Last Decade. Savelieff, MG; Nam, G; Kang, J; Lee, HJ; Lee, M; Lim, MH. CHEMICAL REVIEWS, 119, 2, 1221-1322, DOI: 10.1021/acs.chemrev.8b00138, JAN 23 2019, **Document Type: Review**
MAOI-7	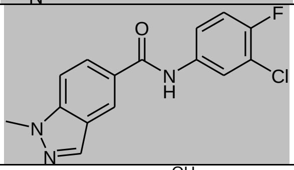	Development of Multifunctional Molecules as Potential Therapeutic Candidates for Alzheimer's Disease, Parkinson's Disease, and Amyotrophic Lateral Sclerosis in the Last Decade. Savelieff, MG; Nam, G; Kang, J; Lee, HJ; Lee, M; Lim, MH. CHEMICAL REVIEWS, 119, 2, 1221-1322, DOI: 10.1021/acs.chemrev.8b00138, JAN 23 2019, **Document Type: Review**
MAOI-8	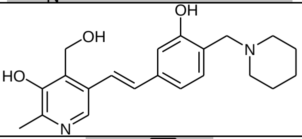	Development of Multifunctional Molecules as Potential Therapeutic Candidates for Alzheimer's Disease, Parkinson's Disease, and Amyotrophic Lateral Sclerosis in the Last Decade. Savelieff, MG; Nam, G; Kang, J; Lee, HJ; Lee, M; Lim, MH. CHEMICAL REVIEWS, 119, 2, 1221-1322, DOI: 10.1021/acs.chemrev.8b00138, JAN 23 2019, **Document Type: Review**
Mavoglurant	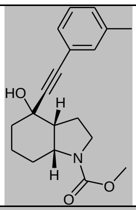	Mavoglurant as a treatment for Parkinson's disease, Petrov, D; Pedros, I; de Lemos, ML; Pallas, M; Canudas, AM; Lazarowski, A; Beas-Zarate, C; Auladell, C; Folch, J; Camins, A, EXPERT OPINION ON INVESTIGATIONAL DRUGS,23, 8, 1165-1179, DOI: 10.1517/13543784.2014.931370, AUG 2014
MDG548	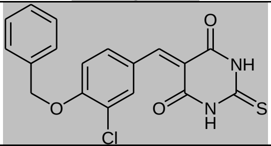	Neuroprotective and anti-inflammatory properties of a novel non-thiazolidinedione PPAR gamma agonist in vitro and in MPTP-treated mice, Lecca, D; Nevin, DK; Mulas, G; Casu, MA; Diana, A; Rossi, D; Sacchetti, G; Carta, AR, NEUROSCIENCE,302, 23-35, DOI: 10.1016/j.neuroscience.2015.04.026, AUG 27 2015
Mebendazole	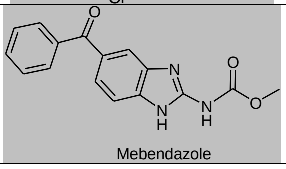 Mebendazole	Antihelminthic Benzimidazoles Are Novel HIF Activators That Prevent Oxidative Neuronal Death via Binding to Tubulin, Aleyasin, H; Karuppagounder, SS; Kumar, A; Sleiman, S; Basso, M; Ma, T; Siddiq, A; Chinta, SJ; Brochier, C; Langley, B et al, ANTIOXIDANTS & REDOX SIGNALING, 22, 2, 121-134, DOI: 10.1089/ars.2013.5595, JAN 10 2015

Meclizine	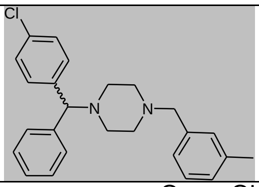	Meclizine-induced enhanced glycolysis is neuroprotective in Parkinson disease cell models, Hong, CT; Chau, KY; Schapira, AHV, SCIENTIFIC REPORTS,6, Article Number: 25344, DOI: 10.1038/srep25344, MAY 5 2016
Meclofenamic acid	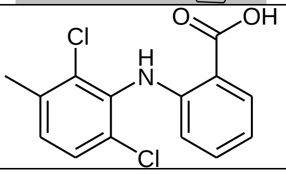	Inhibition of striatal cholinergic interneuron activity by the Kv7 opener retigabine and the nonsteroidal anti-inflammatory drug diclofenac, Paz, RM; Tubert, C; Stahl, A; Diaz, AL; Etchenique, R; Murer, MG; Rela, L, NEUROPHARMACOLOGY,137, 309-321, DOI: 10.1016/j.neuropharm.2018.05.010, JUL 15 2018
Melatonin	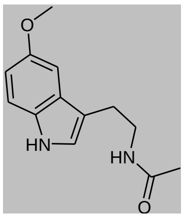	**1)** Melatonin Improves Behavioral and Biochemical Outcomes in a Rotenone-Induced Rat Model of Parkinson's Disease, Rasheed, MZ; Andrabi, SS; Salman, M; Tabassum, H; Shaquiquzzaman, M; Parveen, S; Parvez, S, JOURNAL OF ENVIRONMENTAL PATHOLOGY TOXICOLOGY AND ONCOLOGY,37, 2, 139-150, DOI: 10.1615/JEnvironPatholToxicolOncol.2018025666, 2018, **2)** Melatonin protects against behavioral deficits, dopamine loss and oxidative stress in homocysteine model of Parkinson's disease, Paul, R; Phukan, BC; Thenmozhi, AJ; Manivasagam, T; Bhattacharya, P; Borah, A, LIFE SCIENCES,192, 238-245, DOI: 10.1016/j.lfs.2017.11.016, JAN 1 2018, **3) et cetera**
Meloxicam	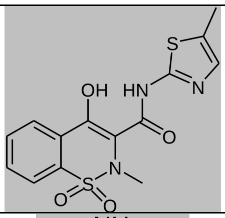	Oxicam-derived non-steroidal anti-inflammatory drugs suppress 1-methyl-4-phenyl pyridinium-induced cell death via repression of endoplasmic reticulum stress response and mitochondrial dysfunction in SH-SY5Y cells, Omura, T; Sasaoka, M; Hashimoto, G; Imai, S; Yamamoto, J; Sato, Y; Nakagawa, S; Yonezawa, A; Nakagawa, T; Yano, I et al, BIOCHEMICAL AND BIOPHYSICAL RESEARCH COMMUNICATIONS,503, 4, 2963-2969, DOI: 10.1016/j.bbrc.2018.08.078, SEP 18 2018
Memantine	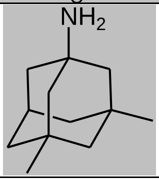	**1)** Memantine selectively blocks extrasynaptic NMDA receptors in rat substantia nigra dopamine neurons, Wu, YN; Johnson, SW, BRAIN RESEARCH,1603, 1-7, DOI: 10.1016/j.brainres.2015.01.041, APR 7 2015, **2)** Effect of memantine on L-dopa-induced dyskinesia in the 6-OHDA-lesioned rat model of Parkinson's disease, Tronci, E; Fidalgo, C; Zianni, E; Collu, M; Stancampiano, R; Morelli, M; Gardoni, F; Carta, M, NEUROSCIENCE,265, 245-252, DOI: 10.1016/j.neuroscience.2014.01.042, APR 18 2014
Merck 22	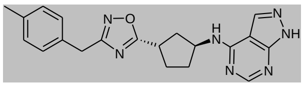	Unprecedented Therapeutic Potential with a Combination of A/NR2B Receptor Antagonists as Observed in the 6-OHDA Lesioned Rat Model of Parkinson's Disease, Michel, A; Downey, P; Nicolas, JM; Scheller, D, PLOS ONE,9, 12, Article Number: e114086, DOI: 10.1371/journal.pone.0114086, DEC 16 2014
Metalloporphyrins		Pre-clinical therapeutic development of a series of metalloporphyrins for Parkinson's disease, Liang, LP; Huang, J; Fulton, R; Pearson-Smith, JN; Day, BJ; Patel, M, TOXICOLOGY AND APPLIED PHARMACOLOGY,326, 34-42, DOI: 10.1016/j.taap.2017.04.004, JUL 1 2017
Metformin (Met)	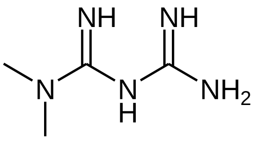	**1)** Metformin Inhibits the Development of L-DOPA-Induced Dyskinesia in a Murine Model of Parkinson's Disease, Ryu, YK; Park, HY; Go, J; Choi, DH; Kim, YH; Hwang, J; Noh, JR; Lee, T; Lee, CH; Kim, KS, MOLECULAR NEUROBIOLOGY,55, 7, 5715-5726, DOI: 10.1007/s12035-017-0752-7, JUL 2018, **2)** Metformin - a Future Therapy for Neurodegenerative Diseases, Markowicz-Piasecka, M; Sikora, J; Szydlowska, A; Skupien, A; Mikiciuk-Olasik, E; Huttunen, KM, PHARMACEUTICAL RESEARCH,34, 12, 2614-2627, DOI: 10.1007/s11095-017-2199-y, DEC 2017, **Document Type:Review**, **3) et cetera**
5-Methoxyflavone	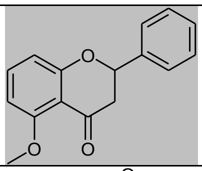	Identification of 5-Methoxyflavone as a Novel DNA Polymerase-Beta Inhibitor and Neuroprotective Agent against Beta-Amyloid Toxicity, Merlo, S; Basile, L; Giuffrida, ML; Sortino, MA; Guccione, S; Copani, A, JOURNAL OF NATURAL PRODUCTS,78, 11, 2704-2711, DOI: 10.1021/acs.jnatprod.5b00621, NOV 2015
Methoxy-6-acetyl-7-methylijuglone	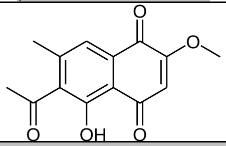	Recent advances in discovery and development of natural products as source for anti-Parkinson's disease lead compounds, Zhang, HJ; Bai, L; He, J; Zhong, L; Duan, XM; Ouyang, L; Zhu, YX; Wang, T; Shi, JY; Zhang, YW, EUROPEAN JOURNAL OF MEDICINAL CHEMISTRY,141, 257-272, DOI: 10.1016/j.ejmech.2017.09.068, DEC 1 2017, **Document Type:Review**
3-((2-Methoxynaphthalen-1-yl)methyl)-7-((pyridin-3-ylmethyl)amino)-5,6,7,8-tetrahydrobenzo[4,5]thieno[2,3-d]pyrimidin-4(3H)-one	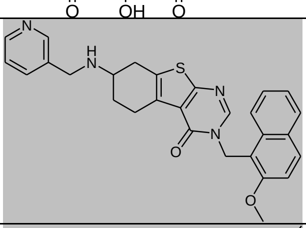	The Discovery of a Highly Selective 5,6,7,8-Tetrahydrobenzothieno pyrimidin-4-one SIRT2 Inhibitor that is Neuroprotective in an in vitro Parkinson's Disease Model, Di Fruscia, P; Zacharioudakis, E; Liu, C; Moniot, S; Laohasinnarong, S; Khongkow, M; Harrison, IF; Koltsida, K; Reynolds, CR; Schmidtkunz, K et al, CHEMMEDCHEM,10, 1, 69-82, DOI: 10.1002/cmdc.201402431, JAN 2015
2-(5-Methyl-1-benzofuran-3-yl)-N-(5-propylsulfanyl-1,3,4-thiadiazol-2-yl) acetamide	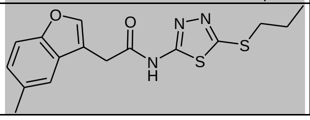	Discovery of a benzofuran derivative as a novel ROCK inhibitor that protects against MPP+-induced oxidative stress and cell death in SH-SY5Y cells, Chong, CM; Shen, MY; Zhou, ZY; Pan, PC; Hoi, PM; Li, S; Liang, W; Ai, NN; Zhang, LQ; Li, CW et al, FREE RADICAL BIOLOGY AND MEDICINE,74, 283-293, DOI:

		10.1016/j.freeradbiomed.2014.06.014, SEP 2014
N'-(4-Methylbenzylidene)-5-phenylisoxazole-3-carbohydrazide	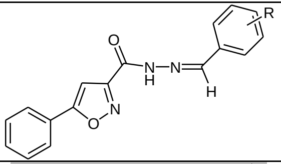	Synthesis, monoamine oxidase inhibitory activity and computational study of novel isoxazole derivatives as potential antiparkinson agents., Agrawal, Neetu; Mishra, Pradeep, Computational biology and chemistry, 79 :63-72, DOI:10.1016/j.compbiolchem.2019.01.012, 2019-Apr
3-Methyl-7H-furo[3,2-g]chromen-7-one	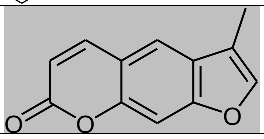	Coumarin analogue 3-methyl-7H-furochromen-7-one as a possible antiparkinsonian agent, Olaya, MD; Vergel, NE; Lopez, JL; Vina, MD; Guerrero, MF, BIOMEDICA,39, 3, 2019
(Z)-Methyl p-hydroxycinnamate	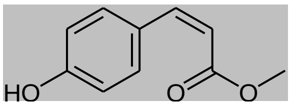	A natural product from Cannabis sativa subsp sativa inhibits homeodomain-interacting protein kinase 2, attenuating MPP+-induced apoptosis in human neuroblastoma SH-SY5Y cells, Wang, G; Zhu, LJ; Zhao, YQ; Gao, SY; Sun, DJ; Yuan, JQ; Huang, YX; Zhang, X; Yao, XS, BIOORGANIC CHEMISTRY,72, 64-73, DOI: 10.1016/j.bioorg.2017.03.011, JUN 2017
1-Methyl-1,2,3,4-tetrahydroisoquinoline	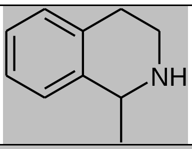	The Protective Effect of Repeated 1MeTIQ Administration on the Lactacystin-Induced Impairment of Dopamine Release and Decline in TH Level in the Rat Brain, Wasik, A; Romanska, I; Zelek-Molik, AZ; Nalepa, I; Antkiewicz-Michaluk, LA, NEUROTOXICITY RESEARCH,34, 3, 706-716, DOI: 10.1007/s12640-018-9939-6, OCT 2018
Methyl yellow	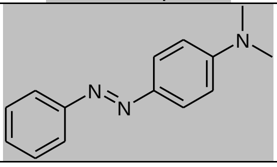	Methyl Yellow: A Potential Drug Scaffold for Parkinson's Disease, Geldenhuys, WJ; Kochi, A; Lin, L; Sutariya, V; Dluzen, DE; Van der Schyf, CJ; Lim, MH, CHEMBIOCHEM,15, 11, 1590-1597, DOI: 10.1002/cbic.201300770, JUL 21 2014
Methylene blue	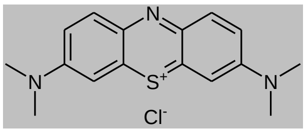	**1)** Methylene blue protects dopaminergic neurons against MPTP-induced neurotoxicity by upregulating brain-derived neurotrophic factor, Bhurtel, S; Katila, N; Neupane, S; Srivastav, S; Park, PH; Choi, DY, ANNALS OF THE NEW YORK ACADEMY OF SCIENCES,1431, 1, 58-71 Special : SI, DOI: 10.1111/nyas.13870, NOV 2018, **2)** Methylene Blue Ameliorates Olfactory Dysfunction and Motor Deficits in a Chronic MPTP/Probenecid Mouse Model of Parkinson's Disease, Biju, KC; Evans, RC; Shrestha, K; Carlisle, DCB; Gelfond, J; Clark, RA, NEUROSCIENCE,380, 111-122, DOI: 10.1016/j.neuroscience.2018.04.008, JUN 1 2018
Mianserin	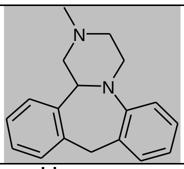	The effect of mianserin on the severity of psychosis and dyskinesia in the parkinsonian marmoset, Hamadjida, A; Nuara, SG; Gourdon, JC; Huot, P, PROGRESS IN NEURO-PSYCHOPHARMACOLOGY & BIOLOGICAL PSYCHIATRY,81, 367-371, DOI: 10.1016/j.pnpbp.2017.09.001, FEB 2 2018
Mildronate	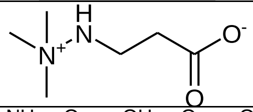	Mildronate Improves Cognition and Reduces Amyloid-beta Pathology in Transgenic Alzheimer's Disease Mice, Beitnere, U; van Groen, T; Kumar, A; Jansone, B; Klusa, V; Kadish, I, JOURNAL OF NEUROSCIENCE RESEARCH,92, 3, 338-346, DOI: 10.1002/jnr.23315, MAR 2014
Minocycline	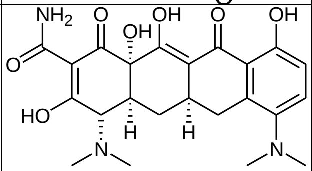	**1)** Novel tactics for neuroprotection in Parkinson's disease: Role of antibiotics, polyphenols and neuropeptides, Reglodi, D; Renaud, J; Tamas, A; Tizabi, Y; Socias, SB; Del-Bel, E; Raisman-Vozari, R; PROGRESS IN NEUROBIOLOGY, 155, 120-148 Special : SI, DOI: 10.1016/j.pneurobio.2015.10.004, AUG 2017, **Document Type:Review, 2)** Development of Multifunctional Molecules as Potential Therapeutic Candidates for Alzheimer's Disease, Parkinson's Disease, and Amyotrophic Lateral Sclerosis in the Last Decade. Savelieff, MG; Nam, G; Kang, J; Lee, HJ; Lee, M; Lim, MH. CHEMICAL REVIEWS, 119, 2, 1221-1322, DOI: 10.1021/acs.chemrev.8b00138, JAN 23 2019, **Document Type:Review, 3)** Minocycline diminishes the rotenone induced neurotoxicity and glial activation via suppression of apoptosis, nitrite levels and oxidative stress, Verma, DK; Singh, DK; Gupta, S; Gupta, P; Singh, A; Biswas, J; Singh, S, NEUROTOXICOLOGY,65, 9-21, DOI: 10.1016/j.neuro.2018.01.006, MAR 2018, **4)** Minocycline protects, rescues and prevents knockdown transgenic parkin Drosophila against paraquat/iron toxicity: Implications for autosomic recessive juvenile parkinsonism, Ortega-Arellano, HF; Jimenez-Del-Rio, M; Velez-Pardo, C, NEUROTOXICOLOGY,60, 42-53 Special : SI, DOI: 10.1016/j.neuro.2017.03.002, MAY 2017, **5) et cetera**
Mitoglitazone (MSDC)	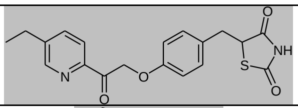	Alpha-Synuclein Glycation and the Action of Anti-Diabetic Agents in Parkinson's Disease, Konig, A; Vicente Miranda, H; Outeiro, TF, JOURNAL OF PARKINSONS DISEASE, 8, 1, 33-43, DOI: 10.3233/JPD-171285, 2018, **Document Type:Review**
Mirtazapine	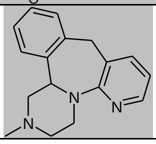	Advances in non-dopaminergic treatments for Parkinson's disease. Stayte, S; Vissel, B; FRONTIERS IN NEUROSCIENCE, 8, Article Number: 113, DOI: 10.3389/fnins.2014.00113, MAY 22 2014, **Document Type: Review**

Mito-apocynin	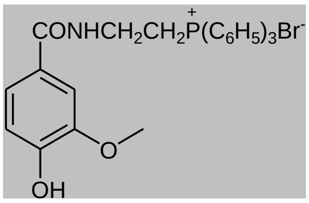	**1)** Mito-Apocynin Prevents Mitochondrial Dysfunction, Microglial Activation, Oxidative Damage, and Progressive Neurodegeneration in MitoPark Transgenic Mice, Langley, M; Ghosh, A; Charli, A; Sarkar, S; Ay, M; Luo, J; Zielonka, J; Brenza, T; Bennett, B; Jin, HJ et al, ANTIOXIDANTS & REDOX SIGNALING,27, 14, 1048-1066, DOI: 10.1089/ars.2016.6905, NOV 10 2017, **2)** Mitoapocynin Treatment Protects Against Neuroinflammation and Dopaminergic Neurodegeneration in a Preclinical Animal Model of Parkinson's Disease, Ghosh, A; Langley, MR; Harischandra, DS; Neal, ML; Jin, HJ; Anantharam, V; Joseph, J; Brenza, T; Narasimhan, B; Kanthasamy, A et al, JOURNAL OF NEUROIMMUNE PHARMACOLOGY,11, 2, 259-278, DOI: 10.1007/s11481-016-9650-4, JUN 2016
Mitomycin C	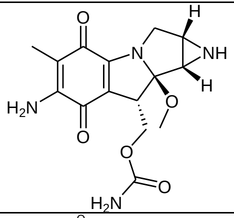	Mitomycin-treated undifferentiated embryonic stem cells as a safe and effective therapeutic strategy in a mouse model of Parkinson's disease, Acquarone, M; de Melo, TM; Meireles, F; Brito-Moreiras, J; Oliveira, G; Ferreira, ST; Castro, NG; Tovar-Moil, F; Houzel, JC; Rehen, SK, FRONTIERS IN CELLULAR NEUROSCIENCE,9, Article Number: 97, DOI: 10.3389/fncel.2015.00097, APR 8 2015
MitoQ	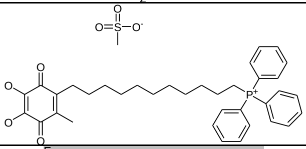	Redox-based therapeutics in neurodegenerative disease. McBean, GJ; Lopez, MG; Wallner, FK. BRITISH JOURNAL OF PHARMACOLOGY, 174, 12, 1750-1770, DOI: 10.1111/bph.13551, JUN 2017, **Document Type:Review**
MK-9470	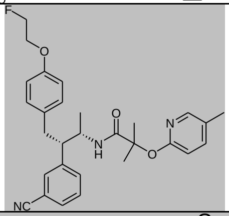	Advances in Drug Development for Parkinson's Disease: Present Status. Reddy, DH; Misra, S; Medhi, B. PHARMACOLOGY, 93, 5-6, 260-271, DOI: 10.1159/000362419, 2014, **Document Type:Review**
ML171	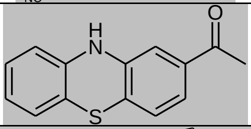	Redox-based therapeutics in neurodegenerative disease. McBean, GJ; Lopez, MG; Wallner, FK. BRITISH JOURNAL OF PHARMACOLOGY, 174, 12, 1750-1770, DOI: 10.1111/bph.13551, JUN 2017, **Document Type: Review**
ML-23	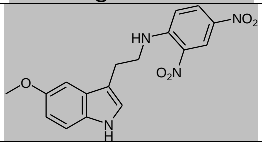	The effect of intravitreal cholinergic drugs on motor control, Willis, GL; Freelance, CB, BEHAVIOURAL BRAIN RESEARCH,339, 232-238, DOI: 10.1016/j.bbr.2017.11.027, FEB 26 2018
Modafinil	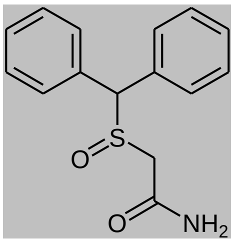	**1)** Modafinil alleviates levodopa-induced excessive nighttime sleepiness and restores monoaminergic systems in a nocturnal animal model of Parkinson's disease, Ando, R; Choudhury, ME; Yamanishi, Y; Kyaw, WT; Kubo, M; Kannou, M; Nishikawa, N; Tanaka, J; Nomoto, M; Nagai, M, JOURNAL OF PHARMACOLOGICAL SCIENCES,136, 4, 266-271, DOI: 10.1016/j.jphs.2018.03.005, APR 2018, **2)** Modafinil attenuates inflammation via inhibiting Akt/NF-kappa B pathway in apoE-deficient mouse model of atherosclerosis, Han, JX; Chen, DW; Liu, DY; Zhu, YA, INFLAMMOPHARMACOLOGY,26, 2, 385-393, DOI: 10.1007/s10787-017-0387-3, APR 2018, **3) et cetera**
Montelukast	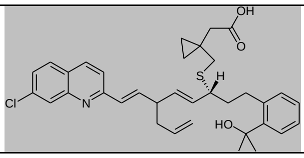	Effect of montelukast in experimental model of Parkinson's disease, Nagarajan, VB; Marathe, PA, NEUROSCIENCE LETTERS,682, 100-105, DOI: 10.1016/j.neulet.2018.05.052, AUG 2018
Morin (3,5,7,29,49-pentahydroxyflavone)	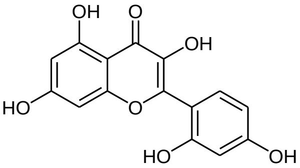	**1)** Efficacy of Morin as a Potential Therapeutic Phytocomponent: Insights into the Mechanism of Action, Choudhury, A; Chakraborty, I; Banerjee, TS; Vana, DR; Adapa, D, INTERNATIONAL JOURNAL OF MEDICAL RESEARCH & HEALTH SCIENCES,6, 11, 175-194, 2017, **Document Type:Review, 2)** Natural polyphenols effects on protein aggregates in Alzheimer's and Parkinson's prion-like diseases, Freyssin, A; Page, G; Fauconneau, B; Bilan, AR, NEURAL REGENERATION RESEARCH,13, 6, 955-961, DOI: 10.4103/1673-5374.233432, JUN 2018, **Document Type:Review**, **3)** Polyphenols in Parkinson's Disease: A Systematic Review of In Vivo Studies, Kujawska, M; Jodynis-Liebert, J, NUTRIENTS,10, 5, Article Number: 642, DOI: 10.3390/nu10050642, MAY 2018, **Document Type:Review**, **4)** Novel tactics for neuroprotection in Parkinson's disease: Role of antibiotics, polyphenols and neuropeptides, Reglodi, D; Renaud, J; Tamas, A; Tizabi, Y; Socias, SB; Del-Bel, E; Raisman-Vozari, R, PROGRESS IN NEUROBIOLOGY,155, 120-148 Special : SI, DOI: 10.1016/j.pneurobio.2015.10.004, AUG 2017, **Document Type:Review**, **5) et cetera**

Moringin	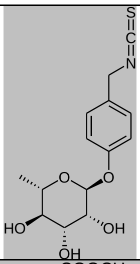	The Isothiocyanate Isolated from Moringa oleifera Shows Potent Anti-Inflammatory Activity in the Treatment of Murine Subacute Parkinson's Disease, Giacoppo, S; Rajan, TS; De Nicola, GR; Iori, R; Rollin, P; Bramanti, P; Mazzon, E, REJUVENATION RESEARCH,20, 1, 52-65, DOI: 10.1089/rej.2016.1828, FEB 2017
Morreniside	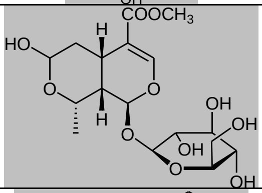	Application of Monoterpenoids and their Derivatives for Treatment of Neurodegenerative Disorders, Volcho, KP; Laev, SS; Ashraf, GM; Aliev, G; Salakhutdinov, NF, CURRENT MEDICINAL CHEMISTRY,25, 39, 5327-5346, DOI: 10.2174/0929867324666170112101837, 2018, **Document Type:Review**
MPEP	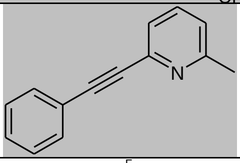	Advances in non-dopaminergic treatments for Parkinson's disease. Stayte, S; Vissel, B; FRONTIERS IN NEUROSCIENCE, 8, Article Number: 113, DOI: 10.3389/fnins.2014.00113, MAY 22 2014, **Document Type: Review**
[18F]MPPF	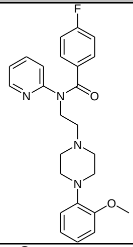	Advances in Drug Development for Parkinson's Disease: Present Status. Reddy, DH; Misra, S; Medhi, B. PHARMACOLOGY, 93, 5-6, 260-271, DOI: 10.1159/000362419, 2014, **Document Type: Review**
MSX-3	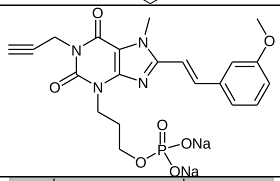	Neuroprotective Potential of Adenosine A2A and Cannabinoid CB1 Receptor Antagonists in an Animal Model of Parkinson Disease, Cerri, S; Levandis, G; Ambrosi, G; Montepeloso, E; Antoninetti, GF; Franco, R; Lanciego, JL; Baqi, Y; Muller, CE; Pinna, A et al, JOURNAL OF NEUROPATHOLOGY AND EXPERIMENTAL NEUROLOGY,73, 5, 414-424, DOI: 10.1097/NEN.0000000000000064, MAY 2014
MT-20R	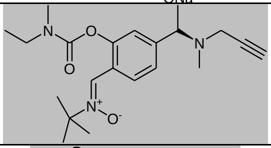	Neuroprotective Effects and Mechanisms of Action of Multifunctional Agents Targeting Free Radicals, Monoamine Oxidase B and Cholinesterase in Parkinson's Disease Model, Liu, Z; Cai, W; Lang, M; Yan, RZ; Li, ZS; Zhang, GX; Yu, P; Wang, YQ; Sun, YW; Zhang, ZJ, JOURNAL OF MOLECULAR NEUROSCIENCE,61, 4, 498-510, DOI: 10.1007/s12031-017-0891-3, APR 2017
MTEP	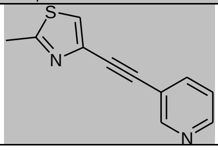	Advances in non-dopaminergic treatments for Parkinson's disease. Stayte, S; Vissel, B; FRONTIERS IN NEUROSCIENCE, 8, Article Number: 113, DOI: 10.3389/fnins.2014.00113, MAY 22 2014, **Document Type: Review**
Myricetin	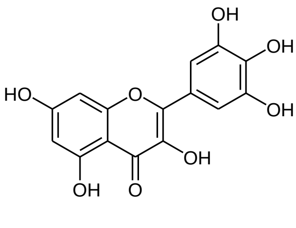	**1)** Effect of Myricetin on the Loss of Dopaminergic Neurons in the Transgenic Drosophila Model of Parkinson's Disease, Ara, G; Afzal, M; Jyoti, S; Naz, F; Rahul; Siddique, YH, CURRENT DRUG THERAPY,14, 1, 58-64, DOI: 10.2174/1574885513666180529114546, 2019, **2)** Exploring the Structural Diversity in Inhibitors of alpha-Synuclein Amyloidogenic Folding, Aggregation, and Neurotoxicity, Das, S; Pukala, TL; Smid, SD, FRONTIERS IN CHEMISTRY,6, Article Number: 181, DOI: 10.3389/fchem.2018.00181, MAY 25 2018, **3)** Flavonoids and its Neuroprotective Effects on Brain Ischemia and Neurodegenerative Diseases, Putteeraj, M; Lim, WL; Teoh, SL; Yahaya, MF, CURRENT DRUG TARGETS,19, 14, 1710-1720, DOI: 10.2174/1389450119666180326125252, 2018, **Document Type:Review, 4) et cetera**
Myricitrin	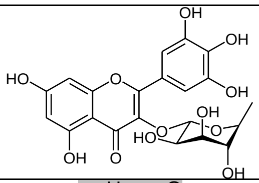	Myricitrin Alleviates Methylglyoxal-Induced Mitochondrial Dysfunction and AGEs/RAGE/NF-kappa B Pathway Activation in SH-SY5Y Cells, Wang, YH; Yu, HT; Pu, XP; Du, GH, JOURNAL OF MOLECULAR NEUROSCIENCE,53, 4, 562-570, DOI: 10.1007/s12031-013-0222-2, AUG 2014
Myrtenal	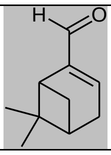	Application of Monoterpenoids and their Derivatives for Treatment of Neurodegenerative Disorders, Volcho, KP; Laev, SS; Ashraf, GM; Aliev, G; Salakhutdinov, NF, CURRENT MEDICINAL CHEMISTRY,25, 39, 5327-5346, DOI: 10.2174/0929867324666170112101837, 2018, **Document Type:Review**

Naloxon	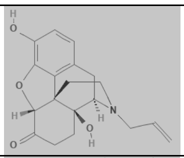	Current and experimental treatments of Parkinson disease: A guide for neuroscientists. Oertel, W; Schulz, JB. JOURNAL OF NEUROCHEMISTRY, 139, 325-337, Supplement: 1, DOI: 10.1111/jnc.13750, OCT 2016
3-(Naphthalen-2-yl(propoxy)methyl)azetidine hydrochloride	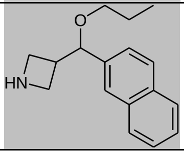	3-azetidine hydrochloride attenuates MPP+-induced cytotoxicity by regulating oxidative stress and mitochondrial dysfunction in SH-SY5Y cells, Yang, SJ; Yang, JW; Na, JM; Ha, JS; Choi, SY; Cho, SW, BMB REPORTS,51, 11, 590-595, DOI: 10.5483/BMBRep.2018.51.11.123, 2018
Naringenin	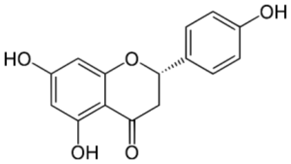	**1)** Flavonoids and its Neuroprotective Effects on Brain Ischemia and Neurodegenerative Diseases, Putteeraj, M; Lim, WL; Teoh, SL; Yahaya, MF, CURRENT DRUG TARGETS,19, 14, 1710-1720, DOI: 10.2174/1389450119666180326125252, 2018, **Document Type:Review, 2)** Naringenin Suppresses Neuroinflammatory Responses Through Inducing Suppressor of Cytokine Signaling 3 Expression, Wu, LH; Lin, C; Lin, HY; Liu, YS; Wu, CYJ; Tsai, CF; Chang, PC; Yeh, WL; Lu, DY, MOLECULAR NEUROBIOLOGY,53, 2, 1080-1091, DOI: 10.1007/s12035-014-9042-9, MAR 2016
Naringin	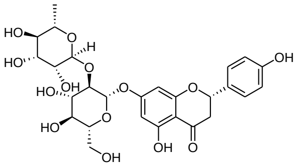	**1)** Recent advances in discovery and development of natural products as source for anti-Parkinson's disease lead compounds, Zhang, HJ; Bai, L; He, J; Zhong, L; Duan, XM; Ouyang, L; Zhu, YX; Wang, T; Shi, JY; Zhang, YW, EUROPEAN JOURNAL OF MEDICINAL CHEMISTRY,141, 257-272, DOI: 10.1016/j.ejmech.2017.09.068, DEC 1 2017, **Document Type:Review, 2)** Effects of naringin, a flavanone glycoside in grapefruits and citrus fruits, on the nigrostriatal dopaminergic projection in the adult brain, Jung, UJ; Kim, SR, NEURAL REGENERATION RESEARCH,9, 16, 1514-1517, DOI: 10.4103/1673-5374.139476, AUG 2014, **Document Type:Review**, **3)** Naringin protects the nigrostriatal dopaminergic projection through induction of GDNF in a neurotoxin model of Parkinson's disease, Leem, E; Nam, JH; Jeon, MT; Shin, WH; Won, SY; Park, SJ; Choi, MS; Jin, BK; Jung, UJ; Kim, SR, JOURNAL OF NUTRITIONAL BIOCHEMISTRY,25, 7, 801-806, DOI: 10.1016/j.jnutbio.2014.03.006, JUL 2014
NBQX (2,3-Dioxo-6-nitro-7-sulfamoylbenzo(f)quinoxaline)	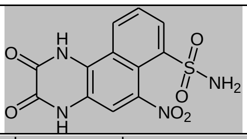	Advances in non-dopaminergic treatments for Parkinson's disease. Stayte, S; Vissel, B; FRONTIERS IN NEUROSCIENCE, 8, Article Number: 113, DOI: 10.3389/fnins.2014.00113, MAY 22 2014, **Document Type: Review**
Nerolidol	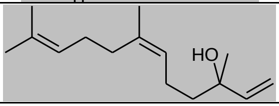	Neuroprotective effect of nerolidol against neuroinflammation and oxidative stress induced by rotenone, Javed, H; Azimullah, S; Khair, SBA; Ojha, S; Haque, ME, BMC NEUROSCIENCE,17, Article Number: 58, DOI: 10.1186/s12868-016-0293-4, AUG 22 2016
Neurotensin analogues NT2 and NT4		Preventive Effect of Two New Neurotensin Analogues on Parkinson's Disease Rat Model, Lazarova, M; Popatanasov, A; Klissurov, R; Stoeva, S; Pajpanova, T; Kalfin, R; Tancheva, L, JOURNAL OF MOLECULAR NEUROSCIENCE,66, 4, 552-560, DOI: 10.1007/s12031-018-1171-6, DEC 2018
Neurturin		Advances in non-dopaminergic treatments for Parkinson's disease. Stayte, S; Vissel, B ; FRONTIERS IN NEUROSCIENCE, 8, Article Number: 113, DOI: 10.3389/fnins.2014.00113, MAY 22 2014
(E)-Nicotinaldehyde O-Cinnamyloxime	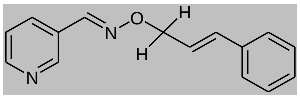	-Nicotinaldehyde O-Cinnamyloxime, a Nicotine Analog, Attenuates Neuronal Cells Death Against Rotenone-Induced Neurotoxicity, Jurado-Coronel, JC; Loaiza, AE; Diaz, JE; Cabezas, R; Ashraf, GM; Sahebkar, A; Echeverria, V; Gonzalez, J; Barreto, GE, MOLECULAR NEUROBIOLOGY,56, 2, 1221-1232, DOI: 10.1007/s12035-018-1163-0, FEB 2019
Nicotinamide	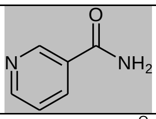	Nicotinamide and neurocognitive function, Rennie, G; Chen, AC; Dhillon, H; Vardy, J; Damian, DL, NUTRITIONAL NEUROSCIENCE,18, 5, 193-200, DOI: 10.1179/1476830514Y.0000000112, JUL 2015, **Document Type:Review**
Nicotinamide adenine dinucleotide phosphate (NADPH)	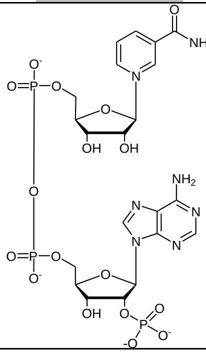	**1)** NADPH ameliorates MPTP-induced dopaminergic neurodegeneration through inhibiting p38MAPK activation, Zhou, JS; Zhu, Z; Wu, F; Zhou, Y; Sheng, R; Wu, JC; Qin, ZH, ACTA PHARMACOLOGICA SINICA,40, 2, 180-191, DOI: 10.1038/s41401-018-0003-0, FEB 2019, **2)** Reduced Nicotinamide Adenine Dinucleotide Phosphate Inhibits MPTP-Induced Neuroinflammation and Neurotoxicity, Zhou, Y; Wu, JC; Sheng, R; Li, M; Wang, Y; Han, R; Han, F; Chen, Z; Qin, ZH, NEUROSCIENCE,391, 140-153, DOI: 10.1016/j.neuroscience.2018.08.032, NOV 1 2018

Nicotinamide mononucleotide	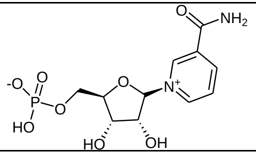	Nicotinamide mononucleotide improves energy activity and survival rate in an in vitro model of Parkinson's disease, Lu, L; Tang, L; Wei, WS; Hong, YY; Chen, HY; Ying, WH; Chen, SD, EXPERIMENTAL AND THERAPEUTIC MEDICINE,8, 3, 943-950, DOI: 10.3892/etm.2014.1842, SEP 2014
Nicotine	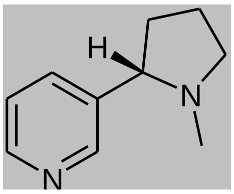	**1)** Recent advances in discovery and development of natural products as source for anti-Parkinson's disease lead compounds, Zhang, HJ; Bai, L; He, J; Zhong, L; Duan, XM; Ouyang, L; Zhu, YX; Wang, T; Shi, JY; Zhang, YW, EUROPEAN JOURNAL OF MEDICINAL CHEMISTRY,141, 257-272, DOI: 10.1016/j.ejmech.2017.09.068, DEC 1 2017, **Document Type:Review, 2)** Disease-Modifying Drugs in Parkinson's Disease, Park, A; Stacy, M, DRUGS,75, 18, 2065-2071, DOI: 10.1007/s40265-015-0497-4, DEC 2015, **Document Type:Review**, **3)** The ongoing pursuit of neuroprotective therapies in Parkinson disease, Athauda, D; Foltynie, T, NATURE REVIEWS NEUROLOGY,11, 1, 25-40, DOI: 10.1038/nrneurol.2014.226, JAN 2015, **Document Type:Review**
Nilotinib	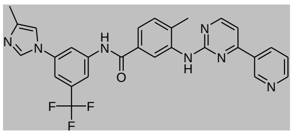	**1)** c-Abl Inhibition Exerts Symptomatic Antiparkinsonian Effects Through a Striatal Postsynaptic Mechanism, Zhou, Y; Yamamura, Y; Ogawa, M; Tsuji, R; Tsuchiya, K; Kasahara, J; Goto, S; FRONTIERS IN PHARMACOLOGY, 9, Article Number: 1311, DOI: 10.3389/fphar.2018.01311, NOV 16 2018, **2)** C-Abl Inhibition; A Novel Therapeutic Target for Parkinson's Disease, Abushouk, AI; Negida, A; Elshenawy, RA; Zein, H; Hammad, AM; Menshawy, A; Mohamed, WMY; CNS & NEUROLOGICAL DISORDERS-DRUG TARGETS, 17, 1, 14-21, DOI: 10.2174/1871527316666170602101538, 2018, **Document Type:Review, 3)** Current approaches to the treatment of Parkinson's Disease, Ellis, JM; Fell, MJ, BIOORGANIC & MEDICINAL CHEMISTRY LETTERS, 27, 18, 4247-4255, DOI: 10.1016/j.bmcl.2017.07.075, Published: SEP 15 2017**, 4)** The c-Abl inhibitor, Nilotinib, protects dopaminergic neurons in a preclinical animal model of Parkinson's disease, Karuppagounder, SS; Brahmachari, S; Lee, Y; Dawson, VL; Dawson, TM; Ko, HS, SCIENTIFIC REPORTS,4, Article Number: 4874, DOI: 10.1038/srep04874, MAY 2 2014
Nitazoxanide	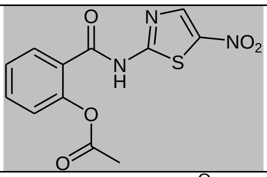	The unintended mitochondrial uncoupling effects of the FDA-approved anti-helminth drug nitazoxanide mitigates experimental parkinsonism in mice, Amireddy, N; Puttapaka, SN; Vinnakota, RL; Ravuri, HG; Thonda, S; Kalivendi, SV, JOURNAL OF BIOLOGICAL CHEMISTRY,292, 38, 15731-15743, DOI: 10.1074/jbc.M117.791863, SEP 22 2017
Nitecapone	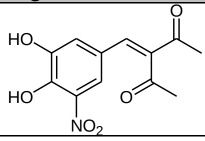	Prevalence and Correlates of Anti-Parkinson Drug Use in a Nationally Representative Sample, Dahodwala, N; Willis, AW; Li, PX; Doshi, JA; MOVEMENT DISORDERS CLINICAL PRACTICE, 4, 3, 335-341, DOI: 10.1002/mdc3.12422, MAY-JUN 2017
NLY01		Block of A1 astrocyte conversion by microglia is neuroprotective in models of Parkinson's disease, Yun, SP; Kam, TI; Panicker, N; Kim, S; Oh, Y; Park, JS; Kwon, SH; Park, YJ; Karuppagounder, SS; Park, H et al, NATURE MEDICINE,24, 7, 931-+, DOI: 10.1038/s41591-018-0051-5, JUL 2018
Nobiletin (citrus flavonoid)	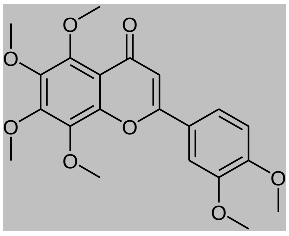	**1)** Neuroprotective Effects of Citrus Fruit-Derived Flavonoids, Nobiletin and Tangeretin in Alzheimer's and Parkinson's Disease, Braidy, N; Behzad, S; Habtemariam, S; Ahmed, T; Daglia, M; Nabavi, SM; Sobarzo-Sanchez, E; Nabavi, SF, CNS & NEUROLOGICAL DISORDERS-DRUG TARGETS,16, 4, 387-397, DOI: 10.2174/1871527316666170328113309, 2017, **Document Type:Review**, **2)** Nobiletin Protects Dopaminergic Neurons in the 1-Methyl-4-Phenylpyridinium-Treated Rat Model of Parkinson's Disease, Jeong, KH; Jeon, MT; Kim, HD; Jung, UJ; Jang, MC; Chu, JW; Yang, SJ; Choi, IY; Choi, MS; Kim, SR, JOURNAL OF MEDICINAL FOOD,18, 4, 409-414, DOI: 10.1089/jmf.2014.3241, APR 1 2015
Nordihydroguaiaretic	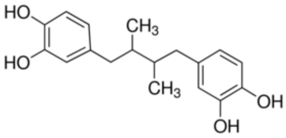	**1)** Natural polyphenols effects on protein aggregates in Alzheimer's and Parkinson's prion-like diseases, Freyssin, A; Page, G; Fauconneau, B; Bilan, AR, NEURAL REGENERATION RESEARCH,13, 6, 955-961, DOI: 10.4103/1673-5374.233432, JUN 2018, **Document Type:Review**, **2)** Polyphenols in Parkinson's Disease: A Systematic Review of In Vivo Studies, Kujawska, M; Jodynis-Liebert, J, NUTRIENTS,10, 5, Article Number: 642, DOI: 10.3390/nu10050642, MAY 2018, **Document Type:Review**, **3)** Novel tactics for neuroprotection in Parkinson's disease: Role of antibiotics, polyphenols and neuropeptides, Reglodi, D; Renaud, J; Tamas, A; Tizabi, Y; Socias, SB; Del-Bel, E; Raisman-Vozari, R, PROGRESS IN NEUROBIOLOGY,155, 120-148 Special : SI, DOI: 10.1016/j.pneurobio.2015.10.004, AUG 2017, **Document Type:Review**, **4) et cetera**
Norfluoxetine	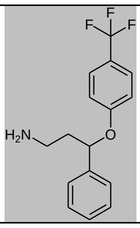	Norfluoxetine Prevents Degeneration of Dopamine Neurons by Inhibiting Microglia-Derived Oxidative Stress in an MPTP Mouse Model of Parkinson's Disease, Kim, KI; Chung, YC; Jin, BK, MEDIATORS OF INFLAMMATION, Article Number: 4591289, DOI: 10.1155/2018/4591289, 2018

Notoginsenoside-Rg1	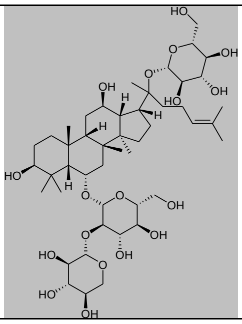	Recent advances in discovery and development of natural products as source for anti-Parkinson's disease lead compounds, Zhang, HJ; Bai, L; He, J; Zhong, L; Duan, XM; Ouyang, L; Zhu, YX; Wang, T; Shi, JY; Zhang, YW, EUROPEAN JOURNAL OF MEDICINAL CHEMISTRY,141, 257-272, DOI: 10.1016/j.ejmech.2017.09.068, DEC 1 2017, **Document Type:Review**
NP-POL nonapeptide		Isolation and Characterization of NP-POL Nonapeptide for Possible Therapeutic Use in Parkinson's Disease, Lemieszewska, M; Polanowski, A; Wilusz, T; Sokolowska, A; Zambrowicz, A; Mikolajewicz, K; Macala, J; Rymaszewska, J; Zablocka, A, OXIDATIVE MEDICINE AND CELLULAR LONGEVITY, Article Number: 3760124, DOI: 10.1155/2018/3760124, 2018
NPT200-11	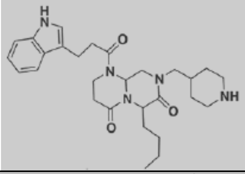	Current and experimental treatments of Parkinson disease: A guide for neuroscientists. Oertel, W; Schulz, JB. JOURNAL OF NEUROCHEMISTRY, 139, 325-337, Supplement: 1, DOI: 10.1111/jnc.13750, OCT 2016
Nuciferoside	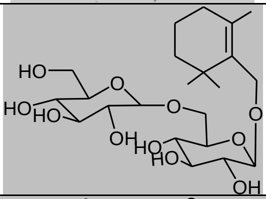	Application of Monoterpenoids and their Derivatives for Treatment of Neurodegenerative Disorders, Volcho, KP; Laev, SS; Ashraf, GM; Aliev, G; Salakhutdinov, NF, CURRENT MEDICINAL CHEMISTRY,25, 39, 5327-5346, DOI: 10.2174/0929867324666170112101837, 2018, **Document Type:Review**
NURR1/NOT agonist	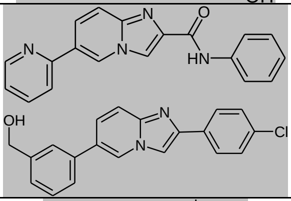	Development of a novel NURR1/NOT agonist from hit to lead and candidate for the potential treatment of Parkinson's disease, Lesuisse, D; Malanda, A; Peyronel, JF; Evanno, Y; Lardenois, P; De-Peretti, D; Abecassis, PY; Barneoud, P; Brunel, P; Burgevin, MC et al, BIOORGANIC & MEDICINAL CHEMISTRY LETTERS,29, 7, 929-932, DOI: 10.1016/j.bmcl.2019.01.024, APR 1 2019
8-OH-DPAT	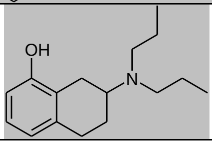	Effect of Buspirone, Fluoxetine and 8-OH-DPAT on Striatal Expression of Bax, Caspase-3 and Bcl-2 Proteins in 6-Hydroxydopamine-Induced Hemi-Parkinsonian Rats, Sharifi, H; Nayebi, AM; Farajnia, S; Haddadi, R, ADVANCED PHARMACEUTICAL BULLETIN,5, 4, 491-495, NOV 2015
Octanoic acid	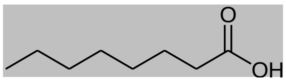	Octanoic acid prevents reduction of striatal dopamine in the MPTP mouse model of Parkinson's disease, Joniec-Maciejak, I; Wawer, A; Turzynska, D; Sobolewska, A; Maciejak, P; Szyndler, J; Mirowska-Guzel, D; Plaznik, A, PHARMACOLOGICAL REPORTS,70, 5, 988-992, DOI: 10.1016/j.pharep.2018.04.008, OCT 2018
Oleoylethanolamide	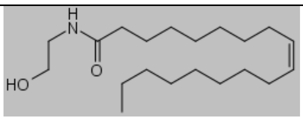	**1)** The role of cannabinoids and leptin in neurological diseases, Agar, E; ACTA NEUROLOGICA SCANDINAVICA, 132, 6, 371-380, DOI: 10.1111/ane.12411, DEC 2015, **Document Type:Review, 2)** Promising cannabinoid-based therapies for Parkinson's disease: motor symptoms to neuroprotection, More, SV; Choi, DK; MOLECULAR NEURODEGENERATION, 10, Article Number: 17, DOI: 10.1186/s13024-015-0012-0, APR 8 2015, **Document Type:Review, 3) et cetera**
Oleuropein	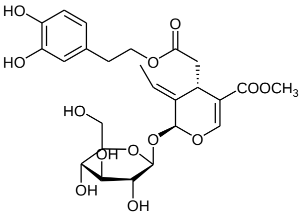	**1)** Oleuropein isolated from Fraxinus rhynchophylla inhibits glutamate-induced neuronal cell death by attenuating mitochondrial dysfunction, Kim, MH; Min, JS; Lee, JY; Chae, U; Yang, EJ; Song, KS; Lee, HS; Lee, HJ; Lee, SR; Lee, DS, NUTRITIONAL NEUROSCIENCE,21, 7, 520-528, DOI: 10.1080/1028415X.2017.1317449, 2018, **2)** Application of Monoterpenoids and their Derivatives for Treatment of Neurodegenerative Disorders, Volcho, KP; Laev, SS; Ashraf, GM; Aliev, G; Salakhutdinov, NF, CURRENT MEDICINAL CHEMISTRY,25, 39, 5327-5346, DOI: 10.2174/0929867324666170112101837, 2018, **Document Type:Review**
Omarigliptin	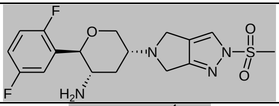	Repositioning of Omarigliptin as a once-weekly intranasal Anti-parkinsonian Agent, Ayoub, BM; Mowaka, S; Safar, MM; Ashoush, N; Arafa, MG; Michel, HE; Tadros, MM; Elmazar, MM; Mousa, SA, SCIENTIFIC REPORTS,8, Article Number: 8959, DOI: 10.1038/s41598-018-27395-0, JUN 12 2018
Omigapil	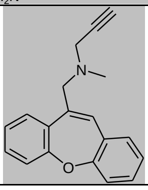	Advances in Drug Development for Parkinson's Disease: Present Status. Reddy, DH; Misra, S; Medhi, B. PHARMACOLOGY, 93, 5-6, 260-271, DOI: 10.1159/000362419, 2014, **Document Type:Review**

Opicapone	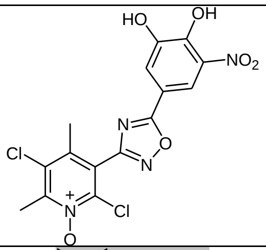	Prevalence and Correlates of Anti-Parkinson Drug Use in a Nationally Representative Sample, Dahodwala, N; Willis, AW; Li, PX; Doshi, JA; MOVEMENT DISORDERS CLINICAL PRACTICE, 4, 3, 335-341, DOI: 10.1002/mdc3.12422, MAY-JUN 2017
Orphenadrine	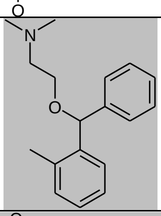	**1)** Prevalence and Correlates of Anti-Parkinson Drug Use in a Nationally Representative Sample, Dahodwala, N; Willis, AW; Li, PX; Doshi, JA; MOVEMENT DISORDERS CLINICAL PRACTICE, 4, 3, 335-341, DOI: 10.1002/mdc3.12422, MAY-JUN 2017, **2)** Advances in non-dopaminergic treatments for Parkinson's disease. Stayte, S; Vissel, B; FRONTIERS IN NEUROSCIENCE, 8, Article Number: 113, DOI: 10.3389/fnins.2014.00113, MAY 22 2014, **Document Type: Review**
Oxibendazole	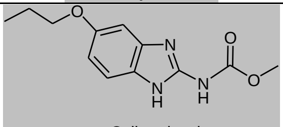 Oxibendazole	Antihelminthic Benzimidazoles Are Novel HIF Activators That Prevent Oxidative Neuronal Death via Binding to Tubulin, Aleyasin, H; Karuppagounder, SS; Kumar, A; Sleiman, S; Basso, M; Ma, T; Siddiq, A; Chinta, SJ; Brochier, C; Langley, B et al, ANTIOXIDANTS & REDOX SIGNALING, 22, 2, 121-134, DOI: 10.1089/ars.2013.5595, JAN 10 2015
Oxycodon	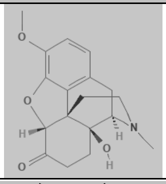	Current and experimental treatments of Parkinson disease: A guide for neuroscientists. Oertel, W; Schulz, JB. JOURNAL OF NEUROCHEMISTRY, 139, 325-337, Supplement: 1, DOI: 10.1111/jnc.13750, OCT 2016
Oxyphylla A = (R)-4-(2-hydroxy-5-methylpheny1)-5-methylhexanoic acid	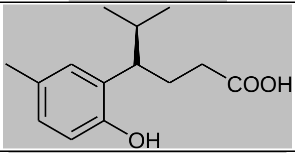	Discovery, Synthesis, and Functional Characterization of a Novel Neuroprotective Natural Product from the Fruit of Alpinia oxyphylla for use in Parkinson's Disease Through LC/MS-Based Multivariate Data Analysis-Guided Fractionation, Li, GH; Zhang, ZJ; Quan, Q; Jiang, RW; Szeto, SSW; Yuan, S; Wong, WT; Lam, HHC; Lee, SMY; Chu, IK, JOURNAL OF PROTEOME RESEARCH,15, 8, 2595-2606, DOI: 10.1021/acs.jproteome.6b00152, AUG 2016
Oxysterols	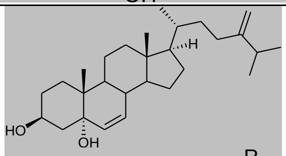 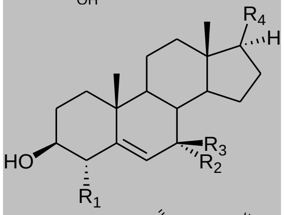 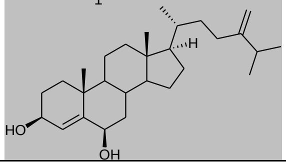	Oxysterols from a Marine Sponge Inflatella sp and Their Action in 6-Hydroxydopamine-Induced Cell Model of Parkinson's Disease, Kolesnikova, SA; Lyakhova, EG; Kalinovsky, AI; Popov, RS; Yurchenko, EA; Stonik, VA, MARINE DRUGS,16, 11, Article Number: 458, DOI: 10.3390/md16110458, NOV 2018
p-BTX-I (Glu-Val-Trp)		A synthetic snake-venom-based tripeptide protects PC12 cells from MPP+ toxicity by activating the NGF-signaling pathway, Bernardes, CP; Santos, NAG; Sisti, FM; Ferreira, RS; Santos, NA; Cintra, ACO; Cilli, EM; Sampaio, SV; Santos, AC, PEPTIDES,104, 24-34, DOI: 10.1016/j.peptides.2018.04.012, JUN 2018
P7C3	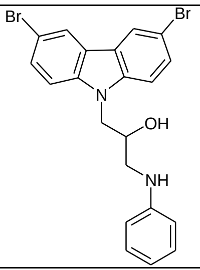	**1)** P7C3 Inhibits LPS-Induced Microglial Activation to Protect Dopaminergic Neurons Against Inflammatory Factor-Induced Cell Death in vitro and in vivo, Gu, C; Hu, QS; Wu, JY; Mu, CC; Ren, HG; Liu, CF; Wang, GH, FRONTIERS IN CELLULAR NEUROSCIENCE,12, Article Number: 400, DOI: 10.3389/fncel.2018.00400, NOV 5 2018, **2)** Protective efficacy of P7C3-S243 in the 6-hydroxydopamine model of Parkinson's disease, De Jesus-Cortest, H; Miller, AD; Britt, JK; DeMarco, AJ; De Jesus-Cortes, M; Stuebing, E; Naidoo, J; Vazquez-Rosa, E; Morlock, L; Williams, NS et al, NPJ PARKINSONS DISEASE,1, Article Number: 15010, DOI: 10.1038/npjparkd.2015.10, 2015

Paeoniflorin	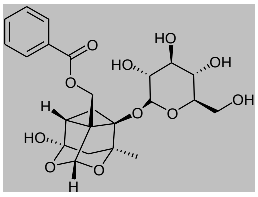	**1)** Recent advances in discovery and development of natural products as source for anti-Parkinson's disease lead compounds, Zhang, HJ; Bai, L; He, J; Zhong, L; Duan, XM; Ouyang, L; Zhu, YX; Wang, T; Shi, JY; Zhang, YW, EUROPEAN JOURNAL OF MEDICINAL CHEMISTRY,141, 257-272, DOI: 10.1016/j.ejmech.2017.09.068, DEC 1 2017, **Document Type:Review, 2)** Synergistic protective effect of paeoniflorin and beta-ecdysterone against rotenone-induced neurotoxicity in PC12 cells, Liu, H; Yu, CL; Xu, TJ; Zhang, XJ; Dong, MX, APOPTOSIS,21, 12, 1354-1365, DOI: 10.1007/s10495-016-1293-7, DEC 2016
Paeonol	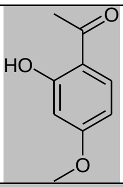	**1)** Pharmacokinetics and Brain Distribution of the Active Components of DA-9805, Saikosaponin A, Paeonol and Imperatorin in Rats, Kwon, MH; Jeong, JS; Ryu, J; Cho, YW; Kang, HE, PHARMACEUTICS,10, 3, Article Number: 133, DOI: 10.3390/pharmaceutics10030133, SEP 2018, **2)** Role of Paeonol in an Astrocyte Model of Parkinson's Disease, Ye, MS; Yi, YX; Wu, SX; Zhou, Y; Zhao, DJ, MEDICAL SCIENCE MONITOR,23, 4740-4748, DOI: 10.12659/MSM.906716, OCT 3 2017
Paliroden	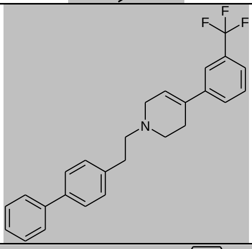	A review on iron chelators as potential therapeutic agents for the treatment of Alzheimer's and Parkinson's diseases. Singh, YP; Pandey, A; Vishwakarma, S; Modi, G, Molecular diversity, DOI:10.1007/s11030-018-9878-4, 2018-Oct-06 (Epub 2018 Oct 06). **Document type: review**
Panaxatriol saponins	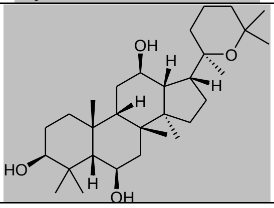	Recent advances in discovery and development of natural products as source for anti-Parkinson's disease lead compounds, Zhang, HJ; Bai, L; He, J; Zhong, L; Duan, XM; Ouyang, L; Zhu, YX; Wang, T; Shi, JY; Zhang, YW, EUROPEAN JOURNAL OF MEDICINAL CHEMISTRY,141, 257-272, DOI: 10.1016/j.ejmech.2017.09.068, DEC 1 2017, **Document Type:Review**
Pantethine	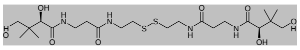	Pantethine Alters Lipid Composition and Cholesterol Content of Membrane Rafts, With Down-Regulation of CXCL12-Induced T Cell Migration, Van Gijsel-Bonnello, M; Acar, N; Molino, Y; Bretillon, L; Khrestchatisky, M; De Reggi, M; Gharib, B, JOURNAL OF CELLULAR PHYSIOLOGY,230, 10, 2415-2425, DOI: 10.1002/jcp.24971, OCT 2015
Paoniflorin	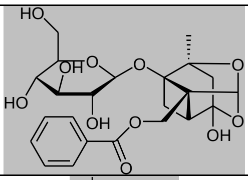	Application of Monoterpenoids and their Derivatives for Treatment of Neurodegenerative Disorders, Volcho, KP; Laev, SS; Ashraf, GM; Aliev, G; Salakhutdinov, NF, CURRENT MEDICINAL CHEMISTRY,25, 39, 5327-5346, DOI: 10.2174/0929867324666170112101837, 2018, **Document Type:Review**
Pardoprunox	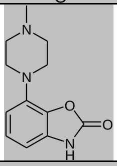	Dual Ligands Targeting Dopamine D-2 and Serotonin 5-HT1A Receptors as New Antipsychotical or Anti-Parkinsonian Agents, Ye, N; Song, ZL; Zhang, A, CURRENT MEDICINAL CHEMISTRY,21, 4, 437-457, FEB 2014, **Document Type:Review**
Paroxetine	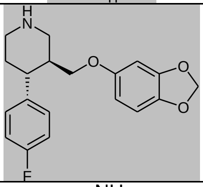	Repurposing of Copper -chelating Drugs for the Treatment of Neurodegenerative Diseases. Lanza, V; Milardi, D; Di Natale, G; Pappalardo, G. CURRENT MEDICINAL CHEMISTRY, 25, 4, 525-539, DOI: 10.2174/0929867324666170518094404, 2018, **Document Type: Review**
PBF-509	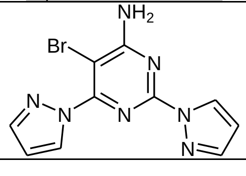	Adenosine A Receptor Antagonists in Parkinson's Disease: Progress in Clinical Trials from the Newly Approved Istradefylline to Drugs in Early Development and Those Already Discontinued, Pinna, A, CNS DRUGS,28, 5, 455-474, DOI: 10.1007/s40263-014-0161-7, MAY 2014, **Document Type: Review**
PBT2	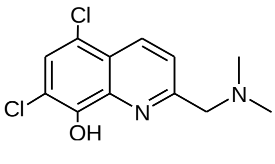	**1)** A review on iron chelators as potential therapeutic agents for the treatment of Alzheimer's and Parkinson's diseases. Singh, YP; Pandey, A; Vishwakarma, S; Modi, G, Molecular diversity, DOI:10.1007/s11030-018-9878-4, 2018-Oct-06. **Document type: review, 2)** Rapid restoration of cognition in alzheimer's transgenic mice with 8-hydroxy quinoline analogs is associated with decreased interstitial abeta. Adlard, P.A., Cherny, R.A., Finkelstein, D.I., Gautier, E., Robb, E., Cortes, M., Volitakis, I., Liu, X., Smith, J.P., Perez, K., Laughton, K., Li, Q.X., Charman, S.A., Nicolazzo, J.A., Wilkins, S., Deleva, K., Lynch, T., Kok, G., Ritchie, C.W., Tanzi, R.E., Cappai, R., Masters, C.L., Barnham, K.J., Bush, A.I. Neuron, 59, 43-55, 2008.

PBT434	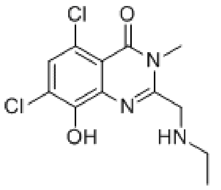	The novel compound PBT434 prevents iron mediated neurodegeneration and alpha-synuclein toxicity in multiple models of Parkinson's disease. Finkelstein, DI; Billings, JL; Adlard, PA; Ayton, S; Sedjahtera, A; Masters, CL; Wilkins, S; Shackleford, DM; Charman, SA; Bal, W; Zawisza, IA; Kurowska, E; Gundlach, AL; Ma, S; Bush, AI; Hare, DJ; Doble, PA; Crawford, S; Gautier, ECL; Parsons, J; Huggins, P; Barnham, KJ; Cherny, RA. ACTA NEUROPATHOLOGICA COMMUNICATIONS, 5, Article Number: 53, DOI: 10.1186/s40478-017-0456-2, JUN 28 2017
Pelargonidin	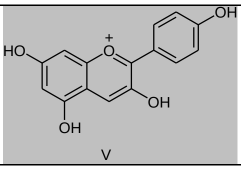 V	Anthocyanins Improve Hippocampus-Dependent Memory Function and Prevent Neurodegeneration via JNK/Akt/GSK3 Signaling in LPS-Treated Adult Mice, Khan, MS; Ali, T; Kim, MW; Jo, MH; Chung, JI; Kim, MO; MOLECULAR NEUROBIOLOGY, 56, 1, 671-687, DOI: 10.1007/s12035-018-1101-1, JAN 2019
Pentamidine	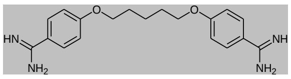	inPentasomes: An innovative nose-to-brain pentamidine delivery blunts MPTP parkinsonism in mice, Rinaldi, F; Seguella, L; Gigli, S; Hanieh, PN; Del Favero, E; Cantu, L; Pesce, M; Sarnelli, G; Marianecci, C; Esposito, G et al, JOURNAL OF CONTROLLED RELEASE,294, 17-26, DOI: 10.1016/j.jconrel.2018.12.007, JAN 28 2019
Peonidin	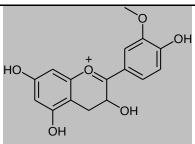 IV	Anthocyanins Improve Hippocampus-Dependent Memory Function and Prevent Neurodegeneration via JNK/Akt/GSK3 Signaling in LPS-Treated Adult Mice, Khan, MS; Ali, T; Kim, MW; Jo, MH; Chung, JI; Kim, MO; MOLECULAR NEUROBIOLOGY, 56, 1, 671-687, DOI: 10.1007/s12035-018-1101-1, JAN 2019
Perampanel	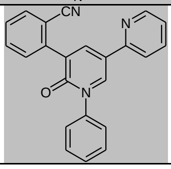	Advances in Drug Development for Parkinson's Disease: Present Status. Reddy, DH; Misra, S; Medhi, B. PHARMACOLOGY, 93, 5-6, 260-271, DOI: 10.1159/000362419, 2014, **Document Type: Review**
Pergolide	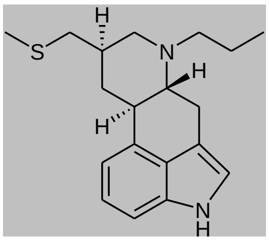	**1)** Prevalence and Correlates of Anti-Parkinson Drug Use in a Nationally Representative Sample, Dahodwala, N; Willis, AW; Li, PX; Doshi, JA, MOVEMENT DISORDERS CLINICAL PRACTICE,4, 3, 335-341, DOI: 10.1002/mdc3.12422, MAY-JUN 2017, **2)** The adenosine A receptor antagonist, istradefylline enhances anti-parkinsonian activity induced by combined treatment with low doses of L-DOPA and dopamine agonists in MPTP-treated common marmosets, Uchida, S; Soshiroda, K; Okita, E; Kawai-Uchida, M; Mori, A; Jenner, P; Kanda, T, EUROPEAN JOURNAL OF PHARMACOLOGY,766, 25-30, DOI: 10.1016/j.ejphar.2015.09.028, NOV 5 2015, **3)** Parkinson's Disease Treatment May Cause Impulse-Control Disorder Via Dopamine D3 Receptors, Seeman, P, SYNAPSE,69, 4, 183-189, DOI: 10.1002/syn.21805, APR 2015, **4)** Advances in non-dopaminergic treatments for Parkinson's disease. Stayte, S; Vissel, B; FRONTIERS IN NEUROSCIENCE, 8, Article Number: 113, DOI: 10.3389/fnins.2014.00113, MAY 22 2014, **Document Type: Review**
Perillyl alcohol	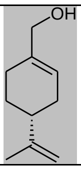	Evaluation of phytomedicinal potential of perillyl alcohol in an in vitro Parkinson's Disease model, Anis, E; Zafeer, MF; Firdaus, F; Islam, SN; Fatima, M; Hossain, MM, DRUG DEVELOPMENT RESEARCH,79, 5, 218-224, DOI: 10.1002/ddr.21436, AUG 2018
Petunidin	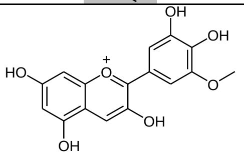 VI	Anthocyanins Improve Hippocampus-Dependent Memory Function and Prevent Neurodegeneration via JNK/Akt/GSK3 Signaling in LPS-Treated Adult Mice, Khan, MS; Ali, T; Kim, MW; Jo, MH; Chung, JI; Kim, MO; MOLECULAR NEUROBIOLOGY, 56, 1, 671-687, DOI: 10.1007/s12035-018-1101-1, JAN 2019
Phenothiazine 2Bc (n > 1)	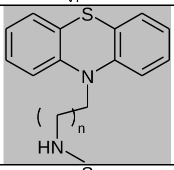	A Focused Library of Psychotropic Analogues with Neuroprotective and Neuroregenerative Potential, Uliassi, E; Pena-Altamira, LE; Morales, AV; Massenzio, F; Petralla, S; Rossi, M; Roberti, M; Gonzalez, LM; Martinez, A; Monti, B et al, ACS CHEMICAL NEUROSCIENCE,10, 1, 279-294, DOI: 10.1021/acschemneuro.8b00242, JAN 2019
Phenothiazine 2Bc (n = 0 and n = 1)	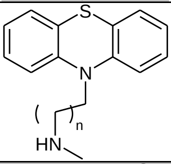	A Focused Library of Psychotropic Analogues with Neuroprotective and Neuroregenerative Potential, Uliassi, E; Pena-Altamira, LE; Morales, AV; Massenzio, F; Petralla, S; Rossi, M; Roberti, M; Gonzalez, LM; Martinez, A; Monti, B et al, ACS CHEMICAL NEUROSCIENCE,10, 1, 279-294, DOI: 10.1021/acschemneuro.8b00242, JAN 2019
N-Phenyl-lactam derivatives	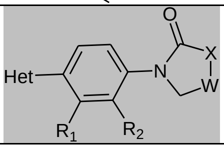	Preparation of N-Phenyl-lactam Derivatives Capable of Stimulating Neurogenesis and Their Use in the Treatment of Neurological Disorders, Blass, B, ACS MEDICINAL CHEMISTRY LETTERS,6, 11, 1092-1094, DOI: 10.1021/acsmedchemlett.5b00355, NOV 2015

1-(Phenyl/4-substituted phenyl)-3-(2-furanyl/thienyl)-5-aryl-2-pyrazolines (3a-i, 4a-i)	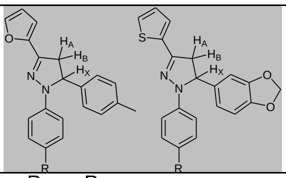	Design, Synthesis, and Neuroprotective Effects of a Series of Pyrazolines against 6-Hydroxydopamine-Induced Oxidative Stress, Ozdemir, A; Sever, B; Altintop, MD; Tilki, EK; Dikmen, M, MOLECULES,23, 9, Article Number: 2151, DOI: 10.3390/molecules23092151, SEP 2018
Phenylhydroxamates	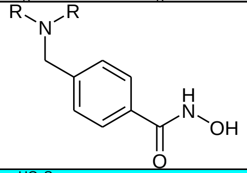	Activation of Nrf2 and Hypoxic Adaptive Response Contribute to Neuroprotection Elicited by Phenylhydroxamic Acid Selective HDAC6 Inhibitors, Gaisina, IN; Lee, SH; Kaidery, NA; Ben Aissa, M; Ahuja, M; Smirnova, NN; Wakade, S; Gaisin, A; Bourassa, MW; Ratan, RR et al, ACS CHEMICAL NEUROSCIENCE,9, 5, 894-900, DOI: 10.1021/acschemneuro.7b00435, MAY 2018
Phthalocyanines	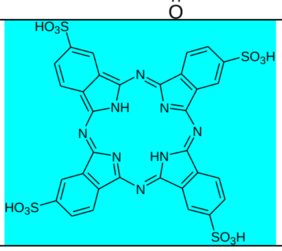	**1)** Development of Multifunctional Molecules as Potential Therapeutic Candidates for Alzheimer's Disease, Parkinson's Disease, and Amyotrophic Lateral Sclerosis in the Last Decade. Savelieff, MG; Nam, G; Kang, J; Lee, HJ; Lee, M; Lim, MH. CHEMICAL REVIEWS, 119, 2, 1221-1322, DOI: 10.1021/acs.chemrev.8b00138, JAN 23 2019, **Document Type: Review, 2)** Phthalocyanines as Molecular Scaffolds to Block Disease-Associated Protein Aggregation, Valiente-Gabioud, AA; Miotto, MC; Chesta, ME; Lombardo, V; Binolfi, A; Fernandez, CO, ACCOUNTS OF CHEMICAL RESEARCH,49, 5, 801-808, DOI: 10.1021/acs.accounts.5b00507, MAY 2016, **Document Type: Review**
Piceatannol	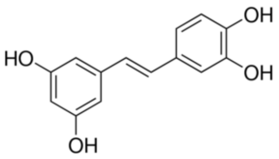	**1)** Natural polyphenols effects on protein aggregates in Alzheimer's and Parkinson's prion-like diseases, Freyssin, A; Page, G; Fauconneau, B; Bilan, AR, NEURAL REGENERATION RESEARCH,13, 6, 955-961, DOI: 10.4103/1673-5374.233432, JUN 2018, **Document Type:Review**, **2)** Polyphenols in Parkinson's Disease: A Systematic Review of In Vivo Studies, Kujawska, M; Jodynis-Liebert, J, NUTRIENTS,10, 5, Article Number: 642, DOI: 10.3390/nu10050642, MAY 2018, **Document Type:Review**, **3)** Novel tactics for neuroprotection in Parkinson's disease: Role of antibiotics, polyphenols and neuropeptides, Reglodi, D; Renaud, J; Tamas, A; Tizabi, Y; Socias, SB; Del-Bel, E; Raisman-Vozari, R, PROGRESS IN NEUROBIOLOGY,155, 120-148 Special : SI, DOI: 10.1016/j.pneurobio.2015.10.004, AUG 2017, **Document Type:Review**, **4) et cetera**
Piceid	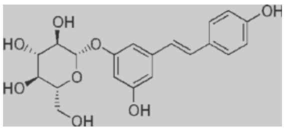	**1)** Natural polyphenols effects on protein aggregates in Alzheimer's and Parkinson's prion-like diseases, Freyssin, A; Page, G; Fauconneau, B; Bilan, AR, NEURAL REGENERATION RESEARCH,13, 6, 955-961, DOI: 10.4103/1673-5374.233432, JUN 2018, **Document Type:Review**, **2)** Polyphenols in Parkinson's Disease: A Systematic Review of In Vivo Studies, Kujawska, M; Jodynis-Liebert, J, NUTRIENTS,10, 5, Article Number: 642, DOI: 10.3390/nu10050642, MAY 2018, **Document Type:Review**, **3)** Novel tactics for neuroprotection in Parkinson's disease: Role of antibiotics, polyphenols and neuropeptides, Reglodi, D; Renaud, J; Tamas, A; Tizabi, Y; Socias, SB; Del-Bel, E; Raisman-Vozari, R, PROGRESS IN NEUROBIOLOGY,155, 120-148 Special : SI, DOI: 10.1016/j.pneurobio.2015.10.004, AUG 2017, **Document Type:Review**, **4) et cetera**
Pimavanserin	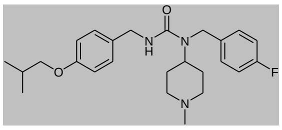	**1)** Current approaches to the treatment of Parkinson's Disease, Ellis, JM; Fell, MJ, BIOORGANIC & MEDICINAL CHEMISTRY LETTERS, 27, 18, 4247-4255, DOI: 10.1016/j.bmcl.2017.07.075, Published: SEP 15 2017**, 2)** On the Discovery and Development of Pimavanserin: A Novel Drug Candidate for Parkinson's Psychosis, Hacksell, U; Burstein, ES; McFarland, K; Mills, RG; Williams, H, NEUROCHEMICAL RESEARCH,39, 10, 2008-2017 Special : SI, DOI: 10.1007/s11064-014-1293-3, OCT 2014
α-Pinene	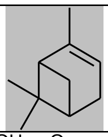	In vitro neuroprotective potential of the monoterpenes alpha-pinene and 1,8-cineole against H2O2-induced oxidative stress in PC12 cells, Porres-Martinez, M; Gonzalez-Burgos, E; Carretero, ME; Gomez-Serranillos, MP, ZEITSCHRIFT FUR NATURFORSCHUNG SECTION C-A JOURNAL OF BIOSCIENCES,71, 7-8, 191-199, DOI: 10.1515/znc-2014-4135, JUL 2016
Pinostrobin	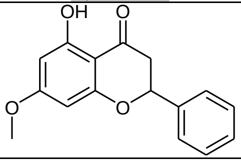	Pinostrobin Exerts Neuroprotective Actions in Neurotoxin-Induced Parkinson's Disease Models through Nrf2 Induction, Li, CW; Tang, BQ; Feng, Y; Tang, F; Hoi, MPM; Su, ZR; Lee, SMY, JOURNAL OF AGRICULTURAL AND FOOD CHEMISTRY,66, 31, 8307-8318, DOI: 10.1021/acs.jafc.8b02607, AUG 8 2018
Piperazine-8-OH-quinolone hybrid	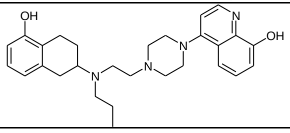	New Perspectives in Iron Chelation Therapy for the Treatment of Neurodegenerative Diseases. Nunez, MT; Chana-Cuevas, P. PHARMACEUTICALS, 11, 4, Article Number: UNSP 109, DOI: 10.3390/ph11040109, DEC 2018, **Document Type: Review**
Piperine	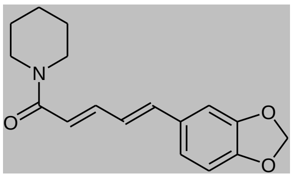	**1)** Piperine in combination with quercetin halt 6-OHDA induced neurodegeneration in experimental rats: Biochemical and neurochemical evidences, Singh, S; Kumar, P, NEUROSCIENCE RESEARCH,133, 38-47, DOI: 10.1016/j.neures.2017.10.006, AUG 2018, **2)** Piperine induces autophagy by enhancing protein phosphotase 2A activity in a rotenone-induced Parkinson's disease model, Liu, J; Chen, M; Wang, X; Wang, Y; Duan, CL; Gao, G; Lu, LL; Wu, X; Wang, XM; Yang, H, ONCOTARGET,7, 38, 60823-60843, DOI: 10.18632/oncotarget.11661, SEP 20 2016 **3)** Delivery of Dual Drug Loaded Lipid Based Nanoparticles across the Blood-Brain Barrier Impart Enhanced

		Neuroprotection in a Rotenone Induced Mouse Model of Parkinson's Disease, Kundu, P; Das, M; Tripathy, K; Sahoo, SK, ACS CHEMICAL NEUROSCIENCE,7, 12, 1658-1670, DOI: 10.1021/acschemneuro.6b00207, DEC 2016
Piperlongumine	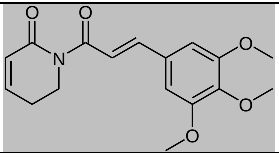	Piperlongumine inhibits neuroinflammation via regulating NF-kappa B signaling pathways in lipopolysaccharide-stimulated BV2 microglia cells, Kim, N; Do, J; Bae, JS; Jin, HK; Kim, JH; Inn, KS; Oh, MS; Lee, JK, JOURNAL OF PHARMACOLOGICAL SCIENCES,137, 2, 195-201, DOI: 10.1016/j.jphs.2018.06.004, JUN 2018
Piribedil	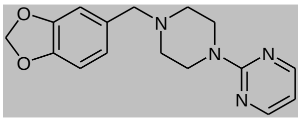	**1)** Development and validation of rapid and sensitive LC methods with PDA and fluorescence detection for determination of piribedil in rat plasma and brain ts and their pharmacokinetic application, Uppuluri, CT; Dalvi, AV; Bommireddy, EP; Ravi, PR, BIOMEDICAL CHROMATOGRAPHY,32, 10, Article Number: e4303, DOI: 10.1002/bmc.4303, OCT 2018, **2)** Prevalence and Correlates of Anti-Parkinson Drug Use in a Nationally Representative Sample, Dahodwala, N; Willis, AW; Li, PX; Doshi, JA, MOVEMENT DISORDERS CLINICAL PRACTICE,4, 3, 335-341, DOI: 10.1002/mdc3.12422, MAY-JUN 2017 2) Advances in non-dopaminergic treatments for Parkinson's disease. Stayte, S; Vissel, B; FRONTIERS IN NEUROSCIENCE, 8, Article Number: 113, DOI: 10.3389/fnins.2014.00113, MAY 22 2014, **Document Type: Review**
Piroxicam	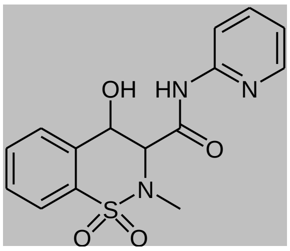	**1)** Oxicam-derived non-steroidal anti-inflammatory drugs suppress 1-methyl-4-phenyl pyridinium-induced cell death via repression of endoplasmic reticulum stress response and mitochondrial dysfunction in SH-SY5Y cells, Omura, T; Sasaoka, M; Hashimoto, G; Imai, S; Yamamoto, J; Sato, Y; Nakagawa, S; Yonezawa, A; Nakagawa, T; Yano, I et al, BIOCHEMICAL AND BIOPHYSICAL RESEARCH COMMUNICATIONS,503, 4, 2963-2969, DOI: 10.1016/j.bbrc.2018.08.078, SEP 18 2018, **2)** Ibuprofen or piroxicam protects nigral neurons and delays the development of L-dopa induced dyskinesia in rats with experimental Parkinsonism: Influence on angiogenesis, Teema, AM; Zaitone, SA; Moustafa, YM, NEUROPHARMACOLOGY,107, 432-450, DOI: 10.1016/j.neuropharm.2016.03.034, AUG 2016
Pituitary adenylate cyclase-activating polypeptide (PACAP)		**1)** New insights about the peculiar role of the 28-38 C-terminal segment and some selected residues in PACAP for signaling and neuroprotection, de Molliens, MP; Letourneau, M; Devost, D; Hebert, TE; Fournier, A; Chatenet, D, BIOCHEMICAL PHARMACOLOGY,154, 193-202, DOI: 10.1016/j.bcp.2018.04.024, AUG 2018, **2)** Novel tactics for neuroprotection in Parkinson's disease: Role of antibiotics, polyphenols and neuropeptides, Reglodi, D; Renaud, J; Tamas, A; Tizabi, Y; Socias, SB; Del-Bel, E; Raisman-Vozari, R, PROGRESS IN NEUROBIOLOGY,155, 120-148 Special : SI, DOI: 10.1016/j.pneurobio.2015.10.004, AUG 2017, **Document Type:Review**, **3) et cetera**
Pitolisant	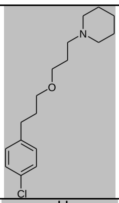	Current and experimental treatments of Parkinson disease: A guide for neuroscientists. Oertel, W; Schulz, JB. JOURNAL OF NEUROCHEMISTRY, 139, 325-337, Supplement: 1, DOI: 10.1111/jnc.13750, OCT 2016, **Document Type: Review**
Pioglitazone	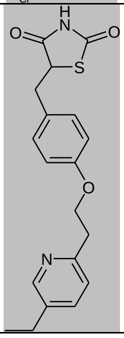	**1)** Pioglitazone use and Parkinson's disease: a retrospective cohort study in Taiwan, Wu, HF; Kao, LT; Shih, JH; Kao, HH; Chou, YC; Li, IH; Kao, SY, BMJ OPEN,8, 8, Article Number: e023302, DOI: 10.1136/bmjopen-2018-023302, AUG 2018, **2)** Pioglitazone reduces mortality, prevents depressive-like behavior, and impacts hippocampal neurogenesis in the 6-OHDA model of Parkinson's disease in rats, Bonato, JM; Bassani, TB; Milani, H; Vital, MABF (Barbato Frazdo Vital, Maria Aparecida); de Oliveira, RMW, EXPERIMENTAL NEUROLOGY,300, 188-200, DOI: 10.1016/j.expneurol.2017.11.009, FEB 2018, **3)** Disease-Modifying Drugs in Parkinson's Disease, Park, A; Stacy, M, DRUGS,75, 18, 2065-2071, DOI: 10.1007/s40265-015-0497-4, DEC 2015, **Document Type:Review**, **4)** The ongoing pursuit of neuroprotective therapies in Parkinson disease, Athauda, D; Foltynie, T, NATURE REVIEWS NEUROLOGY,11, 1, 25-40, DOI: 10.1038/nrneurol.2014.226, JAN 2015, **Document Type:Review**
Plasmid DNA		Chemotherapeutic Drug-Conjugated Microbeads Demonstrate Preferential Binding to Methylated Plasmid DNA, Lin, KN; Grandhi, TSP; Goklany, S; Rege, K, BIOTECHNOLOGY JOURNAL,13, 11, Article Number: 1700701, DOI: 10.1002/biot.201700701, NOV 2018
Polycyclic propargylamine - acetylene	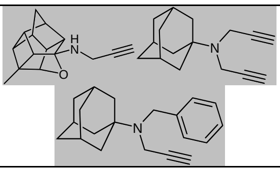	Polycyclic propargylamine and acetylene derivatives as multifunctional neuroprotective agents, Zindo, FT; Barber, QR; Joubert, J; Bergh, JJ; Petzer, JP; Malan, SF, EUROPEAN JOURNAL OF MEDICINAL CHEMISTRY,80, 122-134, DOI: 10.1016/j.ejmech.2014.04.039, JUN 10 2014

Polynorbornene gluconamide	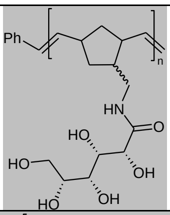	A generic class of amyloid fibril inhibitors, Ow, SY; Bekard, I; Blencowe, A; Qiao, GG; Dunstan, DE, JOURNAL OF MATERIALS CHEMISTRY B,3, 7, 1350-1359, DOI: 10.1039/c4tb01762e, 2015
Polynorbornene glucose ester	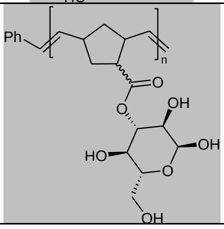	A generic class of amyloid fibril inhibitors, Ow, SY; Bekard, I; Blencowe, A; Qiao, GG; Dunstan, DE, JOURNAL OF MATERIALS CHEMISTRY B,3, 7, 1350-1359, DOI: 10.1039/c4tb01762e, 2015
Polysulfides	RSSnH, RSSnSR	Polysulfide protects midbrain dopaminergic neurons from MPP+-induced degeneration via enhancement of glutathione biosynthesis, Takahashi, S; Hisatsune, A; Kurauchi, Y; Seki, T; Katsuki, H, JOURNAL OF PHARMACOLOGICAL SCIENCES,137, 1, 47-54, DOI: 10.1016/j.jphs.2018.04.004, MAY 2018
Pramipexole	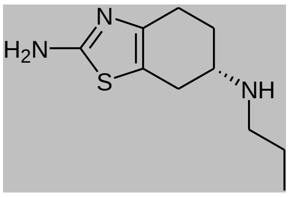	**1)** Pramipexole dihydrochloride loaded chitosan nanoparticles for nose to brain delivery: Development, characterization and in vivo anti-Parkinson activity, Raj, R; Wairkar, S; Sridhar, V; Gaud, R, INTERNATIONAL JOURNAL OF BIOLOGICAL MACROMOLECULES,109, 27-35, DOI: 10.1016/j.ijbiomac.2017.12.056, APR 2018, **2)** A Sensitive Voltammetric Determination of Anti-Parkinson Drug Pramipexole Using Titanium Dioxide Nanoparticles Modified Carbon Paste Electrode, Hassaninejad-Darzi, SK; Shajie, F, JOURNAL OF THE BRAZILIAN CHEMICAL SOCIETY,28, 4, 529-539, DOI: 10.5935/0103-5053.20160192, APR 2017, **3)** A Comparative Pharmacokinetics Study of the Anti-Parkinsonian Drug Pramipexole, Putri, RSI; Setiawati, E; Aziswan, SA; Ong, F; Tjandrawinata, RR; Susanto, LW, SCIENTIA PHARMACEUTICA,84, 4, 715-723, DOI: 10.3390/scipharm84040715, DEC 2016, **4)** Prevalence and Correlates of Anti-Parkinson Drug Use in a Nationally Representative Sample, Dahodwala, N; Willis, AW; Li, PX; Doshi, JA, MOVEMENT DISORDERS CLINICAL PRACTICE,4, 3, 335-341, DOI: 10.1002/mdc3.12422, MAY-JUN 2017 **5)** Advances in non-dopaminergic treatments for Parkinson's disease. Stayte, S; Vissel, B; FRONTIERS IN NEUROSCIENCE, 8, Article Number: 113, DOI: 10.3389/fnins.2014.00113, MAY 22 2014, **Document Type: Review 6) et cetera**
Pravastatin	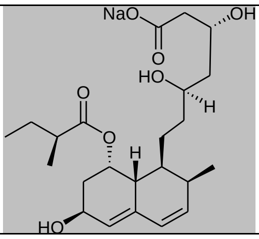	**1)** The beneficial effects of HMG-CoA reductase inhibitors in the processes of neurodegeneration, Saravi, SSS; Saravi, SSS; Arefidoust, A; Dehpour, AR, METABOLIC BRAIN DISEASE,32, 4, 949-965, DOI: 10.1007/s11011-017-0021-5, AUG 2017, **Document Type:Review**, **2)** Neuroprotective mechanisms of statins in neurodegenerative diseases, Yan, JQ; Sun, JC; Li, XM; Zhai, MM; Jia, XF, INTERNATIONAL JOURNAL OF CLINICAL AND EXPERIMENTAL MEDICINE,9, 6, 9799-9805, 2016, **Document Type:Review**
Preladenant	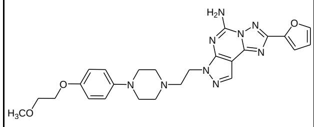	**1)** Current approaches to the treatment of Parkinson's Disease, Ellis, JM; Fell, MJ, BIOORGANIC & MEDICINAL CHEMISTRY LETTERS, 27, 18, 4247-4255, DOI: 10.1016/j.bmcl.2017.07.075, Published: SEP 15 2017, **2)** A preclinical study on the combined effects of repeated eltoprazine and preladenant treatment for alleviating L-DOPA-induced dyskinesia in Parkinson's disease, Ko, WKD; Li, Q; Cheng, LY; Morelli, M; Carta, M; Bezard, E, EUROPEAN JOURNAL OF PHARMACOLOGY,813, 10-16, DOI: 10.1016/j.ejphar.2017.07.030, OCT 15 2017, **3)** Adenosine A Receptor Antagonists in Parkinson's Disease: Progress in Clinical Trials from the Newly Approved Istradefylline to Drugs in Early Development and Those Already Discontinued, Pinna, A, CNS DRUGS,28, 5, 455-474, DOI: 10.1007/s40263-014-0161-7, MAY 2014, **Document Type:Review**
Pridopidine	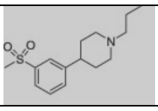	Current approaches to the treatment of Parkinson's Disease, Ellis, JM; Fell, MJ, BIOORGANIC & MEDICINAL CHEMISTRY LETTERS, 27, 18, 4247-4255, DOI: 10.1016/j.bmcl.2017.07.075, Published: SEP 15 2017
Proanthocyanidins	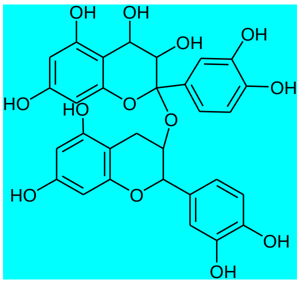	**1)** Proanthocyanidins exert a neuroprotective effect via ROS/JNK signaling in MPTP-induced Parkinson's disease models in vitro and in vivo, Chen, HC; Xu, JY; Lv, Y; He, P; Liu, CY; Jiao, J; Li, SW; Mao, XH; Xue, X, MOLECULAR MEDICINE REPORTS,18, 6, 4913-4921, DOI: 10.3892/mmr.2018.9509, DEC 2018, **2)** Natural polyphenols effects on protein aggregates in Alzheimer's and Parkinson's prion-like diseases, Freyssin, A; Page, G; Fauconneau, B; Bilan, AR, NEURAL REGENERATION RESEARCH,13, 6, 955-961, DOI: 10.4103/1673-5374.233432, JUN 2018, **Document Type:Review**, **3)** Polyphenols in Parkinson's Disease: A Systematic Review of In Vivo Studies, Kujawska, M; Jodynis-Liebert, J, NUTRIENTS,10, 5, Article Number: 642, DOI: 10.3390/nu10050642, MAY 2018, **Document Type:Review**, **4)** Novel tactics for neuroprotection in Parkinson's disease: Role of antibiotics, polyphenols and

		neuropeptides, Reglodi, D; Renaud, J; Tamas, A; Tizabi, Y; Socias, SB; Del-Bel, E; Raisman-Vozari, R, PROGRESS IN NEUROBIOLOGY,155, 120-148 Special : SI, DOI: 10.1016/j.pneurobio.2015.10.004, AUG 2017, **Document Type:Review**, **5) et cetera**
Procyclidine	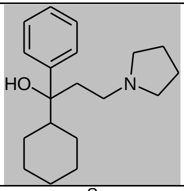	**1)** Prevalence and Correlates of Anti-Parkinson Drug Use in a Nationally Representative Sample, Dahodwala, N; Willis, AW; Li, PX; Doshi, JA; MOVEMENT DISORDERS CLINICAL PRACTICE, 4, 3, 335-341, DOI: 10.1002/mdc3.12422, MAY-JUN 2017, **2)** Advances in non-dopaminergic treatments for Parkinson's disease. Stayte, S; Vissel, B; FRONTIERS IN NEUROSCIENCE, 8, Article Number: 113, DOI: 10.3389/fnins.2014.00113, MAY 22 2014, **Document Type: Review**
Promethazine	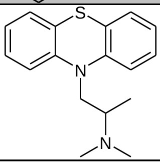	Deciphering the enhanced inhibitory, disaggregating and cytoprotective potential of promethazine towards amyloid fibrillation, Nusrat, S; Zaman, M; Masroor, A; Siddqi, MK; Zaidi, N; Neelofar, K; Abdelhameed, AS; Khan, RH, INTERNATIONAL JOURNAL OF BIOLOGICAL MACROMOLECULES,106, 851-863, DOI: 10.1016/j.ijbiomac.2017.08.081, JAN 2018
Propargyl-substituted pyrimidine derivatives	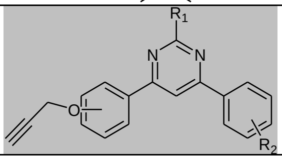	Synthesis, Biological Evaluation and Molecular Modeling Studies of Propargyl-Containing 2,4,6-Trisubstituted Pyrimidine Derivatives as Potential Anti-Parkinson Agents, Kumar, B; Kumar, M; Dwivedi, AR; Kumar, V, CHEMMEDCHEM,13, 7, 705-712, DOI: 10.1002/cmdc.201700589, APR 6 2018
Propofol	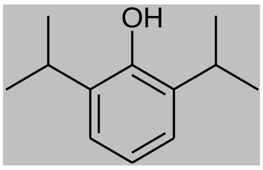	**1)** Effects of Propofol on Oxidative Stress Parameters in Selected Parts of the Brain in a Rat Model of Parkinson Disease, Romuk, E; Szczurek, W; Nowak, P; Skowron, M; Prudel, B; Hudziec, E; Chwalinska, E; Birkner, E, POSTEPY HIGIENY I MEDYCYNY DOSWIADCZALNEJ,70, 1441-1450, DOI: 10.5604/17322693.1227841, DEC 31 2016, **2)** Propofol protects against the neurotoxicity of 1-methy1-4-phenylpyridinium, Wang, SS; Song, TT; Leng, CB; Lan, KT; Ning, JS; Chu, HC, MOLECULAR MEDICINE REPORTS,13, 1, 309-314 Part: A, DOI: 10.3892/mmr.2015.4570, JAN 2016
Prostratin	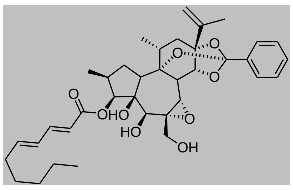	**1)** Daphnane and Phorbol Diterpenes, Anti-neuroinflammatory Compounds with Nurr1 Activation from the Roots and Stems of Daphne genkwa, Han, BS; Van Minh, N; Choi, HY; Byun, JS; Kim, WG, BIOLOGICAL & PHARMACEUTICAL BULLETIN,40, 12, 2205-2212, DEC 2017, **2)** Diterpenes: Advances in Neurobiological Drug Research, Islam, MT; da Silva, CB; de Alencar, MVOB; Paz, MFCJ; Almeida, FRD; Melo-Cavalcante, AAD, PHYTOTHERAPY RESEARCH,30, 6, 915-928, DOI: 10.1002/ptr.5609, JUN 2016, **Document Type: Review**
Protocatechuic acid	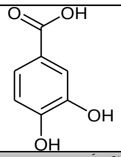	Examining the neuroprotective effects of protocatechuic acid and chrysin on in vitro and in vivo models of Parkinson disease, Zhang, ZJ; Li, GH; Szeto, SSW; Chong, CM; Quan, Q; Huang, C; Cui, W; Guo, BJ; Wang, YQ; Han, YF et al, FREE RADICAL BIOLOGY AND MEDICINE,84, 331-343, DOI: 10.1016/j.freeradbiomed.2015.02.030, JUL 2015
Protodioscin	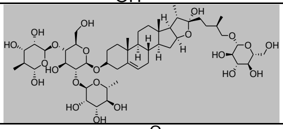	Neuroprotective action and mechanistic evaluation of protodioscin against rat model of Parkinson's disease, Wu, J; Zhao, YM; Deng, ZK, PHARMACOLOGICAL REPORTS,70, 1, 139-145, DOI: 10.1016/j.pharep.2017.08.013, FEB 2018
Protosappanin A	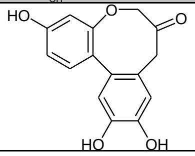	Protosappanin A exerts anti-neuroinflammatory effect by inhibiting JAK2-STAT3 pathway in lipopolysaccharide-induced BV2 microglia, Wang, LC; Liao, LX; Zhao, MB; Dong, X; Zeng, KW; Tu, PF, CHINESE JOURNAL OF NATURAL MEDICINES,15, 9, 674-679, SEP 2017
PRX002/RG7935		**1)** Safety and Tolerability of Multiple Ascending Doses of PRX002/RG7935, an Anti-a-Synuclein Monoclonal Antibody, in Patients With Parkinson Disease A Randomized Clinical Trial, Jankovic, J; Goodman, I; Safirstein, B; Marmon, TK; Schenk, DB; Koller, M; Zago, W; Ness, DK; Griffith, SG; Grundman, M et al, JAMA NEUROLOGY,75, 10, 1206-1214, DOI: 10.1001/jamaneurol.2018.1487, OCT 2018, **2)** First-in-human assessment of PRX002, an anti--synuclein monoclonal antibody, in healthy volunteers, Schenk, DB; Koller, M; Ness, DK; Griffith, SG; Grundman, M; Zago, W; Soto, J; Atiee, G; Ostrowitzki, S; Kinney, GG, MOVEMENT DISORDERS,32, 2, 211-218, DOI: 10.1002/mds.26878, FEB 2017, **3)** Disease-Modifying Drugs in Parkinson's Disease, Park, A; Stacy, M, DRUGS,75, 18, 2065-2071, DOI: 10.1007/s40265-015-0497-4, DEC 2015, **Document Type:Review, 4) et cetera**
PTUPB	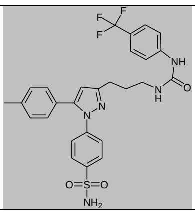	Evaluation of antiparkinson activity of PTUPB by measuring dopamine and its metabolites in Drosophila melanogaster: LC-MS/MS method development, Lakkappa, N; Krishnamurthy, PT; Yamjala, K; Hwang, SH; Hammock, BD; Babu, B, JOURNAL OF PHARMACEUTICAL AND BIOMEDICAL ANALYSIS,149, 457-464, DOI: 10.1016/j.jpba.2017.11.043, FEB 5 2018

Puerarin	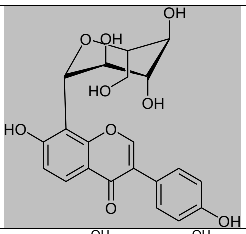	**1)** Puerarin promoted proliferation and differentiation of dopamine-producing cells in Parkinson's animal models, Liu, SY; Qu, XH; Jin, GH; Nie, F; Liu, F; Chen, SM; Hu, F, BIOMEDICINE & PHARMACOTHERAPY,106, 1236-1242, DOI: 10.1016/j.biopha.2018.07.058, OCT 2018, **2)** Recent advances in discovery and development of natural products as source for anti-Parkinson's disease lead compounds, Zhang, HJ; Bai, L; He, J; Zhong, L; Duan, XM; Ouyang, L; Zhu, YX; Wang, T; Shi, JY; Zhang, YW, EUROPEAN JOURNAL OF MEDICINAL CHEMISTRY,141, 257-272, DOI: 10.1016/j.ejmech.2017.09.068, DEC 1 2017, **Document Type:Review**
Punicalagin	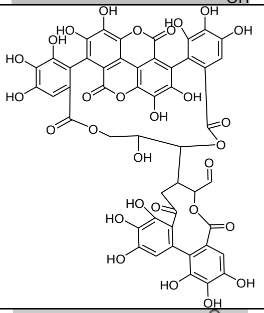	**1)** Punicalagin Exerts Beneficial Functions in 6-Hydroxydopamine-Treated SH-SY5Y Cells by Attenuating Mitochondrial Dysfunction and Inflammatory Responses, Chu, JF; Han, W, MEDICAL SCIENCE MONITOR,24, S905-S913, DOI: 10.12659/MSM.909969, AUG 25 2018, **2)** Exploring the Structural Diversity in Inhibitors of alpha-Synuclein Amyloidogenic Folding, Aggregation, and Neurotoxicity, Das, S; Pukala, TL; Smid, SD, FRONTIERS IN CHEMISTRY,6, Article Number: 181, DOI: 10.3389/fchem.2018.00181, MAY 25 2018
16,17-Pyrazolinyl DHEA analogues	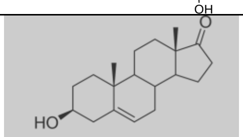	Studies on 16,17-Pyrazoline Substituted Heterosteroids as Anti-Alzheimer and Anti-Parkinsonian Agents Using LPS Induced Neuroinflammation Models of Mice and Rats, Singh, R; Thota, S; Bansal, R, ACS CHEMICAL NEUROSCIENCE,9, 2, 272-283, DOI: 10.1021/acschemneuro.7b00303, FEB 2018
Pyrazolobenzothiazine-based carbothioamides	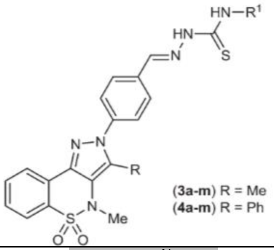	Pyrazolobenzothiazine-based carbothioamides as new structural leads for the inhibition of monoamine oxidases: design, synthesis, in vitro bioevaluation and molecular docking studies, Abid, SMA; Aslam, S; Zaib, S; Bakht, SM; Ahmad, M; Athar, MM; Gardiner, JM; Iqbal, J, MEDCHEMCOMM,8, 2, 452-464, DOI: 10.1039/c6md00570e, FEB 1 2017
4-Pyridinylmethy1-9(10H)-anthracenone (XE991)	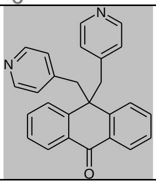	The Kv7/KCNQ channel blocker XE991 protects nigral dopaminergic neurons in the 6-hydroxydopamine rat model of Parkinson's disease, Liu, HX; Jia, L; Chen, XY; Shi, LM; Xie, JX, BRAIN RESEARCH BULLETIN,137, 132-139, DOI: 10.1016/j.brainresbull.2017.11.011, MAR 2018
Pyridoxal isonicotinoyl hydrazone (PIH) and related compounds	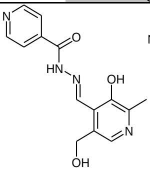 PIH 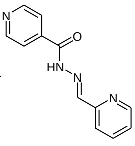 PCIH<br>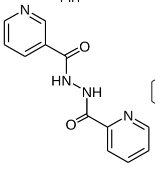 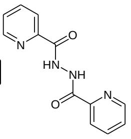<br>H2NPH<br>H2PPH<br>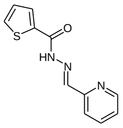<br>PCTH	**1)** A review on iron chelators as potential therapeutic agents for the treatment of Alzheimer's and Parkinson's diseases. Singh, YP; Pandey, A; Vishwakarma, S; Modi, G, Molecular diversity, DOI:10.1007/s11030-018-9878-4, 2018-Oct-06. **Document type: review**, **2)** Iron Chelating Strategies in Systemic Metal Overload, Neurodegeneration and Cancer. Gumienna-Kontecka, E; Pyrkosz-Bulska, M; Szebesczyk, A; Ostrowska, M. CURRENT MEDICINAL CHEMISTRY, 21, 33, 3741-3767, DOI: 10.2174/09298673216661407061434 02, 2014. **Document Type: Review**
Pyrimidinone 8	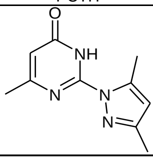	Discovery and characterization of a novel non-competitive inhibitor of the divalent metal transporter DMT1/SLC11A2, Montalbetti, N; Simonin, A; Simonin, C; Awale, M; Reymond, JL; Hediger, MA, BIOCHEMICAL PHARMACOLOGY,96, 3, 216-224, DOI: 10.1016/j.bcp.2015.05.002, AUG 1 2015
(Pyrrolo-pyridin-5-yl)benzamides	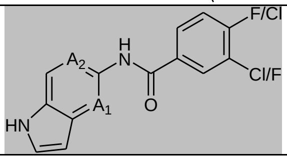	Benzamides: BBB permeable monoamine oxidase B inhibitors with neuroprotective effect on cortical neurons, Tzvetkov, NT; Stammler, HG; Hristova, S; Atanasov, AG; Antonov, L, EUROPEAN JOURNAL OF MEDICINAL CHEMISTRY,162, 793-809, DOI: 10.1016/j.ejmech.2018.11.009, JAN 15 2019

Q1	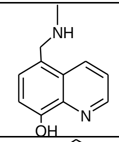	A review on iron chelators as potential therapeutic agents for the treatment of Alzheimer's and Parkinson's diseases. Singh, YP; Pandey, A; Vishwakarma, S; Modi, G, Molecular diversity, DOI:10.1007/s11030-018-9878-4, 2018-Oct-06. **Document type: review**
Q4	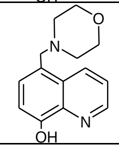	A review on iron chelators as potential therapeutic agents for the treatment of Alzheimer's and Parkinson's diseases. Singh, YP; Pandey, A; Vishwakarma, S; Modi, G, Molecular diversity, DOI:10.1007/s11030-018-9878-4, 2018-Oct-06. **Document type: review**
Quercetin	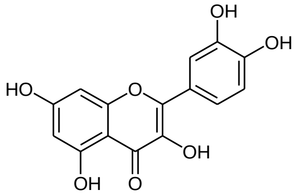	**1)** Piperine in combination with quercetin halt 6-OHDA induced neurodegeneration in experimental rats: Biochemical and neurochemical evidences, Singh, S; Kumar, P, NEUROSCIENCE RESEARCH,133, 38-47, DOI: 10.1016/j.neures.2017.10.006, AUG 2018, **2)** Molecular mechanisms underlying protective effects of quercetin against mitochondrial dysfunction and progressive dopaminergic neurodegeneration in cell culture and MitoPark transgenic mouse models of Parkinson's Disease, Ay, M; Luo, J; Langley, M; Jin, HJ; Anantharam, V; Kanthasamy, A; Kanthasamy, AG, JOURNAL OF NEUROCHEMISTRY,141, 5, 766-782, DOI: 10.1111/jnc.14033, JUN 2017, **3)** Recent advances in discovery and development of natural products as source for anti-Parkinson's disease lead compounds, Zhang, HJ; Bai, L; He, J; Zhong, L; Duan, XM; Ouyang, L; Zhu, YX; Wang, T; Shi, JY; Zhang, YW, EUROPEAN JOURNAL OF MEDICINAL CHEMISTRY,141, 257-272, DOI: 10.1016/j.ejmech.2017.09.068, DEC 1 2017, **Document Type:Review, 4) et cetera**
Quinelorane	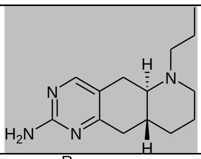	Duration of drug action of dopamine D2 agonists in mice with 6-hydroxydopamine-induced lesions, Tsuchioka, A; Oana, F; Suzuki, T; Yamauchi, Y; Ijiro, T; Kaidoh, K; Hiratochi, M, NEUROREPORT,26, 18, 1126-1132, DOI: 10.1097/WNR.0000000000000484, DEC 16 2015
Quinolines Derivatives as SUMOylation activators	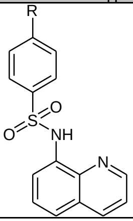	Small molecule SUMOylation activators are novel neuroprotective agents, Krajnak, K; Dahl, R, BIOORGANIC & MEDICINAL CHEMISTRY LETTERS,28, 3, 405-409, DOI: 10.1016/j.bmcl.2017.12.028, FEB 1 2018
Radiprodil	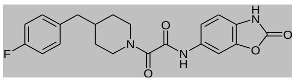	Unprecedented Therapeutic Potential with a Combination of A/NR2B Receptor Antagonists as Observed in the 6-OHDA Lesioned Rat Model of Parkinson's Disease, Michel, A; Downey, P; Nicolas, JM; Scheller, D, PLOS ONE,9, 12, Article Number: e114086, DOI: 10.1371/journal.pone.0114086, DEC 16 2014
Radotinib	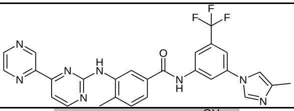	The c-Abl inhibitor, Radotinib HCl, is neuroprotective in a preclinical Parkinson's disease mouse model, Lee, S; Kim, S; Park, YJ; Yun, SP; Kwon, SH; Kim, D; Kim, DY; Shin, JS; Cho, DJ; Lee, GY et al, HUMAN MOLECULAR GENETICS,27, 13, 2344-2356, DOI: 10.1093/hmg/ddy143, JUL 1 2018
Raloxifene	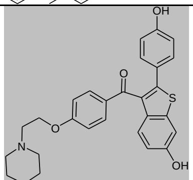	Neuroprotective and immunomodulatory effects of raloxifene in the myenteric plexus of a mouse model of Parkinson's disease, Poirier, AA; Cote, M; Bourque, M; Morissette, M; Di Paolo, T; Soulet, D, NEUROBIOLOGY OF AGING,48, 61-71, DOI: 10.1016/j.neurobiolaging.2016.08.004, DEC 2016
Rapamycin	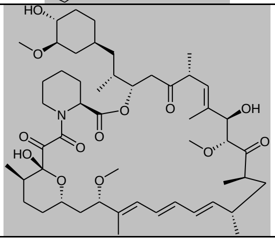	**1)** Novel tactics for neuroprotection in Parkinson's disease: Role of antibiotics, polyphenols and neuropeptides, Reglodi, D; Renaud, J; Tamas, A; Tizabi, Y; Socias, SB; Del-Bel, E; Raisman-Vozari, R; PROGRESS IN NEUROBIOLOGY, 155, 120-148 Special : SI, DOI: 10.1016/j.pneurobio.2015.10.004, AUG 2017, **Document Type:Review 2)** Development of Multifunctional Molecules as Potential Therapeutic Candidates for Alzheimer's Disease, Parkinson's Disease, and Amyotrophic Lateral Sclerosis in the Last Decade. Savelieff, MG; Nam, G; Kang, J; Lee, HJ; Lee, M; Lim, MH. CHEMICAL REVIEWS, 119, 2, 1221-1322, DOI: 10.1021/acs.chemrev.8b00138, JAN 23 2019, **Document Type:Review**
Rasagiline (TVP1022) and derivatives	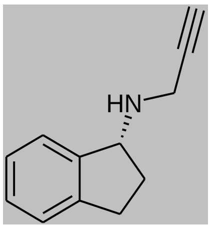	**1)** Design, synthesis and bioevalucation of novel 2,3-dihydro-1H-inden-1-amine derivatives as potent and selective human monoamine oxidase B inhibitors based on rasagiline, Xiao, X; Zhang, XX; Zhan, MM; Cheng, K; Li, SY; Xie, ZL; Liao, CZ, EUROPEAN JOURNAL OF MEDICINAL CHEMISTRY,145, 588-593, DOI: 10.1016/j.ejmech.2018.01.029, FEB 10 2018, **2)** Dopamine D2 receptor gene variants and response to rasagiline in early Parkinson's disease: a pharmacogenetic study, Masellis, M [1,2,3,4,5]; Collinson, S; Freeman, N; Tampakeras, M; Levy, J; Tchelet, A; Eyal, E; Berkovich, E; Eliaz, RE; Abler, V et al, Group Author: ADAGIO Investigators, BRAIN,139, 2050-2062 Part: 7, DOI: 10.1093/brain/aww109, JUL 2016, **3) et cetera**

(R)-RC-33	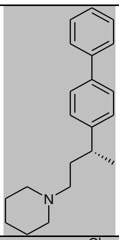	Toward the identification of neuroprotective agents: g-scale synthesis, pharmacokinetic evaluation and CNS distribution of (R)-RC-33, a promising Sigma1 receptor agonist, Marra, A; Rossi, D; Pignataro, L; Bigogno, C; Canta, A; Oggioni, N; Malacrida, A; Corbo, M; Cavaletti, G; Peviani, M et al, FUTURE MEDICINAL CHEMISTRY,8, 3, 287-295, DOI: 10.4155/fmc.15.191, 2016
Rebamipide	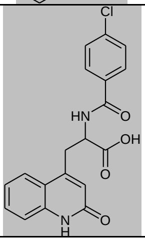	Rebamipide Mitigates Impairments in Mitochondrial Function and Bioenergetics with -Synuclein Pathology in 6-OHDA-Induced Hemiparkinson's Model in Rats, Mishra, A; Krishnamurthy, S, NEUROTOXICITY RESEARCH,35, 3, 542-562, DOI: 10.1007/s12640-018-9983-2, APR 2019
Resveratrol	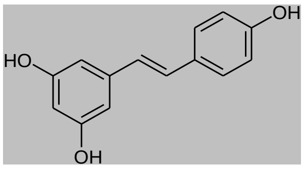	**1)** Resveratrol alleviates motor and cognitive deficits and neuropathology in the A53T-synuclein mouse model of Parkinson's disease, Zhang, LF; Yu, XL; Ji, M; Liu, SY; Wu, XL; Wang, YJ; Liu, RT, FOOD & FUNCTION,9, 12, 6415-6427, DOI: 10.1039/c8fo00964c, DEC 1 2018, **2)** Resveratrol Brain Delivery for Neurological Disorders Prevention and Treatment, Andrade, S; Ramalho, MJ; Pereira, MD; Loureiro, JA, FRONTIERS IN PHARMACOLOGY,9, Article Number: 1261, DOI: 10.3389/fphar.2018.01261, NOV 20 2018, **Document Type:Review, 3)** Connection between Systemic Inflammation and Neuroinflammation Underlies Neuroprotective Mechanism of Several Phytochemicals in Neurodegenerative Diseases, Wang, JT; Song, YT; Chen, Z; Leng, SAX, OXIDATIVE MEDICINE AND CELLULAR LONGEVITY, Article Number: 1972714, DOI: 10.1155/2018/1972714, 2018, **Document Type: Review, 4)** Resveratrol improves cognition of rats impaired by carotid artery stenosis through the cholinergic system., Liu, B; Xu, LL; Guo, MJ; Du, XM; Yan, LH; Wang, QL; Wang, JG; Qin, CK, BIOMEDICAL RESEARCH-INDIA,28, S286-S292 Special : SI, 2017, **5)** Recent advances in discovery and development of natural products as source for anti-Parkinson's disease lead compounds, Zhang, HJ; Bai, L; He, J; Zhong, L; Duan, XM; Ouyang, L; Zhu, YX; Wang, T; Shi, JY; Zhang, YW, EUROPEAN JOURNAL OF MEDICINAL CHEMISTRY,141, 257-272, DOI: 10.1016/j.ejmech.2017.09.068, DEC 1 2017, **Document Type:Review, 6) et cetera**
Rhaponticin	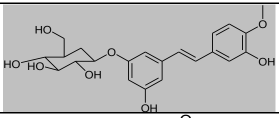	Effects of Rhei Undulati Rhizoma on lipopolysaccharide-induced neuroinflammation in vitro and in vivo, Hwang, DS; Gu, PS; Kim, N; Jang, YP; Oh, MS, ENVIRONMENTAL TOXICOLOGY,33, 1, 23-31, DOI: 10.1002/tox.22463, JAN 2018
Riboflavin	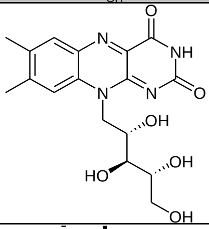	**1)** Riboflavin in Human Health: A Review of Current Evidences., Saedisomeolia, Ahmad; Ashoori, Marziyeh, Advances in food and nutrition research, 83 :57-81, DOI:10.1016/bs.afnr.2017.11.002, 2018, **Document Type:Review**, **2)** Riboflavin Has Neuroprotective Potential: Focus on Parkinson's Disease and Migraine, Marashly, ET; Bohlega, SA, FRONTIERS IN NEUROLOGY,8, Article Number: 333, DOI: 10.3389/fneur.2017.00333, JUL 20 2017, **Document Type:Review**
Rifampicin (ASI-3)	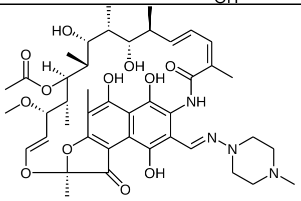	**1)** Novel tactics for neuroprotection in Parkinson's disease: Role of antibiotics, polyphenols and neuropeptides, Reglodi, D; Renaud, J; Tamas, A; Tizabi, Y; Socias, SB; Del-Bel, E; Raisman-Vozari, R; PROGRESS IN NEUROBIOLOGY, 155, 120-148 Special : SI, DOI: 10.1016/j.pneurobio.2015.10.004, AUG 2017, **Document Type:Review 2)** Development of Multifunctional Molecules as Potential Therapeutic Candidates for Alzheimer's Disease, Parkinson's Disease, and Amyotrophic Lateral Sclerosis in the Last Decade. Savelieff, MG; Nam, G; Kang, J; Lee, HJ; Lee, M; Lim, MH. CHEMICAL REVIEWS, 119, 2, 1221-1322, DOI: 10.1021/acs.chemrev.8b00138, JAN 23 2019, **Document Type:Review**
Rimonabant	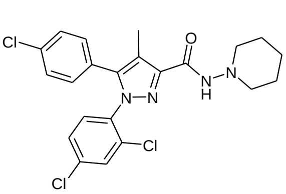	**1)** The role of cannabinoids and leptin in neurological diseases, Agar, E; ACTA NEUROLOGICA SCANDINAVICA, 132, 6, 371-380, DOI: 10.1111/ane.12411, DEC 2015, **Document Type:Review, 2)** Promising cannabinoid-based therapies for Parkinson's disease: motor symptoms to neuroprotection, More, SV; Choi, DK; MOLECULAR NEURODEGENERATION, 10, Article Number: 17, DOI: 10.1186/s13024-015-0012-0, APR 8 2015, **Document Type:Review, 3)** Neuroprotective Potential of Adenosine A2A and Cannabinoid CB1 Receptor Antagonists in an Animal Model of Parkinson Disease, Cerri, S; Levandis, G; Ambrosi, G; Montepeloso, E; Antoninetti, GF; Franco, R; Lanciego, JL; Baqi, Y; Muller, CE; Pinna, A et al, JOURNAL OF NEUROPATHOLOGY AND EXPERIMENTAL NEUROLOGY,73, 5, 414-424, DOI: 10.1097/NEN.0000000000000064, MAY 2014, **4) et cetera**

Rivastigmine	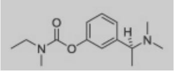	Current approaches to the treatment of Parkinson's Disease, Ellis, JM; Fell, MJ, BIOORGANIC & MEDICINAL CHEMISTRY LETTERS, 27, 18, 4247-4255, DOI: 10.1016/j.bmcl.2017.07.075, Published: SEP 15 2017
Ropinirole	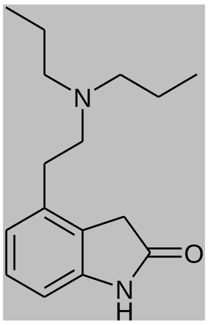	**1)** Orally-dissolving film for sublingual and buccal delivery of ropinirole, Lai, KL; Fang, Y; Han, H; Li, QQ; Zhang, S; Li, HY; Chow, SF; Lam, TN; Lee, WYT, COLLOIDS AND SURFACES B-BIOINTERFACES,163, 9-18, DOI: 10.1016/j.colsurfb.2017.12.015, MAR 1 2018, **2)** D-512, a novel dopamine D-2/3 receptor agonist, demonstrates greater anti-Parkinsonian efficacy than ropinirole in Parkinsonian rats, Lindenbach, D; Das, B; Conti, MM; Meadows, SM; Dutta, AK; Bishop, C, BRITISH JOURNAL OF PHARMACOLOGY,174, 18, 3058-3071, DOI: 10.1111/bph.13937, SEP 2017, **3)** Prevalence and Correlates of Anti-Parkinson Drug Use in a Nationally Representative Sample, Dahodwala, N; Willis, AW; Li, PX; Doshi, JA, MOVEMENT DISORDERS CLINICAL PRACTICE,4, 3, 335-341, DOI: 10.1002/mdc3.12422, MAY-JUN 2017 **4)** Advances in non-dopaminergic treatments for Parkinson's disease. Stayte, S; Vissel, B; FRONTIERS IN NEUROSCIENCE, 8, Article Number: 113, DOI: 10.3389/fnins.2014.00113, MAY 22 2014, **Document Type: Review, 5) et cetera**
Rosiglitazone	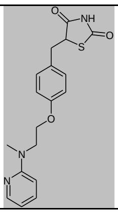	Repurposing of Copper -chelating Drugs for the Treatment of Neurodegenerative Diseases. Lanza, V; Milardi, D; Di Natale, G; Pappalardo, G. CURRENT MEDICINAL CHEMISTRY, 25, 4, 525-539, DOI: 10.2174/0929867324666170518094404, 2018, **Document Type: Review**
Rosmarinic acid	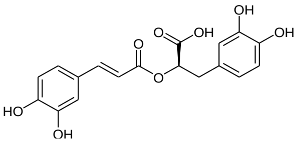	**1)** Rosmarinic acid protects against MPTP-induced toxicity and inhibits iron induced alpha-synuclein aggregation, Qu, L; Xu, HM; Jia, WT; Jiang, H; Xie, JX [1,2,3,4], NEUROPHARMACOLOGY,144, 291-300, DOI: 10.1016/j.neuropharm.2018.09.042, JAN 2019, **2)** Neuroprotection Comparison of Rosmarinic Acid and Carnosic Acid in Primary Cultures of Cerebellar Granule Neurons, Taram, F; Ignowski, E; Duval, N; Linseman, DA, MOLECULES,23, 11, Article Number: 2956, DOI: 10.3390/molecules23112956, NOV 2018, **3) et cetera**
Rosuvastatin	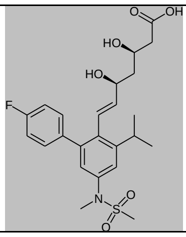	**1)** The beneficial effects of HMG-CoA reductase inhibitors in the processes of neurodegeneration, Saravi, SSS; Saravi, SSS; Arefidoust, A; Dehpour, AR, METABOLIC BRAIN DISEASE,32, 4, 949-965, DOI: 10.1007/s11011-017-0021-5, AUG 2017, **Document Type:Review**, **2)** Neuroprotective mechanisms of statins in neurodegenerative diseases, Yan, JQ; Sun, JC; Li, XM; Zhai, MM; Jia, XF, INTERNATIONAL JOURNAL OF CLINICAL AND EXPERIMENTAL MEDICINE,9, 6, 9799-9805, 2016, **Document Type:Review**
Rotigotine	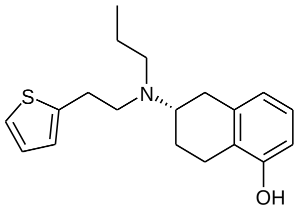	**1)** Prevalence and Correlates of Anti-Parkinson Drug Use in a Nationally Representative Sample, Dahodwala, N; Willis, AW; Li, PX; Doshi, JA, MOVEMENT DISORDERS CLINICAL PRACTICE,4, 3, 335-341, DOI: 10.1002/mdc3.12422, MAY-JUN 2017, **2)** Duration of drug action of dopamine D2 agonists in mice with 6-hydroxydopamine-induced lesions, Tsuchioka, A; Oana, F; Suzuki, T; Yamauchi, Y; Ijiro, T; Kaidoh, K; Hiratochi, M, NEUROREPORT,26, 18, 1126-1132, DOI: 10.1097/WNR.0000000000000484, DEC 16 2015, **3)** Parkinson's Disease Treatment May Cause Impulse-Control Disorder Via Dopamine D3 Receptors, Seeman, P, SYNAPSE,69, 4, 183-189, DOI: 10.1002/syn.21805, APR 2015, **4)** Advances in non-dopaminergic treatments for Parkinson's disease. Stayte, S; Vissel, B; FRONTIERS IN NEUROSCIENCE, 8, Article Number: 113, DOI: 10.3389/fnins.2014.00113, MAY 22 2014, **Document Type: Review, 5) et cetera**
Rutin	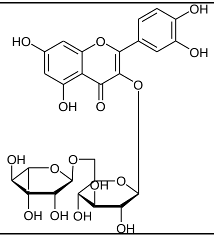	**1)** Flavonoids and its Neuroprotective Effects on Brain Ischemia and Neurodegenerative Diseases, Putteeraj, M; Lim, WL; Teoh, SL; Yahaya, MF, CURRENT DRUG TARGETS,19, 14, 1710-1720, DOI: 10.2174/1389450119666180326125252, 2018, **Document Type:Review, 2)** Protective effects of quercetin glycosides, rutin, and isoquercetrin against 6-hydroxydopamine -induced neurotoxicity in rat pheochromocytoma cells, Magalingam, KB; Radhakrishnan, A; Haleagrahara, N, INTERNATIONAL JOURNAL OF IMMUNOPATHOLOGY AND PHARMACOLOGY,29, 1, 30-39, DOI: 10.1177/0394632015613039, MAR 2016
Safinamide	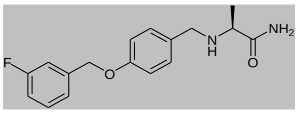	**1)** Safinamide: a new hope for Parkinson's disease?, Teixeira, FG; Gago, MF; Marques, P; Moreira, PS; Magalhaes, R; Sousa, N; Salgado, AJ, DRUG DISCOVERY TODAY,23, 3, 736-744, DOI: 10.1016/j.drudis.2018.01.033, MAR 2018, **Document Type:Review**, **2)** Clinical Pharmacokinetics and Pharmacodynamics of Safinamide, Muller, T; Foley, P, CLINICAL PHARMACOKINETICS,56, 3, 251-261, DOI: 10.1007/s40262-016-0449-5, MAR 2017, **Document Type:Review**, **3)** et cetera
Safranal	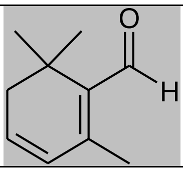	**1)** Application of Monoterpenoids and their Derivatives for Treatment of Neurodegenerative Disorders, Volcho, KP; Laev, SS; Ashraf, GM; Aliev, G; Salakhutdinov, NF, CURRENT MEDICINAL CHEMISTRY,25, 39, 5327-5346, DOI: 10.2174/0929867324666170112101837, 2018, **Document Type:Review, 2)** Safranal prevents rotenone-induced oxidative stress and apoptosis in an in vitro model of Parkinson's disease through regulating Keap1/Nrf2 signaling

		pathway, Pan, PK; Qiao, LY; Wen, XN, CELLULAR AND MOLECULAR BIOLOGY,62, 14, 11-17, DOI: 10.14715/cmb/2016.62.14.2, 2016
SAGE 217	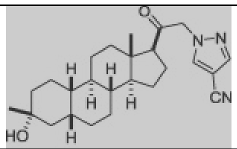	Current approaches to the treatment of Parkinson's Disease, Ellis, JM; Fell, MJ, BIOORGANIC & MEDICINAL CHEMISTRY LETTERS, 27, 18, 4247-4255, DOI: 10.1016/j.bmcl.2017.07.075, Published: SEP 15 2017
Saikosaponin a	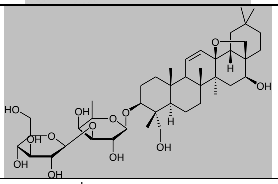	Pharmacokinetics and Brain Distribution of the Active Components of DA-9805, Saikosaponin A, Paeonol and Imperatorin in Rats, Kwon, MH; Jeong, JS; Ryu, J; Cho, YW; Kang, HE, PHARMACEUTICS,10, 3, Article Number: 133, DOI: 10.3390/pharmaceutics10030133, SEP 2018
Salicylate, sodium salt	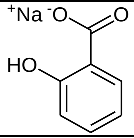	Modulatory effects of sodium salicylate on the factors affecting protein aggregation during rotenone induced Parkinson's disease pathology, Thakur, P; Nehru, B, NEUROCHEMISTRY INTERNATIONAL,75, 1-10, DOI: 10.1016/j.neuint.2014.05.002, SEP 2014
Salidroside	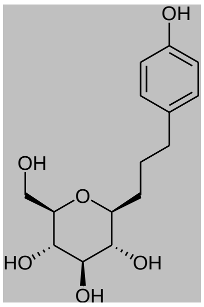	**1)** Pharmacological activities, mechanisms of action, and safety of salidroside in the central nervous system, Zhong, ZF; Han, J; Zhang, JZ; Xiao, Q; Hu, J; Chen, LD, DRUG DESIGN DEVELOPMENT AND THERAPY,12, 1479-1489, DOI: 10.2147/DDDT.S160776, 2018, **Document Type:Review**, **2)** Salidroside Protection Against Oxidative Stress Injury Through the Wnt/beta-Catenin Signaling Pathway in Rats with Parkinson's Disease, Wu, DM; Han, XR; Wen, X; Wang, S; Fan, SH; Zhuang, J; Wang, YJ; Zhang, ZF; Li, MQ; Hu, B et al, CELLULAR PHYSIOLOGY AND BIOCHEMISTRY,46, 5, 1793-1806, DOI: 10.1159/000489365, 2018, **3)** Protective Effects of Salidroside in the MPTP/MPP+-Induced Model of Parkinson's Disease through ROS-NO-Related Mitochondrion Pathway, Wang, SH; He, H; Chen, L; Zhang, W; Zhang, XJ; Chen, JZ, MOLECULAR NEUROBIOLOGY,51, 2, 718-728, DOI: 10.1007/s12035-014-8755-0, APR 2015, **4) et cetera**
Salusin-beta	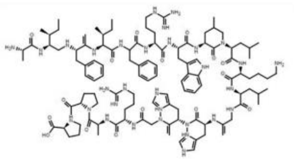	Salusin-beta mediate neuroprotective effects for Parkinson's disease, Chang, Y; Yoo, J; Kim, H; Park, HJ; Jeon, S; Kim, J, BIOCHEMICAL AND BIOPHYSICAL RESEARCH COMMUNICATIONS,503, 3, 1428-1433, DOI: 10.1016/j.bbrc.2018.07.059, SEP 10 2018
Salvianolic Acid B	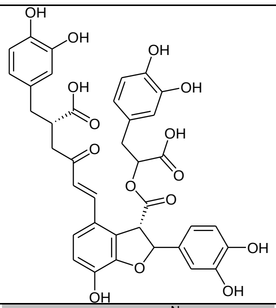	Recent advances in discovery and development of natural products as source for anti-Parkinson's disease lead compounds, Zhang, HJ; Bai, L; He, J; Zhong, L; Duan, XM; Ouyang, L; Zhu, YX; Wang, T; Shi, JY; Zhang, YW, EUROPEAN JOURNAL OF MEDICINAL CHEMISTRY,141, 257-272, DOI: 10.1016/j.ejmech.2017.09.068, DEC 1 2017, **Document Type:Review**
Sarizotan	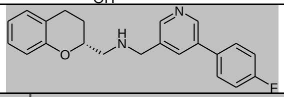	Advances in non-dopaminergic treatments for Parkinson's disease. Stayte, S; Vissel, B; FRONTIERS IN NEUROSCIENCE, 8, Article Number: 113, DOI: 10.3389/fnins.2014.00113, MAY 22 2014, **Document Type: Review**
Sativex	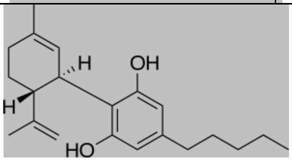	**1)** The role of cannabinoids and leptin in neurological diseases, Agar, E; ACTA NEUROLOGICA SCANDINAVICA, 132, 6, 371-380, DOI: 10.1111/ane.12411, DEC 2015, **Document Type:Review, 2)** Promising cannabinoid-based therapies for Parkinson's disease: motor symptoms to neuroprotection, More, SV; Choi, DK; MOLECULAR NEURODEGENERATION, 10, Article Number: 17, DOI: 10.1186/s13024-015-0012-0, APR 8 2015, **Document Type:Review, 3) et cetera**
Saxagliptin	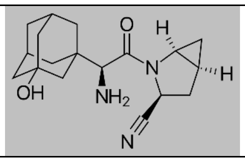	Parkinson's Disease, Diabetes and Cognitive Impairment, Ashraghi, MR; Pagano, G; Polychronis, S; Niccolini, F; Politis, M, RECENT PATENTS ON ENDOCRINE METABOLIC & IMMUNE DRUG DISCOVERY,10, 1, 11-21, DOI: 10.2174/1872214810999160628105549, 2016, **Document Type: Review**
SCH-58261	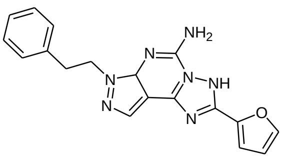	**1)** Advances in non-dopaminergic treatments for Parkinson's disease. Stayte, S; Vissel, B; FRONTIERS IN NEUROSCIENCE, 8, Article Number: 113, DOI: 10.3389/fnins.2014.00113, MAY 22 2014, **Document Type: Review, 2)** Unprecedented Therapeutic Potential with a Combination of A/NR2B Receptor Antagonists as Observed in the 6-OHDA Lesioned Rat Model of Parkinson's Disease, Michel, A; Downey, P; Nicolas, JM; Scheller, D, PLOS ONE,9, 12, Article Number: e114086, DOI: 10.1371/journal.pone.0114086, DEC 16 2014

SCH412348	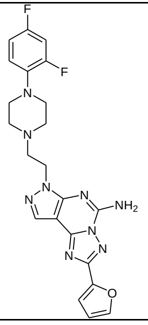	Advances in non-dopaminergic treatments for Parkinson's disease. Stayte, S; Vissel, B; FRONTIERS IN NEUROSCIENCE, 8, Article Number: 113, DOI: 10.3389/fnins.2014.00113, MAY 22 2014, **Document Type: Review**
SCH 900800		Advances in Drug Development for Parkinson's Disease: Present Status. Reddy, DH; Misra, S; Medhi, B. PHARMACOLOGY, 93, 5-6, 260-271, DOI: 10.1159/000362419, 2014
Schisantherin A	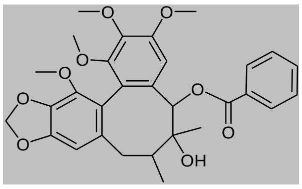	**1)** Small-Sized mPEG PLGA Nanoparticles of Schisantherin A with Sustained Release for Enhanced Brain Uptake and Anti-Parkinsonian Activity, Chen, T; Li, CW; Li, Y; Yi, X; Wang, RB; Lee, SMY; Zheng, Y, ACS APPLIED MATERIALS & INTERFACES,9, 11, 9516-9527, DOI: 10.1021/acsami.7b01171, MAR 22 2017, **2)** Oral Delivery of a Nanocrystal Formulation of Schisantherin A with Improved Bioavailability and Brain Delivery for the Treatment of Parkinson's Disease, Chen, TK; Li, CW; Li, Y; Yi, X; Lee, SMY; Zheng, Y, MOLECULAR PHARMACEUTICS,13, 11, 3864-3875, DOI: 10.1021/acs.molpharmaceut.6b00644, NOV 2016, **3) et cetera**
Scoparone (6,7-dimethoxycoumarin)	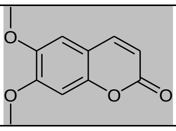	Scoparone Inhibits LPS-Simulated Inflammatory Response by Suppressing IRF3 and ERK in BV-2 Microglial Cells, Cho, DY; Ko, HM; Kim, J; Kim, BW; Yun, YS; Park, JI; Ganesan, P; Lee, JT; Choi, DK, MOLECULES,21, 12, Article Number: 1718, DOI: 10.3390/molecules21121718, DEC 2016
SDZ 220-58		Advances in non-dopaminergic treatments for Parkinson's disease. Stayte, S; Vissel, B ; FRONTIERS IN NEUROSCIENCE, 8, Article Number: 113, DOI: 10.3389/fnins.2014.00113, MAY 22 2014
Securinine aminoderivatives	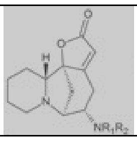	Securinine Derivatives as Potential Anti-amyloid Therapeutic Approach, Neganova, ME; Klochkov, SG; Petrova, LN; Shevtsova, EF; Afanasieva, SV; Chudinova, ES; Fisenko, VP; Bachurin, SO; Barreto, GE; Aliev, G [1,5,6,7], CNS & NEUROLOGICAL DISORDERS-DRUG TARGETS,16, 3, 351-355, DOI: 10.2174/1871527315666161107090525, 2017
Selegiline (1,3-bisbenzylimidazolium, l-deprenyl)	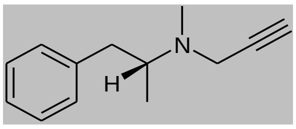	**1)** l-Deprenyl exerts cytotoxicity towards acute myeloid leukemia through inhibition of mitochondrial respiration, Ryu, I; Ryu, MJ; Han, J; Kim, SJ; Lee, MJ; Ju, XS; Yoo, BH; Lee, YL; Jang, Y; Song, IC et al, ONCOLOGY REPORTS,40, 6, 3869-3878, DOI: 10.3892/or.2018.6753, DEC 2018**, 2)** Pharmacokinetics and pharmacodynamics of intranasally administered selegiline nanoparticles with improved brain delivery in Parkinson's disease, Sridhar, V; Gaud, R; Bajaj, A; Wairkar, S, NANOMEDICINE-NANOTECHNOLOGY BIOLOGY AND MEDICINE,14, 8, 2609-2618, DOI: 10.1016/j.nano.2018.08.004, NOV 2018, **3)** Brain targeted delivery of mucoadhesive thermosensitive nasal gel of selegiline hydrochloride for treatment of Parkinson's disease, Sridhar, V; Wairkar, S; Gaud, R; Bajaj, A; Meshram, P, JOURNAL OF DRUG TARGETING,26, 2, 150-161, DOI: 10.1080/1061186X.2017.1350858, 2018, **4)** A novel selective MAO-B inhibitor with neuroprotective and anti-Parkinsonian properties, Chan, HH; Tse, MK; Kumar, S; Zhuo, L; EUROPEAN JOURNAL OF PHARMACOLOGY, 818, 254-262, DOI: 10.1016/j.ejphar.2017.10.023, JAN 5 2018 **5)** The significance of selegiline/-deprenyl after 50 years in research and therapy, Miklya, I, MOLECULAR PSYCHIATRY,21, 11, 1499-1503, DOI: 10.1038/mp.2016.127, NOV 2016, **Document Type:Review**, **6)** et cetera
Semaglutide	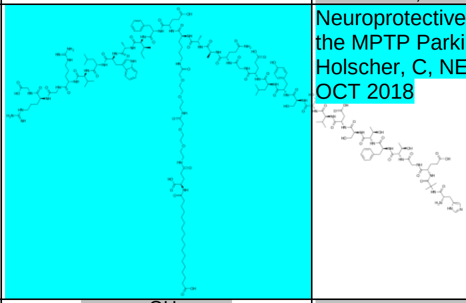	Neuroprotective effects of the novel GLP-1 long acting analogue semaglutide in the MPTP Parkinson's disease mouse model, Zhang, LP; Zhang, LY; Li, L; Holscher, C, NEUROPEPTIDES,71, 70-80, DOI: 10.1016/j.npep.2018.07.003, OCT 2018
Scyllo-inositol	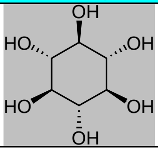	Development of Multifunctional Molecules as Potential Therapeutic Candidates for Alzheimer's Disease, Parkinson's Disease, and Amyotrophic Lateral Sclerosis in the Last Decade. Savelieff, MG; Nam, G; Kang, J; Lee, HJ; Lee, M; Lim, MH. CHEMICAL REVIEWS, 119, 2, 1221-1322, DOI: 10.1021/acs.chemrev.8b00138, JAN 23 2019, **Document Type:Review**
D-Ser2-Oxyntomodulin		Neuroprotective effects of an oxyntomodulin analogue in the MPTP mouse model of Parkinson's disease, Liu, WZ; Li, YW; Jalewa, J; Saunders-Wood, T; Li, L; Holscher, C, EUROPEAN JOURNAL OF PHARMACOLOGY,765, 284-290, DOI: 10.1016/j.ejphar.2015.08.038, OCT 15 2015

Shatavarin IV	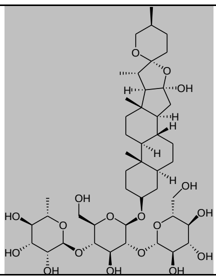	Shatavarin IV elicits lifespan extension and alleviates Parkinsonism in Caenorhabditis elegans, Smita, SS; Sammi, SR; Laxman, TS; Bhatta, RS; Pandey, R, FREE RADICAL RESEARCH,51, 11-12, 954-969, DOI: 10.1080/10715762.2017.1395419, 2017
6-Shogaol	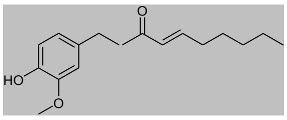	6-Shogaol, an active constituent of ginger, attenuates neuroinflammation and cognitive deficits in animal models of dementia, Moon, M; Kim, HG; Choi, JG; Oh, H; Lee, PKJ; Ha, SK; Kim, SY; Park, Y; Huh, Y; Oh, MS, BIOCHEMICAL AND BIOPHYSICAL RESEARCH COMMUNICATIONS,449, 1, 8-13, DOI: 10.1016/j.bbrc.2014.04.121, JUN 20 2014
Silibinin (silybin) A, B	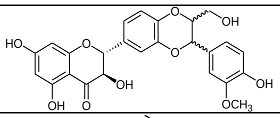	A review on iron chelators as potential therapeutic agents for the treatment of Alzheimer's and Parkinson's diseases. Singh, YP; Pandey, A; Vishwakarma, S; Modi, G, Molecular diversity, DOI:10.1007/s11030-018-9878-4, 2018-Oct-06. **Document type: review**
Silydianin	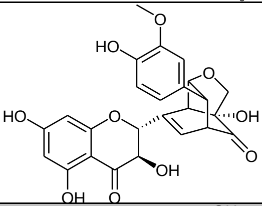	Iron Chelating Strategies in Systemic Metal Overload, Neurodegeneration and Cancer. Gumienna-Kontecka, E; Pyrkosz-Bulska, M; Szebesczyk, A; Ostrowska, M. CURRENT MEDICINAL CHEMISTRY, 21, 33, 3741-3767, DOI: 10.2174/0929867321666140706143402, 2014. **Document Type: Review**
Simvastatin	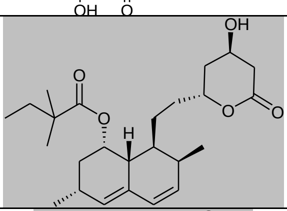	**1)** The beneficial effects of HMG-CoA reductase inhibitors in the processes of neurodegeneration, Saravi, SSS; Saravi, SSS; Arefidoust, A; Dehpour, AR, METABOLIC BRAIN DISEASE,32, 4, 949-965, DOI: 10.1007/s11011-017-0021-5, AUG 2017, **Document Type:Review**, **2)** Neuroprotective mechanisms of statins in neurodegenerative diseases, Yan, JQ; Sun, JC; Li, XM; Zhai, MM; Jia, XF, INTERNATIONAL JOURNAL OF CLINICAL AND EXPERIMENTAL MEDICINE,9, 6, 9799-9805, 2016, **Document Type:Review**
Sinapic acid	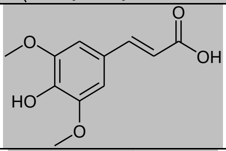	The neuroprotective potential of sinapic acid in the 6-hydroxydopamine-induced hemi-parkinsonian rat, Zare, K; Eidi, A; Roghani, M; Rohani, AH, METABOLIC BRAIN DISEASE,30, 1, 205-213, DOI: 10.1007/s11011-014-9604-6, FEB 2015
Sinomenine	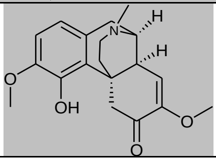	A Systematic Review on the Sinomenine Derivatives, Tang, J; Raza, A; Chen, J; Xu, HX, MINI-REVIEWS IN MEDICINAL CHEMISTRY,18, 11, 906-917, DOI: 10.2174/1389557517666171123212557, 2018, **Document Type:Review**
SIRT3		SIRT3 Acts as a Neuroprotective Agent in Rotenone-Induced Parkinson Cell Model, Zhang, JY; Deng, YN; Zhang, M; Su, H; Qu, QM, NEUROCHEMICAL RESEARCH,41, 7, 1761-1773, DOI: 10.1007/s11064-016-1892-2, JUL 2016
Sitagliptin	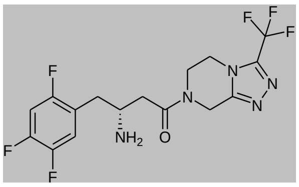	**1)** Sitagliptin rescues memory deficits in Parkinsonian rats via upregulating BDNF to prevent neuron and dendritic spine loss, Li, J; Zhang, SH; Li, CY; Li, M; Ma, L, NEUROLOGICAL RESEARCH,40, 9, 736-743, DOI: 10.1080/01616412.2018.1474840, 2018, **2)** Sitagliptin and liraglutide reversed nigrostriatal degeneration of rodent brain in rotenone-induced Parkinson's disease, Badawi, GA; Abd El Fattah, MA; Zaki, HF; El Sayed, MI, INFLAMMOPHARMACOLOGY,25, 3, 369-382, DOI: 10.1007/s10787-017-0331-6, JUN 2017, **3)** Parkinson's Disease, Diabetes and Cognitive Impairment, Ashraghi, MR; Pagano, G; Polychronis, S; Niccolini, F; Politis, M, RECENT PATENTS ON ENDOCRINE METABOLIC & IMMUNE DRUG DISCOVERY,10, 1, 11-21, DOI: 10.2174/1872214810999160628105549, 2016, **Document Type: Review**
Smilagenin	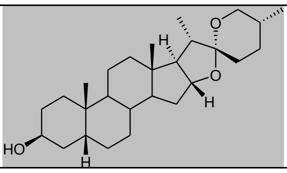	Advances in Drug Development for Parkinson's Disease: Present Status. Reddy, DH; Misra, S; Medhi, B. PHARMACOLOGY, 93, 5-6, 260-271, DOI: 10.1159/000362419, 2014, **Document Type: Review**
Soluble epoxide hydrolase		Humble beginnings with big goals: Small molecule soluble epoxide hydrolase inhibitors for treating CNS disorders, Zarriello, S; Tuazon, JP; Corey, S; Schimmel, S; Rajani, M; Gorsky, A; Incontri, D; Hammock, BD; Borlongan, CV, PROGRESS IN NEUROBIOLOGY,172, 23-39, DOI: 10.1016/j.pneurobio.2018.11.001, JAN 2019, **Document Type:Review**

ST1535	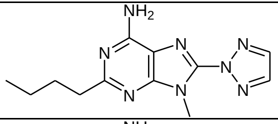	Adenosine A Receptor Antagonists in Parkinson's Disease: Progress in Clinical Trials from the Newly Approved Istradefylline to Drugs in Early Development and Those Already Discontinued, Pinna, A, CNS DRUGS,28, 5, 455-474, DOI: 10.1007/s40263-014-0161-7, MAY 2014, **Document Type: Review**
ST4206	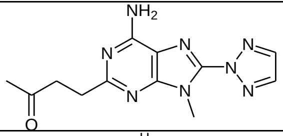	Adenosine A Receptor Antagonists in Parkinson's Disease: Progress in Clinical Trials from the Newly Approved Istradefylline to Drugs in Early Development and Those Already Discontinued, Pinna, A, CNS DRUGS,28, 5, 455-474, DOI: 10.1007/s40263-014-0161-7, MAY 2014, **Document Type: Review**
Staurosporine	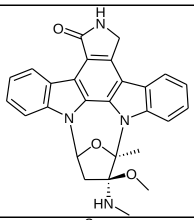	Marine-derived protein kinase inhibitors for neuroinflammatory diseases, Ning, C; Wang, HMD; Gao, R; Chang, YC; Hu, FQ; Meng, XJ; Huang, SY, BIOMEDICAL ENGINEERING ONLINE,17, Article Number: 46, DOI: 10.1186/s12938-018-0477-5, APR 24 2018, **Document Type:Review**
Stemazole	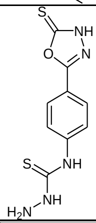	Neuroprotective effects of stemazole in the MPTP-induced acute model of Parkinson's disease: Involvement of the dopamine system, Guo, ZR; Xu, SS; Du, N; Liu, J; Huang, YY; Han, M, NEUROSCIENCE LETTERS,616, 152-159, DOI: 10.1016/j.neulet.2016.01.048, MAR 11 2016
L-Stepholidine	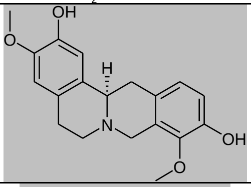	Recent advances in discovery and development of natural products as source for anti-Parkinson's disease lead compounds, Zhang, HJ; Bai, L; He, J; Zhong, L; Duan, XM; Ouyang, L; Zhu, YX; Wang, T; Shi, JY; Zhang, YW, EUROPEAN JOURNAL OF MEDICINAL CHEMISTRY,141, 257-272, DOI: 10.1016/j.ejmech.2017.09.068, DEC 1 2017, **Document Type:Review**
Steroids	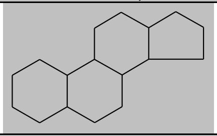	Exploring the potential of natural and synthetic neuroprotective steroids against neurodegenerative disorders: A literature review, Bansal, R; Singh, R, MEDICINAL RESEARCH REVIEWS,38, 4, 1126-1158, DOI: 10.1002/med.21458, JUL 2018, **Document Type:Review**
E-8-Styrylcaffeine	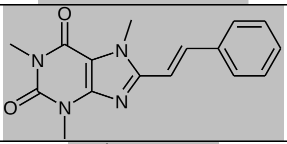	Xanthine derivatives as agents affecting non-dopaminergic neuroprotection in Parkinson`s disease., Kasabova-Angelova, A; Tzankova, D; Mitkov, J; Georgieva, M; Tzankova, V; Zlatkov, A; Kondeva-Burdina, M, Current medicinal chemistry, DOI:10.2174/0929867325666180821153316, 2018-Aug-21
SU4312	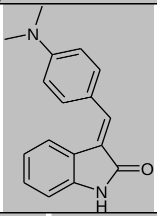	Substantial protection against MPTP-associated Parkinson's neurotoxicity in vitro and in vivo by anti-cancer agent SU4312 via activation of MEF2D and inhibition of MAO-B, Guo, BJ; Hu, SQ; Zheng, CY; Wang, HY; Luo, FC; Li, HT; Cui, W; Yang, XF; Cui, GZ; Mak, SH et al, NEUROPHARMACOLOGY,126, 12-24, DOI: 10.1016/j.neuropharm.2017.08.014, NOV 2017
4- and 5-Sulfanylphthalimide analogues	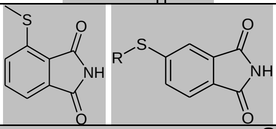	The adenosine receptor affinities and monoamine oxidase B inhibitory properties of sulfanylphthalimide analogues, Van der Walt, MM; Terre'Blanche, G; Petzer, A; Petzer, JP, BIOORGANIC CHEMISTRY,59, 117-123, DOI: 10.1016/j.bioorg.2015.02.005, APR 2015
Sulforaphane	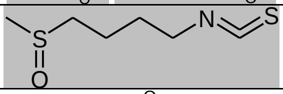	Redox-based therapeutics in neurodegenerative disease. McBean, GJ; Lopez, MG; Wallner, FK. BRITISH JOURNAL OF PHARMACOLOGY, 174, 12, 1750-1770, DOI: 10.1111/bph.13551, JUN 2017, **Document Type:Review**
Sulfuretin	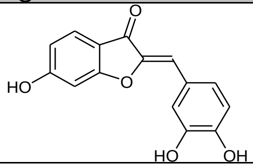	Sulfuretin inhibits 6-hydroxydopamine-induced neuronal cell death via reactive oxygen species-dependent mechanisms in human neuroblastoma SH-SY5Y cells, Kwon, SH; Ma, SX; Lee, SY; Jang, CG, NEUROCHEMISTRY INTERNATIONAL,74, 53-64, DOI: 10.1016/j.neuint.2014.04.016, JUL 2014
Sulphidric acid	H_2S	**1)** Evaluation of the antiparkinsonism and neuroprotective effects of hydrogen sulfide in acute 6-hydroxydopamine-induced animal model of Parkinson's disease: behavioral, histological and biochemical studies, Sarukhani, M; Haghdoost-Yazdi, H; Golezari, AS; Babayan-Tazehkand, A; Dargahi, T; Rastgoo, N, NEUROLOGICAL RESEARCH,40, 7, 525-533, DOI: 10.1080/01616412.2017.1390903, 2018, **2)** Cytoprotective effects of hydrogen sulfide-releasing N-methyl-D-aspartate receptor antagonists mediated by intracellular sulfane sulfur, Marutani, E; Sakaguchi, M; Chen, W; Sasakura, K; Liu, JF; Xian, M; Hanaoka, K; Nagano, T; Ichinose, F, MEDCHEMCOMM,5, 10, 1577-1583, DOI: 10.1039/c4md00180j, OCT 2014

Synphilin-1		Synphilin-1 has neuroprotective effects on MPP+-induced Parkinson's disease model cells by inhibiting ROS production and apoptosis, Shishido, T; Nagano, Y; Araki, M; Kurashige, T; Obayashi, H; Nakamura, T; Takahashi, T; Matsumoto, M; Maruyama, H, NEUROSCIENCE LETTERS,690, 145-150, DOI: 10.1016/j.neulet.2018.10.020, JAN 18 2019
Talampanel	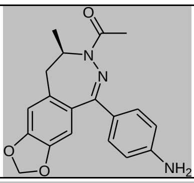	Advances in non-dopaminergic treatments for Parkinson's disease. Stayte, S; Vissel, B; FRONTIERS IN NEUROSCIENCE, 8, Article Number: 113, DOI: 10.3389/fnins.2014.00113, MAY 22 2014, **Document Type: Review**
Talipexole	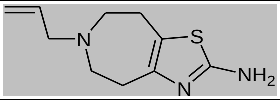	Duration of drug action of dopamine D2 agonists in mice with 6-hydroxydopamine-induced lesions, Tsuchioka, A; Oana, F; Suzuki, T; Yamauchi, Y; Ijiro, T; Kaidoh, K; Hiratochi, M, NEUROREPORT,26, 18, 1126-1132, DOI: 10.1097/WNR.0000000000000484, DEC 16 2015
Taltirelin	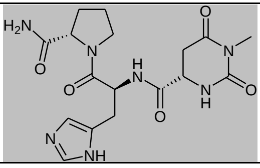	TRH Analog, Taltirelin Protects Dopaminergic Neurons From Neurotoxicity of MPTP and Rotenone, Zheng, C; Chen, GQ; Tan, Y; Zeng, WQ; Peng, QW; Wang, J; Cheng, C; Yang, XM; Nie, SK; Xu, Y et al, FRONTIERS IN CELLULAR NEUROSCIENCE,12, Article Number: 485, DOI: 10.3389/fncel.2018.00485, DEC 20 2018
Tandospirone	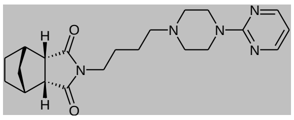	Role of tandospirone, a 5-HT1A receptor partial agonist, in the treatment of central nervous system disorders and the underlying mechanisms, Huang, XF; Yang, J; Yang, SJ; Cao, SS; Qin, DL; Zhou, Y; Li, XL; Ye, Y; Wu, JM, ONCOTARGET, 8, 60, 102705-102720, DOI: 10.18632/oncotarget.22170, Published: NOV 24 2017, **Document Type:Review**
Tangeretin	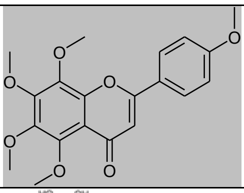	Neuroprotective Effects of Citrus Fruit-Derived Flavonoids, Nobiletin and Tangeretin in Alzheimer's and Parkinson's Disease, Braidy, N; Behzad, S; Habtemariam, S; Ahmed, T; Daglia, M; Nabavi, SM; Sobarzo-Sanchez, E; Nabavi, SF, CNS & NEUROLOGICAL DISORDERS-DRUG TARGETS,16, 4, 387-397, DOI: 10.2174/1871527316666170328113309, 2017, **Document Type:Review**
Tannic acid	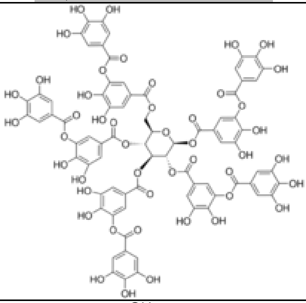	**1)** Natural polyphenols effects on protein aggregates in Alzheimer's and Parkinson's prion-like diseases, Freyssin, A; Page, G; Fauconneau, B; Bilan, AR, NEURAL REGENERATION RESEARCH,13, 6, 955-961, DOI: 10.4103/1673-5374.233432, JUN 2018, **Document Type:Review**, **2)** Polyphenols in Parkinson's Disease: A Systematic Review of In Vivo Studies, Kujawska, M; Jodynis-Liebert, J, NUTRIENTS,10, 5, Article Number: 642, DOI: 10.3390/nu10050642, MAY 2018, **Document Type:Review**, **3)** Novel tactics for neuroprotection in Parkinson's disease: Role of antibiotics, polyphenols and neuropeptides, Reglodi, D; Renaud, J; Tamas, A; Tizabi, Y; Socias, SB; Del-Bel, E; Raisman-Vozari, R, PROGRESS IN NEUROBIOLOGY,155, 120-148 Special : SI, DOI: 10.1016/j.pneurobio.2015.10.004, AUG 2017, **Document Type:Review**, **4) et cetera**
Tanshinol	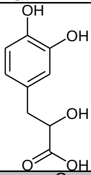	Recent advances in discovery and development of natural products as source for anti-Parkinson's disease lead compounds, Zhang, HJ; Bai, L; He, J; Zhong, L; Duan, XM; Ouyang, L; Zhu, YX; Wang, T; Shi, JY; Zhang, YW, EUROPEAN JOURNAL OF MEDICINAL CHEMISTRY,141, 257-272, DOI: 10.1016/j.ejmech.2017.09.068, DEC 1 2017, **Document Type:Review**
Tanshinone IIA	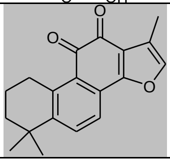	Recent progress in therapeutic strategies for microglia-mediated neuroinflammation in neuropathologies, Gupta, N; Shyamasundar, S; Patnala, R; Karthikeyan, A; Arumugam, TV; Ling, EA; Dheen, ST; EXPERT OPINION ON THERAPEUTIC TARGETS, 22, 9, 765-781, DOI: 10.1080/14728222.2018.1515917, 2018, **Document Type:Review**
TAT-HSA-alpha-MSH	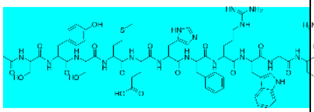	TAT-HSA-alpha-MSH fusion protein with extended half-life inhibits tumor necrosis factor-alpha in brain inflammation of mice, Wang, MZ; Zhi, DJ; Wang, HQ; Ru, Y; Ren, H; Wang, N; Liu, YY; Li, Y; Li, HY, APPLIED MICROBIOLOGY AND BIOTECHNOLOGY,100, 12, 5353-5361, DOI: 10.1007/s00253-015-7251-4, JUN 2016
Taurine	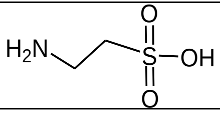	Taurine protects noradrenergic locus coeruleus neurons in a mouse Parkinson's disease model by inhibiting microglial M1 polarization, Hou, LY; Che, YN; Sun, FQ; Wang, QS, AMINO ACIDS,50, 5, 547-556, DOI: 10.1007/s00726-018-2547-1, MAY 2018
Tauroursodeoxycholic Acid	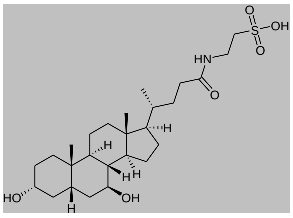	**1)** Tauroursodeoxycholic Acid Improves Motor Symptoms in a Mouse Model of Parkinson's Disease, Rosa, AI; Duarte-Silva, S; Silva-Fernandes, A; Nunes, MJ; Carvalho, AN; Rodrigues, E; Gama, MJ; Rodrigues, CMP (Pereira Rodrigues, Cecilia Maria); Maciel, P; Castro-Caldas, M, MOLECULAR NEUROBIOLOGY,55, 12, 9139-9155, DOI: 10.1007/s12035-018-1062-4, DEC 2018, **2)** Nrf2 activation by tauroursodeoxycholic acid in experimental models of Parkinson's disease, Moreira, S; Fonseca, I; Nunes, MJ; Rosa, A; Lemos, L; Rodrigues, E; Carvalho, AN; Outeiro, TF; Rodrigues, CMP (Pereira Rodrigues, Cecilia Maria); Gama, MJ et al, EXPERIMENTAL NEUROLOGY,295, 77-87, DOI: 10.1016/j.expneurol.2017.05.009, SEP 2017

Taxifolin	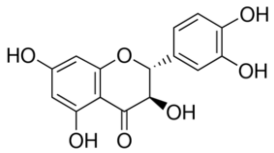	Flavonoids and its Neuroprotective Effects on Brain Ischemia and Neurodegenerative Diseases, Putteeraj, M; Lim, WL; Teoh, SL; Yahaya, MF, CURRENT DRUG TARGETS,19, 14, 1710-1720, DOI: 10.2174/1389450119666180326125252, 2018, **Document Type:Review**
Tectorigenin	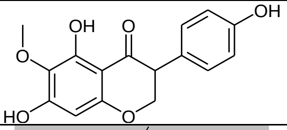	The Anti-neuroinflammatory Activity of Tectorigenin Pretreatment via Downregulated NF-kappa B and ERK/JNK Pathways in BV-2 Microglial and Microglia Inactivation in Mice With Lipopolysaccharide, Lim, HS; Kim, YJ; Kim, BY; Park, G; Jeong, SJ, FRONTIERS IN PHARMACOLOGY,9, Article Number: 462, DOI: 10.3389/fphar.2018.00462, MAY 9 2018
Telmisartan	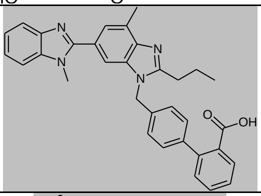	Alpha-Synuclein Glycation and the Action of Anti-Diabetic Agents in Parkinson's Disease, Konig, A; Vicente Miranda, H; Outeiro, TF, JOURNAL OF PARKINSONS DISEASE, 8, 1, 33-43, DOI: 10.3233/JPD-171285, 2018, **Document Type:Review**
Tenoxicam	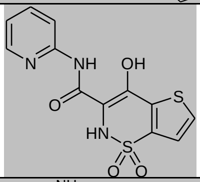	Oxicam-derived non-steroidal anti-inflammatory drugs suppress 1-methyl-4-phenyl pyridinium-induced cell death via repression of endoplasmic reticulum stress response and mitochondrial dysfunction in SH-SY5Y cells, Omura, T; Sasaoka, M; Hashimoto, G; Imai, S; Yamamoto, J; Sato, Y; Nakagawa, S; Yonezawa, A; Nakagawa, T; Yano, I et al, BIOCHEMICAL AND BIOPHYSICAL RESEARCH COMMUNICATIONS,503, 4, 2963-2969, DOI: 10.1016/j.bbrc.2018.08.078, SEP 18 2018
Terazosin	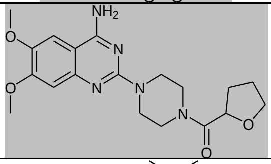	Virtual Screening against Phosphoglycerate Kinase 1 in Quest of Novel Apoptosis Inhibitors, Xia, J; Feng, B; Shao, QH; Yuan, YH; Wang, XS; Chen, NH; Wu, S, MOLECULES,22, 6, Article Number: 1029, DOI: 10.3390/molecules22061029, JUN 2017
Tetracycline	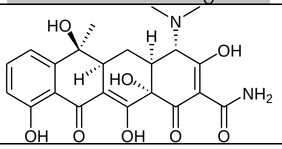	Tetracycline repurposing in neurodegeneration: focus on Parkinson's disease, Bortolanza, M; Nascimento, GC; Socias, SB; Ploper, D; Chehin, RN; Raisman-Vozari, R; Del-Bel, E, JOURNAL OF NEURAL TRANSMISSION,125, 10, 1403-1415, DOI: 10.1007/s00702-018-1913-1, OCT 2018, **Document Type:Review**.
Tetraethylammonium	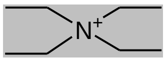	Blockade of fast A-type and TEA-sensitive potassium channels provide an antiparkinsonian effect in a 6-OHDA animal model, Haghdoost-Yazdi, H; Piri, H; Najafipour, R; Faraji, A; Fraidouni, N; Dargahi, T; Heidari, MA, NEUROSCIENCES,22, 1, 44-50, DOI: 10.17712/nsj.2017.1.20160266, JAN 2017
Tetramethylpyrazine	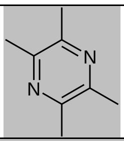	Recent advances in discovery and development of natural products as source for anti-Parkinson's disease lead compounds, Zhang, HJ; Bai, L; He, J; Zhong, L; Duan, XM; Ouyang, L; Zhu, YX; Wang, T; Shi, JY; Zhang, YW, EUROPEAN JOURNAL OF MEDICINAL CHEMISTRY,141, 257-272, DOI: 10.1016/j.ejmech.2017.09.068, DEC 1 2017, **Document Type:Review**
2,3,5,4'-Tetrahydroxystilbene-2-O-beta-D-glucoside	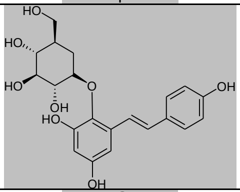	Biological Effects of Tetrahydroxystilbene Glucoside: An Active Component of a Rhizome Extracted from Polygonum multiflorum, Zhang, LL; Chen, JZ, OXIDATIVE MEDICINE AND CELLULAR LONGEVITY, Article Number: 3641960, DOI: 10.1155/2018/3641960, 2018, **Document Type:Review**
2, 3, 4, 4 – Tetramethyl – 5 - methylencyclopent-2-enone	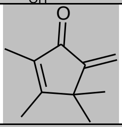	Application of Monoterpenoids and their Derivatives for Treatment of Neurodegenerative Disorders, Volcho, KP; Laev, SS; Ashraf, GM; Aliev, G; Salakhutdinov, NF, CURRENT MEDICINAL CHEMISTRY,25, 39, 5327-5346, DOI: 10.2174/0929867324666170112101837, 2018, **Document Type:Review**
THC (Delta9-Tetrahydrocannabinol)	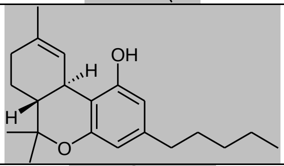	THC Exerts Neuroprotective Effect in Glutamate-affected Murine Primary Mesencephalic Cultures Through Restoring Mitochondrial Membrane Potential and Anti-apoptosis Involving CB1 Receptor-dependent Mechanism, Nguyen, CH; Krewenka, C; Radad, K; Kranner, B; Huber, A; Duvigneau, JC; Miller, I; Moldzio, R, PHYTOTHERAPY RESEARCH,30, 12, 2044-2052, DOI: 10.1002/ptr.5712, DEC 2016
Theophylline	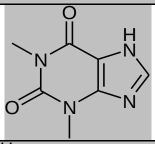	Advances in non-dopaminergic treatments for Parkinson's disease. Stayte, S; Vissel, B; FRONTIERS IN NEUROSCIENCE, 8, Article Number: 113, DOI: 10.3389/fnins.2014.00113, MAY 22 2014, **Document Type: Review**
Thiamine	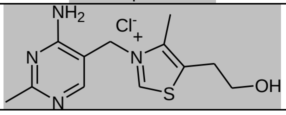	Alpha-Synuclein Glycation and the Action of Anti-Diabetic Agents in Parkinson's Disease, Konig, A; Vicente Miranda, H; Outeiro, TF, JOURNAL OF PARKINSONS DISEASE, 8, 1, 33-43, DOI: 10.3233/JPD-171285, 2018, **Document Type:Review**

5-Thienyl-substituted 2-aminobenzamide-type	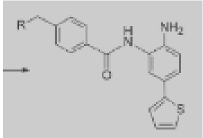	New 5-Aryl-Substituted 2-Aminobenzamide-Type HDAC Inhibitors with a Diketopiperazine Group and Their Ameliorating Effects on Ischemia-Induced Neuronal Cell Death, Hirata, Y; Sasaki, T; Kanki, H; Choong, CJ; Nishiyama, K; Kubo, G; Hotei, A; Taniguchi, M; Mochizuki, H; Uesato, S, SCIENTIFIC REPORTS,8, Article Number: 1400, DOI: 10.1038/s41598-018-19664-9, JAN 23 2018
Thymohydroquinone	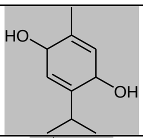	Application of Monoterpenoids and their Derivatives for Treatment of Neurodegenerative Disorders, Volcho, KP; Laev, SS; Ashraf, GM; Aliev, G; Salakhutdinov, NF, CURRENT MEDICINAL CHEMISTRY,25, 39, 5327-5346, DOI: 10.2174/0929867324666170112101837, 2018, **Document Type:Review**
Thymol	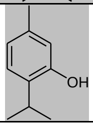	Application of Monoterpenoids and their Derivatives for Treatment of Neurodegenerative Disorders, Volcho, KP; Laev, SS; Ashraf, GM; Aliev, G; Salakhutdinov, NF, CURRENT MEDICINAL CHEMISTRY,25, 39, 5327-5346, DOI: 10.2174/0929867324666170112101837, 2018, **Document Type:Review**
Thymoquinone	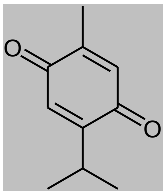	**1)** Application of Monoterpenoids and their Derivatives for Treatment of Neurodegenerative Disorders, Volcho, KP; Laev, SS; Ashraf, GM; Aliev, G; Salakhutdinov, NF, CURRENT MEDICINAL CHEMISTRY,25, 39, 5327-5346, DOI: 10.2174/0929867324666170112101837, 2018, **Document Type:Review, 2)** A Review on Possible Therapeutic Effect of Nigella sativa and Thymoquinone in Neurodegenerative Diseases, Samarghandian, S; Farkhondeh, T; Samini, F, CNS & NEUROLOGICAL DISORDERS-DRUG TARGETS,17, 6, 412-420, Article Number: PMID 29962349, DOI: 10.2174/1871527317666180702101455, 2018, **Document Type:Review**, **3)** Thymoquinone exerts neuroprotective effect in animal model of Parkinson's disease, Ebrahimi, SS; Oryan, S; Izadpanah, E; Hassanzadeh, K, TOXICOLOGY LETTERS,276, 108-114, DOI: 10.1016/j.toxlet.2017.05.018, JUL 5 2017, **4)** A review of Neuropharmacology Effects of Nigella sativa and Its Main Component, Thymoquinone, Javidi, S; Razavi, BM; Hosseinzadeh, H, PHYTOTHERAPY RESEARCH,30, 8, 1219-1229, DOI: 10.1002/ptr.5634, AUG 2016, **Document Type:Review**, **5) et cetera**
Thiol peroxidases		Thiol peroxidases ameliorate LRRK2 mutant-induced mitochondrial and dopaminergic neuronal degeneration in Drosophila, Angeles, DC; Ho, P; Chua, LL; Wang, C; Yap, YW; Ng, C; Zhou, ZD; Lim, KL; Wszolek, ZK; Wang, HY et al, HUMAN MOLECULAR GENETICS,23, 12, 3157-3165, DOI: 10.1093/hmg/ddu026, JUN 15 2014
α-Tocopherol	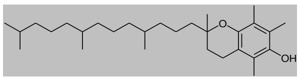	The neuroprotective effects of tocotrienol rich fraction and alpha tocopherol against glutamate injury in astrocytes, Selvaraju, TR; Khaza'ai, H; Vidyadaran, S; Abd Mutalib, MS; Vasudevan, R, BOSNIAN JOURNAL OF BASIC MEDICAL SCIENCES,14, 4, 195-204, DOI: 10.17305/bjbms.2014.4.91, NOV 2014
Tocotrienols (vitamin E)	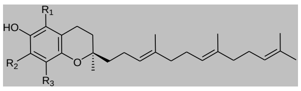	The neuroprotective effects of tocotrienol rich fraction and alpha tocopherol against glutamate injury in astrocytes, Selvaraju, TR; Khaza'ai, H; Vidyadaran, S; Abd Mutalib, MS; Vasudevan, R, BOSNIAN JOURNAL OF BASIC MEDICAL SCIENCES,14, 4, 195-204, DOI: 10.17305/bjbms.2014.4.91, NOV 2014
Tolcapone (ASI-7)	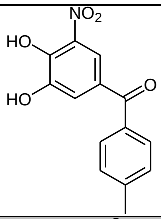	**1)** Advances in non-dopaminergic treatments for Parkinson's disease. Stayte, S; Vissel, B; FRONTIERS IN NEUROSCIENCE, 8, Article Number: 113, DOI: 10.3389/fnins.2014.00113, MAY 22 2014, **Document Type: Review**, **2)** Development of Multifunctional Molecules as Potential Therapeutic Candidates for Alzheimer's Disease, Parkinson's Disease, and Amyotrophic Lateral Sclerosis in the Last Decade. Savelieff, MG; Nam, G; Kang, J; Lee, HJ; Lee, M; Lim, MH. CHEMICAL REVIEWS, 119, 2, 1221-1322, DOI: 10.1021/acs.chemrev.8b00138, JAN 23 2019, **Document Type:Review**
Topiramate	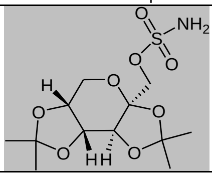	Advances in Drug Development for Parkinson's Disease: Present Status. Reddy, DH; Misra, S; Medhi, B. PHARMACOLOGY, 93, 5-6, 260-271, DOI: 10.1159/000362419, 2014, **Document Type:Review**
Tozadenant	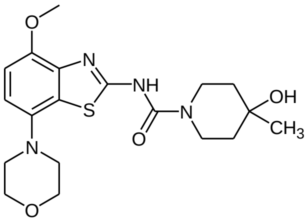	**1)** Current approaches to the treatment of Parkinson's Disease, Ellis, JM; Fell, MJ, BIOORGANIC & MEDICINAL CHEMISTRY LETTERS, 27, 18, 4247-4255, DOI: 10.1016/j.bmcl.2017.07.075, Published: SEP 15 2017, **2)** Adenosine A(2A) Receptor Antagonists in Parkinson's Disease: Progress in Clinical Trials from the Newly Approved Istradefylline to Drugs in Early Development and Those Already Discontinued, Pinna, A, CNS DRUGS,28, 5, 455-474, DOI: 10.1007/s40263-014-0161-7, MAY 2014, **Document Type:Review**, **3)** Unprecedented Therapeutic Potential with a Combination of A(2A)/NR2B Receptor Antagonists as Observed in the 6-OHDA Lesioned Rat Model of Parkinson's Disease, Michel, A; Downey, P; Nicolas, JM; Scheller, D, PLOS ONE,9, 12, Article Number: e114086, DOI: 10.1371/journal.pone.0114086, DEC 16 2014
TPNA10168	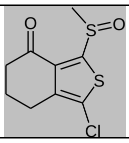	Neuroprotective effect of an Nrf2-ARE activator identified from a chemical library on dopaminergic neurons, Izumi, Y; Kataoka, H; Inose, Y; Akaike, A; Koyama, Y; Kume, T, EUROPEAN JOURNAL OF PHARMACOLOGY,818, 470-479, DOI: 10.1016/j.ejphar.2017.11.023, JAN 5 2018

Transilitin	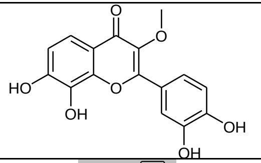	Exploring the Structural Diversity in Inhibitors of alpha-Synuclein Amyloidogenic Folding, Aggregation, and Neurotoxicity, Das, S; Pukala, TL; Smid, SD, FRONTIERS IN CHEMISTRY,6, Article Number: 181, DOI: 10.3389/fchem.2018.00181, MAY 25 2018
Traxoprodil	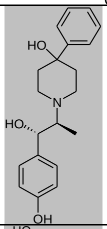	Advances in non-dopaminergic treatments for Parkinson's disease. Stayte, S; Vissel, B; FRONTIERS IN NEUROSCIENCE, 8, Article Number: 113, DOI: 10.3389/fnins.2014.00113, MAY 22 2014, **Document Type: Review**
Trehalose	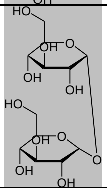	Is trehalose an autophagic inducer? Unraveling the roles of non-reducing disaccharides on autophagic flux and alpha-synuclein aggregation, Yoon, S; Cho, ED; Ahn, WJ; Lee, KW; Lee, SJ; Lee, HJ, CELL DEATH & DISEASE,8, Article Number: e3091, DOI: 10.1038/cddis.2017.501, OCT 2017
O-Trensox	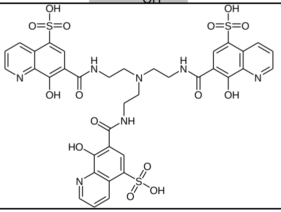	Iron Chelating Strategies in Systemic Metal Overload, Neurodegeneration and Cancer. Gumienna-Kontecka, E; Pyrkosz-Bulska, M; Szebesczyk, A; Ostrowska, M. CURRENT MEDICINAL CHEMISTRY, 21, 33, 3741-3767, DOI: 10.2174/0929867321666140706143402, 2014. **Document Type: Review**
12-O-n-deca-2,4,6-Trienoyl-phorbol-(13)-acetate	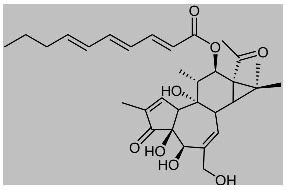	**1)** Daphnane and Phorbol Diterpenes, Anti-neuroinflammatory Compounds with Nurr1 Activation from the Roots and Stems of Daphne genkwa, Han, BS; Van Minh, N; Choi, HY; Byun, JS; Kim, WG, BIOLOGICAL & PHARMACEUTICAL BULLETIN,40, 12, 2205-2212, DEC 2017, **2)** Diterpenes: Advances in Neurobiological Drug Research, Islam, MT; da Silva, CB; de Alencar, MVOB (Oliveira Barros de Alencar, Marcus Vinicius); Paz, MFCJ; Almeida, FRD; Melo-Cavalcante, AAD (de Carvalho Melo-Cavalcante, Ana Amelia), PHYTOTHERAPY RESEARCH,30, 6, 915-928, DOI: 10.1002/ptr.5609, JUN 2016, **Document Type: Review**
Trihexyphenidyl	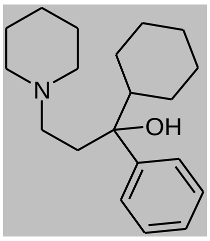	**1)** Current approaches to the treatment of Parkinson's Disease, Ellis, JM; Fell, MJ, BIOORGANIC & MEDICINAL CHEMISTRY LETTERS, 27, 18, 4247-4255, DOI: 10.1016/j.bmcl.2017.07.075, Published: SEP 15 2017**, 2)** Pharmacological characterization of nicotine-induced tremor: Responses to anti-tremor and anti-epileptic agents, Kunisawa, N; Shimizu, S; Kato, M; Iha, HA; Iwai, C; Hashimura, M; Ogawa, M; Kawaji, S; Kawakita, K; Abe, K et al, JOURNAL OF PHARMACOLOGICAL SCIENCES,137, 2, 162-169, DOI: 10.1016/j.jphs.2018.05.007, JUN 2018, **3)** Prevalence and Correlates of Anti-Parkinson Drug Use in a Nationally Representative Sample, Dahodwala, N; Willis, AW; Li, PX; Doshi, JA; MOVEMENT DISORDERS CLINICAL PRACTICE, 4, 3, 335-341, DOI: 10.1002/mdc3.12422, MAY-JUN 2017, **4)** Advances in non-dopaminergic treatments for Parkinson's disease. Stayte, S; Vissel, B; FRONTIERS IN NEUROSCIENCE, 8, Article Number: 113, DOI: 10.3389/fnins.2014.00113, MAY 22 2014, **Document Type: Review, 5)** Cholinergic manipulation of motor disability and L-DOPA-induced dyskinesia in 1-methyl-4-phenyl-1,2,3,6-tetrahydropyridine -treated common marmosets, Jackson, MJ; Swart, T; Pearce, RKB; Jenner, P, JOURNAL OF NEURAL TRANSMISSION,121, 2, 163-169, DOI: 10.1007/s00702-013-1082-1, FEB 2014
2', 3', 4' Trihydroxyflavone	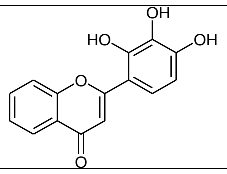	Exploring the Structural Diversity in Inhibitors of alpha-Synuclein Amyloidogenic Folding, Aggregation, and Neurotoxicity, Das, S; Pukala, TL; Smid, SD, FRONTIERS IN CHEMISTRY,6, Article Number: 181, DOI: 10.3389/fchem.2018.00181, MAY 25 2018
(R)-(3,5,6-Trimethylpyrazinyl) methyl-2-acetoxy-3-(3,4-diacetoxyphenyl) propanoate - ADTM	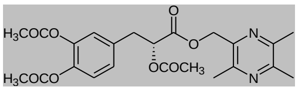	A New Danshensu Derivative Protects Against 6-Hydroxydopamine-Induced Neurotoxicity In Vitro and In Vivo, Cui, GZ; Shan, LC; Chen, Y; Zhou, HF; Wang, YQ; Lee, SMY, AMERICAN JOURNAL OF CHINESE MEDICINE,44, 7, 1349-1361, DOI: 10.1142/S0192415X16500750, 2016
Triptolide	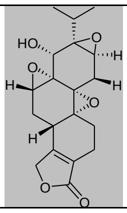	Recent advances in discovery and development of natural products as source for anti-Parkinson's disease lead compounds, Zhang, HJ; Bai, L; He, J; Zhong, L; Duan, XM; Ouyang, L; Zhu, YX; Wang, T; Shi, JY; Zhang, YW, EUROPEAN JOURNAL OF MEDICINAL CHEMISTRY,141, 257-272, DOI: 10.1016/j.ejmech.2017.09.068, DEC 1 2017, **Document Type:Review**

2,3,3-Trisphosphonate	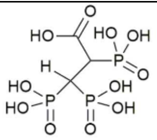	Neuroprotective Effects of Etidronate and 2,3,3-Trisphosphonate Against Glutamate-Induced Toxicity in PC12 Cells, Li, W; Cheong, YK; Wang, H; Ren, GG; Yang, Z, NEUROCHEMICAL RESEARCH,41, 4, 844-854, DOI: 10.1007/s11064-015-1761-4, APR 2016
Umbelliferone	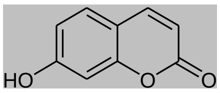	Recent advances in discovery and development of natural products as source for anti-Parkinson's disease lead compounds, Zhang, HJ; Bai, L; He, J; Zhong, L; Duan, XM; Ouyang, L; Zhu, YX; Wang, T; Shi, JY; Zhang, YW, EUROPEAN JOURNAL OF MEDICINAL CHEMISTRY,141, 257-272, DOI: 10.1016/j.ejmech.2017.09.068, DEC 1 2017, **Document Type:Review**
Ursolic acid	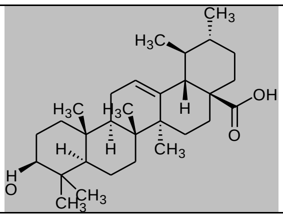	**1)** Ursolic acid derivatives for pharmaceutical use: a patent review, Hussain, H; Green, IR; Ali, I; Khan, IA; Ali, Z; Al-Sadi, AM; Ahmed, I, EXPERT OPINION ON THERAPEUTIC PATENTS,27, 9, 1061-1072, DOI: 10.1080/13543776.2017.1344219, 2017, **Document Type:Review**, **2)** Ursolic acid attenuates oxidative stress in nigrostriatal t and improves neurobehavioral activity in MPTP-induced Parkinsonian mouse model, Rai, SN; Yadav, SK; Singh, D; Singh, SP, JOURNAL OF CHEMICAL NEUROANATOMY,71, 41-49, DOI: 10.1016/j.jchemneu.2015.12.002, JAN 2016
UWA-121	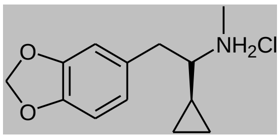	UWA-121, a mixed dopamine and serotonin re-uptake inhibitor, enhances L-DOPA anti-parkinsonian action without worsening dyskinesia or psychosis-like behaviours in the MPTP-lesioned common marmoset, Huot, P; Johnston, TH; Lewis, KD; Koprich, JB; Reyes, MG; Fox, SH; Piggott, MJ; Brotchie, JM, NEUROPHARMACOLOGY,82, 76-87, DOI: 10.1016/j.neuropharm.2014.01.012, JUL 2014
V81444	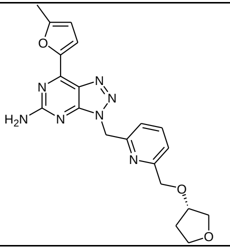	Adenosine A(2A) Receptor Antagonists in Parkinson's Disease: Progress in Clinical Trials from the Newly Approved Istradefylline to Drugs in Early Development and Those Already Discontinued, Pinna, A, CNS DRUGS,28, 5, 455-474, DOI: 10.1007/s40263-014-0161-7, MAY 2014, **Document Type: Review**
(Val (8))GLP-1-Glu-PAL		Neuroprotective role of (Val (8))GLP-1-Glu-PAL in an in vitro model of Parkinsons disease, Li, L; Liu, K; Zhao, J; Holscher, C; Li, GL; Liu, YZ, NEURAL REGENERATION RESEARCH,11, 2, 326-331, DOI: 10.4103/1673-5374.177742, FEB 2016
Valsartan	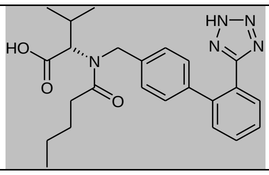	Repurposing of Copper (II)-chelating Drugs for the Treatment of Neurodegenerative Diseases. Lanza, V; Milardi, D; Di Natale, G; Pappalardo, G. CURRENT MEDICINAL CHEMISTRY, 25, 4, 525-539, DOI: 10.2174/0929867324666170518094404, 2018, **Document Type: Review**
Valproic acid or sodium valproate	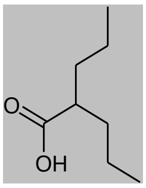	**1)** Valproate is protective against 6-OHDA-induced dopaminergic neurodegeneration in rodent midbrain: A potential role of BDNF up-regulation, Lai, CL; Lu, CC; Lin, HC; Sung, YF; Wu, YP; Hong, JS; Peng, GS, JOURNAL OF THE FORMOSAN MEDICAL ASSOCIATION,118, 1, 420-428 Part: 3, DOI: 10.1016/j.jfma.2018.06.017, JAN 2019, **2)** Valproic acid protects against MPP+-mediated neurotoxicity in SH-SY5Y Cells through autophagy, Zhang, Y; Wu, JY; Weng, LH; Li, XX; Yu, LJ; Xu, Y, NEUROSCIENCE LETTERS,638, 60-68, DOI: 10.1016/j.neulet.2016.12.017, JAN 18 2017, **3)** Neurorestoration induced by the HDAC inhibitor sodium valproate in the lactacystin model of Parkinson's is associated with histone acetylation and up-regulation of neurotrophic factors, Harrison, IF; Crum, WR; Vernon, AC; Dexter, DT, BRITISH JOURNAL OF PHARMACOLOGY,172, 16, 4200-4215, DOI: 10.1111/bph.13208, AUG 2015, **4) et cetera**
Vanillin (4-hydroxy-3-methoxybenzaldehyde)	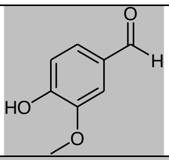	Vanillin Attenuated Behavioural Impairments, Neurochemical Deficts, Oxidative Stress and Apoptosis Against Rotenone Induced Rat Model of Parkinson's Disease, Dhanalakshmi, C; Janakiraman, U; Manivasagam, T; Thenmozhi, AJ; Essa, MM; Kalandar, A; Khan, MAS; Guillemin, GJ, NEUROCHEMICAL RESEARCH,41, 8, 1899-1910, DOI: 10.1007/s11064-016-1901-5, AUG 2016
Varenicline	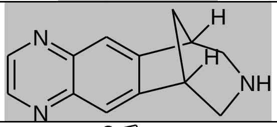	Therapeutic strategies for Parkinson disease: beyond dopaminergic drugs. Charvin, D; Medori, R; Hauser, RA; Rascol, O. NATURE REVIEWS DRUG DISCOVERY, 17, 11, 804-822, DOI: 10.1038/nrd.2018.136, NOV 2018, **Document Type: Review**
VAS3947	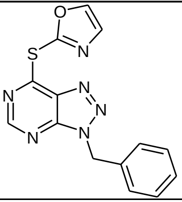	Redox-based therapeutics in neurodegenerative disease. McBean, GJ; Lopez, MG; Wallner, FK. BRITISH JOURNAL OF PHARMACOLOGY, 174, 12, 1750-1770, DOI: 10.1111/bph.13551, JUN 2017**, Document Type: Review**

VAS2870	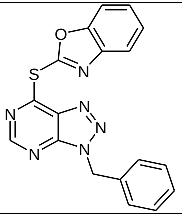	Redox-based therapeutics in neurodegenerative disease. McBean, GJ; Lopez, MG; Wallner, FK. BRITISH JOURNAL OF PHARMACOLOGY, 174, 12, 1750-1770, DOI: 10.1111/bph.13551, JUN 2017, **Document Type: Review**
Vasoactive intestinal peptide		Protective Effects of Poly Cyanoacrylate Nanoparticles Containing Vasoactive Intestinal Peptide Against 6-Hydroxydopamine-Induced Neurotoxicity In Vitro, Xu, ZR; Wang, WF; Liang, XF; Liu, ZH; Liu, Y; Lin, L; Zhu, X, JOURNAL OF MOLECULAR NEUROSCIENCE,55, 4, 854-864, DOI: 10.1007/s12031-014-0438-9, APR 2015
Venglustat	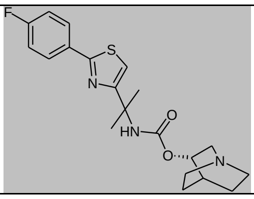	Therapeutic strategies for Parkinson disease: beyond dopaminergic drugs.Charvin, D; Medori, R; Hauser, RA; Rascol, O. NATURE REVIEWS DRUG DISCOVERY, 17, 11, 804-822, DOI: 10.1038/nrd.2018.136, NOV 2018, **Document Type:Review**
Verbascoside	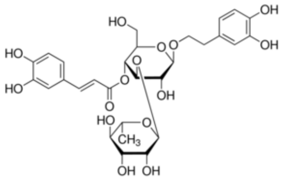	**1)** Natural polyphenols effects on protein aggregates in Alzheimer's and Parkinson's prion-like diseases, Freyssin, A; Page, G; Fauconneau, B; Bilan, AR, NEURAL REGENERATION RESEARCH,13, 6, 955-961, DOI: 10.4103/1673-5374.233432, JUN 2018, **Document Type:Review**, **2)** Polyphenols in Parkinson's Disease: A Systematic Review of In Vivo Studies, Kujawska, M; Jodynis-Liebert, J, NUTRIENTS,10, 5, Article Number: 642, DOI: 10.3390/nu10050642, MAY 2018, **Document Type:Review**, **3)** Novel tactics for neuroprotection in Parkinson's disease: Role of antibiotics, polyphenols and neuropeptides, Reglodi, D; Renaud, J; Tamas, A; Tizabi, Y; Socias, SB; Del-Bel, E; Raisman-Vozari, R, PROGRESS IN NEUROBIOLOGY,155, 120-148 Special : SI, DOI: 10.1016/j.pneurobio.2015.10.004, AUG 2017, **Document Type:Review**, **4) et cetera**
(-) -Verbenone	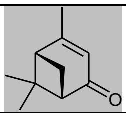	Application of Monoterpenoids and their Derivatives for Treatment of Neurodegenerative Disorders, Volcho, KP; Laev, SS; Ashraf, GM; Aliev, G; Salakhutdinov, NF, CURRENT MEDICINAL CHEMISTRY,25, 39, 5327-5346, DOI: 10.2174/0929867324666170112101837, 2018, **Document Type:Review**
Vildagliptin	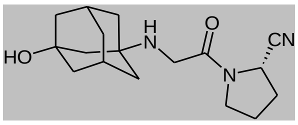	**1)** Parkinson's Disease, Diabetes and Cognitive Impairment, Ashraghi, MR; Pagano, G; Polychronis, S; Niccolini, F; Politis, M, RECENT PATENTS ON ENDOCRINE METABOLIC & IMMUNE DRUG DISCOVERY,10, 1, 11-21, DOI: 10.2174/1872214810999160628105549, 2016, **Document Type: Review**, **2)** Neuroprotective effects of vildagliptin in rat rotenone Parkinson's disease model: role of RAGE-NF kappa B and Nrf2 antioxidant signaling pathways, Abdelsalam, RM; Safar, MM, JOURNAL OF NEUROCHEMISTRY,133, 5, 700-707, DOI: 10.1111/jnc.13087, JUN 2015
Viniferin	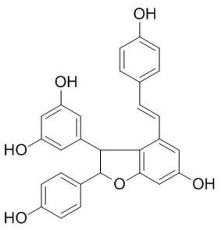	**1)** Natural polyphenols effects on protein aggregates in Alzheimer's and Parkinson's prion-like diseases, Freyssin, A; Page, G; Fauconneau, B; Bilan, AR, NEURAL REGENERATION RESEARCH,13, 6, 955-961, DOI: 10.4103/1673-5374.233432, JUN 2018, **Document Type:Review**, **2)** Polyphenols in Parkinson's Disease: A Systematic Review of In Vivo Studies, Kujawska, M; Jodynis-Liebert, J, NUTRIENTS,10, 5, Article Number: 642, DOI: 10.3390/nu10050642, MAY 2018, **Document Type:Review**, **3)** Novel tactics for neuroprotection in Parkinson's disease: Role of antibiotics, polyphenols and neuropeptides, Reglodi, D; Renaud, J; Tamas, A; Tizabi, Y; Socias, SB; Del-Bel, E; Raisman-Vozari, R, PROGRESS IN NEUROBIOLOGY,155, 120-148 Special : SI, DOI: 10.1016/j.pneurobio.2015.10.004, AUG 2017, **Document Type:Review**, **4) et cetera**
Vinpocetine	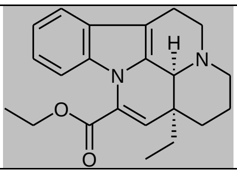	Protective effect of vinpocetine against neurotoxicity of manganese in adult male rats, Nadeem, RI; Ahmed, HI; El-Sayeh, BM, NAUNYN-SCHMIEDEBERGS ARCHIVES OF PHARMACOLOGY,391, 7, 729-742, DOI: 10.1007/s00210-018-1498-0, JUL 2018
Vinyl sulfones	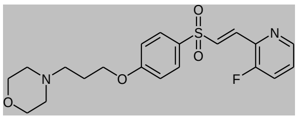	**1)** Optimization of Vinyl Sulfone Derivatives as Potent Nuclear Factor Erythroid 2-Related Factor 2 Activators for Parkinson's Disease Therapy, Choi, JW; Kim, S; Park, JH; Kim, HJ; Shin, SJ; Kim, JW; Woo, SY; Lee, C; Han, SM; Lee, J et al, JOURNAL OF MEDICINAL CHEMISTRY,62, 2, 811-830, DOI: 10.1021/acs.jmedchem.8b01527, JAN 24 2019, **2)** Pharmacophore model prediction, 3D-QSAR and molecular docking studies on vinyl sulfones targeting Nrf2-mediated gene transcription intended for anti-Parkinson drug design, Athar, M; Lone, MY; Khedkar, VM; Jha, PC, JOURNAL OF BIOMOLECULAR STRUCTURE & DYNAMICS,34, 6, 1282-1297, DOI: 10.1080/07391102.2015.1077343, 2016, **3)** Discovery of Vinyl Sulfones as a Novel Class of Neuroprotective Agents toward Parkinson's Disease Therapy, Woo, SY; Kim, JH; Moon, MK; Han, SH; Yeon, SK; Choi, JW; Jang, BK; Song, HJ; Kang, YG; Kim, JW et al, JOURNAL OF MEDICINAL CHEMISTRY,57, 4, 1473-1487, DOI: 10.1021/jm401788m, FEB 27 2014

Vipadenant	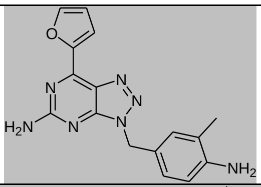	Adenosine A(2A) Receptor Antagonists in Parkinson's Disease: Progress in Clinical Trials from the Newly Approved Istradefylline to Drugs in Early Development and Those Already Discontinued, Pinna, A, CNS DRUGS,28, 5, 455-474, DOI: 10.1007/s40263-014-0161-7, MAY 2014, **Document Type:Review**
Vitamin D	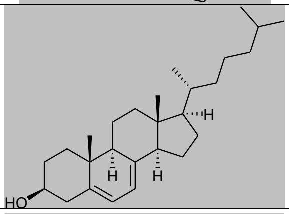	The Role of Vitamin D-3 in the Development and Neuroprotection of Midbrain Dopamine Neurons, Orme, RP; Middleditch, C; Waite, L; Fricker, RA, VITAMIN D HORMONE, Edited Litwack, G, Book Series: Vitamins and Hormones,100, 273-297, DOI: 10.1016/bs.vh.2015.10.007, 2016, **Document Type:Review**; Book Chapter
VU0361737	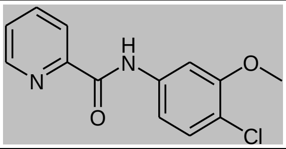	Neuroprotective effects of metabotropic glutamate receptor group II and III activators against MPP(+)-induced cell death in human neuroblastoma SH-SY5Y cells: The impact of cell differentiation state, Jantas, D; Greda, A; Golda, S; Korostynski, M; Grygier, B; Roman, A; Pilc, A; Lason, W, NEUROPHARMACOLOGY,83, 36-53, DOI: 10.1016/j.neuropharm.2014.03.019, AUG 2014
Wikstoemia Factor M1	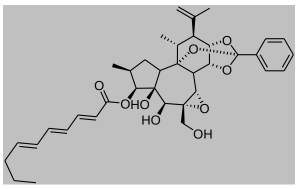	**1)** Daphnane and Phorbol Diterpenes, Anti-neuroinflammatory Compounds with Nurr1 Activation from the Roots and Stems of Daphne genkwa, Han, BS; Van Minh, N; Choi, HY; Byun, JS; Kim, WG, BIOLOGICAL & PHARMACEUTICAL BULLETIN,40, 12, 2205-2212, DEC 2017, **2)** Diterpenes: Advances in Neurobiological Drug Research, Islam, MT; da Silva, CB; de Alencar, MVOB; Paz, MFCJ; Almeida, FRD; Melo-Cavalcante, AAD, PHYTOTHERAPY RESEARCH,30, 6, 915-928, DOI: 10.1002/ptr.5609, JUN 2016, **Document Type: Review**
WIN 55,212-2	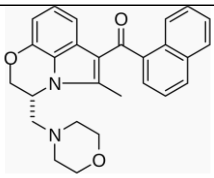	**1)** The role of cannabinoids and leptin in neurological diseases, Agar, E; ACTA NEUROLOGICA SCANDINAVICA, 132, 6, 371-380, DOI: 10.1111/ane.12411, DEC 2015, **Document Type:Review, 2)** Promising cannabinoid-based therapies for Parkinson's disease: motor symptoms to neuroprotection, More, SV; Choi, DK; MOLECULAR NEURODEGENERATION, 10, Article Number: 17, DOI: 10.1186/s13024-015-0012-0, APR 8 2015, **Document Type:Review, 3) et cetera**
WR-1065	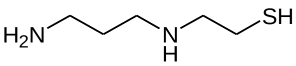	Effect of WR-1065 on 6-hydroxydopamine-induced catalepsy and IL-6 level in rats, Kheradmand, A; Nayebi, AM; Jorjani, M; Haddadi, R, IRANIAN JOURNAL OF BASIC MEDICAL SCIENCES,19, 5, 490-496, MAY 2016
Xanthine derivatives	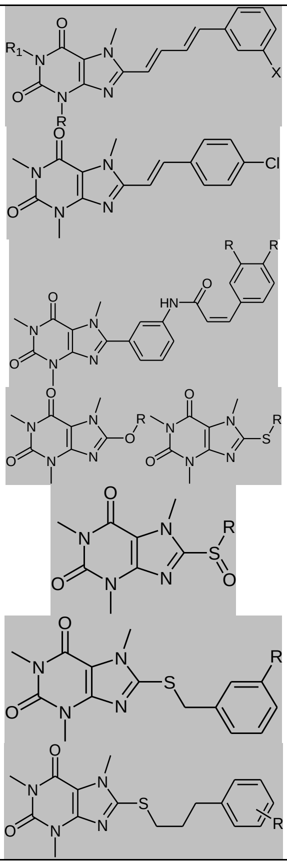	Xanthine derivatives as agents affecting non-dopaminergic neuroprotection in Parkinson`s disease., Kasabova-Angelova, A; Tzankova, D; Mitkov, J; Georgieva, M; Tzankova, V; Zlatkov, A; Kondeva-Burdina, M, Current medicinal chemistry, DOI:10.2174/0929867325666180821153316, 2018-Aug-21

Yuanhuadine	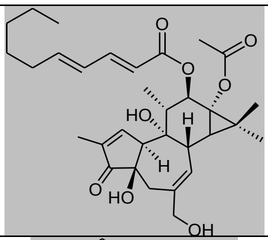	**1)** Daphnane and Phorbol Diterpenes, Anti-neuroinflammatory Compounds with Nurr1 Activation from the Roots and Stems of Daphne genkwa, Han, BS; Van Minh, N; Choi, HY; Byun, JS; Kim, WG, BIOLOGICAL & PHARMACEUTICAL BULLETIN,40, 12, 2205-2212, DEC 2017, **2)** Diterpenes: Advances in Neurobiological Drug Research, Islam, MT; da Silva, CB; de Alencar, MVOB (Oliveira Barros de Alencar, Marcus Vinicius); Paz, MFCJ; Almeida, FRD; Melo-Cavalcante, AAD (de Carvalho Melo-Cavalcante, Ana Amelia), PHYTOTHERAPY RESEARCH,30, 6, 915-928, DOI: 10.1002/ptr.5609, JUN 2016, **Document Type: Review**
Yuanhuatine	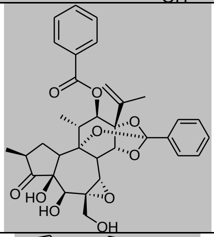	**1)** Daphnane and Phorbol Diterpenes, Anti-neuroinflammatory Compounds with Nurr1 Activation from the Roots and Stems of Daphne genkwa, Han, BS; Van Minh, N; Choi, HY; Byun, JS; Kim, WG, BIOLOGICAL & PHARMACEUTICAL BULLETIN,40, 12, 2205-2212, DEC 2017, **2)** Diterpenes: Advances in Neurobiological Drug Research, Islam, MT; da Silva, CB; de Alencar, MVOB (Oliveira Barros de Alencar, Marcus Vinicius); Paz, MFCJ; Almeida, FRD; Melo-Cavalcante, AAD (de Carvalho Melo-Cavalcante, Ana Amelia), PHYTOTHERAPY RESEARCH,30, 6, 915-928, DOI: 10.1002/ptr.5609, JUN 2016, **Document Type: Review**
Yohimbine	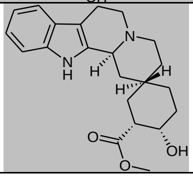	Advances in non-dopaminergic treatments for Parkinson's disease. Stayte, S; Vissel, B; FRONTIERS IN NEUROSCIENCE, 8, Article Number: 113, DOI: 10.3389/fnins.2014.00113, MAY 22 2014, **Document Type: Review**
Zonisamide	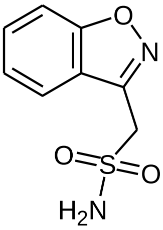	**1)** The anti-parkinsonian drug zonisamide reduces neuroinflammation: Role of microglial Na-v 1.6, Hossain, MM [1,2,3,4]; Weig, B; Reuhl, K; Gearing, M; Wu, LJ; Richardson, JR [1,2,3,4,8], EXPERIMENTAL NEUROLOGY,308, 111-119, DOI: 10.1016/j.expneurol.2018.07.005, OCT 2018, **2)** Zonisamide for DLB parkinsonism: An old drug used in a new context, Hershey, LA; Irwin, DJ, NEUROLOGY,90, 8, 349-350, DOI: 10.1212/WNL.0000000000005006, FEB 20 2018, **3)** Zonisamide inhibits monoamine oxidase and enhances motor performance and social activity, Uemura, MT; Asano, T; Hikawa, R; Yamakado, H; Takahashi, R, NEUROSCIENCE RESEARCH,124, 25-32, DOI: 10.1016/j.neures.2017.05.008, NOV 2017, **4) et cetera**
Zolpidem	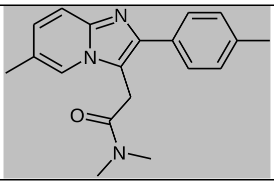	Can a Positive Allosteric Modulation of GABAergic Receptors Improve Motor Symptoms in Patients with Parkinson's Disease? The Potential Role of Zolpidem in the Treatment of Parkinson's Disease, Daniele, A; Panza, F; Greco, A; Logroscino, G; Seripa, D, PARKINSONS DISEASE, Article Number: 2531812, DOI: 10.1155/2016/2531812, 2016, **Document Type:Review**

TABLE S2

Acid-base and metal chelation properties of the compounds listed in Table 2. The formulas in the column marked "H^+" represent the acid-base species, those in the other columns are the metal-ligand complexes, and the numbers are the corresponding values of log*β* (see equation 2 in main text for the definition of *β*). If not differently specified in the notes, values have been obtained at 25 °C and at a 0.1 M (= mol/L) ionic strength, and they have been taken from the IUPAC stability constants database (Academic Software, version 5.84).

Ligand	H^+	Cu(II)	Cu(I)	Fe(III)	Fe(II)	Mn(II)	Zn(II)	Notes
7DH<br>7MH	HL 9.684<br>H_2L 14.70	CuL 12.10<br>CuL_2 23.00	(50% mix solv., 0.3 M)<br>CuL_2 14.7	FeL 13.69<br>FeL_2 26.3<br>FeL_3 36.9	(20 °C, 0.01 M)<br>FeL 8.0<br>FeL_2 15.0	MnL 7.85<br>MnL_2 14.40	ZnL 8.52<br>ZnL_2 15.84	tentative, from 8-hydroxy-quinoline, charges unknown
8A<br>8B<br>8C	HL 9.684<br>H_2L 14.70	CuL 12.10<br>CuL_2 23.00	(50% mix solv., 0.3 M)<br>CuL_2 14.7	FeL 13.69<br>FeL_2 26.3<br>FeL_3 36.9	(20 °C, 0.01 M)<br>FeL 8.0<br>FeL_2 15.0	MnL 7.85<br>MnL_2 14.40	ZnL 8.52<br>ZnL_2 15.84	tentative, from 8-hydroxy-quinoline, charges unknown
8E<br>8F	HL 4.23<br>H_2L 5.55	CuL 9.00<br>CuL_2 14.724	(23 °C, 0.0 M)<br>CuL 4.59<br>CuL_2 9.18	FeL 9.13<br>$FeH_{-1}L$ 6.96<br>$FeH_{-2}L$ 5.11<br>FeL_2 18.11<br>$FeH_{-1}L_2$ 14.74<br>$FeH_{-2}L_2$ 10.76	FeL 3.98<br>FeL_2 9.09<br>FeL_3 12.63	MnL 2.62<br>MnL_2 4.62<br>MnL_3 5.72	ZnL 5.13<br>ZnL_2 9.50	tentative, from 2,2'-bipyridine, charges unknown
N-Acetyl cysteine	HL^- 9.62<br>H_2L 12.70	$CuHL^+$ 13.35<br>CuL 6.64<br>CuL_2^{2-} 12.70		FeL^+ 10.58<br>FeL_2^- 18.80		$MnHL^+$ 12.66<br>MnL 3.64<br>MnL_2^{2-} 7.47	ZnL 4.90<br>$ZnHL_2^-$ 18.39<br>ZnL_2^{2-} 11.48<br>$ZnH_{-1}L_2^{3-}$ 2.71	Fe(III) data (0.12 M) from [Guzeloglu 1998], Cu(II) and Mn(II) data (37 °C, 0.15 M) from [Santoso 2014]
ACPT-I	HL 10.31<br>H_2L 12.71						ZnL 4.76<br>ZnL_2 9.16	tentative, from 1-Amino-cyclopentane-carboxylic acid (20 °C), charges unknown
	HL 9.60<br>H_2L 11.93	CuL 8.38<br>CuL_2 15.25	(0.3 M)<br>CuL_2 10.0	FeL 10.83<br>FeL_2 20.48	FeL 4.13<br>FeL_2 7.65	MnL 2.85		tentative, from glycine, charges unknown
ADX88178	HL 9.74<br>H_2L 16.30	CuL 8.77<br>CuL_2 14.82						tentative, from 2,2'-Aminoethyl-pyrrolidine (30 °C, → 0 M), charges unknown
	HL 10.84<br>H_2L 19.96		(20 °C, 1 M)<br>CuL 11.92<br>CuL_2 15.12					tentative, from 3-amino-propane-1-thiol, charges unknown
Alaternin	(0.0 M)<br>HL 12.46<br>H_2L 20.76	CuL 6.45<br>CuL_2 12.30					ZnL 5.80	tentative, from 1,8-dihydroxy-anthraquinone (100% org. solv.), charges unknown
Alvespimycin	(0.0 M)<br>HL^+ 9.94<br>H_2L^{2+} 16.80	(0.5 M)<br>CuL^{2+} 10.60<br>CuL_2^{2+} 19.75	(0.3 M)<br>CuL_2^+ 11.4		FeL^{2+} 4.34<br>FeL_2^{2+} 7.65<br>FeL_3^{2+} 9.70	MnL^{2+} 2.77<br>MnL_2^{2+} 4.87<br>MnL_3^{2+} 5.79	(0.0 M)<br>ZnL^{2+} 5.77<br>ZnL_2^{2+} 10.83	tentative, from 1,2-diamino-ethane (1.0 M)
AM-251	HL 10.77	CuL 9.77					(20 °C)	tentative, from 1,3-diamino-

	H_2L 19.62	CuL_2 16.94					ZnHL 14.96<br>$ZnHL_2$ 20.22	propane, charges unknown
Ambroxol	HL^+ 10.70<br>H_2L^{2+} 18.3	CuL^+ 11.95		FeL^{2+} 18.54		MnL^+ 9.32	ZnL^+ 10.08<br>ZnL_2 18.99<br>ZnL_3^- 24.94	data from [Naggar 2018]
3-(7-Amino-5-(cyclohexylamino)-[1,2,4]triazolo[1,5-a][1,3,5]triazin-2-yl)-2-cyanoacrylamide	HL 9.35<br>H_2L 14.08	CuL 9.22<br>CuL_2 17.17						tentative, from 4(5)-Amino-methyl-imidazole, charges unknown
	(0.0 M)<br>HL 9.94<br>H_2L 16.80		(0.3 M)<br>CuL_2 11.4		FeL 4.34<br>FeL_2 7.65<br>FeL_3 9.70	MnL 2.77<br>MnL_2 4.87<br>MnL_3 5.79	(0.0 M)<br>ZnL 5.77<br>ZnL_2 10.83	tentative, from 1,2-diamino-ethane (1.0 M), charges unknown
Aminothiazoles derivatives as SUMOylation activators	HL 8.93<br>H_2L 10.87	CuL 9.69<br>CuL_2 16.90	(50% mix solv.)<br>CuL_2 10.66		(0.15 M)<br>FeL 4.105<br>FeL_2 7.441<br>FeL_3 10.116	(20 °C)<br>MnL 2.66	ZnL 5.2	tentative, from 2-(amino-methyl) pyridine, charges unknown
AMN082	0.0 M<br>HL^+ 9.94<br>H_2L^{2+} 16.80	(0.5 M)<br>CuL^{2+} 10.60<br>CuL_2^{2+} 19.75	(0.3 M)<br>CuL_2^+ 11.4		FeL^{2+} 4.34<br>FeL_2^{2+} 7.65<br>FeL_3^{2+} 9.70	MnL^{2+} 2.77<br>MnL_2^{2+} 4.87<br>MnL_3^{2+} 5.79	0.0 M<br>ZnL^{2+} 5.77<br>ZnL_2^{2+} 10.83	tentative, from 1,2-diamino-ethane (1.0 M)
Amodiaquine	HL 10.85<br>H_2L 19.05	CuHL 13.54						tentative, from 2-dimethyl-amino-methyl-phenol (20 °C, 40% mix solv.), charges unknown
Antagonist of the A(2A) adenosine receptor - derivative 49	HL 9.59<br>H_2L 13.51	CuL 7.3<br>CuL_2 12.90	(20 °C, 1 M)<br>CuL_2 10.90					tentative, from 2-(2'-amino-ethyl) pyridine, charges unknown
							(20 °C)<br>ZnHL 14.96<br>$ZnHL_2$ 20.22	tentative, from 1,3-diamino-propane
Apigenin	HL^{2-} 13.14<br>H_2L^- 21.68<br>H_3L 31.61	CuH_2L^+ 29.86<br>CuH_4L_2 58.38						50% mix solv.
	HL 11.34			FeL^{2+} 14.12				tentative, from 5-hydroxy-flavone (20 °C, 50% mix solv.)
	H_3L 10.18<br>H_4L 18.55<br>H_5L 23.52						ZnH_3L 14.46	tentative, from morin (50% mix solv.), charges unknown
Apomorphine	HL 12.8<br>H_2L 22.17	CuL 13.76<br>CuL_2 24.51		(22 °C)<br>FeL 20.05<br>FeL_2 34.71	(1.0 M)<br>FeHL 16.57<br>FeL 7.948	MnL 7.52<br>MnL_2 13.22	ZnL 9.50<br>ZnL_2 17.20	tentative, from catechol, charges unknown

				FeL_3 43.75	FeL_2 13.488			
L-Arginine	HL^+ 9.104<br>$H_2L_2^+$ 11.099	CuL^{2+} 7.652<br>CuL_2^{2+} 14.128<br>$CuL_2H_{-1}^-$ 3.14		(20 °C, 1 M)<br>FeL^{3+} 8.7	20 °C, 0.01 M<br>FeL^{2+} 3.20	MnL^{2+} 2.55	ZnL^{2+} 4.11<br>ZnL_2^{2+} 8.07	
Aromadendrin	HL 11.34	CuL 14.12		FeL 14.12				tentative, from 5-hydroxy-flavone (20 °C, 50% mix solv.), charges unknown
Ascorbic acid	HL 4.045	$CuHL^{2+}$ 3.94<br>CuL^+ 2.32<br>$Cu_2L_2^{2+}$ 6.33<br>$Cu_2H_{-2}L_2$ 0.05		(0.05 M)<br>FeL_2^+ 6.36	(20 °C, 1 M)<br>FeL^+ –0.27<br>FeL_2 1.54		(20 °C, 1 M)<br>ZnL^+ 4.34<br>ZnL_2 7.50	
ASI-1	HL 10.69<br>H_2L 19.99<br>H_3L 28.53			FeH_2L 29.13<br>FeL 22.06				tentative, from curcumin (50% mix solv.),charges unknown
	HL 8.83	CuL^+ 8.0<br>CuL_2 14.8			FeL^+ 5.2	MnL^+ 3.91<br>MnL_2 6.82	ZnL^+ 4.70<br>ZnL_2 8.3	tentative, from acetyl-acetone
ASI-5	HL 5.85	CuL 2.77				MnL 2.12	ZnL 2.07	tentative, from phenyl-phosphoric acid, charges unknown
	HL 10.17<br>H_2L 17.08<br>H_3L 19.79			FeL 19.9<br>FeL_2 26.6	FeHL 16.77<br>FeL 12.6<br>FeH_2L_2 32.24<br>FeL_2 18.8<br>Fe_2HL 19.27<br>Fe_2L 15.4			tentative, from methane-diphosphonic acid, charges unknown
Astilbin	HL 12.8<br>H_2L 22.17	CuL 13.76<br>CuL_2 24.51		(22 °C)<br>FeL 20.05<br>FeL_2 34.71<br>FeL_3 43.75	FeHL 16.57<br>FeL 7.948<br>FeL_2 13.488	MnL 7.52<br>MnL_2 13.22	ZnL 9.50<br>ZnL_2 17.20	tentative, from catechol (1 M), charges unknown
Azilsartan	HL 5.34	CuL 3.45<br>CuL_2 6.30					(35 °C, 50 % mix solv.)<br>ZnL 3.3<br>ZnL_2 5.8	tentative, from N-Methyl-anthranilic acid (22 °C), charges unknown
	HL 10.098<br>H_2L 13.660				(20 °C, 0.01 M)<br>FeL_2 4.0	(20 °C)<br>MnL 2.13		tentative, from β-alanine, charges unknown
Baicalein	HL^- 10.85<br>H_2L 19.61	CuL 12.54<br>CuL_2^{2-} 23.01			FeL 12.88<br>FeL_2^{2-} 23.66	MnL 6.41<br>MnL_2^{2-} 10.82	ZnL 8.41<br>ZnL_2^{2-} 14.87	tentative, from pyrogallol (30 °C)
	HL 11.34			FeL^{2+} 14.12				tentative, from 5-hydroxy-flavone (20 °C, 50% mix solv.)
Benserazide	HL^- 10.85<br>H_2L 19.61	CuL 12.54<br>CuL_2 23.01			FeL 12.88<br>FeL_2 23.66	MnL 6.41<br>MnL_2 10.82	ZnL 8.41<br>ZnL_2 14.87	tentative, from pyrogallol (30 °C), charges unknown
7H-Benzo[e] perimidin-7-	HL 8.75	(30 °C, 75% mix solv.)					ZnL 4.21	tentative, from 5-hydroxy-1,4-

one derivatives (R_6 = OH)		CuL 11.24<br>CuL_2 20.91					ZnL_2 12.07	naphtho-quinone (50% mix solv.), charges unknown
4H-1-Benzopyran-4-one	HL 11.34	CuL^{2+} 14.12		FeL^{2+} 14.12				tentative, from 5-hydroxy-flavone (20 °C, 50% mix solv.)
	H_3L 10.18<br>H_4L 18.55<br>H_5L 23.52						ZnH_3L 14.46	tentative, from morin (50% mix solv.), charges unknown
8-Benzyl-tetrahydropyrazino[2,1-f]purinedione (derivative n. 57)	HL 3.80	CuL 2.58					ZnL 1.10	tentative, from 2-(2'-Pyridyl)-thiophene, charges unknown
	HL 10.69<br>H_2L 18.87		(20 °C, 1.0 M)<br>CuL 10.80<br>CuL_2 13.50					tentative, from 2-amino-ethanethiol, charges unknown
Bikaverin	HL^- 11.35<br>H_2L 16.91						ZnL 8.23<br>$ZnH_{-2}L^{2-}$ –5.95	tentative, from 5,8-dihydroxy-1,4-naphtho-quinone (25 °C, 50% mix solv.)
	HL 8.75	(30 °C, 75% mix solv.)<br>CuL 11.24<br>CuL_2 20.91						tentative, from 5-hydroxy-1,4-naphtho-quinone (50% mix solv.), charges unknown
(-)-N6-(2-(4-(Biphenyl-4-yl)piperazin-1-yl)-ethyl)-N6-propyl-4,5,6,7-tetrahydrobenzo[d]thiazole-2,6-diamine derivatives	(0.0 M)<br>HL^+ 9.94<br>H_2L^{2+} 16.80	(0.5 M)<br>CuL^{2+} 10.60<br>CuL_2^{2+} 19.75	(0.3 M)<br>CuL_2^+ 11.4		FeL^{2+} 4.34<br>FeL_2^{2+} 7.65<br>FeL_3^{2+} 9.70	MnL^{2+} 2.77<br>MnL_2^{2+} 4.87<br>MnL_3^{2+} 5.79	(0.0 M)<br>ZnL^{2+} 5.77<br>ZnL_2^{2+} 10.83	tentative, from 1,2-diamino-ethane (1.0 M)
2,2′-Bipyridyl	HL^+ 4.23<br>H_2L^{2+} 5.55	CuL^{2+} 9.00<br>CuL_2^{2+} 14.724	(23 °C, 0.0 M)<br>CuL^+ 4.59<br>CuL_2^+ 9.18	FeL^{3+} 9.13<br>$FeH_{-1}L^{2+}$ 6.96<br>$FeH_{-2}L^+$ 5.11<br>FeL_2^{3+} 18.11<br>$FeH_{-1}L_2^{2+}$ 14.74<br>$FeH_{-2}L_2^+$ 10.76	FeL^{2+} 3.98<br>FeL_2^{2+} 9.09<br>FeL_3^{2+} 12.63	MnL^{2+} 2.62<br>MnL_2^{2+} 4.62<br>MnL_3^{2+} 5.72	ZnL^{2+} 5.13<br>ZnL_2^{2+} 9.50	
4-((5-Bromo-3-chloro-2-hydroxybenzyl) amino)-2-hydroxybenzoic acid (LX007, ZL006)	HL 13.74<br>H_2L 17.82<br>H_3L 18.10	(37 °C, 0.15 M)<br>CuHL 15.57<br>CuL 10.63		FeL 16.97<br>FeL_2 29.01<br>FeL_3 36.05			(37 °C, 0.15 M)<br>$ZnH_{-1}L$ –0.95	tentative, from 5-amino-salicylic acid (3.0 M), charges unknown
	HL 13.44<br>H_2L 16.22				(20 °C)<br>FeL 6.55<br>FeL_2 11.25	MnL 6.10		tentative, from salicylic acid, charges unknown
C-3 (α carboxyfullerene)	HL 5.29<br>H_2L 8.05	CuL 5.81<br>CuL_2 7.73		FeL 8.04<br>FeL_2 13.54	(1.0 M)<br>FeL 2.17<br>FeL_2 3.21	(0.16 M)<br>MnL 2.30	ZnL 2.7	tentative, from malonic acid, charges unknown

Caffeic acid amide analogues	HL 8.72<br>H_2L 13.13	CuHL 10.46<br>CuL 6.02<br>$CuH_{-1}L$ 0.25<br>$Cu_2H_{-1}L$ 3.54<br>$Cu_2H_{-3}L_3$ 0.97<br>$Cu_3H_{-2}L_2$ 7.41			FeL 3.86<br>$FeH_{-1}L$ –3.83<br>$FeH_{-1}L_2$ –0.36<br>$FeH_{-2}L_3$ –6.14<br>Fe_2L 6.69	$MnH_{-1}L$ –4.88<br>$MnH_{-2}L$ –15.55	ZnL 2.99<br>$ZnH_{-1}L$ –3.03<br>$ZnH_{-1}L_2$ –0.39<br>$ZnH_{-2}L_2$ –8.21<br>$ZnH_{-2}L_3$ –5.51	tentative, from caffeic acid, charges unknown
	HL 12.8<br>H_2L 22.17			(22 °C)<br>FeL 20.05<br>FeL_2 34.71<br>FeL_3 43.75				tentative, from catechol (1 M), charges unknown
Carbazole-derived compounds	(0.0 M)<br>HL^+ 9.94<br>H_2L^{2+} 16.80	(0.5 M)<br>CuL^{2+} 10.60<br>CuL_2^{2+} 19.75	(0.3 M)<br>CuL_2^+ 11.4		FeL^{2+} 4.34<br>FeL_2^{2+} 7.65<br>FeL_3^{2+} 9.70	MnL^{2+} 2.77<br>MnL_2^{2+} 4.87<br>MnL_3^{2+} 5.79	(0.0 M)<br>ZnL^{2+} 5.77<br>ZnL_2^{2+} 10.83	tentative, from 1,2-diamino-ethane (1.0 M)
Carbidopa	HL 9.77<br>H_2L 18.61<br>H_3L 20.78	CuHL 17.25<br>CuH_2L_2 33.70<br>$CuH L_2$ 26.9<br>$CuH_{-1}L$ 9.35<br>$CuH_{-2}L$ 3.20<br>Cu_2L_2 25.7<br>$Cu_2H_{-1}L_2$ 20.83<br>$Cu_2H_{-2}L_2$ 15.22		(0.12 M)<br>FeH_2L 31.92<br>FeL 18.39	FeL 8.80	(0.2 M)<br>MnHL 17.76<br>MnL 8.14<br>MnH_2L_2 33.43<br>$MnHL_2$ 23.75<br>MnL_2 12.43	ZnHL 13.77<br>ZnL_2 11.07<br>Zn_2L_2 16.55	tentative, from L-dopa, charges unknown
Carnosic acid	HL 12.8<br>H_2L 22.17	CuL 13.76<br>CuL_2 24.51		(22 °C)<br>FeL 20.05<br>FeL_2 34.71<br>FeL_3 43.75	(1.0 M)<br>FeHL 16.57<br>FeL 7.948<br>FeL_2 13.488	MnL 7.52<br>MnL_2 13.22	ZnL 9.50<br>ZnL_2 17.20	tentative, from catechol, charges unknown
Catechin	HL^{3-} 13.26<br>H_2L^{2-} 24.52<br>H_3L^- 33.93<br>H_4L 42.57			FeHL 21.8<br>$FeH_2L_2^{3-}$ 37.5<br>$FeH_3L_3^{6-}$ 47.4				unspecified ionic strength
	HL^{3-} 11.50<br>H_2L^{2-} 22.36<br>H_3L^- 31.56<br>H_4L 39.72	CuH_2L 31.81<br>$CuHL^-$ 23.84<br>CuL^{2-} 14.45						data from [Teixeira 2005]
	HL 12.8<br>H_2L 22.17	CuL 13.76<br>CuL_2 24.51			(1.0 M)<br>FeHL 16.57<br>FeL 7.948<br>FeL_2 13.488	MnL 7.52<br>MnL_2 13.22	ZnL 9.50<br>ZnL_2 17.20	tentative, from catechol, charges unknown
Ceftriaxone	HL^- 4.35<br>H_2L 7.54<br>H_3L^+ 9.88	CuL 2.5					ZnL 4.45	data from [Mayakova 2016]
	HL 9.60		(0.3 M)	FeL 10.83	FeL 4.13	MnL 2.85		tentative, from glycine, charges

	H_2L 11.93		CuL_2 10.0	FeL_2 20.48	FeL_2 7.65			unknown
Celastrol	HL 12.5<br>H_2L 21.18	(30 °C, 0.2 M)<br>CuL 11.78<br>CuL_2 20.86		(unspecified temperature)<br>FeL 20.85<br>FeL_2 36.15<br>FeL_3 45.95				tentative, from 2,3-dihydroxy-naphtalene (20 °C), charges unknown
	HL 12.8<br>H_2L 22.17				(1.0 M)<br>FeHL 16.57<br>FeL 7.948<br>FeL_2 13.488	MnL 7.52<br>MnL_2 13.22	ZnL 9.50<br>ZnL_2 17.20	tentative, from catechol, charges unknown
CEP-1347	HL 10.098<br>H_2L 13.660				(20 °C, 0.01 M)<br>FeL_2 4.0	(20 °C)<br>MnL 2.13		tentative, from β-alanine, charges unknown
Chebulagic acid	HL 12<br>H_2L 23.2<br>H_3L 31.8<br>H_4L 36.2	(40% mix solv.)<br>CuHL 21.90<br>CuH_2L_2 43.04		FeH_2L 33.7<br>FeHL 30.05<br>FeL 22.28<br>FeH_4L_2 64.7<br>FeH_2L_2 50.01<br>FeL_2 33.89<br>FeH_6L_3 94.4<br>FeH_3L_3 69.16<br>FeL_3 42.15			Zn_2L 11.4	tentative, from gallic acid, charges unknown
	HL 10.85<br>H_2L 19.61				FeL 12.88<br>FeL_2 23.66	MnL 6.41<br>MnL_2 10.82		tentative, from pyrogallol (30 °C), charges unknown
Chlorogenic acid	HL^{2-} 12.06<br>H_2L^- 20.36<br>H_3L 23.71	CuHL 16.92<br>CuL^- 12.74<br>CuL_2^{4-} 23.35		FeL 17.64<br>$FeHL^+$ 22.20		MnL^- 7.02<br>MnL_2^{4-} 12.13	ZnL^- 8.79<br>ZnL_2^{4-} 16.20	(20 °C, 1.0 M)
	HL 12.20<br>H_2L 21.45				FeHL 16.57<br>FeL 7.948<br>FeL_2 13.488			tentative, from catechol (1.0 M), charges unknown
3'-O-(3-Chloropivaloyl) quercetin	HL 11.34	CuL 14.12		FeL 14.12				tentative, from 5-hydroxy-flavone (20 °C, 50% mix solv.), charges unknown
	H_3L 10.18<br>H_4L 18.55<br>H_5L 23.52						ZnH_3L 14.46	tentative, from morin (50% mix solv.), charges unknown
Chlorpromazine	HL^+ 10.77<br>H_2L^{2+} 19.62	CuL^{2+} 9.77<br>CuL_2^{2+} 16.94					(20 °C)<br>$ZnHL^{3+}$ 14.96<br>$ZnHL_2^{3+}$ 20.22	tentative, from 1,3-diamino-propane
Chrysin	(50% mix solv.)<br>HL^- 12.37<br>H_2L 20.74	(50% mix solv.)<br>$CuHL^+$ 23.01		FeL^+ 11.40				data for Fe(III) (44.6% mix solv.) from [Engelmann 2005]
	H_3L 10.18						ZnH_3L 14.46	tentative, from morin (50% mix

	H_4L 18.55<br>H_5L 23.52							solv.), charges unknown
Clioquinol	HL 9.07<br>H_2L^+ 10.57				FeL^+ 7.61<br>FeL_2 15.41<br>FeL_3 20.23			(50% mix solv., 0.3 M)
	HL 9.684<br>H_2L^+ 14.70	CuL^+ 12.10<br>CuL_2 23.00	(50% mix solv., 0.3 M)<br>CuL_2^- 14.7	FeL^{2+} 13.69<br>FeL_2^+ 26.3<br>FeL_3 36.9		MnL^+ 7.85<br>MnL_2 14.40	ZnL^+ 8.52<br>ZnL_2 15.84	tentative, from 8-hydroxy-quinoline
Clioquinol-selegiline hybrid	HL 10.25<br>H_2L 18.11<br>H_3L 22.52<br>H_4L 25.47			FeH_4L 52.11<br>FeH_2L 27.04<br>FeH_2L_2 45.3<br>$FeHL_2$ 39.25<br>FeL_2 34.0	FeH_4L_2 43.2<br>FeH_2L_2 32.97			tentative, from pyridoxal isonicotinoyl hydrazone, charges unknown
Clovamide analogues (R_1 and R_2 = OH, and/or R_3 and R_4 = OH)	HL 12.8<br>H_2L 22.17	CuL 13.76<br>CuL_2 24.51		(22 °C)<br>FeL 20.05<br>FeL_2 34.71<br>FeL_3 43.75	(1.0 M)<br>FeHL 16.57<br>FeL 7.948<br>FeL_2 13.488	MnL 7.52<br>MnL_2 13.22	ZnL 9.50<br>ZnL_2 17.20	tentative, from catechol, charges unknown
"Compound 1"	(0.0 M)<br>HL^+ 9.94<br>H_2L^{2+} 16.80	(0.5 M)<br>CuL^{2+} 10.60<br>CuL_2^{2+} 19.75	(0.3 M)<br>CuL_2^+ 11.4		FeL^{2+} 4.34<br>FeL_2^{2+} 7.65<br>FeL_3^{2+} 9.70	MnL^{2+} 2.77<br>MnL_2^{2+} 4.87<br>MnL_3^{2+} 5.79	(0.0 M)<br>ZnL^{2+} 5.77<br>ZnL_2^{2+} 10.83	tentative, from 1,2-diamino-ethane (1.0 M)
"Compound (-) -8a"	HL 12.8<br>H_2L 22.17	CuL 13.76<br>CuL_2 24.51		(22 °C)<br>FeL 20.05<br>FeL_2 34.71<br>FeL_3 43.75	(1.0 M)<br>FeHL 16.57<br>FeL 7.948<br>FeL_2 13.488	MnL 7.52<br>MnL_2 13.22	ZnL 9.50<br>ZnL_2 17.20	tentative, from catechol, charges unknown
"Compound 8"	HL 10.69<br>H_2L 18.87	(0.26 M)<br>CuL_2 16.74	(20 °C, 1.0 M)<br>CuL 10.80<br>CuL_2 13.50				ZnL 8.77<br>$ZnH_{-1}L$ 1.94<br>ZnL_2 15.72	tentative, from 2-amino-ethanethiol, charges unknown
"Compound 21", derivative of 3-methyl-1-(2,4,6-trihydroxyphenyl) butan-1-one	HL 9.60<br>H_2L^+ 11.93	CuL^+ 8.38<br>CuL_2 15.25	(0.3 M)<br>CuL_2^- 10.0	FeL^{2+} 10.83<br>FeL_2^+ 20.48	FeL^+ 4.13<br>FeL_2 7.65	MnL^+ 2.85	ZnL^+ 5.03<br>ZnL_2 9.30	tentative, from glycine
"Compound (-) – 21 a", derivative of N-6-(2-(4-(1H-indol-5-yl)piperazin-1-yl)ethyl)-N-6-propyl-4,5,6,7-tetrahydrobenzo[d]thiazole-2,6-diamine	(0.0 M)<br>HL^+ 9.94<br>H_2L^{2+} 16.80	(0.5 M)<br>CuL^{2+} 10.60<br>CuL_2^{2+} 19.75	(0.3 M)<br>CuL_2^+ 11.4		FeL^{2+} 4.34<br>FeL_2^{2+} 7.65<br>FeL_3^{2+} 9.70	MnL^{2+} 2.77<br>MnL_2^{2+} 4.87<br>MnL_3^{2+} 5.79	(0.0 M)<br>ZnL^{2+} 5.77<br>ZnL_2^{2+} 10.83	tentative, from 1,2-diamino-ethane (1.0 M)
Creatine	HL 11.04<br>H_2L 15.29	CuHL 14.41<br>CuL 7.89<br>$CuH_{-1}L$ 1.97						tentative, from phospho-creatine, charges unknown

	HL 9.60<br>H_2L^+ 11.93		(0.3 M)<br>CuL_2^- 10.0	FeL^{2+} 10.83<br>FeL_2^+ 20.48	FeL^+ 4.13<br>FeL_2 7.65	MnL^+ 2.85	ZnL^+ 5.03<br>ZnL_2 9.30	tentative, from glycine
Cudraflavone B	HL 11.34	CuL 14.12		FeL 14.12				tentative, from 5-hydroxy-flavone (20 °C, 50% mix solv.), charges unknown
	H_3L 10.18<br>H_4L 18.55<br>H_5L 23.52						ZnH_3L 14.46	tentative, from morin (50% mix solv.), charges unknown
Curcumin	HL^{2-} 10.51<br>H_2L^- 20.39<br>H_3L 28.77			FeL 22.25<br>$FeH_{-1}L^-$ 12.14	FeH_2L^+ 28.11<br>FeHL 19.76<br>FeL^- 9.20			unspecified ionic strength; data from [Bernabé-Pineda 2004]
	HL 8.83	CuL 8.0<br>CuL_2 14.8				MnL 3.91<br>MnL_2 6.82	ZnL 4.70<br>ZnL_2 8.3	tentative, from acetyl-acetone, charges unknown
Cyanidin	HL 12.8<br>H_2L 22.17	CuL 13.76<br>CuL_2 24.51		(22 °C)<br>FeL 20.05<br>FeL_2 34.71<br>FeL_3 43.75	(1.0 M)<br>FeHL 16.57<br>FeL 7.948<br>FeL_2 13.488	MnL 7.52<br>MnL_2 13.22	ZnL 9.50<br>ZnL_2 17.20	tentative, from catechol, charges unknown
D512	(0.0 M)<br>HL^+ 9.94<br>H_2L^{2+} 16.80	(0.5 M)<br>CuL^{2+} 10.60<br>CuL_2^{2+} 19.75	(0.3 M)<br>CuL_2^+ 11.4		FeL^{2+} 4.34<br>FeL_2^{2+} 7.65<br>FeL_3^{2+} 9.70	MnL^{2+} 2.77<br>MnL_2^{2+} 4.87<br>MnL_3^{2+} 5.79	(0.0 M)<br>ZnL^{2+} 5.77<br>ZnL_2^{2+} 10.83	tentative, from 1,2-diamino-ethane (1.0 M)
D607 (bipyridyl-D2R/D3R agonist hybrid)	HL 4.23<br>H_2L 5.55	CuL 9.00<br>CuL_2 14.724	(23 °C, 0.0 M)<br>CuL 4.59<br>CuL_2 9.18	FeL 9.13<br>$FeH_{-1}L$ 6.96<br>$FeH_{-2}L$ 5.11<br>FeL_2 18.11<br>$FeH_{-1}L_2$ 14.74<br>$FeH_{-2}L_2$ 10.76	FeL 3.98<br>FeL_2 9.09<br>FeL_3 12.63	MnL 2.62<br>MnL_2 4.62<br>MnL_3 5.72	ZnL 5.13<br>ZnL_2 9.50	tentative, from 2,2'-bipyridine, charges unknown
DA-2 (8D)	HL 9.684<br>H_2L 14.70	CuL 12.10<br>CuL_2 23.00	(50% mix solv., 0.3 M)<br>CuL_2 14.7	FeL 13.69<br>FeL_2 26.3<br>FeL_3 36.9	(20 °C, 0.01 M)<br>FeL 8.0<br>FeL_2 15.0	MnL 7.85<br>MnL_2 14.40	ZnL 8.52<br>ZnL_2 15.84	tentative, from 8-hydroxy-quinoline, charges unknown
DA-3	(0.0 M)<br>HL 9.94<br>H_2L 16.80	(0.5 M)<br>CuL 10.60<br>CuL_2 19.75	(0.3 M)<br>CuL_2 11.4		FeL 4.34<br>FeL_2 7.65<br>FeL_3 9.70	MnL 2.77<br>MnL_2 4.87<br>MnL_3 5.79	(0.0 M)<br>ZnL 5.77<br>ZnL_2 10.83	tentative, from 1,2-diamino-ethane (1.0 M), charges unknown
	HL 12.20<br>H_2L 21.45			(22 °C)<br>FeL 20.05<br>FeL_2 34.71<br>FeL_3 43.75				tentative, from catechol (1.0 M), charges unknown
DA-4	(0.0 M)<br>HL 9.94<br>H_2L 16.80	(0.5 M)<br>CuL 10.60<br>CuL_2 19.75	(0.3 M)<br>CuL_2 11.4		FeL 4.34<br>FeL_2 7.65<br>FeL_3 9.70	MnL 2.77<br>MnL_2 4.87<br>MnL_3 5.79	(0.0 M)<br>ZnL 5.77<br>ZnL_2 10.83	tentative, from 1,2-diamino-ethane (1.0 M), charges unknown
	HL 12.20<br>H_2L 21.45			(22 °C)<br>FeL 20.05				tentative, from catechol (1.0 M), charges unknown

				FeL_2 34.71<br>FeL_3 43.75				
Dabigatran etexilate	(0.0 M)<br>HL 9.94<br>H_2L 16.80	(0.5 M)<br>CuL 10.60<br>CuL_2 19.75	(0.3 M)<br>CuL_2 11.4		FeL 4.34<br>FeL_2 7.65<br>FeL_3 9.70	MnL 2.77<br>MnL_2 4.87<br>MnL_3 5.79	(0.0 M)<br>ZnL 5.77<br>ZnL_2 10.83	tentative, from 1,2-diamino-ethane (1.0 M), charges unknown
Dabrafenib	HL 3.80	CuL 2.58					ZnL 1.10	tentative, from 2-(2'-pyridyl)-thiophene, charges unknown
	HL 10.84<br>H_2L 19.96		(20 °C, 1 M)<br>CuL 11.92<br>CuL_2 15.12					tentative, from 3-amino-propane-1-thiol, charges unknown
(S)-3,4-DCPG	HL 4.967<br>H_2L 7.71	CuL 3.45				MnL 2.23	ZnL 2.18	tentative, from phthalic acid, charges unknown
Deferasirox (Exjade)	HL^{2-} 10.61<br>H_2L^- 19.41			$FeHL^+$ 24.3<br>FeL 22.0<br>$FeH_2L_2^-$ 43.4<br>$FeHL_2^{2-}$ 41.2<br>FeL_2^{3-} 36.90				
Deferricoprogen	HL^{2-} 9.84<br>H_2L^- 18.84<br>H_3L 26.84	CuH_2L^+ 26.89<br>CuHL 23.70<br>CuL^- 15.25		FeL 29.35			ZnH_2L^+ 23.60<br>ZnHL 19.23<br>ZnL^- 11.80	(0.2 M)
Delphinidin	HL 10.85<br>H_2L 19.61	CuL 12.54<br>CuL_2 23.01			FeL 12.88<br>FeL_2 23.66	MnL 6.41<br>MnL_2 10.82	ZnL 8.41<br>ZnL_2 14.87	tentative, from pyrogallol (30 °C), charges unknown
	HL 12.8<br>H_2L 22.17			FeL 20.05<br>FeL_2 34.71<br>FeL_3 43.75				tentative, from catechol (22 °C), charges unknown
Demethoxycurcumin	HL^{2-} 10.69<br>H_2L^- 19.99<br>H_3L 28.53			FeH_2L^{2+} 29.13<br>FeL 22.06				tentative, from curcumin (50% mix solv.)
	HL 8.83	CuL 8.0<br>CuL_2 14.8			FeL 5.2	MnL 3.91<br>MnL_2 6.82	ZnL 4.70<br>ZnL_2 8.3	tentative, from acetyl-acetone, charges unknown
Dendropanax morbifera active compound	HL 12.8<br>H_2L 22.17	CuL 13.76<br>CuL_2 24.51		(22 °C)<br>FeL 20.05<br>FeL_2 34.71<br>FeL_3 43.75	(1.0 M)<br>FeHL 16.57<br>FeL 7.948<br>FeL_2 13.488	MnL 7.52<br>MnL_2 13.22	ZnL 9.50<br>ZnL_2 17.20	tentative, from catechol, charges unknown
Desferrioxamine (deferoxamine, DFO)	HL^{2-} 10.89<br>H_2L^- 20.44<br>H_3L 29.42<br>H_4L^+ 37.74	CuH_3L^{2+} 37.08<br>CuH_2L^+ 33.38<br>CuHL 24.43<br>Cu_2HL^{2+} 32.14		$FeHL^+$ 41.39<br>FeL 30.99	FeH_3L^{2+} 33.22<br>FeH_2L^+ 27.64		ZnH_3L^{2+} 33.71<br>ZnH_2L^+ 28.32<br>ZnHL 20.44	
(S)-N-(3-(3,6-Dibromo-9H-carbazol-9-yl)-2-fluoropropyl)-6-	HL 10.77<br>H_2L 19.62	CuL 9.77<br>CuL_2 16.94					(20 °C)<br>ZnHL 14.96<br>$ZnHL_2$ 20.22	tentative, from 1,3-diamino-propane, charges unknown

methoxypyridin-2-amine								
4,5-O-Dicaffeoyl-1-O-(malic acid methyl ester)-quinic acid derivatives (R_1, R_2, R_3, R_4, or R_5 = caffeoyl)	HL 12.8<br>H_2L 22.17	CuL 13.76<br>CuL_2 24.51		(22 °C)<br>FeL 20.05<br>FeL_2 34.71<br>FeL_3 43.75	(1.0 M)<br>FeHL 16.57<br>FeL 7.948<br>FeL_2 13.488	MnL 7.52<br>MnL_2 13.22	ZnL 9.50<br>ZnL_2 17.20	tentative, from catechol, charges unknown
Dihydromyricetin	HL 10.85<br>H_2L 19.61	CuL 12.54<br>CuL_2 23.01			FeL 12.88<br>FeL_2 23.66	MnL 6.41<br>MnL_2 10.82	ZnL 8.41<br>ZnL_2 14.87	tentative, from pyrogallol (30 °C), charges unknown
	HL 12.8<br>H_2L 22.17			FeL 20.05<br>FeL_2 34.71<br>FeL_3 43.75				tentative, from catechol (22 °C), charges unknown
5-(3,4-Dihydroxybenzylidene) -2,2-dimethyl-1,3-dioxane-4,6-dione	HL^- 12.8<br>H_2L 22.17	CuL 13.76<br>CuL_2^{2-} 24.51		(22 °C)<br>FeL^+ 20.05<br>FeL_2^- 34.71<br>FeL_3^{3-} 43.75	(1.0 M)<br>$FeHL^+$ 16.57<br>FeL 7.948<br>FeL_2^{2-} 13.488	MnL 7.52<br>MnL_2^{2-} 13.22	ZnL 9.50<br>ZnL_2^{2-} 17.20	tentative, from catechol
7,8-Dihydroxycoumarin derivative DHC12	HL 12.8<br>H_2L 22.17	CuL 13.76<br>CuL_2 24.51		(22 °C)<br>FeL 20.05<br>FeL_2 34.71<br>FeL_3 43.75	(1.0 M)<br>FeHL 16.57<br>FeL 7.948<br>FeL_2 13.488	MnL 7.52<br>MnL_2 13.22	ZnL 9.50<br>ZnL_2 17.20	tentative, from catechol, charges unknown
3',4'-Dihydroxyflavone	HL^- 13.43<br>H_2L 21.82			FeL^+ 20.87				data for Fe(III) (44.6% mix solv.) from [Engelmann 2005]
	HL^- 12.8<br>H_2L 22.17	CuL 13.76<br>CuL_2^{2-} 24.51			(1.0 M)<br>$FeHL^+$ 16.57<br>FeL 7.948<br>FeL_2^{2-} 13.488	MnL 7.52<br>MnL_2^{2-} 13.22	ZnL 9.50<br>ZnL_2^{2-} 17.20	tentative, from catechol
7,8-Dihydroxyflavone	HL^- 12.8<br>H_2L 22.17	CuL 13.76<br>CuL_2^{2-} 24.51		(22 °C)<br>FeL^+ 20.05<br>FeL_2^- 34.71<br>FeL_3^{3-} 43.75	(1.0 M)<br>$FeHL^+$ 16.57<br>FeL 7.948<br>FeL_2^{2-} 13.488	MnL 7.52<br>MnL_2^{2-} 13.22	ZnL 9.50<br>ZnL_2^{2-} 17.20	tentative, from catechol
5,7-Dihydroxy-4'-methoxyflavone	HL 11.79	CuL 9.83						tentative, from 5-hydroxy-7-methoxy-flavone (50% mix solv.), charges unknown
	HL 7.74			(20 °C, 10% mix solv.)<br>FeL 12.74<br>FeL_2 23.25				tentative, from 5-hydroxy-chromone, charges unknown
(E)-3,4-Dihydroxystyryl aralkyl sulfones	HL^- 12.8<br>H_2L 22.17	CuL 13.76<br>CuL_2^{2-} 24.51		(22 °C)<br>FeL^+ 20.05<br>FeL_2^- 34.71<br>FeL_3^{3-} 43.75	(1.0 M)<br>$FeHL^+$ 16.57<br>FeL 7.948<br>FeL_2^{2-} 13.488	MnL 7.52<br>MnL_2^{2-} 13.22	ZnL 9.50<br>ZnL_2^{2-} 17.20	tentative, from catechol
(E)-3,4-Dihydroxystyryl aralkyl sulfoxides	HL^- 12.8<br>H_2L 22.17	CuL 13.76<br>CuL_2^{2-} 24.51		(22 °C)<br>FeL^+ 20.05	(1.0 M)<br>$FeHL^+$ 16.57	MnL 7.52<br>MnL_2^{2-} 13.22	ZnL 9.50<br>ZnL_2^{2-} 17.20	tentative, from catechol

				FeL_2^{-} 34.71<br>FeL_3^{3-} 43.75	FeL 7.948<br>FeL_2^{2-} 13.488			
5,3'-Dihydroxy-3,7,4'-trimethoxyflavone	HL 11.79	CuL 9.83						tentative, from 5-hydroxy-7-methoxy-flavone (50% mix solv.), charges unknown
	HL 7.74			(20 °C, 10% mix solv.)<br>FeL 12.74<br>FeL_2 23.25				tentative, from 5-hydroxy-chromone, charges unknown
2-[[(1,1-Dimethylethyl) oxidoimino]-methyl]-3,5,6-trimethylpyrazine	HL 10.01<br>H_2L 13.60	CuHL 13.94<br>CuL 10.91<br>CuH_2L_2 27.50<br>$CuHL_2$ 25.45<br>CuL_2 18.49<br>$Cu_3H_{-1}L_3$ 35.6<br>$Cu_3H_{-2}L_3$ 29.06	(20 °C, 0.5 M)<br>CuH_2L_2 31.07<br>CuL_2 14.4	(24 °C)<br>FeL 11.40<br>FeL_2 21.70<br>FeL_3 30.10	(24 °C)<br>FeL 9.40<br>FeL_2 17.40<br>FeL_3 22.50	(0.3 M)<br>MnL 5.20<br>MnL_2 9.10	ZnHL 11.912<br>ZnL 5.20<br>$ZnHL_2$ 17.52<br>ZnL_2 10.58<br>ZnL_3 12.94<br>Zn_2L_2 13.26<br>$Zn_2H_{-1}L_2$ 6.724<br>$Zn_2H_{-2}L_2$ –2.64	tentative, from pyridine-2-aldoxime, charges unknown
DKP	HL 9.81<br>H_2L 15.88	CuHL 12.88<br>CuL 9.56<br>$CuHL_2$ 21.82<br>CuL_2 16.06	CuL 8.87<br>CuH_2L_2 20.13	FeL_2 7.05	(0.058 M)<br>FeL 5.80<br>FeL_2 10.06	(35 °C)<br>MnHL 12.83	ZnHL 11.72<br>ZnL 5.21<br>ZnL_2 10.13	tentative, from histamine, charges unknown
L-DOPA (levodopa, CVT-301)	HL^{2-} 9.77<br>H_2L^{-} 18.61<br>H_3L 20.78	CuHL 17.25<br>$CuH_2L_2^{2-}$ 33.70<br>$CuH\ L_2^{3-}$ 26.9<br>$CuH_{-1}L^{2-}$ 9.35<br>$CuH_{-2}L^{3-}$ 3.20<br>$Cu_2L_2^{2-}$ 25.7<br>$Cu_2H_{-1}L_2^{3-}$ 20.83<br>$Cu_2H_{-2}L_2$ 15.22		(0.12 M)<br>FeH_2L^{2+} 31.92<br>FeL 18.39	FeL^{-} 8.80	(0.2 M)<br>MnHL 17.76<br>MnL^{-} 8.14<br>$MnH_2L_2^{2-}$ 33.43<br>$MnHL_2^{3-}$ 23.75<br>MnL_2^{4-} 12.43	ZnHL 13.77<br>ZnL_2^{4-} 11.07<br>$Zn_2L_2^{2-}$ 16.55	
DOPA-derived peptido-mimetics (deprotected)	HL 9.77<br>H_2L 18.61<br>H_3L 20.78	CuHL 17.25<br>CuH_2L_2 33.70<br>$CuH\ L_2$ 26.9<br>$CuH_{-1}L$ 9.35<br>$CuH_{-2}L$ 3.20<br>Cu_2L_2 25.7<br>$Cu_2H_{-1}L_2$ 20.83<br>$Cu_2H_{-2}L_2$ 15.22		(0.12 M)<br>FeH_2L 31.92<br>FeL 18.39	FeL 8.80	(0.2 M)<br>MnHL 17.76<br>MnL 8.14<br>MnH_2L_2 33.43<br>$MnHL_2$ 23.75<br>MnL_2 12.43	ZnHL 13.77<br>ZnL_2 11.07<br>Zn_2L_2 16.55	tentative, from L-dopa, charges unknown
DOPA-derived peptido-mimetics (protected)	HL 12.8<br>H_2L 22.17	CuL 13.76<br>CuL_2 24.51		(22 °C)<br>FeL 20.05<br>FeL_2 34.71<br>FeL_3 43.75	(1.0 M)<br>FeHL 16.57<br>FeL 7.948<br>FeL_2 13.488	MnL 7.52<br>MnL_2 13.22	ZnL 9.50<br>ZnL_2 17.20	tentative, from catechol, charges unknown
L-DOPA deuterated	HL^{2-} 9.77	CuHL 17.25		(0.12 M)	FeL^{-} 8.80	(0.2 M)	ZnHL 13.77	tentative, from L-dopa

	H_2L^- 18.61<br>H_3L 20.78	$CuH_2L_2^{2-}$ 33.70<br>$CuH\ L_2^{3-}$ 26.9<br>$CuH_{-1}L^{2-}$ 9.35<br>$CuH_{-2}L^{3-}$ 3.20<br>$Cu_2L_2^{2-}$ 25.7<br>$Cu_2H_{-1}L_2^{3-}$ 20.83<br>$Cu_2H_{-2}L_2$ 15.22		FeH_2L^{2+} 31.92<br>FeL 18.39		MnHL 17.76<br>MnL^- 8.14<br>$MnH_2L_2^{2-}$ 33.43<br>$MnHL_2^{3-}$ 23.75<br>MnL_2^{4-} 12.43	ZnL_2^{4-} 11.07<br>$Zn_2L_2^{2-}$ 16.55	
Doxycycline	HL 13.00<br>H_2L 18.35	CuL 13.45<br>CuL_2 23.71	(30 °C)<br>Cu_2L 22.68	FeL 20.6<br>FeL_2 33.50	(27 °C)<br>FeL 6.00<br>FeL_2 9.45		ZnL 10.03	tentative, from chromotropic acid, charges unknown
Droxidopa	HL^{2-} 9.77<br>H_2L^- 18.61<br>H_3L 20.78	CuHL 17.25<br>$CuH_2L_2^{2-}$ 33.70<br>$CuH\ L_2^{3-}$ 26.9<br>$CuH_{-1}L^{2-}$ 9.35<br>$CuH_{-2}L^{3-}$ 3.20<br>$Cu_2L_2^{2-}$ 25.7<br>$Cu_2H_{-1}L_2^{3-}$ 20.83<br>$Cu_2H_{-2}L_2$ 15.22		(0.12 M)<br>FeH_2L^{2+} 31.92<br>FeL 18.39	FeL^- 8.80	(0.2 M)<br>MnHL 17.76<br>MnL^- 8.14<br>$MnH_2L_2^{2-}$ 33.43<br>$MnHL_2^{3-}$ 23.75<br>MnL_2^{4-} 12.43	ZnHL 13.77<br>ZnL_2^{4-} 11.07<br>$Zn_2L_2^{2-}$ 16.55	tentative, from L-dopa
Echinacoside	HL 12.8<br>H_2L 22.17	CuL 13.76<br>CuL_2 24.51		(22 °C)<br>FeL 20.05<br>FeL_2 34.71<br>FeL_3 43.75	(1.0 M)<br>FeHL 16.57<br>FeL 7.948<br>FeL_2 13.488	MnL 7.52<br>MnL_2 13.22	ZnL 9.50<br>ZnL_2 17.20	tentative, from catechol, charges unknown
Ellagic acid	HL 12.8<br>H_2L 22.17	CuL 13.76<br>CuL_2 24.51		(22 °C)<br>FeL 20.05<br>FeL_2 34.71<br>FeL_3 43.75	(1.0 M)<br>FeHL 16.57<br>FeL 7.948<br>FeL_2 13.488	MnL 7.52<br>MnL_2 13.22	ZnL 9.50<br>ZnL_2 17.20	tentative, from catechol, charges unknown
Entacapone (comtan, ASI-6)	HL^- 9.0<br>H_2L 13.5			FeL^+ 11.9<br>FeL_2^- 22.9<br>FeL_3^{3-} 33.6				[Orama 1997]
	HL^- 11.40<br>H_2L 17.87	CuL 12.30<br>CuL_2^{2-} 22.33				MnL 7.22<br>MnL_2^{2-} 12.5	ZnL 8.64<br>ZnL_2^{2-} 15.80	tentative, from 3-nitro-catechol
	(30 °C)<br>HL^- 10.67<br>H_2L 17.19				(30 °C)<br>FeL 13.53<br>FeL_2^{2-} 23.52			tentative, from 4-nitro-catechol
Enzastaurin	(0.0 M)<br>HL 9.94<br>H_2L 16.80	(0.5 M)<br>CuL 10.60<br>CuL_2 19.75	(0.3 M)<br>CuL_2 11.4		FeL 4.34<br>FeL_2 7.65<br>FeL_3 9.70	MnL 2.77<br>MnL_2 4.87<br>MnL_3 5.79	(0.0 M)<br>ZnL 5.77<br>ZnL_2 10.83	tentative, from 1,2-diamino-ethane (1.0 M), charges unknown
Epicatechin	HL 12.8<br>H_2L 22.17	CuL 13.76<br>CuL_2 24.51		(22 °C)<br>FeL 20.05<br>FeL_2 34.71<br>FeL_3 43.75	(1.0 M)<br>FeHL 16.57<br>FeL 7.948<br>FeL_2 13.488	MnL 7.52<br>MnL_2 13.22	ZnL 9.50<br>ZnL_2 17.20	tentative, from catechol, charges unknown

Epigallocatechin-3-gallate	HL 12<br>H_2L 23.2<br>H_3L 31.8<br>H_4L 36.2	(40% mix solv.)<br>CuLH 21.90<br>CuL_2H_2 43.04		FeH_2L 33.7<br>FeHL 30.05<br>FeL 22.28<br>FeH_4L_2 64.7<br>FeH_2L_2 50.01<br>FeL_2 33.89<br>FeH_6L_3 94.4<br>FeH_3L_3 69.16<br>FeL_3 42.15			Zn_2L 11.4	tentative, from gallic acid, charges unknown
Etidronate (HEDPA)	HL^{3-} 11.2<br>H_2L^{2-} 18.22<br>H_3L^{-} 20.92	CuH_2L 20.4<br>$CuHL^{-}$ 17.4<br>CuL^{2-} 12.0		FeHL 27.4<br>FeL^{-} 24.2<br>$FeH^{-1}L$ 19.1	FeH_2L 21.07<br>$FeHL^{-}$ 17.77<br>FeL^{2-} 12.9	MnH_2L 14.54<br>$MnHL^{-}$ 11.36<br>MnL^{2-} 6.94	ZnH_2L 20.37<br>$ZnHL^{-}$ 16.57<br>ZnL^{2-} 10.3	
Exifone	HL 12<br>H_2L 23.2<br>H_3L 31.8<br>H_4L 36.2	(40% mix solv.)<br>CuHL 21.90<br>CuH_2L_2 43.04		FeH_2L 33.7<br>FeHL 30.05<br>FeL 22.28<br>FeH_4L_2 64.7<br>FeH_2L_2 50.01<br>FeL_2 33.89<br>FeH_6L_3 94.4<br>FeH_3L_3 69.16<br>FeL_3 42.15			Zn_2L 11.4	tentative, from gallic acid, charges unknown
F13714,<br>F15599	HL 8.93<br>H_2L 10.87	CuL 9.69<br>CuL_2 16.90	(50% mix solv.)<br>CuL_2 10.66		(0.15 M)<br>FeL 4.105<br>FeL_2 7.441<br>FeL_3 10.116	(20 °C)<br>MnL 2.66	ZnL 5.2	tentative, from 2-(amino-methyl) pyridine, charges unknown
Farrerol	HL 11.34	CuL 14.12		FeL 14.12				tentative, from 5-hydroxy-flavone (20 °C, 50% mix solv.), charges unknown
Fisetin (3,3',4',7-tetra-hydroxy-flavone)	HL 12.8<br>H_2L 22.17	CuL 13.76<br>CuL_2 24.51		(22 °C)<br>FeL 20.05<br>FeL_2 34.71<br>FeL_3 43.75	(1.0 M)<br>FeHL 16.57<br>FeL 7.948<br>FeL_2 13.488	MnL 7.52<br>MnL_2 13.22	ZnL 9.50<br>ZnL_2 17.20	tentative, from catechol, charges unknown
Fraxetin	HL^{-} 12.8<br>H_2L 22.17	CuL 13.76<br>CuL_2^{2-} 24.51		(22 °C)<br>FeL^{+} 20.05<br>FeL_2^{-} 34.71<br>FeL_3^{3-} 43.75	(1.0 M)<br>$FeHL^{+}$ 16.57<br>FeL 7.948<br>FeL_2^{2-} 13.488	MnL 7.52<br>MnL_2^{2-} 13.22	ZnL 9.50<br>ZnL_2^{2-} 17.20	tentative, from catechol
Galangin	HL 11.0<br>H_2L 20.77<br>H_3L 28.98<br>H_4L 35.93<br>H_5L 41.47	CuH_2L 28.09<br>CuHL 22.22<br>CuL 14.85<br>$CuH_{-1}L$ 5.53<br>Cu_2L 17.37		FeH_2L 43.54<br>FeHL 38.59<br>FeL 32.30<br>$FeH_{-1}L$ 25.54<br>$FeH_{-2}L$ 16.66				tentative from quercetin (20 °C), charges unknown

		$Cu_2H_{-1}L$ 11.62<br>$Cu_2H_{-2}L$ 4.09<br>$Cu_2H_{-3}L$ –5.09<br>$Cu_2H_{-4}L$ –15.31		$Fe_2H_{-1}L$ 35.40<br>$Fe_2H_{-2}L$ 29.90<br>$Fe_2H_{-3}L$ 23.82<br>$Fe_2H_{-4}L$ 17.09<br>$Fe_2H_{-5}L$ 8.85<br>$Fe_2H_{-6}L$ –0.50				
Gallic acid and derivatives	HL^{3-} 12<br>H_2L^{2-} 23.2<br>H_3L^{-} 31.8<br>H_4L 36.2	(40% mix solv.)<br>$CuHL^{-}$ 21.90<br>$CuH_2L_2^{2-}$ 43.04		FeH_2L^{+} 33.7<br>FeHL 30.05<br>FeL^{-} 22.28<br>$FeH_4L_2^{+}$ 64.7<br>$FeH_2L_2^{3+}$ 50.01<br>FeL_2^{5+} 33.89<br>$FeH_6L_3^{3+}$ 94.4<br>$FeH_3L_3^{6+}$ 69.16<br>FeL_3^{9+} 42.15			Zn_2L 11.4	
Gallocatechin	HL 10.85<br>H_2L 19.61	CuL 12.54<br>CuL_2 23.01			FeL 12.88<br>FeL_2 23.66	MnL 6.41<br>MnL_2 10.82	ZnL 8.41<br>ZnL_2 14.87	tentative, from pyrogallol (30 °C), charges unknown
	HL 12.8<br>H_2L 22.17			FeL 20.05<br>FeL_2 34.71<br>FeL_3 43.75				tentative, from catechol (22 °C), charges unknown
Garcinol	HL 12.8<br>H_2L 22.17	CuL 13.76<br>CuL_2 24.51		(22 °C)<br>FeL 20.05<br>FeL_2 34.71<br>FeL_3 43.75	(1.0 M)<br>FeHL 16.57<br>FeL 7.948<br>FeL_2 13.488	MnL 7.52<br>MnL_2 13.22	ZnL 9.50<br>ZnL_2 17.20	tentative, from catechol, charges unknown
Genistein	HL 11.34	CuL 14.12		FeL 14.12				tentative, from 5-hydroxy-flavone (20 °C, 50% mix solv.), charges unknown
Glutamine	HL 8.68<br>H_2L^{+} 10.864	CuL^{+} 7.475<br>CuL_2 13.59			(3.0 M)<br>FeL^{+} 4.43<br>FeL_2 7.26	MnL^{+} 2.94	ZnL^{+} 4.38<br>ZnL_2 8.38<br>ZnL_3^{-} 10.24	
Glutathione derivatives	HL^{2-} 9.63<br>H_2L^{-} 18.06<br>H_3L 21.58<br>H_4L^{+} 23.68	CuL^{-} 7.545	(0.5 M)<br>$CuHL^{-}$ 24.9<br>$CuH_2L_2^{3-}$ 38.8			MnL^{-} 6.69	ZnHL 14.74<br>ZnL^{-} 8.31<br>$ZnH_2L_2^{2-}$ 29.50<br>$ZnHL_2^{3-}$ 22.533<br>ZnL_2^{4-} 13.62<br>$ZnH_{-1}L_2^{5-}$ 3.817<br>$ZnH_{-2}L_2^{6-}$ –6.485	tentative for derivatives
Glutathione - hydroxy-quinoline compound	HL^{+} 4.56	$CuH_{-1}L^{+}$ 1.0		$FeH_{-1}L^{2+}$ 6.37<br>$FeH_{-2}L^{+}$ 2.24			$ZnH_{-1}L^{+}$ –0.52	(0.15 M). Data from [Cacciatore 2013]
	HL 9.684<br>H_2L^{+} 14.70		(50% mix solv., 0.3 M)<br>CuL_2^{-} 14.7		(20 °C, 0.01 M)<br>FeL^{+} 8.0	MnL^{+} 7.85<br>MnL_2 14.40		tentative, from 8-hydroxy-quinoline

					FeL_2 15.0			
Glutathione - L-DOPA compound	HL 9.3	$CuH_{-1}L$ 7.0<br>$CuH_{-2}L^-$ –2.4					$ZnH_{-1}L$ –0.72<br>$ZnH_{-2}L^-$ –8.49<br>$ZnH_{-2}L_2^{2-}$ –2.84	(0.15 M). Data from [Cacciatore 2018]
	HL 12.8<br>H_2L 22.17			(22 °C)<br>FeL 20.05<br>FeL_2 34.71<br>FeL_3 43.75	(1.0 M)<br>FeHL 16.57<br>FeL 7.948<br>FeL_2 13.488	MnL 7.52<br>MnL_2 13.22		tentative, from catechol, charges unknown
Gly-N-C-DOPA	HL^{2-} 9.77<br>H_2L^- 18.61<br>H_3L 20.78	CuHL 17.25<br>$CuH_2L_2^{2-}$ 33.70<br>$CuH L_2^{3-}$ 26.9<br>$CuH_{-1}L^{2-}$ 9.35<br>$CuH_{-2}L^{3-}$ 3.20<br>$Cu_2L_2^{2-}$ 25.7<br>$Cu_2H_{-1}L_2^{3-}$ 20.83<br>$Cu_2H_{-2}L_2$ 15.22		(0.12 M)<br>FeH_2L^{2+} 31.92<br>FeL 18.39	FeL^- 8.80	(0.2 M)<br>MnHL 17.76<br>MnL^- 8.14<br>$MnH_2L_2^{2-}$ 33.43<br>$MnHL_2^{3-}$ 23.75<br>MnL_2^{4-} 12.43	ZnHL 13.77<br>ZnL_2^{4-} 11.07<br>$Zn_2L_2^{2-}$ 16.55	tentative, from L-dopa
GSK2795039	HL 10.01<br>H_2L 13.73<br>H_3L 15.60	CuL 11.96<br>CuL_2 21.40					ZnL 9.65<br>ZnL_2 19.11	tentative (50% mixed solvent), from 2-Methyl-8-(toluene-4-sulfonamide)-6-quinolylethanoic acid, charges unknown
Guanabenz	(0.0 M)<br>HL 9.94<br>H_2L 16.80	(0.5 M)<br>CuL 10.60<br>CuL_2 19.75	(0.3 M)<br>CuL_2 11.4		FeL 4.34<br>FeL_2 7.65<br>FeL_3 9.70	MnL 2.77<br>MnL_2 4.87<br>MnL_3 5.79	(0.0 M)<br>ZnL 5.77<br>ZnL_2 10.83	tentative, from 1,2-diamino-ethane (1.0 M), charges unknown
Hesperidin	HL 11.34	CuL 14.12		FeL 14.12				tentative, from 5-hydroxy-flavone (20 °C, 50% mix solv.), charges unknown
Hinokitiol	HL 7.66	CuL^+ 6.71<br>CuL_2 10.71					ZnL^+ 5.15<br>ZnL_2 8.05	50% mixed solvent (data from [Pan 1955])
	HL 6.68			(2.0 M)<br>FeL^{2+} 10.50		MnL^+ 4.60		tentative, from tropolone
8-HQ-MC-5 (VK-28)	HL 9.684<br>H_2L 14.70	CuL 12.10<br>CuL_2 23.00	(50% mix solv., 0.3 M)<br>CuL_2 14.7	FeL 13.69<br>FeL_2 26.3<br>FeL_3 36.9	(20 °C, 0.01 M)<br>FeL 8.0<br>FeL_2 15.0	MnL 7.85<br>MnL_2 14.40	ZnL 8.52<br>ZnL_2 15.84	tentative, from 8-hydroxy-quinoline, charges unknown
4-Hydroxyisophthalic acid	HL 13.44<br>H_2L 16.22	CuL 10.83<br>CuL_2 18.88		FeL 16.45<br>FeL_2 29.12<br>FeL_3 40.89	(20 °C)<br>FeL 6.55<br>FeL_2 11.25	MnL 6.10	(35 °C)<br>ZnL 7.10	tentative, from salicylic acid, charges unknown
1-Hydroxy-2-pyridinone derivatives	HL 5.78	CuL 6.84<br>CuL_2 12.46		(0.1 M)<br>FeL 10.61<br>FeL_2 20.11<br>FeL_3 27.21			ZnL 4.97<br>ZnL_2 9.09<br>ZnL_3 12.00	tentative, from 1-hydroxypyridin-2-one (0.2 M), charges unknown
3-Hydroxy-	HL 9.77	CuL^+ 10.42		FeL^{2+} 15.10			ZnL^+ 7.19	

4(1H)pyridinone (deferiprone, ferriprox)	H_2L^+ 13.45	$CuHL_2^+$ 21.98<br>CuL_2 19.09<br>$CuH_{-1}L_2^+$ 8.49		FeL_2^+ 26.61<br>FeL_3 35.88			ZnL_2 13.53	
3-Hydroxy-4(1H)pyridinone derivatives (R = H)	HL 9.77<br>H_2L 13.45	CuL 10.42<br>$CuHL_2$ 21.98<br>CuL_2 19.09<br>$CuH_{-1}L_2$ 8.49		FeL 15.10<br>FeL_2 26.61<br>FeL_3 35.88			ZnL 7.19<br>ZnL_2 13.53	tentative, from deferiprone, charges unknown
8-Hydroxyquinoline	HL 9.684<br>H_2L^+ 14.70	CuL^+ 12.10<br>CuL_2 23.00	(50% mix solv., 0.3 M)<br>CuL_2^- 14.7	FeL^{2+} 13.69<br>FeL_2^+ 26.3<br>FeL_3 36.9	(20 °C, 0.01 M)<br>FeL^+ 8.0<br>FeL_2 15.0	MnL^+ 7.85<br>MnL_2 14.40	ZnL^+ 8.52<br>ZnL_2 15.84	
8-Hydroxyquinoline-2-carboxaldehyde isonicotinoyl hydrazone	HL 9.684<br>H_2L 14.70	CuL 12.10<br>CuL_2 23.00	(50% mix solv., 0.3 M)<br>CuL_2 14.7	FeL 13.69<br>FeL_2 26.3<br>FeL_3 36.9	(20 °C, 0.01 M)<br>FeL 8.0<br>FeL_2 15.0	MnL 7.85<br>MnL_2 14.40	ZnL 8.52<br>ZnL_2 15.84	tentative, from 8-hydroxy-quinoline, charges unknown
Hydroxyquinoline-propargyl hybrid (HLA 20)	HL 9.684<br>H_2L 14.70	CuL 12.10<br>CuL_2 23.00	(50% mix solv., 0.3 M)<br>CuL_2 14.7	FeL 13.69<br>FeL_2 26.3<br>FeL_3 36.9	(20 °C, 0.01 M)<br>FeL 8.0<br>FeL_2 15.0	MnL 7.85<br>MnL_2 14.40	ZnL 8.52<br>ZnL_2 15.84	tentative, from 8-hydroxy-quinoline, charges unknown
Hydroxytyrosol butyrate	HL^- 12.8<br>H_2L 22.17	CuL 13.76<br>CuL_2^{2-} 24.51		(22 °C)<br>FeL^+ 20.05<br>FeL_2^- 34.71<br>FeL_3^{3-} 43.75	(1.0 M)<br>$FeHL^+$ 16.57<br>FeL 7.948<br>FeL_2^{2-} 13.488	MnL 7.52<br>MnL_2^{2-} 13.22	ZnL 9.50<br>ZnL_2^{2-} 17.20	tentative, from catechol
Hyperoside	HL 11.0<br>H_2L 20.77<br>H_3L 28.98<br>H_4L 35.93<br>H_5L 41.47	CuH_2L 28.09<br>CuHL 22.22<br>CuL 14.85<br>$CuH_{-1}L$ 5.53<br>Cu_2L 17.37<br>$Cu_2H_{-1}L$ 11.62<br>$Cu_2H_{-2}L$ 4.09<br>$Cu_2H_{-3}L$ –5.09<br>$Cu_2H_{-4}L$ –15.31		FeH_2L 43.54<br>FeHL 38.59<br>FeL 32.30<br>$FeH_{-1}L$ 25.54<br>$FeH_{-2}L$ 16.66<br>$Fe_2H_{-1}L$ 35.40<br>$Fe_2H_{-2}L$ 29.90<br>$Fe_2H_{-3}L$ 23.82<br>$Fe_2H_{-4}L$ 17.09<br>$Fe_2H_{-5}L$ 8.85<br>$Fe_2H_{-6}L$ –0.50				tentative, from quercetin (20 °C), charges unknown
IC87201	HL 10.85<br>H_2L 19.05	CuHL 13.54						tentative, from 2-dimethyl-amino-methyl-phenol (20 °C, 40% mix solv.), charges unknown
Icariin	HL 11.79	CuL 9.83						tentative, from 5-hydroxy-7-methoxy-flavone (50% mix solv.), charges unknown
	HL 7.74			(20 °C, 10% mix solv.)<br>FeL 12.74<br>FeL_2 23.25				tentative, from 5-hydroxy-chromone, charges unknown

Icariside II	HL 11.79	CuL 9.83						tentative, from 5-hydroxy-7-methoxy-flavone (50% mix solv.), charges unknown
	HL 7.74			(20 °C, 10% mix solv.)<br>FeL 12.74<br>FeL_2 23.25				tentative, from 5-hydroxy-chromone, charges unknown
l-(7-Imino-3-propyl-2,3-dihydrothiazolo [4, 5-d]pyrimidin-6(7H)-yl)urea	HL 10.69<br>H_2L 18.87	(0.26 M)<br>CuL_2 16.74	(20 °C, 1.0 M)<br>CuL 10.80<br>CuL_2 13.50				ZnL 8.77<br>$ZnH_{-1}L$ 1.94<br>ZnL_2 15.72	tentative, from 2-amino-ethanethiol, charges unknown
Imipramine	HL^{+} 10.77<br>H_2L^{2+} 19.62	CuL^{2+} 9.77<br>CuL_2^{2+} 16.94					(20 °C)<br>$ZnHL^{3+}$ 14.96<br>$ZnHL_2^{3+}$ 20.22	tentative, from 1,3-diamino-propane
Isobavachalcone	HL 10.01	CuL^{+} 6.80<br>CuL_2 12.55		(27 °C, 75% mix solv.)<br>FeL^{2+} 11.56<br>FeL_2^{+} 20.76		(30 °C, 75% mix solv.)<br>MnL^{+} 7.42	(40 °C, 50% mix solv.)<br>ZnL^{+} 6.30	tentative, from 2-hydroxy-acetophenone
Isochlorogenic acid	HL^{2-} 12.06<br>H_2L^{-} 20.36<br>H_3L 23.71	CuHL 16.92<br>CuL^{-} 12.74<br>CuL_2^{4-} 23.35		FeL 17.64<br>$FeHL^{+}$ 22.20		MnL^{-} 7.02<br>MnL_2^{4-} 12.13	ZnL^{-} 8.79<br>ZnL_2^{4-} 16.20	tentative, from chlorogenic acid (20 °C, 1 M)
	HL 12.20<br>H_2L 21.45				FeHL 16.57<br>FeL 7.948<br>FeL_2 13.488			tentative, from catechol (1.0 M), charges unknown
Isoquercetin (isoquercitrin)	HL 11.0<br>H_2L 20.77<br>H_3L 28.98<br>H_4L 35.93<br>H_5L 41.47	CuH_2L 28.09<br>CuHL 22.22<br>CuL 14.85<br>$CuH_{-1}L$ 5.53<br>Cu_2L 17.37<br>$Cu_2H_{-1}L$ 11.62<br>$Cu_2H_{-2}L$ 4.09<br>$Cu_2H_{-3}L$ –5.09<br>$Cu_2H_{-4}L$ –15.31		FeH_2L 43.54<br>FeHL 38.59<br>FeL 32.30<br>$FeH_{-1}L$ 25.54<br>$FeH_{-2}L$ 16.66<br>$Fe_2H_{-1}L$ 35.40<br>$Fe_2H_{-2}L$ 29.90<br>$Fe_2H_{-3}L$ 23.82<br>$Fe_2H_{-4}L$ 17.09<br>$Fe_2H_{-5}L$ 8.85<br>$Fe_2H_{-6}L$ –0.50				tentative, from quercetin (20 °C), charges unknown
	H_4L 9.22<br>H_5L 17.07						ZnH_4L 14.02	tentative, from quercetin (50% mix solv.), charges unknown
Kaempferol	HL 11.0<br>H_2L 20.77<br>H_3L 28.98<br>H_4L 35.93<br>H_5L 41.47	CuH_2L 28.09<br>CuHL 22.22<br>CuL 14.85<br>$CuH_{-1}L$ 5.53<br>Cu_2L 17.37<br>$Cu_2H_{-1}L$ 11.62<br>$Cu_2H_{-2}L$ 4.09		FeH_2L 43.54<br>FeHL 38.59<br>FeL 32.30<br>$FeH_{-1}L$ 25.54<br>$FeH_{-2}L$ 16.66<br>$Fe_2H_{-1}L$ 35.40<br>$Fe_2H_{-2}L$ 29.90				Tentative, from quercetin (20 °C), charges unknown

		$Cu_2H_{-3}L$ –5.09<br>$Cu_2H_{-4}L$ –15.31		$Fe_2H_{-3}L$ 23.82<br>$Fe_2H_{-4}L$ 17.09<br>$Fe_2H_{-5}L$ 8.85<br>$Fe_2H_{-6}L$ –0.50				
Kaempferol, 3-O-a-L arabino-furanoside-7-O-a-L-rhamno-pyranoside	HL 11.34			FeL^{2+} 14.12				tentative, from 5-hydroxy-flavone (20 °C, 50% mix solv.)
	H_3L 10.18<br>H_4L 18.55<br>H_5L 23.52						ZnH_3L 14.46	tentative, from morin (50% mix solv.), charges unknown
KR33493	HL 9.60<br>H_2L 11.93	CuL 8.38<br>CuL_2 15.25	(0.3 M)<br>CuL_2 10.0	FeL 10.83<br>FeL_2 20.48	FeL 4.13<br>FeL_2 7.65	MnL 2.85	ZnL 5.03<br>ZnL_2 9.30	tentative, from glycine, charges unknown
Kukoamine	HL 12.8<br>H_2L 22.17	CuL 13.76<br>CuL_2 24.51		(22 °C)<br>FeL 20.05<br>FeL_2 34.71<br>FeL_3 43.75	(1.0 M)<br>FeHL 16.57<br>FeL 7.948<br>FeL_2 13.488	MnL 7.52<br>MnL_2 13.22	ZnL 9.50<br>ZnL_2 17.20	tentative, from catechol, charges unknown
Lestaurtinib	(0.0 M)<br>HL 9.94<br>H_2L 16.80	(0.5 M)<br>CuL 10.60<br>CuL_2 19.75	(0.3 M)<br>CuL_2 11.4		FeL 4.34<br>FeL_2 7.65<br>FeL_3 9.70	MnL 2.77<br>MnL_2 4.87<br>MnL_3 5.79	(0.0 M)<br>ZnL 5.77<br>ZnL_2 10.83	tentative, from 1,2-diamino-ethane (1.0 M), charges unknown
Lipoic acid	HL 6.37	CuL^+ 3.50				MnL^+ 2.06	ZnL^+ 2.47	
Luteolin	HL 12.8<br>H_2L 22.17	CuL 13.76<br>CuL_2 24.51		(22 °C)<br>FeL 20.05<br>FeL_2 34.71<br>FeL_3 43.75	(1.0 M)<br>FeHL 16.57<br>FeL 7.948<br>FeL_2 13.488	MnL 7.52<br>MnL_2 13.22	ZnL 9.50<br>ZnL_2 17.20	tentative, from catechol, charges unknown
LY354740	HL 10.31<br>H_2L 12.71						ZnL 4.76<br>ZnL_2 9.16	tentative, from 1-Amino-cyclopentane-carboxylic acid (20 °C), charges unknown
M10<br>M30 (VAR10303)<br>M99	HL 9.684<br>H_2L 14.70	CuL 12.10<br>CuL_2 23.00	(50% mix solv., 0.3 M)<br>CuL_2 14.7	FeL 13.69<br>FeL_2 26.3<br>FeL_3 36.9	(20 °C, 0.01 M)<br>FeL 8.0<br>FeL_2 15.0	MnL 7.85<br>MnL_2 14.40	ZnL 8.52<br>ZnL_2 15.84	tentative, from 8-hydroxy-quinoline, charges unknown
Macranthoin G	HL 12.8<br>H_2L 22.17	CuL 13.76<br>CuL_2 24.51		(22 °C)<br>FeL 20.05<br>FeL_2 34.71<br>FeL_3 43.75	(1.0 M)<br>FeHL 16.57<br>FeL 7.948<br>FeL_2 13.488	MnL 7.52<br>MnL_2 13.22	ZnL 9.50<br>ZnL_2 17.20	tentative, from catechol, charges unknown
Magnesium lithospermate B	HL 12.8<br>H_2L 22.17	CuL 13.76<br>CuL_2 24.51		(22 °C)<br>FeL 20.05<br>FeL_2 34.71<br>FeL_3 43.75	(1.0 M)<br>FeHL 16.57<br>FeL 7.948<br>FeL_2 13.488	MnL 7.52<br>MnL_2 13.22	ZnL 9.50<br>ZnL_2 17.20	tentative, from catechol, charges unknown
α-Mangostin	HL 12.8<br>H_2L 22.17	CuL 13.76<br>CuL_2 24.51		(22 °C)<br>FeL 20.05<br>FeL_2 34.71	(1.0 M)<br>FeHL 16.57<br>FeL 7.948	MnL 7.52<br>MnL_2 13.22	ZnL 9.50<br>ZnL_2 17.20	tentative, from catechol, charges unknown

				FeL_3 43.75	FeL_2 13.488			
γ-Mangostin	HL 12.8<br>H_2L 22.17	CuL 13.76<br>CuL_2 24.51		(22 °C)<br>FeL 20.05<br>FeL_2 34.71<br>FeL_3 43.75	(1.0 M)<br>FeHL 16.57<br>FeL 7.948<br>FeL_2 13.488	MnL 7.52<br>MnL_2 13.22	ZnL 9.50<br>ZnL_2 17.20	tentative, from catechol, charges unknown
MAOI-1	HL 10.85<br>H_2L 19.61	CuL 12.54<br>CuL_2 23.01			FeL 12.88<br>FeL_2 23.66	MnL 6.41<br>MnL_2 10.82	ZnL 8.41<br>ZnL_2 14.87	tentative, from pyrogallol (30 °C), charges unknown
MAOI-2	(0.0 M)<br>HL 9.94<br>H_2L 16.80	(0.5 M)<br>CuL 10.60<br>CuL_2 19.75	(0.3 M)<br>CuL_2 11.4		FeL 4.34<br>FeL_2 7.65<br>FeL_3 9.70	MnL 2.77<br>MnL_2 4.87<br>MnL_3 5.79	(0.0 M)<br>ZnL 5.77<br>ZnL_2 10.83	tentative, from 1,2-diamino-ethane (1.0 M), charges unknown
MAOI-4	HL 10.77<br>H_2L 19.62	CuL 9.77<br>CuL_2 16.94					(20 °C)<br>ZnHL 14.96<br>$ZnHL_2$ 20.22	tentative, from 1,3-diamino-propane, charges unknown
MAOI-8	HL 10.85<br>H_2L 19.05	CuHL 13.54						tentative, from 2-dimethyl-amino-methyl-phenol (20 °C, 40% mix solv.), charges unknown
Meclofenamic acid	HL 10.098<br>H_2L 13.660				(20 °C, 0.01 M)<br>FeL_2 4.0	(20 °C)<br>MnL 2.13		tentative, from β-alanine, charges unknown
Metformin (Met)	HL 11.10<br>H_2L^+ 14.00	CuL^+ 8.595<br>CuL_2 14.949		FeL^{2+} 11.872<br>FeL_2^+ 22.318			ZnL^+ 6.444<br>ZnL_2 11.637	20% mixed solvent (data from [Thakur 2012])
Methoxy-6-acetyl-7-methylijuglone	HL 8.75	(30 °C, 75% mix solv.)<br>CuL^+ 11.24<br>CuL_2 20.91					ZnL^+ 4.21<br>ZnL_2 12.07	tentative, from 5-hydroxy-1,4-naphtho-quinone (50% mix solv.)
N'-(4-Methylbenzylidene)-5-phenylisoxazole-3-carbohydrazide	HL 4.14<br>H_2L 6.24	CuL 4.92<br>CuL_2 8.97					ZnL 2.77	tentative, from carbohydrazide (20 °C), charges unknown
Mildronate	HL 10.098<br>H_2L 13.660				(20 °C, 0.01 M)<br>FeL_2 4.0	(20 °C)<br>MnL 2.13		tentative, from β-alanine, charges unknown
Minocycline	HL 8.968<br>H_2L 16.392<br>H_3L 21.268<br>H_4L 24.311	CuH_3L 25.391<br>CuH_2L 23.065<br>CuHL 18.316<br>CuL 12.523<br>Cu_2H_4L 41.730					ZnH_2L 19.165<br>ZnHL 14.267<br>ZnH_3L_2 33.803<br>ZnH_2L_2 28.225<br>Zn_2HL 16.811	37 °C, 0.15 M, charges unknown
Mitomycin C	HL 9.87<br>H_2L 14.61	CuL 8.49<br>CuL_2 15.52			(20 °C, 0.0 M)<br>FeL 3.66<br>FeL_2 6.34	(20 °C, 0.0 M)<br>MnL 3.60	(0.2 M)<br>ZnL 4.945<br>ZnL_2 8.86	tentative, from 2-aminophenol, charges unknown
MitoQ	HL 6.06<br>H_2L 9.11			FeL 7.26<br>FeL_2 13.30		MnL 3.91	ZnL 6.04<br>ZnL_2 10.22	tentative, from 2,5-dihydroxy-1,4-benzoquinone (30 °C, 25% mix solv.), charges unknown
Morin	(0.05 M)	CuH_3L 15.95					ZnH_3L 14.46	(50% mix solv.)

	H_3L^{2-} 10.18<br>H_4L^{-} 18.55<br>H_5L 23.52							
	HL 7.74			(20 °C, 10% mix solv.)<br>FeL 12.74<br>FeL_2 23.25				tentative, from 5-hydroxy-chromone, charges unknown
[18F]MPPF	(0.0 M)<br>HL 9.94<br>H_2L 16.80	(0.5 M)<br>CuL 10.60<br>CuL_2 19.75	(0.3 M)<br>CuL_2 11.4		FeL 4.34<br>FeL_2 7.65<br>FeL_3 9.70	MnL 2.77<br>MnL_2 4.87<br>MnL_3 5.79	(0.0 M)<br>ZnL 5.77<br>ZnL_2 10.83	tentative, from 1,2-diamino-ethane (1.0 M), charges unknown
MSX-3	HL 6.23	CuL 2.83				MnL 2.21	ZnL 2.13	tentative, from glycerol-1-phosphate, charges unknown
Myricetin<br>Myricitrin	HL 11.0<br>H_2L 20.77<br>H_3L 28.98<br>H_4L 35.93<br>H_5L 41.47	CuH_2L 28.09<br>CuHL 22.22<br>CuL 14.85<br>$CuH_{-1}L$ 5.53<br>Cu_2L 17.37<br>$Cu_2H_{-1}L$ 11.62<br>$Cu_2H_{-2}L$ 4.09<br>$Cu_2H_{-3}L$ –5.09<br>$Cu_2H_{-4}L$ –15.31		FeH_2L 43.54<br>FeHL 38.59<br>FeL 32.30<br>$FeH_{-1}L$ 25.54<br>$FeH_{-2}L$ 16.66<br>$Fe_2H_{-1}L$ 35.40<br>$Fe_2H_{-2}L$ 29.90<br>$Fe_2H_{-3}L$ 23.82<br>$Fe_2H_{-4}L$ 17.09<br>$Fe_2H_{-5}L$ 8.85<br>$Fe_2H_{-6}L$ –0.50				tentative, from quercetin (20 °C), charges unknown
Naringenin	HL^{2-} 11.12<br>H_2L^{-} 19.61<br>H_3L 27.08	CuH_2L^{+} 25.3<br>CuHL 18.31<br>CuL^{-} 6.25<br>CuH_4L_2 49.15<br>$CuH_3L_2^{-}$ 41.17<br>$CuH_2L_2^{2-}$ 32.21<br>$CuHL_2^{3-}$ 21.2<br>CuL_2^{4-} 10.13						data (27 °C, 70% mix solv.) from [Brodowska 2013]
	HL 11.34			FeL 14.12				tentative, from 5-hydroxy-flavone (20 °C, 50% mix solv.), charges unknown
Naringin	HL 11.12<br>H_2L 19.61<br>H_3L 27.08	CuH_2L 25.3<br>CuHL 18.31<br>CuL 6.25<br>CuH_4L_2 49.15<br>CuH_3L_2 41.17<br>CuH_2L_2 32.21<br>$CuHL_2$ 21.2<br>CuL_2 10.13						tentative, from Naringenin (27 °C, 70% mix solv.), charges unknown

	HL 11.34			FeL 14.12				tentative, from 5-hydroxy-flavone (20 °C, 50% mix solv.), charges unknown
Nicotinamide adenine dinucleotide phosphate (NADPH)	HL 9.95<br>H_2L 14.63	CuL 7.70<br>CuL_2 13.26		FeL 9.32<br>FeL_2 18.37		MnL 4.25	ZnL 6.92<br>ZnL_2 13.77	tentative, from adenine, charges unknown
Nicotinamide mononucleotide	HL 6.23	CuL 2.83				MnL 2.21	ZnL 2.13	tentative, from glycerol-1-phosphate, charges unknown
Nitecapone	HL^- 11.40<br>H_2L 17.87	CuL 12.30<br>CuL_2^{2-} 22.33		FeL^+ 15.71<br>FeL_2^- 28.92		MnL 7.22<br>MnL_2^{2-} 12.5	ZnL 8.64<br>ZnL_2^{2-} 15.80	tentative, from 3-nitro-catechol
	(30 °C)<br>HL^- 10.67<br>H_2L 17.19				(30 °C)<br>FeL 13.53<br>FeL_2^{2-} 23.52			tentative, from 4-nitro-catechol
Nordihydroguaiaretic acid	HL 12.8<br>H_2L 22.17	CuL 13.76<br>CuL_2 24.51		(22 °C)<br>FeL 20.05<br>FeL_2 34.71<br>FeL_3 43.75	(1.0 M)<br>FeHL 16.57<br>FeL 7.948<br>FeL_2 13.488	MnL 7.52<br>MnL_2 13.22	ZnL 9.50<br>ZnL_2 17.20	tentative, from catechol, charges unknown
Oleuropein	HL^- 12.8<br>H_2L 22.17	CuL 13.76<br>CuL_2^{2-} 24.51		(22 °C)<br>FeL^+ 20.05<br>FeL_2^- 34.71<br>FeL_3^{3-} 43.75	(1.0 M)<br>$FeHL^+$ 16.57<br>FeL 7.948<br>FeL_2^{2-} 13.488	MnL 7.52<br>MnL_2^{2-} 13.22	ZnL 9.50<br>ZnL_2^{2-} 17.20	tentative, from catechol
Opicapone	HL^- 11.40<br>H_2L 17.87	CuL 12.30<br>CuL_2^{2-} 22.33		FeL^+ 15.71<br>FeL_2^- 28.92		MnL 7.22<br>MnL_2^{2-} 12.5	ZnL 8.64<br>ZnL_2^{2-} 15.80	tentative, from 3-nitro-catechol
	(30 °C)<br>HL^- 10.67<br>H_2L 17.19				(30 °C)<br>FeL 13.53<br>FeL_2^{2-} 23.52			tentative, from 4-nitro-catechol
P7C3	HL^+ 9.54<br>H_2L^{2+} 17.59	CuL^{2+} 8.37<br>CuL_2^{2+} 14.77<br>$Cu_2H_{-2}L_2^{2+}$ 10.25					(30 °C, 1 M)<br>ZnL^{2+} 4.60<br>ZnL_2^{2+} 9.02	tentative, from 1,3-diamino-propane-2-ol (0.2 M)
PBF-509	(0.0 M)<br>HL 9.94<br>H_2L 16.80		(0.3 M)<br>CuL_2 11.4		FeL 4.34<br>FeL_2 7.65<br>FeL_3 9.70	MnL 2.77<br>MnL_2 4.87<br>MnL_3 5.79	(0.0 M)<br>ZnL 5.77<br>ZnL_2 10.83	tentative, from 1,2-diamino-ethane (1.0 M), charges unknown
PBT2	HL 9.49<br>H_2L^+ 15.97	CuL^+ 13.61<br>CuL_2 19.56						data from [Sgarlata 2018]
	HL 9.684<br>H_2L 14.70		(50% mix solv., 0.3 M)<br>CuL_2 14.7	FeL 13.69<br>FeL_2 26.3<br>FeL_3 36.9	(20 °C, 0.01 M)<br>FeL 8.0<br>FeL_2 15.0	MnL 7.85<br>MnL_2 14.40	ZnL 8.52<br>ZnL_2 15.84	tentative, from 8-hydroxy-quinoline, charges unknown
PBT434	HL 9.685<br>H_2L^+ 15.804	CuL^+ 12.42<br>CuL_2 19.33		FeL^{2+} 11.86<br>FeL_2^+ 15.30	FeL^+ 7.06<br>FeL_2 11.06		ZnL^+ 8.57<br>ZnL_2 16.54	[Finkelstein 2017]
Petunidin	HL 12.8<br>H_2L 22.17	CuL 13.76<br>CuL_2 24.51		(22 °C)<br>FeL 20.05	(1.0 M)<br>FeHL 16.57	MnL 7.52<br>MnL_2 13.22	ZnL 9.50<br>ZnL_2 17.20	tentative, from catechol, charges unknown

				FeL_2 34.71<br>FeL_3 43.75	FeL 7.948<br>FeL_2 13.488			
Phenothiazine 2Bc (n=0)	(0.0 M)<br>HL^{+} 9.94<br>H_2L^{2+} 16.80	(0.5 M)<br>CuL^{2+} 10.60<br>CuL_2^{2+} 19.75	(0.3 M)<br>CuL_2^{+} 11.4		FeL^{2+} 4.34<br>FeL_2^{2+} 7.65<br>FeL_3^{2+} 9.70	MnL^{2+} 2.77<br>MnL_2^{2+} 4.87<br>MnL_3^{2+} 5.79	(0.0 M)<br>ZnL^{2+} 5.77<br>ZnL_2^{2+} 10.83	tentative, from 1,2-diamino-ethane (1.0 M)
Phenothiazine 2Bc (n=1)	HL^{+} 10.77<br>H_2L^{2+} 19.62	CuL^{2+} 9.77<br>CuL_2^{2+} 16.94					(20 °C)<br>$ZnHL^{3+}$ 14.96<br>$ZnHL_2^{3+}$ 20.22	tentative, from 1,3-diamino-propane
Phenylhydroxamates	HL 8.63	CuL 7.07<br>CuL_2 12.88		FeL 11.25<br>FeL_2 21.60	(37 °C, 30% mix solv.)<br>FeL_2 8.74	MnL 3.49<br>MnL_2 6.98	ZnL 4.95<br>ZnL_2 9.21	tentative, from benzohydroxamic acid, charges unknown
Piceatannol	HL 12.8<br>H_2L 22.17	CuL 13.76<br>CuL_2 24.51		(22 °C)<br>FeL 20.05<br>FeL_2 34.71<br>FeL_3 43.75	(1.0 M)<br>$FeHL$ 16.57<br>FeL 7.948<br>FeL_2 13.488	MnL 7.52<br>MnL_2 13.22	ZnL 9.50<br>ZnL_2 17.20	tentative, from catechol, charges unknown
Pinostrobin (5-hydroxy-7-methoxy-flavone)	HL 11.79	CuL^{+} 9.83						(50% mix solv.)
	HL 7.74			(20 °C, 10% mix solv.)<br>FeL^{+} 12.74<br>FeL_2 23.25				tentative, from 5-hydroxy-chromone
Piperazine-8-OH-quinolone hybrid	HL 9.684<br>H_2L 14.70	CuL 12.10<br>CuL_2 23.00	(50% mix solv., 0.3 M)<br>CuL_2 14.7	FeL 13.69<br>FeL_2 26.3<br>FeL_3 36.9	(20 °C, 0.01 M)<br>FeL 8.0<br>FeL_2 15.0	MnL 7.85<br>MnL_2 14.40	ZnL 8.52<br>ZnL_2 15.84	tentative, from 8-hydroxy-quinoline, charges unknown
Preladenant	(0.0 M)<br>HL 9.94<br>H_2L 16.80	(0.5 M)<br>CuL 10.60<br>CuL_2 19.75	(0.3 M)<br>CuL_2 11.4		FeL 4.34<br>FeL_2 7.65<br>FeL_3 9.70	MnL 2.77<br>MnL_2 4.87<br>MnL_3 5.79	(0.0 M)<br>ZnL 5.77<br>ZnL_2 10.83	tentative, from 1,2-diamino-ethane (1.0 M), charges unknown
Promethazine	HL^{+} 8.49<br>H_2L^{2+} 11.21	CuL^{2+} 8.32						data from [Shoukry 2011]
	(0.0 M)<br>HL^{+} 9.94<br>H_2L^{2+} 16.80		(0.3 M)<br>CuL_2^{+} 11.4		FeL^{2+} 4.34<br>FeL_2^{2+} 7.65<br>FeL_3^{2+} 9.70	MnL^{2+} 2.77<br>MnL_2^{2+} 4.87<br>MnL_3^{2+} 5.79	(0.0 M)<br>ZnL^{2+} 5.77<br>ZnL_2^{2+} 10.83	tentative, from 1,2-diamino-ethane (1.0 M)
Protocatechuic acid	HL^{2-} 13.1<br>H_2L^{-} 21.77<br>H_3L 26.02	$CuHL$ 19.22<br>CuL^{-} 13.95<br>$CuH_{-1}L^{2-}$ 6.32<br>CuL_2^{4-} 25.02<br>Cu_2L^{+} 16.50<br>$Cu_2L_2^{2-}$ 30.00<br>$Cu_2L_3^{5-}$ 41.30		(1.0 M)<br>$FeHL^{+}$ 25.32<br>$FeH_2L_2^{-}$ 46.04<br>FeL_2^{3-} 40.34<br>FeL_3^{6-} 50.91		(1.0 M)<br>MnL^{-} 7.43<br>MnL_2^{4-} 12.64	ZnL^{-} 8.91<br>ZnL_2^{4-} 15.62	(0.2 M)
Protosappanin A	HL 12.8<br>H_2L 22.17	CuL 13.76<br>CuL_2 24.51		(22 °C)<br>FeL 20.05<br>FeL_2 34.71<br>FeL_3 43.75	(1.0 M)<br>$FeHL$ 16.57<br>FeL 7.948<br>FeL_2 13.488	MnL 7.52<br>MnL_2 13.22	ZnL 9.50<br>ZnL_2 17.20	tentative, from catechol, charges unknown

Punicalagin	–	CuL 7.26		FeL 7.00				[Kulkarni 2007]. Values are conditional constants at pH = 7.5, charges unknown
Pyrazolobenzothiazine-based carbothioamides	HL 1.87	CuL 3.30<br>CuL_2 7.87					ZnL 1.34<br>ZnL_2 2.58<br>ZnL_3 3.71	tentative, from thio-semicarbazide (0.5 M), charges unknown
Pyridoxal isonicotinoyl hydrazone (PIH)	HL 10.25<br>H_2L^+ 18.11<br>H_3L^{2+} 22.52<br>H_4L^{3+} 25.47			FeH_4L^{6+} 52.11<br>FeH_2L^{4+} 27.04<br>$FeH_2L_2^{3+}$ 45.3<br>$FeHL_2^{2+}$ 39.25<br>FeL_2^+ 34.0	$FeH_4L_2^{5+}$ 43.2<br>$FeH_2L_2^{2+}$ 32.97			
Pyridoxal isonicotinoyl hydrazone derivatives:<br>PCIH<br>PCTH<br>H2NPH<br>H2PPH	HL 10.25<br>H_2L 18.11<br>H_3L 22.52<br>H_4L 25.47			FeH_4L 52.11<br>FeH_2L 27.04<br>FeH_2L_2 45.3<br>$FeHL_2$ 39.25<br>FeL_2 34.0	FeH_4L_2 43.2<br>FeH_2L_2 32.97			tentative, from PIH(1)
Pyrimidinone 8	(0.0 M)<br>HL 9.94<br>H_2L 16.80	(0.5 M)<br>CuL 10.60<br>CuL_2 19.75	(0.3 M)<br>CuL_2 11.4		FeL 4.34<br>FeL_2 7.65<br>FeL_3 9.70	MnL 2.77<br>MnL_2 4.87<br>MnL_3 5.79	(0.0 M)<br>ZnL 5.77<br>ZnL_2 10.83	tentative, from 1,2-diamino-ethane (1.0 M), charges unknown
Q1<br>Q4	HL 9.684<br>H_2L 14.70	CuL 12.10<br>CuL_2 23.00	(50% mix solv., 0.3 M)<br>CuL_2 14.7	FeL 13.69<br>FeL_2 26.3<br>FeL_3 36.9	(20 °C, 0.01 M)<br>FeL 8.0<br>FeL_2 15.0	MnL 7.85<br>MnL_2 14.40	ZnL 8.52<br>ZnL_2 15.84	tentative, from 8-hydroxy-quinoline, charges unknown
Quercetin	HL^{4-} 11.0<br>H_2L^{3-} 20.77<br>H_3L^{2-} 28.98<br>H_4L^- 35.93<br>H_5L 41.47	CuH_2L^- 28.09<br>$CuHL^{2-}$ 22.22<br>CuL^{3-} 14.85<br>$CuH_{-1}L^{4-}$ 5.53<br>Cu_2L^- 17.37<br>$Cu_2H_{-1}L^{2-}$ 11.62<br>$Cu_2H_{-2}L^{3-}$ 4.09<br>$Cu_2H_{-3}L^{4-}$ –5.09<br>$Cu_2H_{-4}L^{5-}$ –15.31		FeH_2L 43.54<br>$FeHL^-$ 38.59<br>FeL^{2-} 32.30<br>$FeH_{-1}L^{3-}$ 25.54<br>$FeH_{-2}L^{4-}$ 16.66<br>$Fe_2H_{-1}L$ 35.40<br>$Fe_2H_{-2}L^-$ 29.90<br>$Fe_2H_{-3}L^{2-}$ 23.82<br>$Fe_2H_{-4}L^{3-}$ 17.09<br>$Fe_2H_{-5}L^{4-}$ 8.85<br>$Fe_2H_{-6}L^{5-}$ –0.50				(20 °C)
	H_4L^- 9.22<br>H_5L 17.07						ZnH_4L^+ 14.02	tentative, from quercetin (50% mix solv.), charges unknown
Quinoline derivatives as SUMOylation activators	HL^+ 4.04	CuL^{2+} 6.06<br>CuL_2^{2+} 10.79<br>CuL_3^{2+} 14.75		FeL^{3+} 3.0			ZnL^{2+} 2.42	tentative, from 8-amino-quinoline (20 °C)
Radotinib	HL 4.23<br>H_2L 5.55	CuL 9.00<br>CuL_2 14.724	(23 °C, 0.0 M)<br>CuL 4.59	FeL 9.13<br>$FeH_{-1}L$ 6.96	FeL 3.98<br>FeL_2 9.09	MnL 2.62<br>MnL_2 4.62	ZnL 5.13<br>ZnL_2 9.50	tentative, from 2,2'-bipyridine, charges unknown

			CuL_2 9.18	$FeH_{-2}L$ 5.11<br>FeL_2 18.11<br>$FeH_{-1}L_2$ 14.74<br>$FeH_{-2}L_2$ 10.76	FeL_3 12.63	MnL_3 5.72		
Riboflavin	HL^+ 10.58	(35 °C)<br>$CuHL^{3+}$ 14.32<br>CuL^{2+} 6.53			(20 °C, 0.01 M)<br>FeL^{2+} 7.1	(35 °C)<br>$MnHL^{3+}$ 13.82<br>MnL^{2+} 3.72	(35 °C)<br>$ZnHL^{3+}$ 14.26<br>ZnL^{2+} 4.16	
Rifampicin (ASI-3)	HL 10.97<br>H_2L 18.00	CuL 8.98<br>CuL_2 15.58				MnL 6.71<br>MnL_2 11.93	ZnL 7.89<br>ZnL_2 14.23	30 °C, 50% mix solv., charges unknown
	HL 13.00<br>H_2L 18.35		(30 °C)<br>Cu_2L 22.68	FeL 20.6<br>FeL_2 33.50	(27 °C)<br>FeL 6.00<br>FeL_2 9.45			tentative, from chromotropic acid, charges unknown
Rimonabant	HL 9.81<br>H_2L 15.88	$CuHL$ 12.88<br>CuL 9.56<br>$CuHL_2$ 21.82<br>CuL_2 16.06	CuL 8.87<br>CuH_2L_2 20.13	FeL_2 7.05	(0.058 M)<br>FeL 5.80<br>FeL_2 10.06	(35 °C)<br>$MnHL$ 12.83	$ZnHL$ 11.72<br>ZnL 5.21<br>ZnL_2 10.13	tentative, from histamine, charges unknown
Rosmarinic acid	HL 12.8<br>H_2L 22.17	CuL 13.76<br>CuL_2 24.51		(22 °C)<br>FeL 20.05<br>FeL_2 34.71<br>FeL_3 43.75	(1.0 M)<br>$FeHL$ 16.57<br>FeL 7.948<br>FeL_2 13.488	MnL 7.52<br>MnL_2 13.22	ZnL 9.50<br>ZnL_2 17.20	tentative, from catechol, charges unknown
Rotigotine	HL 10.84<br>H_2L 19.96	(0.17 M)<br>CuL_2 16.28	(20 °C, 1 M)<br>CuL 11.92<br>CuL_2 15.12					tentative, from 3-amino-propane-1-thiol, charges unknown
Rutin	H_4L 9.22<br>H_5L 17.07						ZnH_4L 14.02	tentative, from quercetin (50% mix solv.), charges unknown
	HL 11.0<br>H_2L 20.77<br>H_3L 28.98<br>H_4L 35.93<br>H_5L 41.47	CuH_2L 28.09<br>$CuHL$ 22.22<br>CuL 14.85<br>$CuH_{-1}L$ 5.53<br>Cu_2L 17.37<br>$Cu_2H_{-1}L$ 11.62<br>$Cu_2H_{-2}L$ 4.09<br>$Cu_2H_{-3}L$ –5.09<br>$Cu_2H_{-4}L$ –15.31		FeH_2L 43.54<br>$FeHL^-$ 38.59<br>FeL^{2-} 32.30<br>$FeH_{-1}L^{3-}$ 25.54<br>$FeH_{-2}L^{4-}$ 16.66<br>$Fe_2H_{-1}L$ 35.40<br>$Fe_2H_{-2}L^-$ 29.90<br>$Fe_2H_{-3}L^{2-}$ 23.82<br>$Fe_2H_{-4}L^{3-}$ 17.09<br>$Fe_2H_{-5}L^{4-}$ 8.85<br>$Fe_2H_{-6}L^{5-}$ –0.50				tentative, from quercetin, charges unknown
Salicylate, sodium salt	HL^- 13.44<br>H_2L 16.22	CuL 10.83<br>CuL_2^{2-} 18.88		FeL^+ 16.45<br>FeL_2^- 29.12<br>FeL_3^{3-} 40.89	(20 °C)<br>FeL 6.55<br>FeL_2^{2-} 11.25	MnL 6.10	(35 °C)<br>ZnL 7.10	
Salvianolic acid B	HL 12.8<br>H_2L 22.17	CuL 13.76<br>CuL_2 24.51		(22 °C)<br>FeL 20.05	(1.0 M)<br>$FeHL$ 16.57	MnL 7.52<br>MnL_2 13.22	ZnL 9.50<br>ZnL_2 17.20	tentative, from catechol, charges unknown

				FeL_2 34.71<br>FeL_3 43.75	FeL 7.948<br>FeL_2 13.488			
SCH58261<br>SCH412348	HL 9.35<br>H_2L 14.08	CuL 9.22<br>CuL_2 17.17						tentative, from 4(5)-amino-methyl-imidazole, charges unknown
	(0.0 M)<br>HL 9.94<br>H_2L 16.80		(0.3 M)<br>CuL_2 11.4		FeL 4.34<br>FeL_2 7.65<br>FeL_3 9.70	MnL 2.77<br>MnL_2 4.87<br>MnL_3 5.79	(0.0 M)<br>ZnL 5.77<br>ZnL_2 10.83	tentative, from 1,2-diamino-ethane (1.0 M), charges unknown
Silibinin (silybin) A, B	HL^{2-} 13.67<br>H_2L^- 26.97<br>H_3L 37.27			FeH_3L^{3+} 37.03				(100% org. solv.)
Silydianin	HL^{2-} 13.67<br>H_2L^- 26.97<br>H_3L 37.27			FeH_3L^{3+} 37.03				tentative, from silibinin (100% org. solv.)
ST1535<br>ST4206	HL 9.35<br>H_2L 14.08	CuL 9.22<br>CuL_2 17.17						tentative, from 4(5)-amino-methyl-imidazole, charges unknown
	(0.0 M)<br>HL 9.94<br>H_2L 16.80		(0.3 M)<br>CuL_2 11.4		FeL 4.34<br>FeL_2 7.65<br>FeL_3 9.70	MnL 2.77<br>MnL_2 4.87<br>MnL_3 5.79	(0.0 M)<br>ZnL 5.77<br>ZnL_2 10.83	tentative, from 1,2-diamino-ethane (1.0 M), charges unknown
Staurosporine	(0.0 M)<br>HL 9.94<br>H_2L 16.80	(0.5 M)<br>CuL 10.60<br>CuL_2 19.75	(0.3 M)<br>CuL_2 11.4		FeL 4.34<br>FeL_2 7.65<br>FeL_3 9.70	MnL 2.77<br>MnL_2 4.87<br>MnL_3 5.79	(0.0 M)<br>ZnL 5.77<br>ZnL_2 10.83	tentative, from 1,2-diamino-ethane (1.0 M), charges unknown
Stemazole	HL 1.87	CuL 3.30<br>CuL_2 7.87					ZnL 1.34<br>ZnL_2 2.58<br>ZnL_3 3.71	tentative, from thio-semicarbazide (0.5 M), charges unknown
Sulfuretin	HL 12.8<br>H_2L 22.17	CuL 13.76<br>CuL_2 24.51		(22 °C)<br>FeL 20.05<br>FeL_2 34.71<br>FeL_3 43.75	(1.0 M)<br>FeHL 16.57<br>FeL 7.948<br>FeL_2 13.488	MnL 7.52<br>MnL_2 13.22	ZnL 9.50<br>ZnL_2 17.20	tentative, from catechol, charges unknown
Tannic acid	–	CuL 3.84		Fe_4L 17.45			ZnL 3.93	[Sungur 2008] Unknown temperature and ionic strength; values are conditional constants at pH=8; charges unknown
Tanshinol	HL 12.8<br>H_2L 22.17	CuL 13.76<br>CuL_2 24.51		(22 °C)<br>FeL 20.05<br>FeL_2 34.71<br>FeL_3 43.75	(1.0 M)<br>FeHL 16.57<br>FeL 7.948<br>FeL_2 13.488	MnL 7.52<br>MnL_2 13.22	ZnL 9.50<br>ZnL_2 17.20	tentative, from catechol, charges unknown
Taurine	HL 8.93<br>H_2L^+ 10.61	CuL^+ 3.56<br>CuL_2 6.52				MnL_2 5.28	ZnL_2 5.00	data (20 °C) from [Petrova 2013]
Taxifolin	HL^{3-} 11.68	CuH_3L^+ 36.6						data from [Teixeira 2005]

	H_2L^{2-} 22. 63<br>H_3L^{-} 31.52<br>H_4L 38.2	CuH_2L 31.13<br>$CuHL^{-}$ 25.65<br>CuL^{2-} 18.72<br>$CuH_4L_2^{2-}$ 24.1<br>Cu_2L 23.69						
	HL 11.34			FeL 14.12				tentative, from 5-hydroxy-flavone (20 °C, 50% mix solv.), charges unknown
Tectorigenin	HL 11.34	CuL 14.12		FeL 14.12				tentative, from 5-hydroxy-flavone (20 °C, 50% mix solv.), charges unknown
Tetracycline	HL^{-} 9.57<br>H_2L 17.39<br>H_3L^{+} 20.74	CuL 7.8<br>CuL_2^{2-} 12.80		FeL^{+} 9.9<br>FeL_2^{-} 18.50<br>FeL_3^{3-} 25.3	FeL 5.3<br>FeL_2^{2-} 9.30	MnL 4.4	ZnL 4.9	(20 °C, 0.01 M)
Tolcapone (ASI-7)	HL^{-} 11.40<br>H_2L 17.87	CuL 12.30<br>CuL_2^{2-} 22.33		FeL^{+} 15.71<br>FeL_2^{-} 28.92		MnL 7.22<br>MnL_2^{2-} 12.5	ZnL 8.64<br>ZnL_2^{2-} 15.80	tentative, from 3-nitro-catechol
	(30 °C)<br>HL^{-} 10.67<br>H_2L 17.19				(30 °C)<br>FeL 13.53<br>FeL_2^{2-} 23.52			tentative, from 4-nitro-catechol
Tozadenant	HL 7.90						ZnL 7.33<br>ZnL_2 14.10	tentative, from 2-amino-thiophenol (50% mix solv.), charges unknown
Transilitin	HL 12.8<br>H_2L 22.17	CuL 13.76<br>CuL_2 24.51		(22 °C)<br>FeL 20.05<br>FeL_2 34.71<br>FeL_3 43.75	(1.0 M)<br>FeHL 16.57<br>FeL 7.948<br>FeL_2 13.488	MnL 7.52<br>MnL_2 13.22	ZnL 9.50<br>ZnL_2 17.20	tentative, from catechol, charges unknown
o-Trensox	HL^{5-} 8.62<br>H_2L^{4-} 16.80<br>H_3L^{3-} 24.24<br>H_4L^{2-} 30.6<br>H_5L^{-} 33.61<br>H_6L 36.16<br>H_7L^{+} 37.99	CuH_3L^{-} 38.16<br>CuH_2L^{2-} 35.60<br>$CuHL^{3-}$ 30.99<br>CuL^{4-} 24.20		FeH_5L^{2+} 42.2<br>$FeHL^{2-}$ 36.5<br>FeL^{3-} 30.9			ZnH_3L^{-} 32.76<br>ZnH_2L^{2-} 29.83<br>$ZnHL^{3-}$ 27.06<br>ZnL_4^{-} 23.10	
2', 3', 4' Trihydroxyflavone	HL^{-} 10.85<br>H_2L 19.61	CuL 12.54<br>CuL_2^{2-} 23.01			FeL 12.88<br>FeL_2^{2-} 23.66	MnL 6.41<br>MnL_2^{2-} 10.82	ZnL 8.41<br>ZnL_2^{2-} 14.87	tentative, from pyrogallol (30 °C)
2,3,3-Trisphosphonate	HL 10.17<br>H_2L 17.08<br>H_3L 19.79	CuHL 16.95<br>CuL 13.29<br>CuH_2L_2 33.22<br>CuL_2 23.98<br>Cu_2HL 21.74<br>Cu_2L 18.54		FeL 19.9<br>FeL_2 26.6	FeHL 16.77<br>FeL 12.6<br>FeH_2L_2 32.24<br>FeL_2 18.8<br>Fe_2HL 19.27<br>Fe_2L 15.4		ZnHL 17.67<br>ZnL 13.99<br>ZnH_2L_2 33.88<br>ZnL_2 20.55<br>Zn_2HL 21.4<br>Zn_2L 18.16	tentative, from methane-diphosphonic acid, charges unknown

V81444	HL 8.93<br>H_2L 10.87	CuL 9.69<br>CuL_2 16.90	(50% mix solv.)<br>CuL_2 10.66		(0.15 M)<br>FeL 4.105<br>FeL_2 7.441<br>FeL_3 10.116	(20 °C)<br>MnL 2.66	ZnL 5.2	tentative, from 2-(amino-methyl) pyridine, charges unknown
VAS3947<br>VAS2870	HL 10.69<br>H_2L 18.87	(0.26 M)<br>CuL_2 16.74	(20 °C, 1.0 M)<br>CuL 10.80<br>CuL_2 13.50				ZnL 8.77<br>$ZnH_{-1}L$ 1.94<br>ZnL_2 15.72	tentative, from 2-amino-ethanethiol, charges unknown
Verbascoside	HL 8.72<br>H_2L 13.13	CuHL 10.46<br>CuL 6.02<br>$CuH_{-1}L$ 0.25<br>$Cu_2H_{-1}L$ 3.54<br>$Cu_2H_{-3}L_3$ 0.97<br>$Cu_3H_{-2}L_2$ 7.41			FeL 3.86<br>$FeH_{-1}L$ –3.83<br>$FeH_{-1}L_2$ –0.36<br>$FeH_{-2}L_3$ –6.14<br>Fe_2L 6.69	$MnH_{-1}L$ –4.88<br>$MnH_{-2}L$ –15.55	ZnL 2.99<br>$ZnH_{-1}L$ –3.03<br>$ZnH_{-1}L_2$ –0.39<br>$ZnH_{-2}L_2$ –8.21<br>$ZnH_{-2}L_3$ –5.51	tentative, from caffeic acid, charges unknown
	HL 12.8<br>H_2L 22.17			(22 °C)<br>FeL 20.05<br>FeL_2 34.71<br>FeL_3 43.75				tentative, from catechol (1 M), charges unknown
WIN 55,212-2	(0.0 M)<br>HL^+ 9.94<br>H_2L^{2+} 16.80	(0.5 M)<br>CuL^{2+} 10.60<br>CuL_2^{2+} 19.75	(0.3 M)<br>CuL_2^+ 11.4		FeL^{2+} 4.34<br>FeL_2^{2+} 7.65<br>FeL_3^{2+} 9.70	MnL^{2+} 2.77<br>MnL_2^{2+} 4.87<br>MnL_3^{2+} 5.79	(0.0 M)<br>ZnL^{2+} 5.77<br>ZnL_2^{2+} 10.83	tentative, from 1,2-diamino-ethane (1.0 M)
WR-1065	HL^+ 10.139<br>H_2L^{2+} 19.30	$CuHL^{3+}$ 14.805<br>CuL^{2+} 10.035<br>$CuH_{-1}L^+$ 0.865<br>$CuHL_2^{3+}$ 22.6<br>CuL_2^{2+} 12.90						tentative, from 1,6-diamino-3-thia-hexane (0.5 M)
	HL 10.69<br>H_2L 18.87		(20 °C, 1.0 M)<br>CuL 10.80<br>CuL_2 13.50					tentative, from 2-amino-ethanethiol, charges unknown
	HL^+ 10.77<br>H_2L^{2+} 19.62						(20 °C)<br>$ZnHL^{3+}$ 14.96<br>$ZnHL_2^{3+}$ 20.22	tentative, from 1,3-diamino-propane
Zonisamide	HL 12.49<br>H_2L 15.63	CuL 11.47<br>CuL_2 20.73		FeL 11.70<br>FeL_2 22.94			ZnL 12.16	tentative, from 2-(methane-sulfonamido-methyl)pyridine (30 °C, 45% mix solv.), charges unknown

(1) Coordination chemistry similarity is suggested from the data of [Bernhardt 2005]

References for Table 2S

- The determination of stability constants of N-acetyl-L-cysteine chrome, nickel, cobalt and iron complexes by potentiometric method, S Guzeloglu, G Yalcın, M Pekin, Journal of Organometallic Chemistry 568 (1998) 143–147

- Equilibrium Studies of Complexes between N‑Acetylcysteine and Divalent Metal Ions in Aqueous Solutions, SP Santoso, IK Chandra, FE Soetaredjo, AE Angkawijaya, Y-H Ju, dx.doi.org/10.1021/je500112y | J. Chem. Eng. Data 2014, 59, 1661−1666
- Complexation equilibria of ambroxol hydrochloride in solution by potentiometric and conductometric methods, AH Naggar, HM Al-Saidi, OA El-Moaty Farghaly, TM Hassan, SZ Mohamed Bortata, European Journal of Chemistry 9 (1) (2018) 49-56
- Structure–property studies on the antioxidant activity of flavonoids present in diet, S Teixeira, C Siquet, C Alves, I Boal, MP Marques, F Borges, JLFC Lima, S Reis, Free Radical Biology & Medicine 39 (2005) 1099 – 1108
- Experimental and Theoretical Study of Zn(II) Complexation with Ceftriaxone, MN Mayakova and VG Alekseev, Russian Journal of Inorganic Chemistry, 2016, Vol. 61, No. 3, pp. 314–316
- Stability of Ferric Complexes with 3-Hydroxyflavone (Flavonol), 5,7-Dihydroxyflavone (Chrysin), and 3',4'-Dihydroxyflavone, MD Engelmann, R Hutcheson, IF Cheng, J. Agric. Food Chem. 2005, 53, 2953-2960
- Iron(III)-chelating properties of the novel catechol O-methyltransferase inhibitor entacapone in aqueous solution. Orama, M; Tilus, P; Taskinen, J; Lotta, T. *J. Pharmac. Sci.* 1997, 86, 827-831, DOI: 10.1021/js960458s
- Spectrophotometric and electrochemical determination of the formation constants of the complexes Curcumin–Fe(III)–water and Curcumin–Fe(II)–water, M Bernabé-Pineda, MT Ramırez-Silva, MA Romero-Romo, E González-Vergara, A Rojas-Hernández, Spectrochimica Acta Part A 60 (2004) 1105–1113
- A Glutathione Derivative with Chelating and in vitro Neuroprotective Activities: Synthesis, Physicochemical Properties, and Biological Evaluation, I Cacciatore, C Cornacchia, E Fornasari, L Baldassarre, F Pinnen, P Sozio, A Di Stefano, L Marinelli, A Dean, S Fulle, ES Di Filippo, RML La Rovere, A Patruno, A Ferrone, V Di Marco, ChemMedChem 2013, 8, 1818 – 1829
- Chelating and antioxidant properties of L-Dopa containing tetrapeptide for the treatment of neurodegenerative diseases, I Cacciatore, L Marinelli, A Di Stefano, V Di Marco, G Orlando, M Gabriele, DMP Gatta, A Ferrone, S Franceschelli, L Speranza, A Patruno, Neuropeptides 71 (2018) 11–20
- A Physico-Chemical Study on the Stability of Metal Complexes of Hinokitiol, K Pan, TM Hseu, 2, 1, 23-36, 1955
- Thermodynamic studies of transition metal complexes with Metformin Hydrochloride drug in 20 % (v/v) ethanol-water mixture, SV Thakur, M Farooqui, SD Naikwade, Der Chemica Sinica, 2012, 3(6):1406-1409
- Naringenin complexes with copper ions: potentiometric studies, K Brodowska, Biotechnol Food Sci 2013, 77 (1), 45-53
- Simple and mixed complexes of copper(II) with 8-hydroxyquinoline derivatives and amino acids: Characterization in solution and potential biological implications. Sgarlata, C; Arena, G; Bonomo, RP; Giuffrida, A; Tabbi, G. *J. Inorg. Biochem.* 2018, 180, 89-100, DOI: 10.1016/j.jinorgbio.2017.12.002.
- The novel compound PBT434 prevents iron mediated neurodegeneration and alpha-synuclein toxicity in multiple models of Parkinson's disease. Finkelstein, DI; Billings, JL; Adlard, PA; Ayton, S; Sedjahtera, A; Masters, CL; Wilkins, S; Shackleford, DM; Charman, SA; Bal, W; Zawisza, IA; Kurowska, E; Gundlach, AL; Ma, S; Bush, AI; Hare, DJ; Doble, PA; Crawford, S; Gautier, ECL; Parsons, J; Huggins, P; Barnham, KJ; Cherny, RA. Acta Neuropathologica Communications, 5, Article Number: 53, 2017
- Complex Formation Reactions of Promethazine Copper(II) and Various Biologically Relevant Ligands. Synthesis, Equilibrium Constants, Spectroscopic Characterization and Biological Activity, AA Shoukry, J Solution Chem (2011) 40:1796–1818 DOI 10.1007/s10953-011-9753-8

- In vitro studies on the binding, antioxidant, and cytotoxic actions of punicalagin. Kulkarni, AP; Mahal, HS; Kapoor, S; Aradhya, SM. *J. Agricul. Food Chem.* 2007, 55, 1491-1500, DOI: 10.1021/jf0626720
- Novel diaroylhydrazine ligands as iron chelators: coordination chemistry and biological activity
- By:Bernhardt, PV; Chin, P; Sharpe, PC; Wang, JYC; Richardson, DR, Journal Of Biological Inorganic Chemistry, 10 (7), 761-777, DOI: 10.1007/s00775-005-0018-0, NOV 2005
- Investigation of complexes tannic acid and myricetin with Fe(III). Sungur, S; Uzar, A. *Spectrochim. Acta A - Mol. Biomo. Spectrosc.* 2008, 69, 225-229, DOI: 10.1016/j.saa.2007.03.038, JAN 2008
- Potentiometric Study of Complexation between Taurine and Metal Ions, YS Petrova, LK Neudachina, Russian Journal of Inorganic Chemistry, 2013, Vol. 58, No. 5, pp. 617–620.

TABLE S3

Ionic product of water and stability constants for hydrolysis products of Cu(II), Cu(I), Fe(III), Fe(II), Mn(II), and Zn(II). The ionic product of water (column marked "H^+") has been taken from Nordin et al (Nordin, J; Persson, P; Nordin, A; Sjoberg, S, Langmuir 1998, 14 (13), 3655-3662) and it is valid at 25 °C and at an ionic strength of NaCl 0.1 M. The formulas in the columns marked "Cu(II)", "Cu(I)", etc. represent the hydrolysis products of each metal ion. Numbers are the corresponding values of logβ (see equation 2 in main text for the definition of β). If not differently specified in the notes, values for metal hydrolysis products have been taken from Baes and Mesmer (Baes, CF; Mesmer, RE, The Hydrolysis of cations, John Wiley and Sons, 1976).

ligand	H^+	Cu(II)	Cu(I)	Fe(III)	Fe(II)	Mn(II)	Zn(II)
hydroxide (OH^-)	H_2O –13.775	$Cu(OH)^+$ –8<br>$Cu(OH)_2$ –17.3<br>$Cu(OH)_3^-$ –27.8<br>$Cu_2(OH)_2^{2+}$ –10.36	–	$Fe(OH)^{2+}$ –2.87[(1)]<br>$Fe(OH)_2^+$ –6.16[(1)]<br>$Fe(OH)_3$ –12.16[(2)]<br>$Fe(OH)_4^-$ –22.16[(2)]<br>$Fe_2(OH)_2^{4+}$ –2.9[(2)]<br>$Fe_3(OH)_4^{5+}$ –6.3[(2)]	$Fe(OH)^+$ –9.3<br>$Fe(OH)_2$ –20.5<br>$Fe(OH)_3^-$ –29.4	$Mn(OH)^+$ –10.59	$Zn(OH)^+$ –8.96<br>$Zn(OH)_2$ –16.9<br>$Zn(OH)_3^-$ –28.4<br>$Zn(OH)_4^{2-}$ –41.2

[(1)] data from Di Marco, VB; Tapparo, A; Bombi, GG, Ann. Chim. 2001, 91, 595.
[(2)] data from Flynn, CM Jr., Chem. Rev. 1984, 84, 31
